# Drinking water and the implications for gender equity and empowerment: A systematic review of qualitative and quantitative evidence

**DOI:** 10.1016/j.ijheh.2022.114044

**Published:** 2022-11-14

**Authors:** Kimberly De Guzman, Gabriela Stone, Audrey R. Yang, Kristen E. Schaffer, Shelton Lo, Rola Kojok, Colette R. Kirkpatrick, Ada G. Del Pozo, Tina T. Le, Lindsey DePledge, Elizabeth L. Frost, Georgia L. Kayser

**Affiliations:** a Department of Family Medicine and Public Health, University of California, San Diego, United States; b Department of Global Health, University of California, San Diego, United States; c T.H. Chan School of Public Health, Harvard University, 677 Huntington Avenue, Boston, MA, 02115, USA; d Department of Health Promotion and Behavioral Science, Public Health Program, San Diego State University, San Diego, CA, United States; e Department of Sociomedical Sciences, Columbia University Mailman School of Public Health, New York, NY, United States; f London School of Economics, United Kingdom; g School of Public Health, San Diego State University, The Herbert Wertheim School of Public Health and Human Longevity Science, University of California, San Diego, La Jolla, CA, USA; h The Herbert Wertheim School of Public Health and Human Longevity Science, University of California, San Diego, La Jolla, CA, USA

**Keywords:** Gender Equity and Empowerment, Water, Sanitation and Hygiene, WaSH, women, girls, systematic review, water quality

## Abstract

**Background:**

Safe drinking water is a fundamental human right, yet more than 785 million people do not have access to it. The burden of water management disproportionately falls on women and young girls, and they suffer the health, psychosocial, political, educational, and economic effects. While water conditions and disease outcomes have been widely studied, few studies have summarized the research on drinking water and implications for gender equity and empowerment (GEE).

**Methods:**

A systematic review of primary literature published between 1980 and 2019 was conducted on drinking water exposures and management and the implications for GEE. Ten databases were utilized (EMBASE, PubMed, Web of Science, Cochrane, ProQuest, Campbell, the British Library for Development Studies, SSRN, 3ie International Initiative for Impact Evaluation, and clinicaltrials.gov). Drinking water studies with an all-female cohort or disaggregated findings according to gender were included.

**Results:**

A total of 1280 studies were included. GEE outcomes were summarized in five areas: health, psychosocial stress, political power and decision-making, social-educational conditions, and economic and time-use conditions. Water quality exposures and implications for women’s health dominated the literature reviewed. Women experienced higher rates of bladder cancer when exposed to arsenic, trihalomethanes, and chlorine in drinking water and higher rates of breast cancer due to arsenic, trichloroethylene, and disinfection byproducts in drinking water, compared to men. Women that were exposed to arsenic experienced higher incidence rates of anemia and adverse pregnancy outcomes compared to those that were not exposed. Water-related skin diseases were associated with increased levels of psychosocial stress and social ostracization among women. Women had fewer decision-making responsibilities, economic independence, and employment opportunities around water compared to men.

**Conclusion:**

This systematic review confirms the interconnected nature of gender and WaSH outcomes. With growing attention directed towards gender equity and empowerment within WaSH, this analysis provides key insights to inform future research and policy.

## 1. Introduction

Access to safe drinking water is a fundamental human right; however, the World Health Organization (WHO) and United Nations Children’s Fund (UNICEF) report that more than 785 million people do not have access to safe drinking water (United Nations Children’s Fund and World Health Organization, 2019). Globally, 29% of the population does not have access to a safely managed water source (United Nations Children’s Fund and World Health Organization, 2019). Inadequate access to WaSH services is responsible for 9.1% of the global disease burden and 6.3% of all deaths worldwide (Prüss-Üstün et al., 2008). Additionally, barriers to safe drinking water often lead to various negative health outcomes such as diarrhea, cholera, trachoma, typhoid, shigellosis, and malaria (Beer et al., 2015; Bisung and Elliott, 2017; Hunter et al., 2010; Moura et al., 2019; Mourad et al., 2019; Sengupta, 2013; Tomberge et al., 2021). A global study in 2014 of 145 countries concluded that WaSH-related diarrheal deaths accounted for 1.5% of the total disease burden, 58% of all diarrheal diseases, and 9% of all deaths for children younger than 5 years old (Liu et al., 2012; Prüss-Ustün et al., 2014).

Women and girls are disproportionately affected by inadequate water access because they are largely responsible for household water management (Graham et al., 2016; Kayser et al., 2019; Pouramin et al., 2020; Tomberge et al., 2021) When water sources are not readily accessible at the home, women and girls are responsible for collecting water in 4 out of 5 households worldwide (United Nations Children’s FundWorld Health Organization, 2019). Compared to men, women experience many negative WaSH-related health outcomes, some of which have been disaggregated (Stevenson et al., 2012; Wutich and Ragsdale, 2008).Women and girls account for a higher number of deaths due to diarrheal diseases and higher disability adjusted life years (DALYs) caused by inadequate hygiene (Prüss-Ustün et al., 2019; Pal et al., 2018). Contaminated drinking water and water carriage can induce complications during pregnancy, increase perinatal health issues, negatively affect menstrual health, and increase the incidence of reproductive tract infections in women (Ademas et al., 2020; Gall et al., 2015; Geere et al., 2018a; Kayser et al., 2019).

While WaSH-related health inequities have been widely studied, relatively few studies have evaluated how drinking water impacts gender inequities (Kayser et al., 2019). Among studies that have explored the impact of drinking water on gender, the focus has been on water fetching, sanitation, and sexual violence. Women must travel long distances to retrieve drinking water and find a private place to openly defecate due to a lack of proper sanitation facilities (Sommer et al., 2015; Kayser et al., 2021). This puts them at a much higher risk of being physically assaulted, abused, or harassed (Sommer et al., 2015; Kayser et al., 2021). Among the one in three women that suffer from gender-based violence (World Health Organization, 2021), many attribute their struggle to access adequate WaSH services as a contributing factor (Sommer et al., 2015).

Women often suffer the social-educational and economic ramifications associated with finding and accessing safe drinking water (Stevenson et al., 2012, United Nations Children’s Fund, 2016). According to UNICEF, one in five girls of primary-school age are not in school, compared to one in six boys (United Nations, 2007). Young girls are often taken out of school to help manage the household while young boys are allowed to continue their education (House et al., 2012; UNICEF and WHO, 2019). Additionally, reported school absences increase when girls are menstruating due to ‘inadequate WaSH facilities at school (House et al., 2012; Goodman and Norden, 2005). The lack of education regarding proper menstrual hygiene and the presence of cultural stigma causes girls to miss up to one week of school per month (House et al., 2012, Goodman and Norden, 2005). Studies suggest that such school absences contribute to high drop-out rates for girls (Vanneste et al., 2016).

This systematic review evaluates the current state of the literature regarding drinking water management and exposures and gender equity and empowerment outcomes (GEE). Drinking water management and exposures include elements of accessibility, quality, quantity, reliability, continuity (Kayser et al., 2013). GEE is defined as the association between gender and self-determination (United Nations Development Programme, 2005). Specific GEE outcomes included: psychosocial stress, political power and decision-making, health outcomes, social-educational conditions, and economic conditions. This systematic review includes both quantitative and qualitative published literature. The review assesses the relationship between drinking water and GEE outcomes and highlights areas where future research is needed. The overarching goal is to provide awareness of the connection between drinking water and GEE in order to benefit the health and wellbeing of women and girls, globally.

## 2. Methods

This study was conducted in accordance with the International Prospective Register of Systematic Reviews (PROSPERO) guidelines and the Preferred Reporting Items for Systematic Reviews and Meta-Analyses (PRISMA) statement. Prospero registration number is CRD42021198202.

### 2.1. Search strategy

Our search strategy identified published studies in the following databases – EMBASE, PubMed, Web of Science, Cochrane, ProQuest, Campbell, the British Library for Development Studies, SSRN, 3ie International Initiative for Impact Evaluation, and clinicaltrials.gov. Key terms used in the search included *“*water OR sanitation OR hygiene OR WaSH”, “drinking-water OR drinking”, and “gender OR women OR girl OR girls OR woman OR female OR females”. While search terms remained the same, some variability in search term capabilities between databases existed. The primary objective was to identify existing research studies that focused on the relationship between drinking water and gender equity and empowerment.

### 2.2. Inclusion criteria

**Fig. 1.**
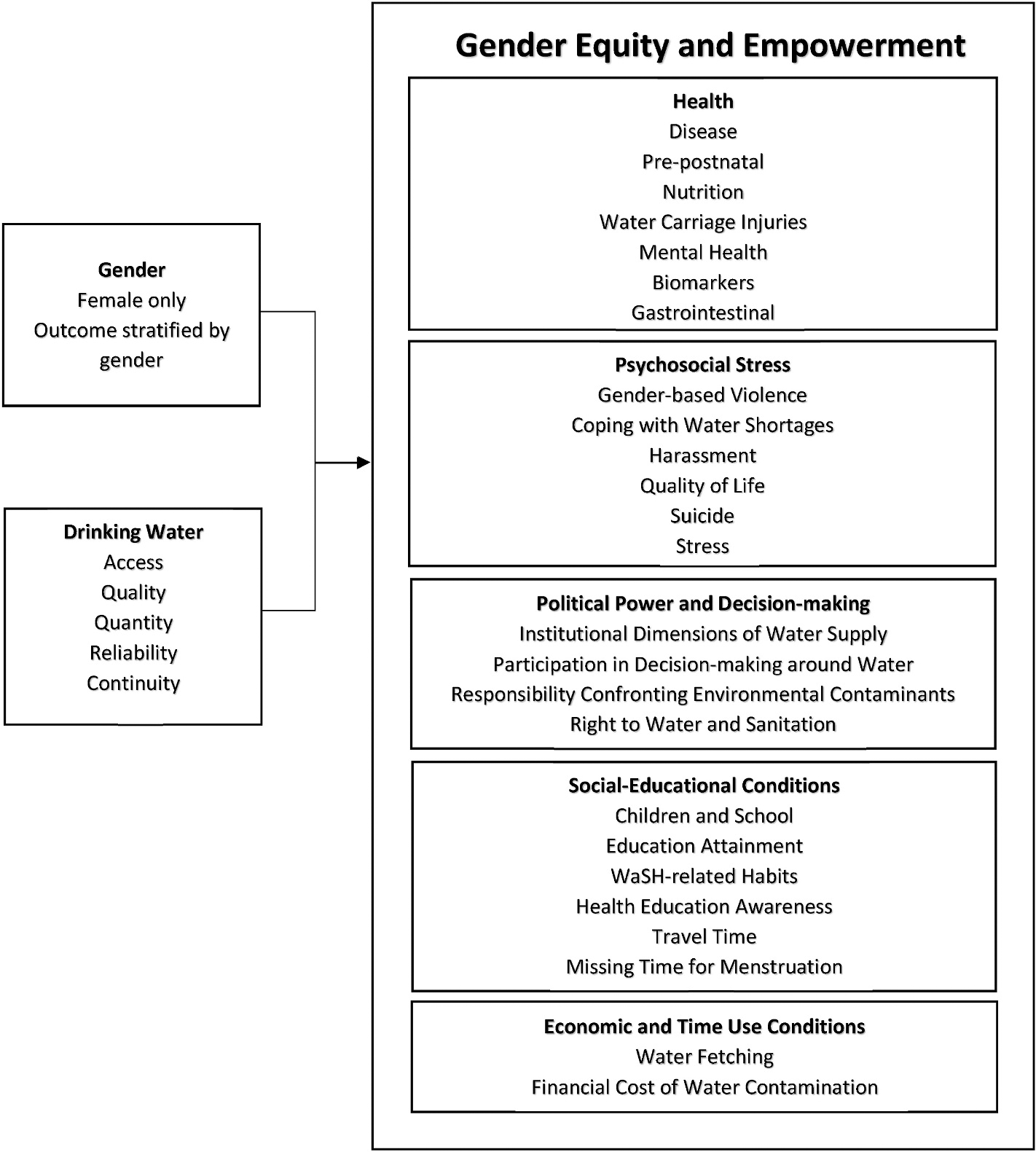
Study schematic for drinking water and gender equity and empowerment.

Inclusion criteria included peer-reviewed published studies conducted between January 1, 1980–September 30, 2019, written in the English language. Quantitative and qualitative studies were included. Studies were required to explicitly consider a gendered outcome, as demonstrated by a female study population or gender-stratified study outcomes. Additionally, drinking water components, defined as water access, quality, quantity, reliability, or continuity were clearly evaluated. Duplicate articles were removed after the database search was completed. The methodological approach used in this study is outlined in Fig. 1.

### 2.3. Exclusion criteria

Studies that did not provide primary data or analysis (i.e., commentaries, systematic review articles, periodicals, theses, dissertations, and meta-analyses), did not consider a population (i.e., case reports), or had small sample sizes (< 10) were excluded for quality, transparency, and to reduce risk of bias. Studies that were missing either a gender or drinking water component were not included in this systematic review. Studies with small sample sizes (< 10) and studies that evaluated outcomes unrelated to GEE or drinking water were excluded. Studies evaluating dental health, exercise, and mineral water were excluded because they were not considered drinking water related.

### 2.4. Additional criteria

Studies that included sanitation and hygiene components were included only if drinking water and gender remained a primary focus of the research question. Sanitation was defined as the adequate treatment of human excreta and sewage, including but not limited to sanitation systems, toiletry availability and cleanliness, feces disposal, and open defecation (UNICEF, 2022). Hygiene refers to the behaviors, habits, and actions of individuals that can improve cleanliness and sanitation conditions and decrease the spread of infectious diseases (UNICEF, 2022). Hygiene practices included handwashing, bathing, and menstrual hygiene. While data was collected on whether a study included hygiene and sanitation measures, neither hygiene nor sanitation were part of the inclusion criteria for the systematic review.

### 2.5. Outcomes

The studies included in the review evaluated the association between drinking water in relation to its implications for GEE. Drinking water’s implications for GEE were separated into five main areas–psychosocial stress, political power and decision-making, health, social-educational conditions, and economic and time use conditions–to create a theoretical framework, which developed through our systematic review and analysis. Each GEE category was subdivided and is listed in Fig. 1.

### 2.6. Data extraction

Three rounds of article review were conducted by the research team. The research team met weekly to discuss study patterns and present articles. The first round analyzed study titles, the second round analyzed study abstracts, and the third round was a full-text review. Using the established exclusion criterion, articles were eliminated at each round.

### 2.7. Title exclusion

During the first round of exclusion, only publication titles were reviewed. To reduce potential bias, five independent reviewers conducted the title exclusion process and inclusion/exclusion criteria was clearly defined. Article titles needed to explicitly mention either drinking water or water-related outcomes. Gender did not have to be explicitly mentioned in the study title, but studies that included gender in the title were included if present. If mentioned in the title, commentaries were excluded. If a study could not be definitively excluded, all five readers independently decided whether to include or exclude the study during weekly meetings. The final decision to include or exclude was dependent on a majority vote.

#### 2.7.1. Abstract exclusion

Following the title exclusion stage, eligible quantitative and qualitative studies were screened based on the article’s abstract where both gender and drinking water were required to be mentioned. To reduce potential bias, five independent reviewers conducted the abstract exclusion. During this stage, commentaries, reviews, and studies with small sample sizes were excluded. All five readers independently decided whether to include or exclude their assigned articles. If a study could not be definitively excluded, the final decision was dependent on a majority vote during weekly meetings.

#### 2.7.2. Full-text review

In the final round of the review process, a full-text analysis was completed. Both gender and drinking water components were required at this stage for the study to be included. The study team designed an intake survey to extract information on study type, gender component, drinking water component, GEE outcome, and method of data analysis. To reduce potential bias, potential for random error, and to ensure quality, each article was reviewed by two blinded readers. Each reviewer independently completed an intake survey for the same article. Any exclusions at the full-text stage needed to provide justification for exclusion in the intake survey. A third senior reviewer resolved any discrepancies between the two intake surveys and made the final decision if the initial reviewers did not agree.

### 2.8. Data analysis

The data from the intake surveys were used for data analysis and exported to Microsoft EXCEL. Percentage-based counts and frequency distributions were calculated for study type, gender component, drinking water component, GEE outcome, and method of data analysis. Drinking water was disaggregated according to water access, quality, quantity, reliability, and continuity. Each GEE subcategory was quantitatively assessed and qualitatively evaluated using thematic analysis.

## 3. Results

**Fig. 2.**
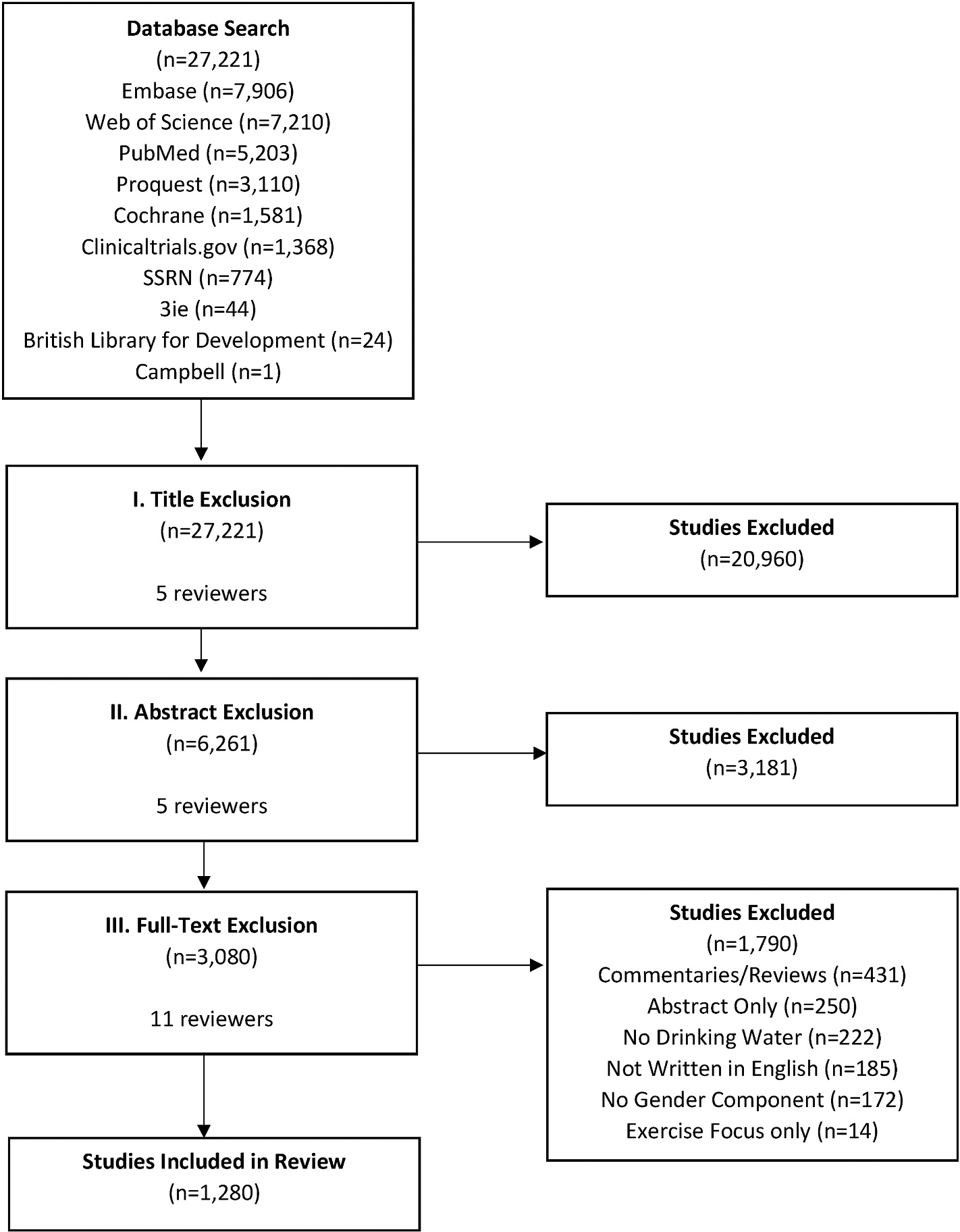
The identification, screening, and inclusion process for the WaSH systematic review.

A total of 1280 studies were included in this systematic review. The full screening process is outlined in Fig. 2. The initial database search yielded 27,221 studies. After reviewing titles, 20,960 studies were excluded. The abstract review excluded an additional 3181 studies. This left a total of 3080 studies eligible for the full-text review. After full-text review, the final 1280 studies were included. Roughly 87% (n = 1107) of the included studies were published between 2000 and 2020 with approximately 34% (n = 439) published within the last five years. The remaining 173 studies were published between 1980 and 1999. A total of 1202 (93.9%) quantitative studies, one (0.08%) qualitative study, and 47 (3.7%) mixed methods studies were included. The most commonly utilized study designs were cross-sectional (n = 581, 45.4%), observational cohort (n = 329, 25.7%), and case control (n = 195, 15.2%).

### 3.1. Drinking water

In each study, drinking water was evaluated according to five different parameters – quality, quantity, accessibility, reliability, continuity. Approximately 85.4% (n = 1093) of all studies focused on water quality by specifically examining chemical contaminants (n = 845, 66%) and biological contaminants (n = 252, 19.7%). Chemical contaminants measured across all the studies are listed in Table 1. Roughly 12% of all studies (n = 157) measured water quantity while only 7.6% (n = 97) evaluated water access. Far fewer studies focused on water reliability (n = 17, 1.3%) and water continuity (n = 14, 1%). Table 2.

### 3.2. Gender

The gender component in each study was categorized as either “study population entirely women” or “data stratified according to gender.” Approximately 31.6% (n = 405) of all studies included only female participants while most studies (n = 875, 68.4%) were stratified by gender. While both drinking water and gender components were essential study inclusion criteria for this systematic review, not all studies found a significant association between the two. Significant associations were quantitatively determined (p < 0.5) in the individual studies. Most studies (n = 854, 66.7%) noted a gender-based association with water and a GEE outcome.

### 3.3. Primary GEE outcome

GEE outcomes from the 1280 studies included in this systematic review were separated into five categories: health outcomes, psychosocial stress, political power/decision-making, social-education conditions, and economic/time use conditions. The intake survey that was used to review full-text articles gathered information about the primary GEE outcomes listed in Table 3.

### 3.4. Health

The health outcomes of study participants were assessed in 91.2% (n = 1167) of all studies. Of those 1167 studies, health was categorized as disease (n = 747, 64%), pre-postnatal conditions (n = 250, 21.4%), gastrointestinal (n = 115, 9.9%), nutrition (n = 113, 9.7%), biomarkers (n = 75, 6.4%), mental health and neurobehavioral conditions (n = 34, 2.9%), and physical injuries related to water-carrying (n = 21, 1.8%). As the most prevalent GEE health outcome, disease was further broken down into 19 categories after the initial categories were established. The association between each disease category and the five drinking water components were determined based on the individual study’s findings, along with the presence of a gender association (Table 4).

#### Cancer –

Of the 747 studies that focused on disease, 191 studies identified cancer as the primary health outcome of interest, largely in relation to water quality. The three most widely studied cancer types were bladder (n = 39, 20.4%), lung (n = 38, 19.9%), and breast (23, 12.0%) due to various chemical contaminants such as arsenic, trihalomethane, disinfection byproducts, and asbestos in drinking water.

Arsenic exposure was found to be the main contaminant linked to incidence of bladder cancer. All but one study found a gender-based association between bladder cancer and chemical contamination of drinking water. In cases where the data were stratified and a gender-based association was identified, 70% of the studies found that women had a greater prevalence and increased risk of developing bladder cancer compared to men when exposed to arsenic, trihalomethanes, and chlorine in their drinking water (Hopenhayn-Rich et al., 1996a; Yeh et al., 2015; Llopis-González et al., 2011; Fernandez et al., 2012; Mallin, 1990; Koivusalo et al., 1997; Chen et al., 1985; Steinmaus et al., 2013; Marshall et al., 2007; Smith et al., 2018).

A gender-based association was found between lung cancer and drinking water quality in roughly 80% of all lung cancer studies due to environmental contaminants such as arsenic, uranium, hexavalent chromium, asbestos, and radioactive material. In cases where the data were stratified and a gender-based association was found, 67% of the studies found that men had a greater prevalence and increased risk of developing lung cancer compared to women due to ingestion of arsenic, asbestos, and radioactive material in drinking water (Chen et al., 1992; Argos et al., 2014; Bean et al., 1982a; Buchet and Lison, 1998; Han et al., 2009; Vinceti et al., 2004; Bean et al., 1982b, Kanarek et al., 1980; Lee et al., 2007; Liu et al., 2011; Hopenhayn-Rich et al., 1998; Chung et al., 2013; Smith et al., 2018).

Drinking water contaminated with arsenic, tetrachloroethylene, or disinfection biproducts was associated with an increased risk of developing breast cancer (Aschengrau et al., 2003; Bean et al., 1982b; Aschengrau et al., 1998; Brody et al., 2006; Gallagher et al., 2010; Garland et al., 1996; Michel-Ramirez et al., 2020; Font-Ribera et al., 2018; Vinceti et al., 2004). Although arsenic contamination was associated with an increased risk of breast cancer, the proposed mechanism involving genetic polymorphisms did not appear to be a contributing factor (Michel-Ramirez et al., 2020).

#### Cardiovascular Disease (CVD), Stroke, and Anemia –

Of the 63 studies that focused on cardiovascular disease, anemia, and stroke, 37 studies evaluated CVD, 18 studies evaluated anemia, and 8 studies evaluated stroke as the primary health outcome. Among the CVD studies, 21 studies found a gender-based association with drinking water contaminated with arsenic, magnesium, and calcium. With regards to children, there were higher rates of arsenic-related congenital heart anomalies and cardiovascular deaths among girls (Marie et al., 2018; Rahman et al., 2013). While women were more likely to develop abnormal cardiac rhythms (QT elongation) due to chronic arsenic exposure in drinking water (Mumford et al., 2007; Chen et al., 2013), there were overall higher rates of sudden cardiac death, ischemic heart disease, and carotid atherosclerosis among men (Bernardi et al., 1995; Nerbrand et al., 1992; Huang et al., 2009; Wang et al., 2007) due to magnesium and calcium contamination in drinking water. Only one study uncovered higher incidence rates of CVD for women due to arsenic contamination (Moon et al., 2013).

Out of the 17 studies that evaluated anemia, 15 found a gender-based association with drinking water contaminated with arsenic, iron, and iodine (Kile et al., 2016; Surdu et al., 2015; Henjum et al., 2012; Merrill et al., 2012). Women routinely had higher prevalence rates of arsenic-related anemia compared to men, with an additional association found between maternal anemia and child anemia (Heck et al., 2008; Kang and Kim, 2019). In studies that focused on arsenic contaminated drinking water and stroke, four studies found a gender-based association. Arsenic-contaminated drinking water was associated with an increased stroke mortality risk among women with no improvement in vascular response after switching to low-arsenic water when compared to men (Rahman et al., 2014; Pi et al., 2005). The two remaining studies found that arsenic concentration in well water was associated with a higher cerebrovascular disease prevalence and mortality rate among men (Chiou et al., 1997; Cheng et al., 2010).

#### Infectious Diseases –

Of the 130 studies that identified an infectious or enteric disease as the primary health outcome from an exposure in water, 22 studies evaluated Toxoplasma gondii, 16 studies evaluated Helicobacter pylori, 12 studies evaluated typhoid, 12 studies evaluated intestinal parasites, and 11 studies evaluated schistosomiasis exposure. Of the 22 Toxoplasma gondii studies, 18 found a gender-based association. However, there were mixed results as to whether women (Fu et al., 2014a; Messier et al., 2009) or men (Sucilathangam and Anna, 2016) had higher Toxoplasma gondii infection rates. Results seemed to vary by study location and population. In the 40 studies that focused on Helicobacter pylori, typhoid, or intestinal parasite infections, no discernible consensus regarding gender-based associations were found. When data were disaggregated by gender, the majority of Helicobacter pylori, typhoid, and intestinal parasite studies found no gender-based association at all. However, in the schistosomiasis studies, nine studies found a gender-based association. Men had an increased risk and higher prevalence rates of schistosomiasis with lower reduction in disease severity after intervention (Lee et al., 2015; Yang et al., 2009, Hajissa et al., 2018).

#### Arsenicosis and Skin Lesions –

A total of 70 studies identified either arsenicosis or skin lesions as the primary health outcome. Fifty-four studies found a gender-based association with arsenic in drinking water and skin lesions. In cases where the data was stratified and a gender-based association was identified, 77% of the studies found that men had a greater prevalence and increased risk of developing skin lesions from arsenicosis compared to women (Adhikari and Ghimire, 2009; Gamble et al., 2005; Nahar, 2009; Hadi and Parveen, 2004; Maharjan et al., 2006; von Ehrenstein et al., 2005; Guha Mazumder et al., 1998; Liao et al., 2007; Argos et al., 2013; Lindberg et al., 2008a; Lindberg et al., 2008b; Yang et al., 2017; Tondel et al., 1999; Ahsan et al., 2006; Watanabe et al., 2001; Argos et al., 2011; Fu, S et al., 2014; Fu et al., 2014b; Smith et al., 2000; Chen et al., 2006). However, several studies reported increased risk of adverse pregnancy outcomes in women with chronic arsenic exposure and arsenicosis including spontaneous abortion, stillbirth, neonatal death, low birthweight, preterm birth, and congenital abnormalities (Chakraborti et al., 2016a; Chakraborti et al., 2016b; von Ehrenstein et al., 2006; Chakraborti et al., 2003; Marie et al., 2018).

#### Musculoskeletal Injuries –

Of the 21 studies that evaluated physical injuries due to water fetching, 18 studies identified women as the main collectors of household drinking water. Three studies identified water fetching as the responsibility of women and men (Sarkar et al., 2015; Subbaraman et al., 2015; Mercer and Hanrahan, 2017). The act of water carriage, the distance traveled to fetch water, and the number of trips necessary to collect a sufficient amount of water were contributing factors to the incidence of musculoskeletal injuries (Zolnikov and Blodgett Salafia, 2016; Narain 2014; Geere et al., 2018b; Mercer and Hanrahan, 2017). To transport containers of water while walking, women balanced containers on their heads, carried containers in their arms, or utilized both methods of carriage simultaneously (Bisung et al., 2015; Stevenson et al., 2012). These methods of water carriage resulted in head and neck pain, axial compression, upper and lower back pain, and joint pain (Geere et al., 2010, 2018b; Narain 2014; Stevenson et al., 2012; Sarkar et al., 2015). Long distances to water sources and frequent number of trips resulted in exhaustion, neck and hip pain, chronic pain, and foot injuries, especially when walking barefoot (Mercer and Hanrahan, 2017; Zolnikov and Blodgett Salafia, 2016; Sarkar et al., 2015; Bisung et al., 2015).

#### Health Outcomes in Children –

Fifty-two studies found a gender-based association between water access and contamination with arsenic, lead, manganese, and biologic agents on health outcomes in children. Health outcomes in children primarily focused on parasitic and waterborne infections such as cryptosporidiosis, dysentery, giardiasis, helicobacter pylori, schistosomiasis, diarrhea (n = 32) and effects on cognitive function and intelligence quotient (IQ) (n = 8), blood pressure (n = 7), and thyroid function (n = 6). In general, studies found that girls had higher infection rates for helminth, helicobacter pylori, urinary tract infections, and diarrheal disease due to biological contaminants in drinking water compared to boys (Onyido et al., 2017; Awuku et al., 2017; Pirinççioğlu et al., 2018; Zincir et al., 2012; Komarulzaman et al., 2019). While boys were more likely to be infected with cryptosporidium, giardiasis, schistosomiasis, and hookworm compared to girls (Al-Shamiri et al., 2010; Nimri, 1994; Bigwan et al., 2013; Abdulkareem et al., 2018; Lee et al., 2015; Forrer et al., 2018), studies found mixed results for intestinal parasitosis with no general consensus whether girls or boys had higher prevalence rates (Shakya et al., 2012; Fentie et al., 2013).

Higher prevalence rates of waterborne diseases in girls or boys were generally ascribed to higher levels of exposures – often through contaminated drinking water sources (Onyido et al., 2017; Nimri, 1994; Awuku et al., 2017; Bigwan et al., 2013; Shakya et al., 2012). The authors explained these different levels of exposures with the different behaviors of boys and girls. Abdulkareem et al. ascribed higher schistosomiasis infection rates in Nigerian boys to more leisure activities in the water, like swimming, fishing, and playing in rivers (2018). Komarulzaman et al. attributed higher rates of diarrheal disease in females to increased exposure through water-fetching (2018).

Longitudinal studies found gender-based associations with arsenic and manganese contamination in drinking water and IQ. A population-based cohort study in Bangladesh (n = 2853) found that arsenic exposure was negatively associated with IQ for girls at age five, but not for boys (Hamadani et al., 2011). The effects of manganese contamination in drinking water on IQ were mixed. Some studies found that manganese exposure improved performance IQ in boys and decreased performance in girls (Bouchard et al., 2018; Dion et al., 2018), while Rahman et al. found that manganese exposure improved cognitive function in girls and did not affect boys (2017).

### 3.5. Psychosocial stress

Psychosocial stress was evaluated in 13% (n = 166) of all studies. Of those 166 studies, psychosocial stress encompassed topics such as quality of life (n = 33, 19.9%), coping with water shortages (n = 26, 15.2%), stress (n = 22, 12.9%), suicide (n = 11, 6.4%), harassment (n = 6, 3.5%), and gender-based violence related to water (n = 5, 2.9%).

#### Gender-based Violence and Harassment –

None of the articles analyzed a direct association between gender-based violence and drinking water as their primary focus. Only one qualitative study in Uganda documented gender-based violence experienced by women when they travel to collect drinking water (Pommells et al., 2018). However, eight articles studied gender-based violence in relation to drinking water-related health outcomes such as anemia, arsenicosis, preterm birth, and low birth weight. Most of these studies took place in South Asian countries (Ahmad et al., 2007; Baker et al., 2018; Devasia, 1998; Gautam et al., 2019; Nerkar et al., 2013). In the 2016 Nepal Demographic and Health Survey, 24% of study participants reported having ever experienced physical or sexual violence with a partner and out of these participants 41% were anemic. However, the association between intimate partner violence and water-related anemia was not statistically significant (Gautam et al., 2019, Ministry of Health, Nepal, 2017). Gender-based violence was only discussed in relation to sanitation, which was not the primary focus of this review.

Women and girls with arsenicosis were more likely than men to suffer from socio-emotional difficulties including rejection, discrimination, and ostracization (Ahmad et al., 2007; Sarker, 2010; Syed et al., 2012). In a cross-sectional study of 750 respondents in Bangladesh, women were more likely to experience extensive socio-emotional and economic problems with development of cutaneous arsenicosis, less likely to receive treatment compared to men, and more likely to be denied care (Ahmad et al., 2007). Common barriers to care include lack of transportation to clinics due to long distances from homes, poor transportation availability, social expectations of traveling with men, and a lack of access to female doctors (Ahmad et al., 2007). Due to religious and cultural practices in Bangladesh, physical examinations and treatments need to be either conducted by female doctors or approved via consent from a husband (Ahmad et al., 2007). Additionally, other studies found that women and girls with physical manifestations of arsenicosis experienced rejection from family members and the community (Sarker, 2010; Ahmad et al., 2007; Syed et al., 2012). Women and girls with arsenicosis were seen as contagious and consequently denied marriages, abandoned by their husbands, isolated from society, and barred from social functions (Sarker, 2010; Ahmad et al., 2007; Syed et al., 2012).

### 3.6. Political power and decision-making power

Women’s political power and decision-making were evaluated in 23.9% (n = 306) of all studies. Of those 306 studies, political power and decision-making included topics such as institutional dimensions of water supply (n = 244, 79.7%), participation in decision-making around water (n = 42, 13.7%), responsibility confronting environmental contaminants (n = 40, 13.1%), and the right to water and sanitation (n = 38, 12.4%).

#### Decision-making Power –

Decision-making demonstrates the power dynamic within households and communities and is grounded in cultural practices, norms, and societal expectations (Barker et al., 2016). Decision-making power was defined as a woman’s ability to make decisions regarding clean water sources, drinking water collection, and the use of water filters (Adams et al., 2018; Kookana et al., 2016; Mohammed and Rilwanu, 2016; Baker et al., 2018). There were 74 studies that specifically focused on women’s decision-making. Most studies were conducted in Africa and India within the last 5 years. Although women’s decision-making was identified as the primary outcome of interest in 17 studies, only 6 directly measured women’s decision-making ability. The remaining articles focused on the impact that study findings had on women’s decision-making ability, rather than measuring the impact women’s decision-making had on drinking water.

Six studies measured women’s individual decision-making ability while confirming the existing limitations on women’s power in their community. For example, a study conducted by Trinies et al. evaluated the decision-making process surrounding the use of water filters in India. The study found that the successful adoption of water filters would require negotiations led by women in the community (Trinies et al., 2011). However, the study concluded that successful negotiations would also have to include family and community members that had more control and the power to make household purchasing decisions (Trinies et al., 2011).

Similarly, women’s low levels of power and decision-making were reinforced in a study conducted in Malawi (Adams et al., 2018). Water User Associations (WUAs) were created to promote women’s participation in decision-making regarding drinking water utilization and irrigation systems (Adams et al., 2018). The study found that formalized, elected committees did not increase women’s ability to make decisions regarding drinking water as women were relegated to lower positions that afforded them little to no real power (Adams et al., 2018). Even when women were acknowledged in community-based negotiations, women’s power and decision-making were not impactful.

### 3.7. Social-educational conditions

Social-educational conditions were evaluated in 51.3% (n = 657) of all studies. Of those 657 studies, social-educational conditions were related to children and school (n = 88, 13.1%), education attainment of the study participants (n = 559, 85.1%), WaSH-related habits (n = 204, 31.1%), health education/awareness (n = 126, 19.2%), travel time to water source (n = 38, 5.8%), and missing time for menstruation (n = 1, 0.2%).

#### Children and School –

Eighty-eight studies evaluated the relationship that drinking water access and water quality had on children at school. Most studies were conducted in South Asia and Africa (n = 42), while 11 studies were conducted in the U.S. and Canada. Approximately 90% (n = 78) of the studies were conducted after the year 2000 with 30 studies published within the last 5 years. The recency of these studies suggests that the impact of drinking water on children and school is an emerging concern.

Water fetching was discussed in 11% (n = 10) of studies and heavily impacted girls’ participation in school when compared to their male counterparts (Kookana et al., 2016; Alhassan and Kwakwa, 2014; Tayeh et al., 1993; Demie et al., 2016; Komarulzaman et al., 2019; Ravichandran and Boopathi, 2005). A cross-sectional study of 500 families in India that looked at school attendance records for children (ages 13–14) found that female students missed school almost twice as often as male students due to domestic responsibilities, including water fetching (Kookana et al., 2016). Female students missed five or more days of school per month, at a rate of 2–10 times more than their male counterparts, when faced with limited water accessibility (Kookana et al., 2016 et al.). Overall, girls spent less time receiving an education and more time collecting water. A mixed-methods study in Ethiopia used multiple stage sampling to collect data on 197 households and found that women and girls spend 3–4 h per day fetching water, which corresponds to the loss of 37–51 days of education per year (Demie et al., 2016). Nearly 50% of girls were not enrolled in school at all due to their domestic responsibilities (Demie et al., 2016). Similarly, a cross-sectional study in India found that girls had more limited options than boys due to their domestic duties (Ravichandran and Boopathi, 2005). During summer months, water fetching was highly dependent on rainwater availability, which led to increased travel time to water sources (Ravichandran and Boopathi, 2005). The burden of water fetching was made easier for young girls if they dropped out of school, as water fetching often conflicted with school hours (Ravichandran and Boopathi, 2005).

### 3.8. Economic and time-use conditions

Economic and time-use conditions were evaluated in 46.2% (n = 591) of all studies. Economic and time-use conditions were defined as the missed economic opportunities due to time spent fetching water (n = 49, 8.3%) and the financial costs associated with contaminated crops, food, and livestock (n = 161, 27%).

#### Water Fetching –

A total of 49 studies focused on water fetching with the majority of studies published within the last 10 years. Studies evaluated the uneven distribution of domestic tasks between men and women and the negative impact of water fetching on women’s participation in the labor market (Hoque and Hope, 2018; Devasia 1998; Nerkar et al., 2013; Alfredo et al., 2014; Demie et al., 2016). The majority of studies identified women and girls as the primary collectors of water with limited contribution from their male counterparts and found that time spent fetching water equated to less time for women to pursue economic ventures (Hoque and Hope, 2018; Devasia 1998; Nerkar et al., 2013; Alfredo et al., 2014; Demie et al., 2016). In Ethiopia, a mixed-methods study of 197 households found that women, on average, traveled greater than 1 km for water, which is equivalent to 5 h collecting water per day (Demie et al., 2016).

When employment opportunities were available in water management, operation and/or maintenance, women were largely responsible for monitoring water distribution for communities that needed to fetch water (Adams et al., 2018). One study in Malawi assessed the economic opportunities available to women in relation to drinking water (Adams et al., 2018). When women were able to find water management jobs, they were relegated to lower-paying positions (ie. Water kiosk attendants), compared to men, and were given lower wages than stated in their contracts (Adams et al., 2018). Women were also given more labor-intensive jobs, such as field inspectors, and had to travel long distances to fetch and monitor and read water meters (Adams et al., 2018).

#### Climate Change and Water –

In Zimbabwe, climate change was responsible for depleting water resources, thereby impacting food reserves and livestock (Phiri et al., 2014). As women are primarily responsible for domestic duties, including water and food preparation, they are affected by climate change’s impact on the water supply. Depleted water resources led to sick livestock, more money spent on medications, and less income generated from sick livestock (Phiri et al., 2014). An additional burden was placed on women due to the climate change-induced migration of the male workforce (Phiri et al., 2014). In Bangladesh, climate change was responsible for the salinization of water sources, river erosion, water logging in homes and loss of agricultural fields resulting in fewer employment opportunities (Beier et al., 2015). Older females were more likely to be negatively impacted by climate-induced changes in employment opportunities and drinking water supplies (Beier et al., 2015). Women were also more likely to suffer negative health outcomes due to food scarcity and agricultural crop destruction (Beier et al., 2015).

## 4. Discussion

While previous studies have evaluated WaSH conditions and its association with gender roles, this is the first systematic review to assess the management of drinking water and drinking water related disease outcomes and its implications for gender equity and empowerment (GEE). A theoretical framework was created that separates drinking water conditions and GEE outcomes, and divides outcomes into categories – health, psychosocial stress, political power and decision-making, social/educational conditions, and economic and time use conditions (Fig. 1). We synthesized evidence on drinking water exposures and GEE outcomes resulting in the largest and most comprehensive gender-focused systematic review on drinking water to date. The results span across multiple domains, alluding to the highly gendered nature of drinking water and the intersectionality between gender equity and empowerment. We found that drinking water and GEE were most commonly studied by assessing water quality, specifically its chemical and microbiological contaminants and the relationship with women and girls’ health. Within the GEE outcome of health, cancer, cardiovascular disease/stroke/anemia, infectious diseases, and arsenicosis were most commonly studied. Water access, reliability, and continuity as they relate to gender were less well studied.

### Health –

Over 90% of full text articles reviewed focused on the association between drinking water exposures and health outcomes. Overall, women had higher incidence rates of bladder cancer due to drinking water contaminated with arsenic, trihalomethanes, and chlorine (Hopenhayn-Rich et al., 1996b; Yeh et al., 2015; González-Weller et al., 2012; Fernandez et al., 2012; Mallin et al., 1990; Koivusalo et al., 1997; Koivusalo et al., 1995; Chen et al., 1985; Steinmaus et al., 2013; Marshall et al., 2007; Smith et al., 2018). Arsenic, trichloroethylene, and disinfection byproducts exposure in drinking water was associated with increased incidence of breast cancer in women (Aschengrau et al., 2003; Bean et al., 1982b; Aschengrau et al., 1998; Brody et al., 2006; Gallagher et al., 2010; Garland et al., 1996; Michel-Ramirez et al., 2020; Font-Ribera et al., 2018; Vinceti et al., 2004). While we found some evidence, more research is needed on arsenic and trihalomethanes and the relationship with breast cancer in women, as the results are mixed (Pullella and Kotsopoulos, 2020; Font-Ribera et al., 2018). Women experienced higher incidence rates of anemia when exposed to drinking water contaminated with arsenic and iodine (Kile et al., 2016; Surdu et al., 2015, Henjum et al., 2012). Conversely, we found that men had higher incidence rates for lung cancer due to exposure to arsenic, asbestos, and radioactive material (Chen et al., 1992; Argos et al., 2014; Bean et al., 1982a, Buchet and Lison, 1998; Han et al., 2009; Vinceti et al., 2004; Bean et al., 1982b; Kanarek et al., 1980; Lee et al., 2007; Hopenhayn-Rich et al., 1998; Chung et al., 2013; Smith et al., 2018). Men also had higher incidence rates of cardiovascular disease due to magnesium and calcium exposure in drinking water (Bernardi et al., 1995; Nerbrand et al., 1992; Huang et al., 2009; Wang et al., 2007).

While water-borne diseases are an important area of research for GEE and drinking water, comprising roughly 10% of our data, few studies disaggregated data by gender. Of those that did, results were often mixed with no consensus regarding Helicobacter pylori, typhoid, and intestinal parasitosis for adult populations. This finding aligns with previous systematic reviews, which have found a lack of WaSH-related water-borne disease studies among adult populations; most focus on children (Pouramin et al., 2020). As drinking water is a heavily gendered issue that disproportionately impacts women and is a known cause of many parasitic, infectious, and diarrheal diseases, we recommend future studies also include adult populations and disaggregate data by gender when evaluating water-related disease incidence (Mourad et al., 2019; Hunter et al., 2001; Beer et al., 2015; Tomberge et al., 2021; Graham et al., 2016). Furthermore, it is important to note the lack of research regarding the impact of water-related parasitic, infectious, and diarrheal diseases on pregnant women and newborns.

One area of health that demonstrates a strong correlation between GEE and drinking water is arsenicosis and skin lesions, with 77% of arsenic studies finding a gender-based association. Women’s prenatal health outcomes are severely impacted by arsenic exposure. Higher rates of spontaneous abortion, stillbirth, neonatal death, low birth weight, preterm birth, and congenital abnormalities have been found among female cohorts that have been exposed to chronic arsenic contamination in drinking water during pregnancy (Chakraborti et al., 2016a; Chakraborti et al., 2016b; von Ehrenstein et al., 2006; Chakraborti et al., 2003; Marie et al., 2018).

Studies suggest that there is a strong correlation between water fetching, water carriage, and the incidence of musculoskeletal injuries in women (Geere et al., 2010; Stevenson et al., 2012; Bisung et al., 2015; Zolnikov and Blodgett Salafia, 2016; Geere et al., 2018b; Narain, 2014). Common injuries included head and neck pain, axial compression, upper and lower back pain, joint pain, hip pain, and foot injuries (Geere et al., 2010; Geere et al., 2018b; Narain, 2014; Stevenson et al., 2012; Mercer et al. 2017; Zolnikov and Blodgett Salafia, 2016; Sarkar et al., 2015; Bisung et al., 2015). Comparatively fewer studies identified both men and women as household water collectors (Sarkar et al., 2015; Subbaraman et al., 2015; Mercer and Hanrahan, 2017). However, of the studies that did, none compared the types of physical injuries suffered by men and women (Sarkar et al., 2015; Subbaraman et al., 2015; Mercer and Hanrahan, 2017). Additionally, only one study evaluated pregnant women as water collectors, but physical injuries were not evaluated (Ghosh et al., 2016). Although water fetching and water carriage have been found to negatively impact the physical health of water collectors, future research should compare the physical injuries suffered by men and women that fetch water and evaluate the impact water fetching and water carriage have on the physical health of pregnant women.

### Psychosocial Stress –

Psychologically, we found substantial evidence that women face the added burden of arsenicosis-related harassment that often does not apply to men (Ahmad et al., 2007; Sarker, 2010; Syed et al., 2012). Even though men are more likely than women to develop skin lesions from arsenic (Adhikari and Ghimire, 2009,; Gamble et al., 2005; Nahar, 2009; Hadi and Parveen, 2004; Maharjan et al., 2005; Maharjan et al., 2006; von Ehrenstein et al., 2005; Guha Mazumder et al., 1998; Liao et al., 2007; Argos et al., 2013; Lindberg et al., 2008b; Yang et al., 2017; Tondel et al., 1999; Ahsan et al., 2006; Watanabe et al., 2001; Argos et al., 2011; Fu et al., 2014b; Smith et al., 2000; Chen et al., 2006), women and girls face social ostracization from the community and are pulled out of school, rejected by their families and husbands, denied health care, and lose employment opportunities due to stigma (Brinkel et al., 2009; Ahmad et al., 2007; Sarker, 2010; Syed et al., 2012). While the physical health impacts of water-related arsenicosis may not be as pronounced for women, the psychological burden disproportionately impacts their quality of life. However, in order to adequately address the ramifications of inequitable treatment, we acknowledge that the basic physiological health and safety needs of women and girls must be met first. In accordance with Maslow’s hierarchy of needs, health and safety must be established before steps can be taken to improve social connections, self-esteem, and empowerment (Lester et al., 1983). Likewise, the GEE outcomes of psychosocial stress, political power and decision-making, social-educational achievement, economic power, and time use cannot be addressed until the social structures women live in are more accepting of women’s empowerment and gender equity. We found little research on psychosocial stress related to water availability, continuity, or reliability of water supply.

None of the studies evaluated the direct association between gender-based violence (GBV) and drinking water, a topic better studied as it relates to sanitation. This may be due to the lack of research that quantifies gender-based violence and harassment as it is associated with drinking water as well as methodological and ethical challenges that arise when conducting research of this nature (Sommer et al., 2015; Cepeda et al., 2021; Caruso et al., 2021). Stigma and shame associated with gender-based violence can negatively impact incident reporting (Barnett et al., 2016). It will be difficult to evaluate the direct association between drinking water, water fetching, gender-based violence, and psychosocial stress without first addressing the need to destigmatize violence against women and developing validated measurement strategies. Recently, there have been efforts to develop quantitative toolkits for gender-based violence in order to uniformly define and measure violence against women (House et al., 2012; Sommer et al., 2015; Raj, 2021). We believe that these are crucial steps towards ensuring that future research can evaluate how and to what extent drinking water and water fetching impact gender-based violence or harassment.

### Political Power and Decision-making –

The existing literature on gender and women’s participation in water management suggests that when women must walk to collect water, women bear an increased burden compared to men (United Nations Children’s Fund, 2016). Furthermore, women’s participation in water planning and decision-making is low compared to men despite evidence that their participation may lead to better outcomes (Adams et al., 2018; Andajani-Sutjahjo et al., 2015). While studies acknowledged the important role women play in household water collection and management, they are not typically in positions of power where they are able to exercise their decision-making power in meaningful ways. While we were interested in assessing how women’s political power and decision-making impacted drinking water conditions, we found that the vast majority of studies focused on how women’s decision-making abilities were impacted by drinking water conditions. For example, women were occupied with water collection duties, which left little time to engage in political matters or offer their insight when decisions needed to be made. While that is an important factor in GEE, it is also critical to study how women’s decision-making about drinking water impacts drinking water quality, continuity, reliability, quantity, and cost and subsequent water-related health outcomes. With so few studies quantitatively measuring women’s decision-making, future research could focus on the social and political factors that affect how and when women make decisions about drinking water usage. Further examination of women’s decision making in water management and how it is associated with water outcomes (quality, continuity, reliability, quantity, and cost) and community health is also needed.

### Social-educational Conditions –

We found that girl’s participation in school was severely impacted by household domestic duties, including water fetching. Young girls were more likely to miss up to two months of schooling per year due to time spent collecting water (Kookana et al., 2016; Demie et al., 2016; Ravichandran and Boopathi, 2005). The studies we reviewed focused solely on the impact water fetching has on young girls’ school absenteeism and did not comprehensively study the long-term implications for school dropout rates. Education research suggests that missed educational opportunities, including missed school, can lead to drop out and impact girls’ life trajectory by limiting financial independence through income-generating endeavors and basic health knowledge acquisition (U.S. Department of Education, 2019). Future water studies should expand on the effects of girls’ school absenteeism while water fetching and dropout on gender roles as they age. Additionally, very few articles mentioned the impact water inequities may have on young girls outside of school. Although most studies did not measure the physical health impact of water collection on young girls, it is likely that young girls experience some of the same water carriage injuries as women that fetch water. These can include physical pain, fatigue, perinatal health problems, uterine prolapse, spinal fractures and dislocations, and cervical compression syndromes, which lead to serious long-term disabilities (Geere et al., 2018a). However, there is limited research regarding the prevalence of these health outcomes in young school-aged girls. This is an important study area for future research due to the degenerative nature of these health problems.

### Economic/Time use Conditions –

Women and girls were identified as the primary water collectors in the household (Hoque and Hope, 2018; Devasia 1998; Nerkar et al., 2013; Alfredo et al., 2014; Adams et al., 2018). Due to the time-consuming nature of water fetching, some women do not have enough time to pursue careers, thereby limiting their economic opportunities (Hoque and Hope, 2018; Devasia, 1998; Nerkar et al., 2013; Alfredo et al., 2014; Demie et al., 2016). The scarcity of water-related jobs for women was further highlighted by the fact that only one study evaluated employment and economic opportunities for women in relation to drinking water (Adams et al., 2018). When women were able to obtain paid work in drinking water operations, they were subjected to physically demanding conditions with lower wages and lower positions than their male colleagues due to gender roles and expectations (Adams et al., 2018).

### Climate Change –

Women can face the financial burden of depleted food and water reserves and ill livestock as a result of climate change-induced alterations in water availability (Phiri et al., 2014; United Nations Women, 2022). As household managers, women suffer the consequences of contaminated and diminished water reserves–they may have to purchase additional food and medication for sick livestock and make less profit selling unhealthy livestock (Phiri et al., 2014). However, most disconcerting is the effect climate change has on the labor market and women’s domestic duties. In some places, climate change has triggered the migration of the male workforce, thereby increasing the workload for women (Phiri et al., 2014). Additionally, droughts and floods caused by climate change can increase drinking water contamination, and lead to increased incidence of household enteric diseases and reduced availability of water supply (Nounkeu et al., 2019; Khodarahimi and DehghaniNikpourian, 2014; Peragallo Urrutia et al., 2012). Women who are tasked with caring for sick household members can lead to an increase in domestic duties and additional stress (Nounkeu et al., 2019; Khodarahimi and DehghaniNikpourian, 2014; Peragallo Urrutia et al., 2012). Women’s roles and responsibilities pertaining to drinking water and management are dependent on the current environment, leaving them vulnerable when environmental change occurs (Devasia 1998; United Nations WomenWatch, 2009).

While the effects of climate change on water and women’s responsibilities and economic independence are likely widespread, only one study evaluated the impact of climate change on drinking water and women’s livelihoods. Additionally, climate change exacerbates the burden of water fetching, especially in rural areas due to decreased water supply, increasing travel distances triggered by water shortages (Sultana, 2014). We believe that research on the economic impact of climate change on women, as it relates to water supply and quality is both important and time sensitive as climate change continues to become a bigger and more pressing problem. If not addressed, the burden on women will only become more prominent.

### Strengths and Limitations –

The results of this systematic review give a broad overview of the existing research at the intersection of drinking water and gender equity and empowerment (GEE). We hope that this may provide a framework and guide areas for future investigations to fill in the gaps in knowledge identified in this review. As a large systematic review, there are both strengths and limitations with this study design. During the study identification process, a broad literature review was conducted using 10 different research databases. Teams of reviewers worked in pairs to analyze each study, thereby ensuring a high level of internal validity, and decreasing risk of bias. We also separated WaSH-related drinking water into five different components (quality, quantity, access, continuity, reliability) and GEE outcomes into five different components (health, psychosocial stress, political power and decision-making, social-educational conditions, economic/time use conditions). The large number of studies included in this systematic review allowed us to uncover and credibly support common trends, while using specific water and GEE criteria to identify detailed associations between drinking water and GEE and point out areas where further research is needed to improve the health and wellbeing of women and girls, globally. However, our review did not find articles or assess the relationship between drinking water and non-binary genders. This remains an important area of study for future research. However, studies spanned a large time frame of 40 years and findings from earlier studies may not fully represent the current state of drinking water quantity, accessibility, continuity, and reliability as it affects GEE, as there is limited research related to gender on these topics. Studies were restricted to those published in the English language, which may have limited the sources available for review. Lastly, as studies were not restricted by study location, general findings of this systematic review may not equally apply to every country.

## 5. Conclusion

Ninety percent of full text studies reviewed studied the association between drinking water quality and health outcomes. Research on the relationship between drinking water and other equity and empowerment outcomes was sparse. Women had higher incidence of breast cancer due to arsenic, trichloroethylene, and disinfection byproduct exposure and greater incidence of bladder cancer due to arsenic, trihalomethane, and chlorine in drinking water. Men had higher incidence rates of lung cancer due to arsenic, asbestos, and radioactive material exposure, and increased incidence of cardiovascular disease due to magnesium and calcium in drinking water. Importantly, while men had an increased risk of developing arsenicosis and skin lesions compared to women, women were subjected to social ostracization and psychosocial stress, denied healthcare, and pulled out of school and work as a result of arsenicosis. Despite how extensive our findings for drinking water and health outcomes were, there was a notable lack of research regarding drinking water contamination and parasitic, infectious, or diarrheal diseases that disaggregated the data by gender or focused on pregnant women and newborns. Additionally, there were few studies that assessed water carriage injuries, particularly among young girls. As poor water-related health outcomes contracted at young ages can progress and worsen into adulthood, it is important to study how drinking water conditions affect vulnerable populations throughout the life course.

Although we found an association between water fetching and school absenteeism, there is less evidence of the subsequent association with school dropout rates among young girls. Research on the longitudinal impact of water fetching and the impact on girls’ economic independence and employment opportunities is sparse. We found that women have poor political power and decision-making ability related to water, few economic opportunities related to water, and limited employment options outside of domestic duties related to water. The lack of economic and decision-making opportunities related to water may also influence women and girls’ efforts to obtain independence and self-actualization. Women may face the threat of gender-based violence while collecting drinking water; however, no research in the review studied this relationship. There is underreporting of gender-based violence due to stigma and shame. Further research should be conducted to measure and ensure the safety of girls and women as it relates to water, globally.

This systematic review underscores many ways in which drinking water and gender inequities intersect while identifying critical areas of future research to improve the health and wellbeing of women and girls now and in the future. Findings from this review help us to understand some strategies and solutions to address gender inequities related to drinking water.

## Supplementary Material

SupplementaryMaterial

Appendix A. Supplementary data

Supplementary data to this article can be found online at https://doi.org/10.1016/j.ijheh.2022.114044.

## Figures and Tables

**Table 1:** Water quality chemical contaminants.

Chemical	n (%)

All Chemicals	845
Arsenic	312 (36.9)
Trace Elements(Ca, Mg, Mn, Fe, Cd)	127 (15.0)
Fluoride	67 (7.9)
Disinfectant by-products(Chlorine, Chlorine by-products, Perchlorate)	58 (6.9)
Nitrate/Nitrite	48 (5.7)
Lead	37 (4.4)
Trihalomethanes	37 (4.4)
PFAs/PFOs/PCE	34 (4.0)
Iodine	32 (3.8)
Lithium	17 (2.0)
Phthalates	4 (0.5)
^a^All other	72 (8.5)

a All other includes: antibiotics, asbestos, BPA, chloroform, herbicides, or unspecified.

**Table 2:** Included continuity and reliability studies.

Publication [Author, Date]	Title	Study Design	Country	Main Findings	Association with GEE outcome	Significant GEE association?

Alfredo et al. (2014)	Fluoride contamination in the Bongo District of Ghana, West Africa: geogenic contamination and cultural complexities	Observational	Ghana	Fluoride concentrations in the region are higher than reported and are inversely related to rainfall; women should be involved in decision-making to adequately treat water	Women’s participation in decision making; Right to water and sanitation	Yes
Alhassan and Kwakwa (2014)	When water is scarce: the perception of water quality and effects on the vulnerable	Cross-sectional	Ghana	Water insecurity increases burdens and gender-specific hazards on women and children and decreases quality of life	Psychosocial stress	Yes
Baguma et al. (2013)	Safe-water shortages, gender perspectives, and related challenges in developing countries: The case of Uganda	Cross-sectional	Uganda	Efficient water management is related to women’s years of water harvesting, family size, and tank operation and maintenance; emphasis should be placed on women-related water management activities	Women’s participation in decision-making; Women’s responsibility in confronting environmental contaminants	Yes
Habi and Harrouz (2015)	Domestic water conservation practices in Tlemcen City (Algeria)	Cross-sectional	Algeria	Informal water rationing due to scarcity leads to increased tension among women in the community to store enough water and large discrepancies in water distribution	Quality of life; Women’s participation in decision-making	Yes
Phiri et al. (2014)	Climate Change Impacts on Rural Based Women: Emerging Evidence on Coping and Adaptation Strategies in Tsholotsho, Zimbabwe	Cross-sectional	Zimbabwe	Impact of climate change affect women’s quality of life leading to water and food insecurity and increased poverty, particularly for those in rural areas	Women’s responsibility in confronting environmental contaminants; Quality of life	Yes
Stoler et al. (2012)	When urban taps run dry: sachet water consumption and health effects in low income neighborhoods of Accra, Ghana	Cross-sectional	Ghana	Neighborhood rationing is predictive of water sachet uptake. Water sachet uptake in low SES leads to better health outcomes and less diarrheal diseases	Women’s responsibility in confronting environmental contaminants; Gastrointestinal	Yes
Khan et al. (2017)	Optimizing household survey methods to monitor the Sustainable Development Goals targets 6.1 and 6.2 on drinking water, sanitation and hygiene: A mixed-methods field-test in Belize	Cross-sectional	Belize	MISC surveys for WASH monitoring should be further refined; safely managed drinking water is underestimated; stored drinking water is more likely to be contaminated	Coping with water shortages; Women’s responsibility in confronting environmental contaminants	Yes
Trudeau et al. (2018)	Water system unreliability and diarrhea incidence among children in Guatemala	Retrospective Cohort	Guatemala	Age, female gender, Spanish language, garbage disposal, and interrupted water service is associated with diarrheal incidence in children	Women’s responsibility in confronting environmental contaminants; Gastrointestinal	Yes
Abu Morad (2004)	Palestinian refugee conditions associated with intestinal parasites and diarrhea: Nuseirat refugee camp as a case study	Case study	Palestine	Intestinal parasites are associated with low socioeconomic status, interrupted water service, drinking water source, drinking water storage methods, and women’s attitude and practice of personal hygiene	Habits (ie. hand washing, open defecation); Women’s responsibility in confronting environmental contaminants	Yes
Morgan et al. (2017)	Water, sanitation, and hygiene in schools: Status and implications of low coverage in Ethiopia, Kenya, Mozambique, Rwanda, Uganda, and Zambia	Cross-sectional	Ethiopia, Kenya, Mozambique, Rwanda, Uganda, Zambia	Basic WaSH deficiencies in rural schools in these countries are associated with low attendance and adverse health outcomes	Children and school	Yes
Khodarahimi and DehghaniNikpourian, 2014	Mental Health and Coping Styles of Rural Residents Affected by Drinking Water Shortage in Fars Province: An Ecopsychological Perspective	Cross-sectional	Iran	Rural residents with water shortages had higher a psychopathology indicator, lower rational coping styles, and higher emotion focused avoidant coping styles	Coping with water shortages	No
Prasad et al. (2018)	Epidemiology and risk factors for typhoid fever in Central Division, Fiji, 2014ñ2017: A case-control study	Case-control	Fiji	Salmonella typhii transmission is attributed to water availability, drinking contaminated water, habits, and poor sanitation facilities	Disease	No
Joshi et al. (2013)	Water and sanitation hygiene knowledge attitude practice in urban slum settings.	Cross-sectional	India	Women and girls are primarily responsible for water collection, particularly from long distances during shortages; most respondents believed contaminated water was safe to drink	Habits (ie. hand washing, open defecation)	Yes

**Table 3:** Primary GEE outcomes for included studies.

Primary GEE Outcome	n (%)

Disease	743 (58.0)
Pre-postnatal	192 (15.0)
Nutrition	60 (4.7)
Gastrointestinal	57 (4.4)
Institutional Dimensions of Water Supply	24 (1.9)
WaSH-related Habits	23 (1.8)
Water Exposure/Water-carrying Injury	21 (1.6)
Women’s Participation in Decision-Making	17 (1.3)
Mental Health	17 (1.3)
Contamination in Crops and Food	16 (1.3)
Socioeconomic Status and Disease Risk	15 (1.2)
Children and School	12 (0.9)
Health Education	11 (0.9)
Right to Water and Sanitation	9 (0.7)
Women’s Responsibility Confronting Environmental Contaminants	5 (0.4)
Education Attainment	5 (0.4)
Coping with Water Shortages	4 (0.3)
Unspecified	52 (4.1)

**Table 4:** Evaluation of disease outcomes with drinking water and gender association.

Disease	*n (%)	^+^Gender Association n (%)	^+^Water Quality n (%)	^+^Water Quantity n (%)	^+^Water Access n (%)	^+^Water Continuity n (%)	^+^Water Reliability n (%)

Cancer	191 (25.6)	137 (71.7)	179 (93.7)	12 (6.3)	1 (0.5)	–	–
Infectious Disease	130 (17.4)	80 (61.5)	126 (96.9)	5 (3.8)	11 (8.5)	2 (1.5)	2 (1.5)
Cardiovascular disease, stroke, anemia	63 (8.4)	40 (63.5)	54 (85.7)	7 (11.1)	1 (16)	–	–
Blood Pressure, Obesity, Cholesterol, Diabetes	47 (6.3)	25 (53.2)	42 (89.4)	5 (10.6)	1 (2.1)	–	–
Skin Lesions	42 (5.6)	35 (83.3)	42 (100)	1 (2.9)	–	–	–
Thyroid Disorders	39 (5.2)	27 (69.2)	39 (100)	2 (5.1)	–	–	–
Liver Disorders	32 (4.3)	21 (65.6)	31 (96.9)	1 (3.1)	1 (3.1)	–	–
Bone Disorders	31 (4.1)	21 (67.7)	31 (100)	1 (3.2)	–	–	–
Arsenicosis	28 (3.7)	19 (67.9)	28 (100)	6 (21.4)	2 (7.1)	–	–
Children	24 (3.2)	20 (83.3)	16 (66.7)	1 (4.2)	4 (16.7)	–	–
Kidney Disorders	21 (2.8)	10 (47.6)	16 (76.2)	4 (19.0)	1 (4.8)	–	–
Genetic	19 (2.5)	15 (78.9)	19 (100)	–	–	–	–
Neurobehavioral	19 (2.5)	11 (57.9)	17 (89.5)	2 (10.5)	–	–	–
Women’s Health	17 (2.3)	12 (70.6)	13 (76.5)	1 (5.9)	–	–	–
Toxicity	8 (1.1)	5 (62.5)	7 (87.5)	2 (25.0)	–	–	–
Respiratory Illness	5 (0.7)	4 (80.0)	5 (100)	–	–	–	–
Gastrointestinal	4 (0.5)	2 (50.0)	3 (75.0)	1 (25.0)	–	–	–
Urinary Disorders	4 (0.5)	3 (75.0)	1 (25.0)	3 (75.0)	–	–	–
All Other	46 (6.1)	32 (69.6)	40 (87.0)	8 (17.4)	3 (6.5)	–	–

* Percentages were calculated using the 747 studies where disease was indicated as a health GEE outcome

+ Percentages were calculated using the total number of studies for the specific disease indicated in that row.
