# Supplementary material for: Drinking water and the implications for gender equity and empowerment: A systematic review of qualitative and quantitative evidence
Source: Int J Hyg Environ Health. Author manuscript; Available in PMC 2026 Jan 26. (PMC12834121; doi:10.1016/j.ijheh.2022.114044)
Supplement: SupplementaryMaterial [file NIHMS2128159-supplement-SupplementaryMaterial.docx]

**Supplementary file: All full-text inclusions for Water-GEE systematic review**

Aakre, I., Bjøro, T., Norheim, I., Strand, T.A., Barikmo, I., Henjum, S., 2015. Excessive iodine intake and thyroid dysfunction among lactating Saharawi women. J Trace Elem Med Biol 31, 279–284. <https://doi.org/10.1016/j.jtemb.2014.09.009>

Aakre, I., Henjum, S., Folven Gjengedal, E.L., Risa Haugstad, C., Vollset, M., Moubarak, K., Saleh Ahmed, T., Alexander, J., Kjellevold, M., Molin, M., 2018. Trace Element Concentrations in Drinking Water and Urine among Saharawi Women and Young Children. Toxics 6, 40. <https://doi.org/10.3390/toxics6030040>

Aballay, L.R., Díaz, M. del P., Francisca, F.M., Muñoz, S.E., 2012. Cancer incidence and pattern of arsenic concentration in drinking water wells in Córdoba, Argentina. International Journal of Environmental Health Research 22, 220–231. <https://doi.org/10.1080/09603123.2011.628792>

Abbas, M., Cheema, K.J., 2015. Arsenic levels in drinking water and associated health risk in District Sheikhupura, Pakistan. Journal of Animal and Plant Sciences 25, 719–724.

Abdi, J., Farhadi, M., Aghaee, S., 2014. Prevalence of Intestinal Parasites among Children Attending the Daycare Centers of Ilam, Western Iran. Journal of Medical Sciences (Faisalabad) 14, 143–146. <https://doi.org/10.3923/jms.2014.143.146>

Abdulkareem, B.O., Habeeb, K.O., Kazeem, A., Adam, A.O., Samuel, U.U., 2018. Urogenital Schistosomiasis among Schoolchildren and the Associated Risk Factors in Selected Rural Communities of Kwara State, Nigeria. Journal of Tropical Medicine 2018, 6913918. <https://doi.org/10.1155/2018/6913918>

Abu Mourad, T.A., 2004. Palestinian refugee conditions associated with intestinal parasites and diarrhoea: Nuseirat refugee camp as a case study. Public Health 118, 131–142. <https://doi.org/10.1016/j.puhe.2003.09.002>

Abuaku, B., Zhou, J., Li, Xinhua, Li, S., Li, Xingli, Liu, A., Yang, T., Tan, H., 2009. Morbidity and mortality among populations suffering floods in Hunan, China: the role of socioeconomic status. <https://doi.org/10.1111/J.1753-318X.2009.01037.X>

Acheampong, M., Ejiofor, C., Salinas-Miranda, A., 2017. An Analysis of Determinants of Under-5 Mortality across Countries: Defining Priorities to Achieve Targets in Sustainable Developmental Goals. Matern Child Health J 21, 1428–1447. <https://doi.org/10.1007/s10995-017-2260-9>

Acquah, E., Darteh, E.K.M., Amu, H., Adjei, D.K.A., 2019. Predictors of underweight in children under-five years in Ghana. Ghana Med J 53, 71–78. <https://doi.org/10.4314/gmj.v53i1.11>

Adams, E.A., Juran, L., Ajibade, I., 2018. ‘Spaces of Exclusion’ in community water governance: A Feminist Political Ecology of gender and participation in Malawi’s Urban Water User Associations. Geoforum 95, 133–142. <https://doi.org/10.1016/j.geoforum.2018.06.016>

Adams, S.V., Barrick, B., Freney, E.P., Shafer, M.M., Song, X., Vilchis, H., Newcomb, P.A., Ulery, A., 2016. Urinary heavy metals in Hispanics 40–85 years old in Doña Ana County, New Mexico. Arch Environ Occup Health 71, 338–346. <https://doi.org/10.1080/19338244.2015.1129301>

Adhikari, H., Ghimire, T., 2009. Prevalence of Arsenicosis in Ramgram Municipality, Nawalparasi, Nepal. International Journal of Health Research 2, 183–188.

Adimalla, N., 2020. Controlling factors and mechanism of groundwater quality variation in semiarid region of South India: an approach of water quality index (WQI) and health risk assessment (HRA). Environ Geochem Health 42, 1725–1752. <https://doi.org/10.1007/s10653-019-00374-8>

Adimalla, N., 2019. Groundwater Quality for Drinking and Irrigation Purposes and Potential Health Risks Assessment: A Case Study from Semi-Arid Region of South India. Expo Health 11, 109–123. <https://doi.org/10.1007/s12403-018-0288-8>

Adimalla, N., Li, P., Qian, H., 2019. Evaluation of groundwater contamination for fluoride and nitrate in semi-arid region of Nirmal Province, South India: A special emphasis on human health risk assessment (HHRA). Human and Ecological Risk Assessment: An International Journal 25, 1107–1124. <https://doi.org/10.1080/10807039.2018.1460579>

Adoka, S.O., Anyona, D.N., Abuom, P.O., Dida, G.O., Karanja, D., Vulule, J.M., Okurut, T., Matano, A.S., Gichere, S.K., Ofulla, A.V.O., 2014. Community perceptions of Schistosomiasis transmission, prevalence, and control in relation to aquatic habitats in the Lake Victoria basin of Kenya. East Afr Med J 91, 232–244.

Adriko, M., Tinkitina, B., Arinaitwe, M., Kabatereine, N.B., Nanyunja, M., M Tukahebwa, E., 2018. Impact of a national deworming campaign on the prevalence of soil-transmitted helminthiasis in Uganda (2004-2016): Implications for national control programs. PLoS Negl Trop Dis 12, e0006520. <https://doi.org/10.1371/journal.pntd.0006520>

Agbadi, P., Darkwah, E., Kenney, P.L., 2019. A Multilevel Analysis of Regressors of Access to Improved Drinking Water and Sanitation Facilities in Ghana. Journal of Environmental and Public Health 2019, e3983869. <https://doi.org/10.1155/2019/3983869>

Agbor, N.E., Esemu, S.N., Ndip, L.M., Tanih, N.F., Smith, S.I., Ndip, R.N., 2018. Helicobacter pylori in patients with gastritis in West Cameroon: prevalence and risk factors for infection. BMC Research Notes 11, 559. <https://doi.org/10.1186/s13104-018-3662-5>

Aghaei, M., Derakhshani, R., Raoof, M., Dehghani, M., Mahvi, A., 2015a. Effect of fluoride in drinking water on birth height and weight: An ecological study in Kerman Province, Zarand county, Iran. Fluoride 48, 160–168.

Aghaei, M., Karimzadeh, S., Yaseri, M., Khorsandi, H., Zolfi, E., Mahvi, A., 2015b. Hypertension and fluoride in drinking water: Case study from West Azerbaijan, Iran. Fluoride 48, 252–258.

Aghapour, S., Bina, B., Tarrahi, M.J., Amiri, F., Ebrahimi, A., 2018. Distribution and health risk assessment of natural fluoride of drinking groundwater resources of Isfahan, Iran, using GIS. Environ Monit Assess 190, 137. <https://doi.org/10.1007/s10661-018-6467-z>

Aguemon, B.D., Struelens, M.J., Massougbodji, A., Ouendo, E.M., 2005. Prevalence and risk-factors for Helicobacter pylori infection in urban and rural Beninese populations. Clin Microbiol Infect 11, 611–617. <https://doi.org/10.1111/j.1469-0691.2005.01189.x>

Ahamed, S., Sengupta, M.K., Mukherjee, S.C., Pati, S., Mukherjeel, A., Rahman, Mohammad Mahmudur, Hossain, M.A., Das, B., Nayakl, B., Pal, A., Zafar, A., Kabir, S., Banu, S.A., Morshed, S., Islam, T., Rahman, M. Mahmuder, Quamruzzaman, Q., Chakraborti, D., 2006. An eight-year study report on arsenic contamination in groundwater and health effects in Eruani village, Bangladesh and an approach for its mitigation. J Health Popul Nutr 24, 129–141.

Ahenkorah, B., Nsiah, K., Baffoe, P., 2016. Sociodemographic and Obstetric Characteristics of Anaemic Pregnant Women Attending Antenatal Clinic in Bolgatanga Regional Hospital. Scientifica (Cairo) 2016, 4687342. <https://doi.org/10.1155/2016/4687342>

Ahmad, S.A., Sayed, M.H., Barua, S., Khan, M.H., Faruquee, M.H., Jalil, A., Hadi, S.A., Talukder, H.K., 2001. Arsenic in drinking water and pregnancy outcomes. Environ Health Perspect 109, 629–631.

Ahmad, S.A., Sayed, M.H., Faruquee, M.H., Khan, M.H., Jalil, M.A., Ahmed, R., Razzaque, M.A., Moyeen-us-safa, null, 1999. Arsenicosis: sex differentials. J Prev Soc Med 18, 35–40.

Ahmad, S.A., Sayed, M.H.S., Khan, M.H., Karim, M.N., Haque, M.A., Bhuiyan, M.S.A., Rahman, M.S., Faruquee, M.H., 2007. Sociocultural aspects of arsenicosis in Bangladesh: community perspective. J Environ Sci Health A Tox Hazard Subst Environ Eng 42, 1945–1958. <https://doi.org/10.1080/10934520701567247>

Ahmed, F., Khan, M.R., Shaheen, N., Ahmed, K.M.U., Hasan, A., Chowdhury, I.A., Chowdhury, R., 2018. Anemia and iron deficiency in rural Bangladeshi pregnant women living in areas of high and low iron in groundwater. Nutrition 51–52, 46–52. <https://doi.org/10.1016/j.nut.2018.01.014>

Ahmed, M., Billoo, A.G., Murtaza, G., 1995. Risk factors of persistent diarrhoea in children below five years of age. J Pak Med Assoc 45, 290–292.

Ahmed, S., Rekha, R.S., Ahsan, K.B., Doi, M., Grandér, M., Roy, A.K., Ekström, E.-C., Wagatsuma, Y., Vahter, M., Raqib, R., 2013. Arsenic Exposure Affects Plasma Insulin-Like Growth Factor 1 (IGF-1) in Children in Rural Bangladesh. PLoS ONE 8, e81530. <https://doi.org/10.1371/journal.pone.0081530>

Ahsan, H., Chen, Y., Parvez, F., Argos, M., Hussain, A.I., Momotaj, H., Levy, D., van Geen, A., Howe, G., Graziano, J., 2006a. Health Effects of Arsenic Longitudinal Study (HEALS): Description of a multidisciplinary epidemiologic investigation. J Expo Sci Environ Epidemiol 16, 191–205. <https://doi.org/10.1038/sj.jea.7500449>

Ahsan, H., Chen, Y., Parvez, F., Zablotska, L., Argos, M., Hussain, I., Momotaj, H., Levy, D., Cheng, Z., Slavkovich, V., van Geen, A., Howe, G.R., Graziano, J.H., 2006b. Arsenic exposure from drinking water and risk of premalignant skin lesions in Bangladesh: baseline results from the Health Effects of Arsenic Longitudinal Study. Am J Epidemiol 163, 1138–1148. <https://doi.org/10.1093/aje/kwj154>

Akter, S., 2019. Impact of drinking water salinity on children’s education: Empirical evidence from coastal Bangladesh. Science of The Total Environment 690, 1331–1341. <https://doi.org/10.1016/j.scitotenv.2019.06.458>

Akter, T., Jhohura, F.T., Akter, F., Chowdhury, T.R., Mistry, S.K., Dey, D., Barua, M.K., Islam, M.A., Rahman, M., 2016. Water Quality Index for measuring drinking water quality in rural Bangladesh: a cross-sectional study. Journal of Health, Population and Nutrition 35, 4. <https://doi.org/10.1186/s41043-016-0041-5>

al-Saleh, I., Shinwari, N., 1996. Aluminum in Saudi children. Biometals 9, 385–392. <https://doi.org/10.1007/BF00140608>

Alam, Dr.Md.A., Hakim, M., Rouf, M.A., Haque, M., Ali, M.E., Sarker, M.Z., 2011. Nutritional status of urban slum children below five years: Assessment by anthropometric measurements with special reference to socioeconomic status. Journal of Food, Agriculture and Environment 9, 85–90.

Alam, N., Wojtyniak, B., Henry, F.J., Rahaman, M.M., 1989. Mothers’ personal and domestic hygiene and diarrhoea incidence in young children in rural Bangladesh. Int J Epidemiol 18, 242–247. <https://doi.org/10.1093/ije/18.1.242>

Alaofè, H., Zee, J., Dossa, R., O’Brien, H.T., 2008. Intestinal parasitic infections in adolescent girls from two boarding schools in southern Benin. Trans R Soc Trop Med Hyg 102, 653–661. <https://doi.org/10.1016/j.trstmh.2008.02.013>

Al-Areeqi, M.A., Sady, H., Al-Mekhlafi, H.M., Anuar, T.S., Al-Adhroey, A.H., Atroosh, W.M., Dawaki, S., Elyana, F.N., Nasr, N.A., Ithoi, I., Lau, Y.-L., Surin, J., 2017. First molecular epidemiology of Entamoeba histolytica, E. dispar and E. moshkovskii infections in Yemen: different species-specific associated risk factors. Trop Med Int Health 22, 493–504. <https://doi.org/10.1111/tmi.12848>

Albouy-Llaty, M., Dupuis, A., Grignon, C., Strezlec, S., Pierre, F., Rabouan, S., Migeot, V., 2015. Estimating drinking-water ingestion and dermal contact with water in a French population of pregnant women: the EDDS cohort study. J Expo Sci Environ Epidemiol 25, 308–316. <https://doi.org/10.1038/jes.2014.48>

Albouy-Llaty, M., Limousi, F., Carles, C., Dupuis, A., Rabouan, S., Migeot, V., 2016. Association between Exposure to Endocrine Disruptors in Drinking Water and Preterm Birth, Taking Neighborhood Deprivation into Account: A Historic Cohort Study. Int J Environ Res Public Health 13, E796. <https://doi.org/10.3390/ijerph13080796>

Alfredo, K., Lawler, D., Katz, L., 2014. Fluoride contamination in the Bongo District of Ghana, West Africa: Geogenic contamination and cultural complexities. Water International 39. <https://doi.org/10.1080/02508060.2014.926234>

Alhassan, H., Kwakwa, P.A., 2013. When water is scarce: the perception of water quality and effects on the vulnerable. Journal of Water, Sanitation and Hygiene for Development 4, 43–50. <https://doi.org/10.2166/washdev.2013.140>

Alhava, E.M., Olkkonen, H., Kauranen, P., Kari, T., 1980. The effect of drinking water fluoridation on the fluoride content, strength and mineral density of human bone. Acta Orthop Scand 51, 413–420. <https://doi.org/10.3109/17453678008990817>

Ali, F., Kazi, T.G., Afridi, H.I., Baig, J.A., 2018. Exposure of cadmium via smoking and drinking water on zinc levels of biological samples of malnutrition pregnant women: A prospective cohort study. Environ Toxicol Pharmacol 63, 48–54. <https://doi.org/10.1016/j.etap.2018.08.013>

Ali, H.I., Al Dhaheri, A.S., Elmi, F., Ng, S.W., Zaghloul, S., Ohuma, E.O., Qazaq, H.S., 2019. Water and Beverage Consumption among a Nationally Representative Sample of Children and Adolescents in the United Arab Emirates. Nutrients 11, 2110. <https://doi.org/10.3390/nu11092110>

Ali, M., Ghenghesh, K., Ridha, B.A., Abuhelfaia, A., Dufani, M., 2005. Etiology of childhood diarrhea in Zliten-Libya. Saudi medical journal 26, 1759–65.

Aliyu, A.A., Oguntunde, O.O., Dahiru, T., Raji, T., 2012. Prevalence and Determinants of Malnutrition among Pre-School Children in Northern Nigeria. Pakistan J. of Nutrition 11, 1092–1095. <https://doi.org/10.3923/pjn.2012.1092.1095>

Alman, B.L., Coffman, E., Siega-Riz, A.M., Luben, T.J., National Birth Defects Prevention Study, 2017. Associations between Maternal Water Consumption and Birth Defects in the National Birth Defects Prevention Study (2000-2005). Birth Defects Res 109, 193–202. <https://doi.org/10.1002/bdra.23569>

Al-Mazrou, Y., Khan, U., Aziz, K., 1997. Determinants of under-five mortality in Saudi Arabia. Saudi Medical Journal 18, 31–36.

Almeida, L.M., Werneck, G.L., Cairncross, S., Coeli, C.M., Costa, M.C., Coletty, P.E., 2001. The epidemiology of hepatitis A in Rio de Janeiro: environmental and domestic risk factors. Epidemiol Infect 127, 327–333.

Al-Mekhlafi, H.M., 2017. Giardia duodenalis infection among rural communities in Yemen: A community-based assessment of the prevalence and associated risk factors. Asian Pac J Trop Med 10, 987–995. <https://doi.org/10.1016/j.apjtm.2017.09.011>

Al-Mohammad, H.I., Amin, T.T., Balaha, M.H., Al-Moghannum, M.S., 2010. Toxoplasmosis among the pregnant women attending a Saudi maternity hospital: seroprevalence and possible risk factors. Ann Trop Med Parasitol 104, 493–504. <https://doi.org/10.1179/136485910X12786389891443>

Al-Naama, L., Hassan, M., Hehdi, J.K., Sadoon, I.O., 2010. Screening for Blood Lead Levels in Basrah, Southern Iraq. Qatar Medical Journal 19, 43–47. <https://doi.org/10.5339/qmj.2010.2.15>

Al-Shamiri, A., Al-Zubairy, A., Al-Mamari, R., 2010. The Prevalence of Cryptosporidium spp. in Children, Taiz District, Yemen. Iran J Parasitol 5, 26–32.

Al-Sheyab, N.A., Obaidat, M.M., Bani Salman, A.E., Lafi, S.Q., 2015. Toxoplasmosis-Related Knowledge and Preventive Practices among Undergraduate Female Students in Jordan. J Food Prot 78, 1161–1166. <https://doi.org/10.4315/0362-028X.JFP-14-579>

Alyousefi, N.A., Mahdy, M.A.K., Mahmud, R., Lim, Y.A.L., 2011. Factors associated with high prevalence of intestinal protozoan infections among patients in Sana’a City, Yemen. PLoS One 6, e22044. <https://doi.org/10.1371/journal.pone.0022044>

Amano, I., Kurisu, K., Hanaki, K., 2016. Evaluation of consumers’ preferences on drinking waters considering the information provision effect. Water Supply 16, 1057–1067. <https://doi.org/10.2166/ws.2016.019>

Amanya, G., Kizito, S., Nabukenya, I., Kalyango, J., Atuheire, C., Nansumba, H., Abwoye, S.A., Opio, D.N., Kibuuka, E., Karamagi, C., 2017. Risk factors, person, place and time characteristics associated with Hepatitis E Virus outbreak in Napak District, Uganda. BMC Infect Dis 17, 451. <https://doi.org/10.1186/s12879-017-2542-2>

Ameer, S.S., Engström, K., Harari, F., Concha, G., Vahter, M., Broberg, K., 2015. The effects of arsenic exposure on blood pressure and early risk markers of cardiovascular disease: Evidence for population differences. Environ Res 140, 32–36. <https://doi.org/10.1016/j.envres.2015.03.010>

Ameer, S.S., Engström, K., Hossain, M.B., Concha, G., Vahter, M., Broberg, K., 2017. Arsenic exposure from drinking water is associated with decreased gene expression and increased DNA methylation in peripheral blood. Toxicol Appl Pharmacol 321, 57–66. <https://doi.org/10.1016/j.taap.2017.02.019>

Ameer, S.S., Xu, Y., Engström, K., Li, H., Tallving, P., Nermell, B., Boemo, A., Parada, L.A., Peñaloza, L.G., Concha, G., Harari, F., Vahter, M., Broberg, K., 2016. Exposure to Inorganic Arsenic Is Associated with Increased Mitochondrial DNA Copy Number and Longer Telomere Length in Peripheral Blood. Front Cell Dev Biol 4, 87. <https://doi.org/10.3389/fcell.2016.00087>

Amegah, A.K., Näyhä, S., Jaakkola, J., 2017. Do biomass fuel use and consumption of unsafe water mediate educational inequalities in stillbirth risk? An analysis of the 2007 Ghana Maternal Health Survey. BMJ Open 7, e012348. <https://doi.org/10.1136/bmjopen-2016-012348>

Amini, H., Taghavi Shahri, S.M., Amini, M., Ramezani Mehrian, M., Mokhayeri, Y., Yunesian, M., 2011. Drinking water fluoride and blood pressure? An environmental study. Biol Trace Elem Res 144, 157–163. <https://doi.org/10.1007/s12011-011-9054-5>

Amster, E.D., Cho, J.I., Christiani, D., 2011. Urine arsenic concentration and obstructive pulmonary disease in the U.S. population. J Toxicol Environ Health A 74, 716–727. <https://doi.org/10.1080/15287394.2011.556060>

An, R., McCaffrey, J., 2016. Plain water consumption in relation to energy intake and diet quality among US adults, 2005-2012. J Hum Nutr Diet 29, 624–632. <https://doi.org/10.1111/jhn.12368>

Anand, P.K., Ramakrishnan, R., 2010. Investigation of the outbreak of typhoid in a village of Thar Desert Rajasthan, India. Indian J Med Res 131, 799–803.

Anatol, T., Pinto Pereira, L., Simeon, D., Sawh, L., 2003. Risk factors for urinary tract calculi in Trinidad. Trop Med Int Health 8, 348–353. <https://doi.org/10.1046/j.1365-3156.2003.01009.x>

Andajani-Sutjahjo, S., Chirawatkul, S., Saito, E., 2015. Gender and Water in Northeast Thailand: Inequalities and Women’s Realities. Journal of International Women’s Studies 16, 200–212.

Andersen, S., Iversen, F., Terpling, S., Pedersen, K.M., Gustenhoff, P., Laurberg, P., 2012. Iodine deficiency influences thyroid autoimmunity in old age--a comparative population-based study. Maturitas 71, 39–43. <https://doi.org/10.1016/j.maturitas.2011.10.001>

Andersson, E.M., Scott, K., Xu, Y., Li, Y., Olsson, D.S., Fletcher, T., Jakobsson, K., 2019. High exposure to perfluorinated compounds in drinking water and thyroid disease. A cohort study from Ronneby, Sweden. Environmental Research 176, 108540. <https://doi.org/10.1016/j.envres.2019.108540>

Andiappan, H., Nissapatorn, V., Sawangjaroen, N., Chemoh, W., Lau, Y.L., Kumar, T., Onichandran, S., Suwanrath, C., Chandeying, V., 2014. Toxoplasma infection in pregnant women: a current status in Songklanagarind hospital, southern Thailand. Parasites & Vectors 7, 239. <https://doi.org/10.1186/1756-3305-7-239>

Andrianou, X.D., Charisiadis, P., Andra, S.S., Makris, K.C., 2014. Spatial and seasonal variability of urinary trihalomethanes concentrations in urban settings. Environ Res 135, 289–295. <https://doi.org/10.1016/j.envres.2014.09.015>

Anh, N.T.N., Nishijo, M., Tai, P.T., Maruzeni, S., Morikawa, Y., Anh, T.H., Van Luong, H., Dam, P.M., Nakagawa, H., Son, L.K., Nishijo, H., 2014. Maternal risk factors associated with increased dioxin concentrations in breast milk in a hot spot of dioxin contamination in Vietnam. J Expo Sci Environ Epidemiol 24, 489–496. <https://doi.org/10.1038/jes.2013.73>

Ankarklev, J., Hestvik, E., Lebbad, M., Lindh, J., Kaddu-Mulindwa, D.H., Andersson, J.O., Tylleskär, T., Tumwine, J.K., Svärd, S.G., 2012. Common coinfections of Giardia intestinalis and Helicobacter pylori in non-symptomatic Ugandan children. PLoS Negl Trop Dis 6, e1780. <https://doi.org/10.1371/journal.pntd.0001780>

Aptel, I., Cance-Rouzaud, A., Grandjean, H., 1999. Association between calcium ingested from drinking water and femoral bone density in elderly women: evidence from the EPIDOS cohort. J Bone Miner Res 14, 829–833. <https://doi.org/10.1359/jbmr.1999.14.5.829>

Aramayo, C.F., Gil, J.F., Cruz, M.C., Poma, H.R., Last, M.S., Rajal, V.B., 2009. Diarrhea and parasitosis in Salta, Argentina. J Infect Dev Ctries 3, 105–111. <https://doi.org/10.3855/jidc.57>

Araya, M., Olivares, M., Pizarro, F., Llanos, A., Figueroa, G., Uauy, R., 2004. Community-Based Randomized Double-Blind Study of Gastrointestinal Effects and Copper Exposure in Drinking Water. Environ Health Perspect 112, 1068–1073. <https://doi.org/10.1289/ehp.6913>

Archer, J., Hudson-Edwards, K.A., Preston, D.A., Howarth, R.J., Linge, K., 2005. Aqueous exposure and uptake of arsenic by riverside communities affected by mining contamination in the Río Pilcomayo basin, Bolivia. Mineralogical Magazine 69, 719–736. <https://doi.org/10.1180/0026461056950283>

Archer, N.P., Napier, T.S., Villanacci, J.F., 2016. Fluoride exposure in public drinking water and childhood and adolescent osteosarcoma in Texas. Cancer Causes Control 27, 863–868. <https://doi.org/10.1007/s10552-016-0759-9>

Argos, M., Kalra, T., Pierce, B.L., Chen, Y., Parvez, F., Islam, T., Ahmed, A., Hasan, R., Hasan, K., Sarwar, G., Levy, D., Slavkovich, V., Graziano, J.H., Rathouz, P.J., Ahsan, H., 2011. A Prospective Study of Arsenic Exposure From Drinking Water and Incidence of Skin Lesions in Bangladesh. Am J Epidemiol 174, 185–194. <https://doi.org/10.1093/aje/kwr062>

Argos, M., Kibriya, M.G., Parvez, F., Jasmine, F., Rakibuz-Zaman, M., Ahsan, H., 2006. Gene expression profiles in peripheral lymphocytes by arsenic exposure and skin lesion status in a Bangladeshi population. Cancer Epidemiol Biomarkers Prev 15, 1367–1375. <https://doi.org/10.1158/1055-9965.EPI-06-0106>

Argos, M., Parvez, F., Chen, Y., Hussain, A.Z.M.I., Momotaj, H., Howe, G.R., Graziano, J.H., Ahsan, H., 2007. Socioeconomic Status and Risk for Arsenic-Related Skin Lesions in Bangladesh. Am J Public Health 97, 825–831. <https://doi.org/10.2105/AJPH.2005.078816>

Argos, M., Parvez, F., Rahman, M., Rakibuz-Zaman, M., Ahmed, A., Hore, S.K., Islam, T., Chen, Y., Pierce, B.L., Slavkovich, V., Olopade, C., Yunus, M., Baron, J.A., Graziano, J.H., Ahsan, H., 2014. Arsenic and lung disease mortality in Bangladeshi adults. Epidemiology 25, 536–543. <https://doi.org/10.1097/EDE.0000000000000106>

Argos, M., Rahman, M., Parvez, F., Dignam, J., Islam, T., Quasem, I., K Hore, S., T Haider, A., Hossain, Z., I Patwary, T., Rakibuz-Zaman, M., Sarwar, G., La Porte, P., Harjes, J., Anton, K., Kibriya, M.G., Jasmine, F., Khan, R., Kamal, M., Shea, C.R., Yunus, M., Baron, J.A., Ahsan, H., 2013. Baseline comorbidities in a skin cancer prevention trial in Bangladesh. Eur J Clin Invest 43, 579–588. <https://doi.org/10.1111/eci.12085>

Arıkan, İ., Gülcan, A., Dıbeklıoğlu, S.E., 2016. Investigation of Factors Affecting Frequency of Intestinal Parasites in Primary School Students in an Urban Region in Turkey. Cent Eur J Public Health 24, 193–198. <https://doi.org/10.21101/cejph.a4231>

Armstrong, B.K., Margetts, B.M., Binns, C.W., Campbell, N.A., Masarei, J.R., McCall, M.G., 1982. Water sodium and blood pressure in rural school children. Arch Environ Health 37, 236–245. <https://doi.org/10.1080/00039896.1982.10667571>

Arnold, C.M., Bailey, D.A., Faulkner, R.A., McKay, H.A., McCulloch, R.G., 1997. The Effect of Water Fluoridation on the Bone Mineral Density of Young Women. Can J Public Health 88, 388–391. <https://doi.org/10.1007/BF03403912>

Arya, S., Subramani, T., Vennila, G., Karunanidhi, D., 2021. Health risks associated with fluoride intake from rural drinking water supply and inverse mass balance modeling to decipher hydrogeochemical processes in Vattamalaikarai River basin, South India. Environ Geochem Health 43, 705–716. <https://doi.org/10.1007/s10653-019-00489-y>

Aryaeipour, M., Kia, E.B., Heidari, Z., Sayyad Talaie, Z., Rokni, M.B., 2015. Serological study of Human Fasciolosis in Patients Referring to the School of Public Health, Tehran University of Medical Sciences, Tehran, Iran during 2008–2014. Iran J Parasitol 10, 517–522.

Aschebrook-Kilfoy, B., Heltshe, S.L., Nuckols, J.R., Sabra, M.M., Shuldiner, A.R., Mitchell, B.D., Airola, M., Holford, T.R., Zhang, Y., Ward, M.H., 2012. Modeled nitrate levels in well water supplies and prevalence of abnormal thyroid conditions among the Old Order Amish in Pennsylvania. Environ Health 11, 6. <https://doi.org/10.1186/1476-069X-11-6>

Aschengrau, A., Gallagher, L.G., Winter, M., Butler, L., Patricia Fabian, M., Vieira, V.M., 2018. Modeled exposure to tetrachloroethylene-contaminated drinking water and the occurrence of birth defects: a case-control study from Massachusetts and Rhode Island. Environ Health 17, 75. <https://doi.org/10.1186/s12940-018-0419-5>

Aschengrau, A., Paulu, C., Ozonoff, D., 1998. Tetrachloroethylene-contaminated drinking water and the risk of breast cancer. Environmental Health Perspectives 106, 947–953. <https://doi.org/10.1289/ehp.98106s4947>

Aschengrau, A., Rogers, S., Ozonoff, D., 2003. Perchloroethylene-contaminated drinking water and the risk of breast cancer: additional results from Cape Cod, Massachusetts, USA. Environ Health Perspect 111, 167–173. <https://doi.org/10.1289/ehp.4980>

Aschengrau, A., Weinberg, J.M., Gallagher, L.G., Winter, M.R., Vieira, V.M., Webster, T.F., Ozonoff, D.M., 2009a. Exposure to Tetrachloroethylene-Contaminated Drinking Water and the Risk of Pregnancy Loss. Water Qual Expo Health 1, 23–34. <https://doi.org/10.1007/s12403-009-0003-x>

Aschengrau, A., Weinberg, J.M., Janulewicz, P.A., Gallagher, L.G., Winter, M.R., Vieira, V.M., Webster, T.F., Ozonoff, D.M., 2009b. Prenatal exposure to tetrachloroethylene-contaminated drinking water and the risk of congenital anomalies: a retrospective cohort study. Environ Health 8, 44. <https://doi.org/10.1186/1476-069X-8-44>

Aschengrau, A., Zierler, S., Cohen, A., 1993. Quality of Community Drinking Water and the Occurrence of Late Adverse Pregnancy Outcomes. Archives of Environmental Health: An International Journal 48, 105–113. <https://doi.org/10.1080/00039896.1993.9938403>

Aschengrau, A., Zierler, S., Cohen, A., 1989. Quality of community drinking water and the occurrence of spontaneous abortion. Arch Environ Health 44, 283–290. <https://doi.org/10.1080/00039896.1989.9935895>

Assiri, A., Hassan, G., Marzouk, N., Ahmed, A., Al-Eyadhy, A., Mohamed, E., 2015. Characteristics of Hepatitis E Virus Infection in Children Attending a Tertiary Care Teaching Hospital in Upper Egypt: 2111. American Journal of Gastroenterology 110, S883. <https://doi.org/10.14309/00000434-201510001-02111>

Athanasatou, A., Kandyliari, A., Malisova, O., Kapsokefalou, M., 2019. Fluctuation of Water Intake and of Hydration Indices during the Day in a Sample of Healthy Greek Adults. Nutrients 11, 793. <https://doi.org/10.3390/nu11040793>

Atuyambe, L.M., Ediau, M., Orach, C.G., Musenero, M., Bazeyo, W., 2011. Land slide disaster in eastern Uganda: rapid assessment of water, sanitation and hygiene situation in Bulucheke camp, Bududa district. Environ Health 10, 38. <https://doi.org/10.1186/1476-069X-10-38>

Awuku, Y.A., Simpong, D.L., Alhassan, I.K., Tuoyire, D.A., Afaa, T., Adu, P., 2017. Prevalence of helicobacter pylori infection among children living in a rural setting in Sub-Saharan Africa. BMC Public Health 17, 360. <https://doi.org/10.1186/s12889-017-4274-z>

Ayazi, T., Swartz, L., Eide, A.H., Lien, L., Hauff, E., 2015. Perceived current needs, psychological distress and functional impairment in a war-affected setting: a cross-sectional study in South Sudan. BMJ Open 5, e007534. <https://doi.org/10.1136/bmjopen-2014-007534>

Ayotte, J.D., Baris, D., Cantor, K.P., Colt, J., Robinson, G.R., Lubin, J.H., Karagas, M., Hoover, R.N., Fraumeni, J.F., Silverman, D.T., 2006. Bladder cancer mortality and private well use in New England: an ecological study. J Epidemiol Community Health 60, 168–172. <https://doi.org/10.1136/jech.2005.038620>

Aziz, S.N., Boyle, K.J., Rahman, M., 2006. Knowledge of arsenic in drinking-water: risks and avoidance in Matlab, Bangladesh. J Health Popul Nutr 24, 327–335.

Bachir, K., Salah, B., 2016. A study of epidemic of typhoid fever in the Souf oasis (Eastern South of Algeria). Research Journal of Pharmaceutical, Biological and Chemical Sciences 7, 1299–1307.

Baguma, D., Hashim, J.H., Aljunid, S.M., Loiskandl, W., 2013. Safe-water shortages, gender perspectives, and related challenges in developing countries: the case of Uganda. Sci Total Environ 442, 96–102. <https://doi.org/10.1016/j.scitotenv.2012.10.004>

Baig, J.A., Kazi, T.G., Mustafa, M.A., Solangi, I.B., Mughal, M.J., Afridi, H.I., 2016. Arsenic Exposure in Children through Drinking Water in Different Districts of Sindh, Pakistan. Biol Trace Elem Res 173, 35–46. <https://doi.org/10.1007/s12011-016-0636-0>

Baingana, R.K., Kiboko Enyaru, J., Davidsson, L., 2014. Helicobacter pylori infection in pregnant women in four districts of Uganda: role of geographic location, education and water sources. BMC Public Health 14, 915. <https://doi.org/10.1186/1471-2458-14-915>

Baker, K.K., Story, W.T., Walser-Kuntz, E., Zimmerman, M.B., 2018. Impact of social capital, harassment of women and girls, and water and sanitation access on premature birth and low infant birth weight in India. PLoS ONE 13, e0205345. <https://doi.org/10.1371/journal.pone.0205345>

Balaghi, S., Faramarzi, E., Mahdavi, R., Ghaemmaghami, J., 2011. Fluids Intake and Beverage Consumption Pattern among University Students. Health Promot Perspect 1, 54–61. <https://doi.org/10.5681/hpp.2011.005>

Banu, S.A., Kile, M.L., Christiani, D.C., Qumruzzaman, Q., 2013. Study of Prenatal Arsenic Exposure and Reproductive Health Outcome in Bangladesh. Bangladesh Journal of Obstetrics & Gynaecology 28, 76–81. <https://doi.org/10.3329/bjog.v28i2.30094>

Barakat, A., Mouhtarim, G., Saji, R., Touhami, F., 2020. Health risk assessment of nitrates in the groundwater of Beni Amir irrigated perimeter, Tadla plain, Morocco. Human and Ecological Risk Assessment: An International Journal 26, 1864–1878. <https://doi.org/10.1080/10807039.2019.1613631>

Barati, A.H., Maleki, A., Alasvand, M., 2010. Multi-trace elements level in drinking water and the prevalence of multi-chronic arsenical poisoning in residents in the west area of Iran. Sci Total Environ 408, 1523–1529. <https://doi.org/10.1016/j.scitotenv.2009.12.035>

Barbone, F., Valent, F., Brussi, V., Tomasella, L., Triassi, M., Di Lieto, A., Scognamiglio, G., Righi, E., Fantuzzi, G., Casolari, L., Aggazzotti, G., 2002. Assessing the exposure of pregnant women to drinking water disinfection byproducts. Epidemiology 13, 540–544. <https://doi.org/10.1097/00001648-200209000-00009>

Bardosono, S., Morin, C., Guelinckx, I., Pohan, R., 2017. Pregnant and Breastfeeding Women: Drinking for Two? Ann Nutr Metab 70, 13–17. <https://doi.org/10.1159/000462998>

Basílio, I.L.D., Catão, M. de F.C., Carvalho, J.D. de S., Freire-Neto, F.P., Ferreira, L.C., Jerônimo, S.M.B., 2018. Risk factors of Helicobacter pylori infection in an urban community in Northeast Brazil and the relationship between the infection and gastric diseases. Rev. Soc. Bras. Med. Trop. 51, 183–189. <https://doi.org/10.1590/0037-8682-0412-2016>

Bassin, E.B., Wypij, D., Davis, R.B., Mittleman, M.A., 2006. Age-specific fluoride exposure in drinking water and osteosarcoma (United States). Cancer Causes Control 17, 421–428. <https://doi.org/10.1007/s10552-005-0500-6>

Bean, J.A., Isacson, P., Hahne, R.M., Kohler, J., 1982a. Drinking water and cancer incidence in Iowa. II. Radioactivity in drinking water. Am J Epidemiol 116, 924–932. <https://doi.org/10.1093/oxfordjournals.aje.a113494>

Bean, J.A., Isacson, P., Hausler, W.J., Kohler, J., 1982b. Drinking water and cancer incidence in Iowa. I. Trends and incidence by source of drinking water and size of municipality. Am J Epidemiol 116, 912–923. <https://doi.org/10.1093/oxfordjournals.aje.a113493>

Beer, K.D., Gargano, J.W., Roberts, V.A., Reses, H.E., Hill, V.R., Garrison, L.E., Kutty, P.K., Hilborn, E.D., Wade, T.J., Fullerton, K.E., Yoder, J.S., 2015. Outbreaks Associated With Environmental and Undetermined Water Exposures — United States, 2011–2012. MMWR Morb Mortal Wkly Rep 64, 849–851.

Beier, D., Brzoska, P., Khan, M.M.H., 2015. Indirect consequences of extreme weather and climate events and their associations with physical health in coastal Bangladesh: a cross-sectional study. Glob Health Action 8, 29016. <https://doi.org/10.3402/gha.v8.29016>

Benbassat, C., Tsvetov, G., Schindel, B., Hod, M., Blonder, Y., Sela, B.A., 2004. Assessment of iodine intake in the Israel coastal area. Isr Med Assoc J 6, 75–77.

Bennett, S.D., Otieno, R., Ayers, T.L., Odhiambo, A., Faith, S.H., Quick, R., 2015. Acceptability and use of portable drinking water and hand washing stations in health care facilities and their impact on patient hygiene practices, Western kenya. PLoS One 10, e0126916. <https://doi.org/10.1371/journal.pone.0126916>

Bentley, K., Soebandrio, A., 2017. Dietary exposure assessment for arsenic and mercury following submarine tailings placement in Ratatotok Sub-district, North Sulawesi, Indonesia. Environ Pollut 227, 552–559. <https://doi.org/10.1016/j.envpol.2017.04.081>

Beresford, S.A.A., 1983. Cancer Incidence and Reuse of Drinking Water. American Journal of Epidemiology 117, 258–268. <https://doi.org/10.1093/oxfordjournals.aje.a113538>

Berhe, K., Fseha, B., Gebremariam, G., Teame, H., Etsay, N., Welu, G., Tsegay, T., 2019. Risk factors of anemia among pregnant women attending antenatal care in health facilities of Eastern Zone of Tigray, Ethiopia, case-control study, 2017/18. Pan Afr Med J 34, 121. <https://doi.org/10.11604/pamj.2019.34.121.15999>

Bernardi, D., Dini, F.L., Azzarelli, A., Giaconi, A., Volterrani, C., Lunardi, M., 1995. Sudden cardiac death rate in an area characterized by high incidence of coronary artery disease and low hardness of drinking water. Angiology 46, 145–149. <https://doi.org/10.1177/000331979504600208>

Beyene, H., Deressa, W., Kumie, A., Grace, D., 2018. Determinants of diarrhoeal morbidity: The case of children under five years of age among agricultural and agro-pastoralist community of southern Ethiopia. Ethiopian Journal of Health Development 32. <https://doi.org/10.4314/ejhd.v32i1>

Bhagavan, S.V.B.K., Raghu, V., 2005. Utility of check dams in dilution of fluoride concentration in ground water and the resultant analysis of blood serum and urine of villagers, Anantapur District, Andhra Pradesh, India. Environ Geochem Health 27, 97–108. <https://doi.org/10.1007/s10653-004-0786-4>

Bhengra, M.P., Kumar, A., Kumar, Prabhat, Kumar, Pradeep, Chaudhary, S., 2019. A cross-sectional observational study on the effect of usage of chronic arsenic contamination of ground water among residents of Pathalkudwa Mohalla in Ranchi district as reported in a tertiary care centre in Jharkhand. undefined 28.

Bhunia, R., Ramakrishnan, R., Hutin, Y., Gupte, M.D., 2009. Cholera outbreak secondary to contaminated pipe water in an urban area, West Bengal, India, 2006. Indian J Gastroenterol 28, 62–64. <https://doi.org/10.1007/s12664-009-0020-5>

Bierkens, J., Smolders, R., Van Holderbeke, M., Cornelis, C., 2011. Predicting blood lead levels from current and past environmental data in Europe. Sci Total Environ 409, 5101–5110. <https://doi.org/10.1016/j.scitotenv.2011.08.034>

Bigwan, E., Kunihya, R.Z., John, T., 2013. Epidemiological survey of urinary Schistosomiasis among primary school children in Michika, Adamawa state, North-Eastern Nigeria. International journal of current research and review 5, 111–116.

Bile, K., Isse, A., Mohamud, O., Allebeck, P., Nilsson, L., Norder, H., Mushahwar, I.K., Magnius, L.O., 1994. Contrasting roles of rivers and wells as sources of drinking water on attack and fatality rates in a hepatitis E epidemic in Somalia. Am J Trop Med Hyg 51, 466–474.

Bile, M.K., Asghar, H., Burney, M.I., Sami, Z., Rab, M.A., Dil, A.S., Chaudhry, M.A., Mubarik, M.M., Siddiqi, S., Barzgar, M.A., 1997. Water-Borne Hepatitis E Virus Epidemic in Islamabad, Pakistan: A Common Source Outbreak Traced to the Malfunction of a Modern Water Treatment Plant. The American Journal of Tropical Medicine and Hygiene 57, 151–157. <https://doi.org/10.4269/ajtmh.1997.57.151>

Bisayher, S., Barennes, H., Nicand, E., Buisson, Y., 2019. Seroprevalence and risk factors of hepatitis E among women of childbearing age in the Xieng Khouang province (Lao People’s Democratic Republic), a cross-sectional survey. Trans R Soc Trop Med Hyg 113, 298–304. <https://doi.org/10.1093/trstmh/try143>

Bisung, E., Elliott, S.J., Abudho, B., Schuster-Wallace, C.J., Karanja, D.M., 2015. Dreaming of toilets: using photovoice to explore knowledge, attitudes and practices around water-health linkages in rural Kenya. Health Place 31, 208–215. <https://doi.org/10.1016/j.healthplace.2014.12.007>

Biswas, A., Deb, D., Ghose, A., Du Laing, G., De Neve, J., Santra, S.C., Guha Mazumder, D.N., 2014a. Dietary arsenic consumption and urine arsenic in an endemic population: response to improvement of drinking water quality in a 2-year consecutive study. Environ Sci Pollut Res Int 21, 609–619. <https://doi.org/10.1007/s11356-013-1947-8>

Biswas, A., Deb, D., Ghose, A., Santra, S.C., Guha Mazumder, D.N., 2014b. Seasonal perspective of dietary arsenic consumption and urine arsenic in an endemic population. Environ Monit Assess 186, 4543–4551. <https://doi.org/10.1007/s10661-014-3718-5>

Biswas, D.K., Bhunia, R., Maji, D., Das, P., 2014. Contaminated Pond Water Favors Cholera Outbreak at Haibatpur Village, Purba Medinipur District, West Bengal, India. Journal of Tropical Medicine 2014, e764530. <https://doi.org/10.1155/2014/764530>

Bivolarska, A., Gatseva, P., 2015. Thyroid status in pregnant women and association with nitrates as an environmental factor stimulating the manifestation of iodine deficiency. Trace Elements and Electrolytes 32, 60–64. <https://doi.org/10.5414/TEX01365>

Blaurock-Busch, E., Busch, Y.M., Friedle, A., Buerner, H., Parkash, C., Kaur, A., 2014. Comparing the Metal Concentration in the Hair of Cancer Patients and Healthy People Living in the Malwa Region of Punjab, India. Clin Med Insights Oncol 8, 1–13. <https://doi.org/10.4137/CMO.S13410>

Bloom, M.S., Neamtiu, I.A., Surdu, S., Pop, C., Anastasiu, D., Appleton, A.A., Fitzgerald, E.F., Gurzau, E.S., 2016. Low level arsenic contaminated water consumption and birth outcomes in Romania-An exploratory study. Reprod Toxicol 59, 8–16. <https://doi.org/10.1016/j.reprotox.2015.10.012>

Bloom, M.S., Neamtiu, I.A., Surdu, S., Pop, C., Lupsa, I.R., Anastasiu, D., Fitzgerald, E.F., Gurzau, E.S., 2014. Consumption of low-moderate level arsenic contaminated water does not increase spontaneous pregnancy loss: a case control study. Environ Health 13, 81. <https://doi.org/10.1186/1476-069X-13-81>

Bogart, L.M., Babey, S.H., Patel, A.I., Wang, P., Schuster, M.A., 2016. Lunchtime School Water Availability and Water Consumption Among California Adolescents. J Adolesc Health 58, 98–103. <https://doi.org/10.1016/j.jadohealth.2015.09.007>

Bonnet, F., Lepicard, E.M., Cathrin, L., Letellier, C., Constant, F., Hawili, N., Friedlander, G., 2012. French children start their school day with a hydration deficit. Ann Nutr Metab 60, 257–263. <https://doi.org/10.1159/000337939>

Boone, C., Glick, P., Sahn, D.E., 2011. Household Water Supply Choice and Time Allocated to Water Collection: Evidence from Madagascar. The Journal of Development Studies 47, 1826–1850. <https://doi.org/10.1080/00220388.2011.579394>

Borges, V.T.M., Rososchansky, J., Abbade, J.F., Dias, A., Peraçoli, J.C., Rudge, M.V.C., 2011. Effect of maternal hydration on the increase of amniotic fluid index. Braz J Med Biol Res 44, 263–266. <https://doi.org/10.1590/s0100-879x2011007500009>

Borjan, M., Marcella, S., Blount, B., Greenberg, M., Zhang, J.J., Murphy, E., Valentin-Blasini, L., Robson, M., 2011. Perchlorate exposure in lactating women in an urban community in New Jersey. Sci Total Environ 409, 460–464. <https://doi.org/10.1016/j.scitotenv.2010.10.045>

Bouchard, M., Laforest, F., Vandelac, L., Bellinger, D., Mergler, D., 2007. Hair manganese and hyperactive behaviors: pilot study of school-age children exposed through tap water. Environ Health Perspect 115, 122–127. <https://doi.org/10.1289/ehp.9504>

Bouchard, M.F., Surette, C., Cormier, P., Foucher, D., 2018. Low level exposure to manganese from drinking water and cognition in school-age children. Neurotoxicology 64, 110–117. <https://doi.org/10.1016/j.neuro.2017.07.024>

Bougatsas, D., Arnaoutis, G., Panagiotakos, D.B., Seal, A.D., Johnson, E.C., Bottin, J.H., Tsipouridi, S., Kavouras, S.A., 2018. Fluid consumption pattern and hydration among 8-14 years-old children. Eur J Clin Nutr 72, 420–427. <https://doi.org/10.1038/s41430-017-0012-y>

Bound, J.P., Harvey, P.W., Francis, B.J., Awwad, F., Gatrell, A.C., 1997. Involvement of deprivation and environmental lead in neural tube defects: a matched case-control study. Arch Dis Child 76, 107–112. <https://doi.org/10.1136/adc.76.2.107>

Bowie, W.R., King, A.S., Werker, D.H., Isaac-Renton, J.L., Bell, A., Eng, S.B., Marion, S.A., 1997. Outbreak of toxoplasmosis associated with municipal drinking water. The Lancet 350, 173–177. <https://doi.org/10.1016/S0140-6736(96)11105-3>

Boyce, J.M., Hughes, J.M., Alim, A.R., Khan, M., Aziz, K.M., Wells, J.G., Curlin, G.T., 1982. Patterns of Shigella infection in families in rural Bangladesh. Am J Trop Med Hyg 31, 1015–1020. <https://doi.org/10.4269/ajtmh.1982.31.1015>

Brahmbhatt, S., Brahmbhatt, R.M., Boyages, S.C., 2000. Thyroid ultrasound is the best prevalence indicator for assessment of iodine deficiency disorders: a study in rural/tribal schoolchildren from Gujarat (Western India). Eur J Endocrinol 143, 37–46. <https://doi.org/10.1530/eje.0.1430037>

Brahmbhatt, S.R., Fearnley, R., Brahmbhatt, R.M., Eastman, C.J., Boyages, S.C., 2001. Study of biochemical prevalence indicators for the assessment of iodine deficiency disorders in adults at field conditions in Gujarat (India). Asia Pac J Clin Nutr 10, 51–57. <https://doi.org/10.1046/j.1440-6047.2001.00197.x>

Brender, J.D., Olive, J.M., Felkner, M., Suarez, L., Marckwardt, W., Hendricks, K.A., 2004. Dietary nitrites and nitrates, nitrosatable drugs, and neural tube defects. Epidemiology 15, 330–336. <https://doi.org/10.1097/01.ede.0000121381.79831.7b>

Brender, J.D., Suarez, L., Felkner, M., Gilani, Z., Stinchcomb, D., Moody, K., Henry, J., Hendricks, K., 2006. Maternal exposure to arsenic, cadmium, lead, and mercury and neural tube defects in offspring. Environ Res 101, 132–139. <https://doi.org/10.1016/j.envres.2005.08.003>

Brenniman, G.R., Kojola, W.H., Levy, P.S., Carnow, B.W., Namekata, T., 1981. High barium levels in public drinking water and its association with elevated blood pressure. Arch Environ Health 36, 28–32. <https://doi.org/10.1080/00039896.1981.10667602>

Breton, C.V., Kile, M.L., Catalano, P.J., Hoffman, E., Quamruzzaman, Q., Rahman, M., Mahiuddin, G., Christiani, D.C., 2007. GSTM1 and APE1 genotypes affect arsenic-induced oxidative stress: a repeated measures study. Environ Health 6, 39. <https://doi.org/10.1186/1476-069X-6-39>

Broberg, K., Concha, G., Engström, K., Lindvall, M., Grandér, M., Vahter, M., 2011. Lithium in drinking water and thyroid function. Environ Health Perspect 119, 827–830. <https://doi.org/10.1289/ehp.1002678>

Brody, J.G., Aschengrau, A., McKelvey, W., Swartz, C.H., Kennedy, T., Rudel, R.A., 2006. Breast cancer risk and drinking water contaminated by wastewater: a case control study. Environ Health 5, 28. <https://doi.org/10.1186/1476-069X-5-28>

Brown, J., Sobsey, M.D., Loomis, D., 2008. Local drinking water filters reduce diarrheal disease in Cambodia: a randomized, controlled trial of the ceramic water purifier. Am J Trop Med Hyg 79, 394–400.

Brown, S., 2003. Spatial Analysis of Socioeconomic Issues: Gender and GIS in Nepal. Mountain Research and Development 23, 338–344. [https://doi.org/10.1659/0276-4741(2003)023[0338:SAOSIG]2.0.CO;2](https://doi.org/10.1659/0276-4741(2003)023%5b0338:SAOSIG%5d2.0.CO;2)

Browne, M.L., Varadarajulu, D., Lewis-Michl, E.L., Fitzgerald, E.F., 2005. Cancer incidence and asbestos in drinking water, Town of Woodstock, New York, 1980-1998. Environ Res 98, 224–232. <https://doi.org/10.1016/j.envres.2004.07.017>

Bruemmer, B., White, E., Vaughan, T.L., Cheney, C.L., 1997. Fluid intake and the incidence of bladder cancer among middle-aged men and women in a three-county area of western Washington. Nutr Cancer 29, 163–168. <https://doi.org/10.1080/01635589709514619>

Buchet, J.P., Lison, D., 1998. Mortality by cancer in groups of the Belgian population with a moderately increased intake of arsenic. Int Arch Occup Environ Health 71, 125–130. <https://doi.org/10.1007/s004200050259>

Bukhari, N., Saleem, A., Jabbar, A., Khan, S., Ahmad, B., Habib, N., Haseeb, A., Khan, Azhar, Zahid Ali, M., Khan, M., Khan, Aftab, 2016. Frequency of typhoid fever and its association with seasonal variations in Taxila, Pakistan. Asian Pacific Journal of Tropical Disease 6, 608–610. <https://doi.org/10.1016/S2222-1808(16)61094-0>

Bulliyya, G., Dwibedi, B., Mallick, G., Sethy, P.G.S., Kar, S.K., 2008. Determination of iodine nutrition and community knowledge regarding iodine deficiency disorders in selected tribal blocks of Orissa, India. J Pediatr Endocrinol Metab 21, 79–87. <https://doi.org/10.1515/jpem.2008.21.1.79>

Bu-Olayan, A.H., Al-Yakoob, S.N., Alhazeem, S., 1996. Lead in drinking water from water coolers and in fingernails from subjects in Kuwait City, Kuwait. Sci Total Environ 181, 209–214. <https://doi.org/10.1016/0048-9697(95)05011-6>

Burch, J.B., Everson, T.M., Seth, R.K., Wirth, M.D., Chatterjee, S., 2015. Trihalomethane exposure and biomonitoring for the liver injury indicator, alanine aminotransferase, in the United States population (NHANES 1999–2006). Science of The Total Environment 521–522, 226–234. <https://doi.org/10.1016/j.scitotenv.2015.03.050>

Burgess, J.L., Kurzius-Spencer, M., O’Rourke, M.K., Littau, S.R., Roberge, J., Meza-Montenegro, M.M., Gutiérrez-Millán, L.E., Harris, R.B., 2013. Environmental arsenic exposure and serum matrix metalloproteinase-9. J Expo Sci Environ Epidemiol 23, 163–169. <https://doi.org/10.1038/jes.2012.107>

Butts, C.D., Bloom, M.S., Neamtiu, I.A., Surdu, S., Pop, C., Anastasiu, D., Fitzgerald, E.F., Gurzau, E.S., 2015. A pilot study of low-moderate drinking water arsenic contamination and chronic diseases among reproductive age women in Timiş County, Romania. Environ Toxicol Pharmacol 40, 1001–1004. <https://doi.org/10.1016/j.etap.2015.11.003>

Buyukkamaci, N., Alkan, H.S., 2013. Public acceptance potential for reuse applications in Turkey. Resources, Conservation and Recycling 80, 32–35. <https://doi.org/10.1016/j.resconrec.2013.08.001>

Cabada, M.M., Goodrich, M.R., Graham, B., Villanueva-Meyer, P.G., Lopez, M., Arque, E., White, A.C., 2014. Fascioliasis and eosinophilia in the highlands of Cuzco, Peru and their association with water and socioeconomic factors. Am J Trop Med Hyg 91, 989–993. <https://doi.org/10.4269/ajtmh.14-0169>

Cai, Y., Qin, S.-Y., Qian, A., Xu, P., Xu, Y.-T., Xie, L.-H., Zhao, Q., Zhang, X.-X., 2017. Seroprevalence and risk factors of hepatitis E virus infection among the Korean, Manchu, Mongol, and Han ethnic groups in Eastern and Northeastern China. J Med Virol 89, 1988–1994. <https://doi.org/10.1002/jmv.24871>

Calabrese, E.J., Tuthill, R.W., 1985a. The Massachusetts Blood Pressure Study, Part 3. Experimental reduction of sodium in drinking water: effects on blood pressure. Toxicol Ind Health 1, 19–34. <https://doi.org/10.1177/074823378500100103>

Calabrese, E.J., Tuthill, R.W., 1985b. The Massachusetts Blood Pressure Study, Part 1. Elevated levels of sodium in drinking water and blood pressure levels in children. Toxicol Ind Health 1, 1–10. <https://doi.org/10.1177/074823378500100101>

Calabrese, E.J., Tuthill, R.W., 1981. The influence of elevated levels of sodium in drinking water on elementary and high school students in Massachusetts. Sci Total Environ 18, 117–133. <https://doi.org/10.1016/s0048-9697(81)80054-x>

Calafat, A.M., Kuklenyik, Z., Caudill, S.P., Ashley, D.L., 2003. Urinary levels of trichloroacetic acid, a disinfection by-product in chlorinated drinking water, in a human reference population. Environ Health Perspect 111, 151–154. <https://doi.org/10.1289/ehp.5644>

Calderon, R.L., Hudgens, E., Le, X.C., Schreinemachers, D., Thomas, D.J., 1999. Excretion of arsenic in urine as a function of exposure to arsenic in drinking water. Environ Health Perspect 107, 663–667. <https://doi.org/10.1289/ehp.99107663>

Calderon, R.L., Hudgens, E.E., Carty, C., He, B., Le, X.C., Rogers, J., Thomas, D.J., 2013. Biological and behavioral factors modify biomarkers of arsenic exposure in a U.S. population. Environ Res 126, 134–144. <https://doi.org/10.1016/j.envres.2013.04.004>

Caldwell, B.K., Smith, W.T., Lokuge, K., Ranmuthugala, G., Dear, K., Milton, A.H., Sim, M.R., Ng, J.C., Mitra, S.N., 2006. Access to drinking-water and arsenicosis in Bangladesh. J Health Popul Nutr 24, 336–345.

Campos, R., Montenegro-Bethancourt, G., Vossenaar, M., Doak, C.M., Solomons, N.W., 2009. Volume, frequency and participation in plain drinking water consumption by third and fourth-grade schoolchildren in Quetzaltenango, Guatemala. Asia Pac J Clin Nutr 18, 164–170.

Cañete Villafranca, R., Campos, Y., Valdes, R., Rodriguez, P., 2015. Prevalence and Factors Associated with Intestinal Parasitic Infection among School Children from Jagüey Grande Municipality in Matanzas Province, Cuba. West Indian Medical Journal 66. <https://doi.org/10.7727/wimj.2014.288>

Cantor, K.P., Lynch, C.F., Hildesheim, M.E., Dosemeci, M., Lubin, J., Alavanja, M., Craun, G., 1999. Drinking water source and chlorination byproducts in Iowa. III. Risk of brain cancer. Am J Epidemiol 150, 552–560. <https://doi.org/10.1093/oxfordjournals.aje.a010052>

Cantor, K.P., Lynch, C.F., Hildesheim, M.E., Dosemeci, M., Lubin, J., Alavanja, M., Craun, G., 1998. Drinking water source and chlorination byproducts. I. Risk of bladder cancer. Epidemiology 9, 21–28.

Cao, W.-C., Zeng, Q., Luo, Y., Chen, H.-X., Miao, D.-Y., Li, L., Cheng, Y.-H., Li, M., Wang, F., You, L., Wang, Y.-X., Yang, P., Lu, W.-Q., 2016. Blood Biomarkers of Late Pregnancy Exposure to Trihalomethanes in Drinking Water and Fetal Growth Measures and Gestational Age in a Chinese Cohort. Environ Health Perspect 124, 536–541. <https://doi.org/10.1289/ehp.1409234>

Cardoso, O.O., Julião, F.C., Alves, R.I.S., Baena, A.R., Díez, I.G., Suzuki, M.N., Celere, B.S., Nadal, M., Domingo, J.L., Segura-Muñoz, S.I., 2014. Concentration profiles of metals in breast milk, drinking water, and soil: relationship between matrices. Biol Trace Elem Res 160, 116–122. <https://doi.org/10.1007/s12011-014-0030-8>

Carrington, C.D., Bolger, P.M., Scheuplein, R.J., 1996. Risk analysis of dietary lead exposure. Food Additives & Contaminants 13, 61–76. <https://doi.org/10.1080/02652039609374381>

Carrique-Mas, J., Andersson, Y., Hjertqvist, M., Svensson, A., Torner, A., Giesecke, J., 2005. Risk factors for domestic sporadic campylobacteriosis among young children in Sweden. Scand J Infect Dis 37, 101–110. <https://doi.org/10.1080/00365540510027165>

Carter, F., Seaton, T., Yuan, Y., Armstrong, D., 2012. Prevalence of Helicobacter pylori infection in children in the Bahamas. West Indian Med J 61, 698–702.

Carwile, J.L., Mahalingaiah, S., Winter, M.R., Aschengrau, A., 2014. Prenatal drinking-water exposure to tetrachloroethylene and ischemic placental disease: a retrospective cohort study. Environmental Health 13, 72. <https://doi.org/10.1186/1476-069X-13-72>

Cau, B.M., Falcão, J., Arnaldo, C., 2016. Determinants of poor self-rated health among adults in urban Mozambique. BMC Public Health 16, 856. <https://doi.org/10.1186/s12889-016-3552-5>

Cauley, J.A., Buhari, A.M., Murphy, P.A., Riley, T.J., 1995. Effects of fluoridated drinking water on bone mass and fractures: The study of osteoporotic fractures. J Bone Miner Res 10, 1076–1086. <https://doi.org/10.1002/jbmr.5650100712>

Cavalcante, G.T., Aguiar, D.M., Camargo, L.M.A., Labruna, M.B., de Andrade, H.F., Meireles, L.R., Dubey, J.P., Thulliez, P., Dias, R.A., Gennari, S.M., 2006. Seroprevalence of Toxoplasma gondii Antibodies in Humans From Rural Western Amazon, Brazil. Journal of Parasitology 92, 647–649. <https://doi.org/10.1645/GE-774R.1>

Cech, I., Holguin, A.H., Littell, A.S., Henry, J.P., O’Connell, J., 1987. Health Significance of Chlorination Byproducts in Drinking Water: The Houston Experience. International Journal of Epidemiology 16, 198–207. <https://doi.org/10.1093/ije/16.2.198>

Cedergren, M.I., Selbing, A.J., Löfman, O., Källen, B.A.J., 2002. Chlorination byproducts and nitrate in drinking water and risk for congenital cardiac defects. Environ Res 89, 124–130. <https://doi.org/10.1006/enrs.2001.4362>

Chakraborti, D., Mukherjee, S.C., Pati, S., Sengupta, M.K., Rahman, M.M., Chowdhury, U.K., Lodh, D., Chanda, C.R., Chakraborti, A.K., Basu, G.K., 2003. Arsenic groundwater contamination in Middle Ganga Plain, Bihar, India: a future danger? Environ Health Perspect 111, 1194–1201.

Chakraborti, D., Rahman, M.M., Ahamed, S., Dutta, R.N., Pati, S., Mukherjee, S.C., 2016a. Arsenic contamination of groundwater and its induced health effects in Shahpur block, Bhojpur district, Bihar state, India: risk evaluation. Environ Sci Pollut Res Int 23, 9492–9504. <https://doi.org/10.1007/s11356-016-6149-8>

Chakraborti, D., Rahman, M.M., Ahamed, S., Dutta, R.N., Pati, S., Mukherjee, S.C., 2016b. Arsenic groundwater contamination and its health effects in Patna district (capital of Bihar) in the middle Ganga plain, India. Chemosphere 152, 520–529. <https://doi.org/10.1016/j.chemosphere.2016.02.119>

Chami, G.F., Kontoleon, A.A., Bulte, E., Fenwick, A., Kabatereine, N.B., Tukahebwa, E.M., Dunne, D.W., 2016. Profiling Nonrecipients of Mass Drug Administration for Schistosomiasis and Hookworm Infections: A Comprehensive Analysis of Praziquantel and Albendazole Coverage in Community-Directed Treatment in Uganda. Clin Infect Dis 62, 200–207. <https://doi.org/10.1093/cid/civ829>

Chang, C.-C., Chen, C.-C., Wu, D.-C., Yang, C.-Y., 2010. Nitrates in drinking water and the risk of death from rectal cancer: does hardness in drinking water matter? J Toxicol Environ Health A 73, 1337–1347. <https://doi.org/10.1080/15287394.2010.490178>

Chang, C.-C., Ho, S.-C., Wang, L.-Y., Yang, C.-Y., 2007. Bladder cancer in Taiwan: relationship to trihalomethane concentrations present in drinking-water supplies. J Toxicol Environ Health A 70, 1752–1757. <https://doi.org/10.1080/15287390701459031>

Channa, K., Odland, J.Ø., Kootbodien, T., Theodorou, P., Naik, I., Sandanger, T.M., Röllin, H.B., 2013. Differences in prenatal exposure to mercury in South African communities residing along the Indian Ocean. Sci Total Environ 463–464, 11–19. <https://doi.org/10.1016/j.scitotenv.2013.05.055>

Chaturvedi, R., Banerjee, S., Chattopadhyay, P., Bhattacharjee, C.R., Raul, P., Borah, K., 2014. High iron accumulation in hair and nail of people living in iron affected areas of Assam, India. Ecotoxicology and Environmental Safety 110, 216–220. <https://doi.org/10.1016/j.ecoenv.2014.08.028>

Chen, C.J., Chen, C.W., Wu, M.M., Kuo, T.L., 1992. Cancer potential in liver, lung, bladder and kidney due to ingested inorganic arsenic in drinking water. Br J Cancer 66, 888–892. <https://doi.org/10.1038/bjc.1992.380>

Chen, C.J., Chuang, Y.C., Lin, T.M., Wu, H.Y., 1985. Malignant neoplasms among residents of a blackfoot disease-endemic area in Taiwan: high-arsenic artesian well water and cancers. Cancer Res 45, 5895–5899.

Chen, C.-J., Hsueh, Y.-M., Lai, M.-S., Shyu, M.-P., Chen, S.-Y., Wu, M.-M., Kuo, T.-L., Tai, T.-Y., 1995. Increased Prevalence of Hypertension and Long-term Arsenic Exposure. Hypertension 25, 53–60. <https://doi.org/10.1161/01.HYP.25.1.53>

Chen, H., Zhang, Y., Ma, L., Liu, F., Zheng, W., Shen, Q., Zhang, H., Wei, X., Tian, D., He, G., Qu, W., 2012. Change of water consumption and its potential influential factors in Shanghai: A cross-sectional study. BMC Public Health 12, 450. <https://doi.org/10.1186/1471-2458-12-450>

Chen, J., Wu, H., Qian, H., Gao, Y., 2017. Assessing Nitrate and Fluoride Contaminants in Drinking Water and Their Health Risk of Rural Residents Living in a Semiarid Region of Northwest China. Exposure and Health 9, 183–195. <https://doi.org/10.1007/s12403-016-0231-9>

Chen, W., Li, X., Wu, Y., Bian, J., Shen, J., Jiang, W., Tan, L., Wang, X., Wang, W., Pearce, E.N., Zimmermann, M.B., Carriquiry, A.L., Zhang, W., 2017. Associations between iodine intake, thyroid volume, and goiter rate in school-aged Chinese children from areas with high iodine drinking water concentrations. Am J Clin Nutr 105, 228–233. <https://doi.org/10.3945/ajcn.116.139725>

Chen, W., Sang, Z., Tan, L., Zhang, S., Dong, F., Chu, Z., Wei, W., Zhao, N., Zhang, G., Yao, Z., Shen, J., Zhang, W., 2015. Neonatal thyroid function born to mothers living with long-term excessive iodine intake from drinking water. Clin Endocrinol (Oxf) 83, 399–404. <https://doi.org/10.1111/cen.12625>

Chen, Y., Chen, W., Du, C., Fan, L., Wang, W., Gao, M., Zhang, Y., Cui, T., Hao, Y., Pearce, E.N., Wang, C., Zhang, W., 2019. Iodine Nutrition and Thyroid Function in Pregnant Women Exposed to Different Iodine Sources. Biol Trace Elem Res 190, 52–59. <https://doi.org/10.1007/s12011-018-1530-8>

Chen, Y., Graziano, J.H., Parvez, F., Hussain, I., Momotaj, H., van Geen, A., Howe, G.R., Ahsan, H., 2006. Modification of risk of arsenic-induced skin lesions by sunlight exposure, smoking, and occupational exposures in Bangladesh. Epidemiology 17, 459–467. <https://doi.org/10.1097/01.ede.0000220554.50837.7f>

Chen, Y., Graziano, J.H., Parvez, F., Liu, M., Slavkovich, V., Kalra, T., Argos, M., Islam, T., Ahmed, A., Rakibuz-Zaman, M., Hasan, R., Sarwar, G., Levy, D., van Geen, A., Ahsan, H., 2011. Arsenic exposure from drinking water and mortality from cardiovascular disease in Bangladesh: prospective cohort study. BMJ 342, d2431. <https://doi.org/10.1136/bmj.d2431>

Chen, Y., Wu, F., Liu, M.L., Segers, S., Ahsan, H., Parvez, F., Slavkovich, V., Levy, D., Graziano, J.H., Van Geen, A., Islam, T., Ahmed, A., Rakibuz-Zaman, M., Hasan, R., Sarwar, G., 2012. An update of findings on arsenic exposure and cardiovascular disease outcomes from the Health Effects of Arsenic Longitudinal Study (HEALS): 4th International Congress on Arsenic in the Environment, As 2012. Understanding the Geological and Medical Interface of Arsenic, As 2012 - 4th International Congress, Understanding the Geological and Medical Interface of Arsenic, As 2012 - 4th International Congress: Arsenic in the Environment 165–167. <https://doi.org/10.1201/b12522-67>

Chen, Y., Wu, F., Parvez, F., Ahmed, A., Eunus, M., McClintock, T.R., Patwary, T.I., Islam, T., Ghosal, A.K., Islam, S., Hasan, R., Levy, D., Sarwar, G., Slavkovich, V., van Geen, A., Graziano, J.H., Ahsan, H., 2013. Arsenic Exposure from Drinking Water and QT-Interval Prolongation: Results from the Health Effects of Arsenic Longitudinal Study. Environ Health Perspect 121, 427–432. <https://doi.org/10.1289/ehp.1205197>

Chen, Y.-J., Liu, C., Huang, L.-L., Ai, S.-H., Sun, L., Huang, Z., Li, J., Lei, H.-S., Liu, J., Liu, Y.-A., Wang, X., Liu, X.-Y., Cheng, Y.-H., Wang, Y.-X., Pan, A., Lu, W.-Q., 2019. First-trimester blood concentrations of drinking water trihalomethanes and neonatal neurobehavioral development in a Chinese birth cohort. J Hazard Mater 362, 451–457. <https://doi.org/10.1016/j.jhazmat.2018.09.040>

Cheng, M.-H., Chiu, H.-F., Tsai, S.-S., Chen, C.-C., Yang, C.-Y., 2012. Calcium and magnesium in drinking-water and risk of death from lung cancer in women. Magnes Res 25, 112–119. <https://doi.org/10.1684/mrh.2012.0318>

Cheng, T.-J., Ke, D.-S., Guo, H.-R., 2010. The association between arsenic exposure from drinking water and cerebrovascular disease mortality in Taiwan. Water Research, Groundwater Arsenic: From Genesis to Sustainable Remediation 44, 5770–5776. <https://doi.org/10.1016/j.watres.2010.05.040>

Cherian, A., Syam, U.K., Sreevidya, D., Jayaraman, T., Oommen, A., Rajshekhar, V., Radhakrishnan, K., Thomas, S.V., 2014. Low seroprevalence of systemic cysticercosis among patients with epilepsy in Kerala--South India. J Infect Public Health 7, 271–276. <https://doi.org/10.1016/j.jiph.2013.08.005>

Cherinet, A., Kelbessa, U., 2000. Determinants of iodine deficiency in school children in different regions of Ethiopia. East Afr Med J 77, 133–137. <https://doi.org/10.4314/eamj.v77i3.46608>

Cherry, N., McDonald, C., Chowdhury, Z., 2012. Zinc in well water and infant mortality in bangladesh: a report from gonoshasthaya kendra. Int J Environ Res Public Health 9, 171–177. <https://doi.org/10.3390/ijerph9010171>

Cherry, N., Shaikh, K., McDonald, C., Chowdhury, Z., 2008. Stillbirth in rural Bangladesh: arsenic exposure and other etiological factors: a report from Gonoshasthaya Kendra. Bull World Health Organ 86, 172–177. <https://doi.org/10.2471/blt.07.043083>

Chervona, Y., Hall, M.N., Arita, A., Wu, F., Sun, H., Tseng, H.-C., Ali, E., Uddin, M.N., Liu, X., Zoroddu, M.A., Gamble, M.V., Costa, M., 2012. Association Between Arsenic Exposure and Global Post-translational Histone Modifications Among Adults in Bangladesh. Cancer Epidemiol Biomarkers Prev 21, 2252–2260. <https://doi.org/10.1158/1055-9965.EPI-12-0833>

Chevrier, C., Serrano, T., Lecerf, R., Limon, G., Petit, C., Monfort, C., Hubert-Moy, L., Durand, G., Cordier, S., 2014. Environmental determinants of the urinary concentrations of herbicides during pregnancy: the PELAGIE mother-child cohort (France). Environ Int 63, 11–18. <https://doi.org/10.1016/j.envint.2013.10.010>

Chiou, H.Y., Hsueh, Y.M., Hsieh, L.L., Hsu, L.I., Hsu, Y.H., Hsieh, F.I., Wei, M.L., Chen, H.C., Yang, H.T., Leu, L.C., Chu, T.H., Chen-Wu, C., Yang, M.H., Chen, C.J., 1997a. Arsenic methylation capacity, body retention, and null genotypes of glutathione S-transferase M1 and T1 among current arsenic-exposed residents in Taiwan. Mutat Res 386, 197–207. <https://doi.org/10.1016/s1383-5742(97)00005-7>

Chiou, H.Y., Huang, W.I., Su, C.L., Chang, S.F., Hsu, Y.H., Chen, C.J., 1997b. Dose-response relationship between prevalence of cerebrovascular disease and ingested inorganic arsenic. Stroke 28, 1717–1723. <https://doi.org/10.1161/01.str.28.9.1717>

Chiou, J.-M., Wang, S.-L., Chen, C.-J., Deng, C.-R., Lin, W., Tai, T.-Y., 2005. Arsenic ingestion and increased microvascular disease risk: observations from the south-western arseniasis-endemic area in Taiwan. International Journal of Epidemiology 34, 936–943. <https://doi.org/10.1093/ije/dyi108>

Chirande, L., Charwe, D., Mbwana, H., Victor, R., Kimboka, S., Issaka, A.I., Baines, S.K., Dibley, M.J., Agho, K.E., 2015. Determinants of stunting and severe stunting among under-fives in Tanzania: evidence from the 2010 cross-sectional household survey. BMC Pediatr 15, 165. <https://doi.org/10.1186/s12887-015-0482-9>

Chisholm, K., Cook, A., Bower, C., Weinstein, P., 2008. Risk of Birth Defects in Australian Communities with High Levels of Brominated Disinfection By-products. Environmental Health Perspectives 116, 1267–1273. <https://doi.org/10.1289/ehp.10980>

Chiu, H.-F., Chang, C.-C., Chen, C.-C., Yang, C.-Y., 2011. Calcium and magnesium in drinking water and risk of death from kidney cancer. J Toxicol Environ Health A 74, 62–70. <https://doi.org/10.1080/15287394.2010.514236>

Chiu, H.-F., Chang, C.-C., Tsai, S.-S., Yang, C.-Y., 2006. Does arsenic exposure increase the risk for diabetes mellitus? J Occup Environ Med 48, 63–67. <https://doi.org/10.1097/01.jom.0000184854.75053.03>

Chiu, H.-F., Tsai, S.-S., Wu, T.-N., Yang, C.-Y., 2010. Effect modification of the association between trihalomethanes and pancreatic cancer by drinking water hardness: evidence from an ecological study. Environ Res 110, 513–518. <https://doi.org/10.1016/j.envres.2010.03.007>

Chiu, H.-F., Tsai, S.-S., Yang, C.-Y., 2007. Nitrate in drinking water and risk of death from bladder cancer: an ecological case-control study in Taiwan. J Toxicol Environ Health A 70, 1000–1004. <https://doi.org/10.1080/15287390601171801>

Choubisa, S., 2012. Fluoride in Drinking Water and its Toxicosis in Tribals of Rajasthan, India. Proceedings of the National Academy of Sciences, India Section B: Biological Sciences 82. <https://doi.org/10.1007/s40011-012-0047-8>

Choudhury, M.I.M., Shabnam, N., Ahsan, T., Ahsan, S.M.A., Kabir, M.S., Khan, R.M., Miah, M.A., Uddin, M.K., Liton, M.A.R., 2018. Cutaneous Malignancy due to Arsenicosis in Bangladesh: 12-Year Study in Tertiary Level Hospital. Biomed Res Int 2018, 4678362. <https://doi.org/10.1155/2018/4678362>

Chowdhury, F., Khan, I.A., Patel, S., Siddiq, A.U., Saha, N.C., Khan, A.I., Saha, A., Cravioto, A., Clemens, J., Qadri, F., Ali, M., 2015. Diarrheal Illness and Healthcare Seeking Behavior among a Population at High Risk for Diarrhea in Dhaka, Bangladesh. PLoS One 10, e0130105. <https://doi.org/10.1371/journal.pone.0130105>

Christen, A., Duran Pacheco, G., Hattendorf, J., Arnold, B.F., Cevallos, M., Indergand, S., Colford, J.M., Mäusezahl, D., 2011. Factors associated with compliance among users of solar water disinfection in rural Bolivia. BMC Public Health 11, 210. <https://doi.org/10.1186/1471-2458-11-210>

Christian, W.J., Hopenhayn, C., Centeno, J.A., Todorov, T., 2006. Distribution of urinary selenium and arsenic among pregnant women exposed to arsenic in drinking water. Environ Res 100, 115–122. <https://doi.org/10.1016/j.envres.2005.03.009>

Chung, Y.-L., Liaw, Y.-P., Hwang, B.-F., Cheng, Y.-Y., Lin, M.-S., Kuo, Y.-C., Guo, H.-R., 2013. Arsenic in drinking and lung cancer mortality in Taiwan. Journal of Asian Earth Sciences 77, 327–331. <https://doi.org/10.1016/j.jseaes.2013.04.038>

Cifuentes, E., Suárez, L., Espinosa, M., Juárez-Figueroa, L., Martínez-Palomo, A., 2004. Risk of Giardia intestinalis infection in children from an artificially recharged groundwater area in Mexico City. Am J Trop Med Hyg 71, 65–70.

Clark, W.F., Huang, S.-H., Garg, A.X., Gallo, K., House, A.A., Moist, L., Weir, M.A., Sontrop, J.M., 2017. The Chronic Kidney Disease Water Intake Trial: Protocol of a Randomized Controlled Trial. Can J Kidney Health Dis 4, 2054358117725106. <https://doi.org/10.1177/2054358117725106>

Clasen, T.F., Bastable, A., 2003. Faecal contamination of drinking water during collection and household storage: the need to extend protection to the point of use. J Water Health 1, 109–115.

Cohen, A., 2020. Rural China Electric Kettle Promotion Program: A Cluster-Randomized Controlled Trial of Pilot Program Impact on Electric Kettle Adoption, Drinking Water Quality, Indoor Air Pollution, and Reported Health Outcomes in Poverty Households in the People’s Republic of China (Clinical trial registration No. NCT03376152). clinicaltrials.gov.

Cohen, A., Zhang, Q., Luo, Q., Tao, Y., Colford, J.M., Ray, I., 2017. Predictors of Drinking Water Boiling and Bottled Water Consumption in Rural China: A Hierarchical Modeling Approach. Environ Sci Technol 51, 6945–6956. <https://doi.org/10.1021/acs.est.7b01006>

Colli, J.L., Kolettis, P.N., 2010. Bladder cancer incidence and mortality rates compared to ecologic factors among states in America. Int Urol Nephrol 42, 659–665. <https://doi.org/10.1007/s11255-009-9655-5>

Concha, G., Broberg, K., Grandér, M., Cardozo, A., Palm, B., Vahter, M., 2010. High-level exposure to lithium, boron, cesium, and arsenic via drinking water in the Andes of northern Argentina. Environ Sci Technol 44, 6875–6880. <https://doi.org/10.1021/es1010384>

Concha, G., Nermell, B., Vahter, M., 2006. Spatial and temporal variations in arsenic exposure via drinking-water in northern Argentina. J Health Popul Nutr 24, 317–326.

Concha, G, Nermell, B., Vahter, M., 1998. Metabolism of inorganic arsenic in children with chronic high arsenic exposure in northern Argentina. Environmental Health Perspectives 106, 355–359.

Concha, Gabriela, Vogler, G., Lezcano, D., Nermell, B., Vahter, M., 1998a. Exposure to Inorganic Arsenic Metabolites during Early Human Development. Toxicol Sci 44, 185–190. <https://doi.org/10.1093/toxsci/44.2.185>

Concha, Gabriela, Vogler, G., Nermell, B., Vahter, M., 1998b. Low-level arsenic excretion in breast milk of native Andean women exposed to high levels of arsenic in the drinking water. International Archives of Occupational and Environmental Health 71, 42–46. <https://doi.org/10.1007/s004200050248>

Conforti, P.M., Kanarek, M.S., Jackson, L.A., Cooper, R.C., Murchio, J.C., 1981. Asbestos in drinking water and cancer in the San Francisco bay area: 1969–1974 incidence. Journal of Chronic Diseases 34, 211–224. <https://doi.org/10.1016/0021-9681(81)90065-5>

Corradini, S.G., Ferri, F., Mordenti, M., Iuliano, L., Siciliano, M., Burza, M.A., Sordi, B., Caciotti, B., Pacini, M., Poli, E., Santis, A.D., Roda, A., Colliva, C., Simoni, P., Attili, A.F., 2012. Beneficial effect of sulphate-bicarbonate-calcium water on gallstone risk and weight control. World J Gastroenterol 18, 930–937. <https://doi.org/10.3748/wjg.v18.i9.930>

Coss, A., Cantor, K.P., Reif, J.S., Lynch, C.F., Ward, M.H., 2004. Pancreatic cancer and drinking water and dietary sources of nitrate and nitrite. Am J Epidemiol 159, 693–701. <https://doi.org/10.1093/aje/kwh081>

Costet, N., Garlantézec, R., Monfort, C., Rouget, F., Gagnière, B., Chevrier, C., Cordier, S., 2012. Environmental and urinary markers of prenatal exposure to drinking water disinfection by-products, fetal growth, and duration of gestation in the PELAGIE birth cohort (Brittany, France, 2002-2006). Am J Epidemiol 175, 263–275. <https://doi.org/10.1093/aje/kwr419>

Costi, D., Calcaterra, P.G., Iori, N., Vourna, S., Nappi, G., Passeri, M., 1999. Importance of bioavailable calcium drinking water for the maintenance of bone mass in post-menopausal women. J Endocrinol Invest 22, 852–856. <https://doi.org/10.1007/BF03343658>

Cragin, L.A., Kesner, J.S., Bachand, A.M., Barr, D.B., Meadows, J.W., Krieg, E.F., Reif, J.S., 2011. Menstrual cycle characteristics and reproductive hormone levels in women exposed to atrazine in drinking water. Environ Res 111, 1293–1301. <https://doi.org/10.1016/j.envres.2011.09.009>

Cubadda, F., Aureli, F., D’Amato, M., Raggi, A., Turco, A.C., Mantovani, A., 2012. Speciated urinary arsenic as a biomarker of dietary exposure to inorganic arsenic in residents living in high-arsenic areas in Latium, Italy. Pure and Applied Chemistry 84, 203–214. <https://doi.org/10.1351/PAC-CON-11-09-29>

Cummings, M.J., Wamala, J.F., Eyura, M., Malimbo, M., Omeke, M.E., Mayer, D., Lukwago, L., 2012. A cholera outbreak among semi-nomadic pastoralists in northeastern Uganda: epidemiology and interventions. Epidemiol Infect 140, 1376–1385. <https://doi.org/10.1017/S0950268811001956>

Curtis, V., Dreibelbis, R., Buxton, H., Izang, N., Adekunle, D., Aunger, R., 2019. Behaviour settings theory applied to domestic water use in Nigeria: A new conceptual tool for the study of routine behaviour. Soc Sci Med 235, 112398. <https://doi.org/10.1016/j.socscimed.2019.112398>

Dahl, Cecilie, Søgaard, A.J., Tell, G.S., Flaten, T.P., Hongve, D., Omsland, T.K., Holvik, K., Meyer, H.E., Aamodt, G., 2013. Nationwide data on municipal drinking water and hip fracture: could calcium and magnesium be protective? A NOREPOS study. Bone 57, 84–91. <https://doi.org/10.1016/j.bone.2013.06.017>

Dahl, C., Søgaard, A.J., Tell, G.S., Flaten, T.P., Hongve, D., Omsland, T.K., Holvik, K., Meyer, H.E., Aamodt, G., Norwegian Epidemiologic Osteoporosis Study (NOREPOS) Core Research Group, 2014. Do cadmium, lead, and aluminum in drinking water increase the risk of hip fractures? A NOREPOS study. Biol Trace Elem Res 157, 14–23. <https://doi.org/10.1007/s12011-013-9862-x>

Dahl, C., Søgaard, A.J., Tell, G.S., Flaten, T.P., Krogh, T., Aamodt, G., NOREPOS Core Research Group, 2013. Is the quality of drinking water a risk factor for self-reported forearm fractures? Cohort of Norway. Osteoporos Int 24, 541–551. <https://doi.org/10.1007/s00198-012-1989-7>

Dahl, C., Søgaard, A.J., Tell, G.S., Forsén, L., Flaten, T.P., Hongve, D., Omsland, T.K., Holvik, K., Meyer, H.E., Aamodt, G., 2015. Population data on calcium in drinking water and hip fracture: An association may depend on other minerals in water. A NOREPOS study. Bone 81, 292–299. <https://doi.org/10.1016/j.bone.2015.07.020>

Dalal, B., Nayak, K., Gangopadhyay, P.K., Mukherjee, A., 2010. Identification of indicators of arsenic induced hepatic damage in human. Internet Journal of Toxicology 7.

Danileviciute, A., Grazuleviciene, R., Vencloviene, J., Paulauskas, A., Nieuwenhuijsen, M.J., 2012. Exposure to drinking water trihalomethanes and their association with low birth weight and small for gestational age in genetically susceptible women. Int J Environ Res Public Health 9, 4470–4485. <https://doi.org/10.3390/ijerph9124470>

Danjou, A., Patel, M., Espina, C., Pentz, A., Joffe, M., Winde, F., Schüz, J., 2019. Prospective case-series analysis of haematological malignancies in goldmining areas in South Africa. S Afr Med J 109, 340–346. <https://doi.org/10.7196/SAMJ.2019.v109i5.13538>

Dan-Nwafor, C.C., Ogbonna, U., Onyiah, P., Gidado, S., Adebobola, B., Nguku, P., Nsubuga, P., 2019. A cholera outbreak in a rural north central Nigerian community: an unmatched case-control study. BMC Public Health 19, 112. <https://doi.org/10.1186/s12889-018-6299-3>

Darrow, L.A., Howards, P.P., Winquist, A., Steenland, K., 2014. PFOA and PFOS serum levels and miscarriage risk. Epidemiology 25, 505–512. <https://doi.org/10.1097/EDE.0000000000000103>

Darrow, L.A., Stein, C.R., Steenland, K., 2013. Serum perfluorooctanoic acid and perfluorooctane sulfonate concentrations in relation to birth outcomes in the Mid-Ohio Valley, 2005-2010. Environ Health Perspect 121, 1207–1213. <https://doi.org/10.1289/ehp.1206372>

Darsow, U., Fedorov, M., Schwegler, U., Twardella, D., Schaller, K.-H., Habernegg, R., Fromme, H., Ring, J., Behrendt, H., 2012. Influence of dietary factors, age and nickel contact dermatitis on nickel excretion. Contact Dermatitis 67, 351–358. <https://doi.org/10.1111/j.1600-0536.2012.02153.x>

Das, K., Ghosh, M., Nag, C., Nandy, S.P., Banerjee, M., Datta, M., Devi, G., Chaterjee, G., 2011. Role of familial, environmental and occupational factors in the development of Parkinson’s disease. Neurodegener Dis 8, 345–351. <https://doi.org/10.1159/000323797>

Datta, S.S., Ramakrishnan, R., Murhekar, M.V., 2012. A rapidly-progressing outbreak of cholera in a shelter-home for mentally-retarded females, amta-II block, Howrah, West Bengal, India. J Health Popul Nutr 30, 109–112. <https://doi.org/10.3329/jhpn.v30i1.11290>

Dauphiné, D.C., Smith, A.H., Yuan, Y., Balmes, J.R., Bates, M.N., Steinmaus, C., 2013. Case-Control Study of Arsenic in Drinking Water and Lung Cancer in California and Nevada. Int J Environ Res Public Health 10, 3310–3324. <https://doi.org/10.3390/ijerph10083310>

De Michele, G., Filla, A., Volpe, G., De Marco, V., Gogliettino, A., Ambrosio, G., Marconi, R., Castellano, A.E., Campanella, G., 1996. Environmental and genetic risk factors in Parkinson’s disease: A case-control study in southern italy. Mov Disord. 11, 17–23. <https://doi.org/10.1002/mds.870110105>

De Moura, F.L., Millar, P.R., Fonseca, A.B.M., Amendoeira, M.R.R., 2017. Congenital toxoplasmosis: perception of knowledge and primary prevention measures among healthcare professionals and pregnant women treated in public healthcare facilities. Sci Med 27, 25389. <https://doi.org/10.15448/1980-6108.2017.1.25389>

de Queiroz, J.T.M., Doria, M. de F., Rosenberg, M.W., Heller, L., Zhouri, A., 2013. Perceptions of bottled water consumers in three Brazilian municipalities. J Water Health 11, 520–531. <https://doi.org/10.2166/wh.2013.222>

Deane, M., Swan, S.H., Harris, J.A., Epstein, D.M., Neutra, R.R., 1989. Adverse pregnancy outcomes in relation to water contamination, Santa Clara County, California, 1980-1981. Am J Epidemiol 129, 894–904. <https://doi.org/10.1093/oxfordjournals.aje.a115223>

Deb, D., Biswas, A., Ghose, A., Das, A., Majumdar, K.K., Guha Mazumder, D.N., 2013a. Nutritional deficiency and arsenical manifestations: a perspective study in an arsenic-endemic region of West Bengal, India. Public Health Nutr. 16, 1644–1655. <https://doi.org/10.1017/S1368980012004697>

Deb, D., Majumdar, K.K., Mazumder, D.N.G., 2013b. Arsenicosis and Dietary Nutrient Intake Among Men and Women. Proc. Natl. Acad. Sci., India, Sect. B Biol. Sci. 83, 405–413. <https://doi.org/10.1007/s40011-013-0161-2>

Deepthi, R., Sandeep, S.R., Rajini, M., Rajeshwari, H., Shetty, A., 2013. Cholera outbreak in a village in south India - Timely action saved lives. J Infect Public Health 6, 35–40. <https://doi.org/10.1016/j.jiph.2012.05.003>

Dehghani, M., Omrani, R., Zamanian, Z., Hashemi, H., 2013. Determination of DMFT index among 7-11 year-old students and its relation with fluoride in Shiraz drinking water in Iran. Pakistan Journal of Medical Sciences 29. <https://doi.org/10.12669/pjms.291(Suppl).3537>

DellaValle, D.M., Roe, L.S., Rolls, B.J., 2005. Does the consumption of caloric and non-caloric beverages with a meal affect energy intake? Appetite 44, 187–193. <https://doi.org/10.1016/j.appet.2004.11.003>

Demie, G., Bekele, M., Seyoum, B., 2016. Water accessibility impact on girl and women’s participation in education and other development activities: the case of Wuchale and Jidda Woreda, Ethiopia. Environmental Systems Research 5, 11. <https://doi.org/10.1186/s40068-016-0061-6>

Deng, Y.-L., Yang, P., Cao, W.-C., Wang, Y.-X., Liu, C., Chen, Y.-J., Huang, L.-L., Lu, W.-Q., Wang, L.-Q., Zeng, Q., 2019. Urinary biomarker of late pregnancy exposure to drinking water disinfection by-products and ultrasound measures of fetal growth in Wuhan, China. Environ Res 170, 128–133. <https://doi.org/10.1016/j.envres.2018.12.018>

Desai, U., 1991. Determinants of Educational Performance in India: Role of Home and Family. International Review of Education/Internationale Zeitschrift fur Erziehungswissenschaft/Revue Internationale de pedagogie 37, 245–265.

Devasia, L., 1998. Safe drinking water and its acquisition: Rural women’s participation in water management in Maharashtra, India. International Journal of Water Resources Development 14, 537.

Devoto, F., Duflo, E., Dupas, P., Parienté, W., Pons, V., 2012. Happiness on Tap: Piped Water Adoption in Urban Morocco. American Economic Journal: Economic Policy 4, 68–99. <https://doi.org/10.1257/pol.4.4.68>

Dhillon, K.S., Dhillon, S.K., 2016. Selenium in groundwater and its contribution towards daily dietary Se intake under different hydrogeological zones of Punjab, India. Journal of Hydrology 533, 615–626. <https://doi.org/10.1016/j.jhydrol.2015.12.016>

Dhingra, R., Darrow, L.A., Klein, M., Winquist, A., Steenland, K., 2016. Perfluorooctanoic acid exposure and natural menopause: A longitudinal study in a community cohort. Environmental Research 146, 323–330. <https://doi.org/10.1016/j.envres.2015.12.037>

Dias, R.C.F., Lopes-Mori, F.M.R., Mitsuka-Breganó, R., Dias, R.A.F., Tokano, D.V., Reiche, E.M.V., Freire, R.L., Navarro, I.T., 2011. Factors associated to infection by Toxoplasma gondii in pregnant women attended in Basic Health Units in the city of Rolândia, Paraná, Brazil. Rev Inst Med Trop Sao Paulo 53, 185–191. <https://doi.org/10.1590/s0036-46652011000400002>

Díaz-Villaseñor, A., Cruz, L., Cebrián, A., Hernández-Ramírez, R.U., Hiriart, M., García-Vargas, G., Bassol, S., Sordo, M., Gandolfi, A.J., Klimecki, W.T., López-Carillo, L., Cebrián, M.E., Ostrosky-Wegman, P., 2013. Arsenic exposure and calpain-10 polymorphisms impair the function of pancreatic beta-cells in humans: a pilot study of risk factors for T2DM. PLoS One 8, e51642. <https://doi.org/10.1371/journal.pone.0051642>

Dion, L.-A., Saint-Amour, D., Sauvé, S., Barbeau, B., Mergler, D., Bouchard, M.F., 2018. Changes in water manganese levels and longitudinal assessment of intellectual function in children exposed through drinking water. Neurotoxicology 64, 118–125. <https://doi.org/10.1016/j.neuro.2017.08.015>

Diouf, K., Tabatabai, P., Rudolph, J., Marx, M., 2014. Diarrhoea prevalence in children under five years of age in rural Burundi: an assessment of social and behavioural factors at the household level. Glob Health Action 7, 24895. <https://doi.org/10.3402/gha.v7.24895>

D’Ippoliti, D., Santelli, E., De Sario, M., Scortichini, M., Davoli, M., Michelozzi, P., 2015. Arsenic in Drinking Water and Mortality for Cancer and Chronic Diseases in Central Italy, 1990-2010. PLoS One 10, e0138182. <https://doi.org/10.1371/journal.pone.0138182>

Dodds, L., King, W., Allen, A.C., Armson, B.A., Fell, D.B., Nimrod, C., 2004. Trihalomethanes in public water supplies and risk of stillbirth. Epidemiology 15, 179–186. <https://doi.org/10.1097/01.ede.0000112209.47765.d9>

Dodds, L., King, W., Woolcott, C., Pole, J., 1999. Trihalomethanes in public water supplies and adverse birth outcomes. Epidemiology 10, 233–237.

Dorsch, M.M., Scragg, R.K., McMichael, A.J., Baghurst, P.A., Dyer, K.F., 1984. Congenital malformations and maternal drinking water supply in rural South Australia: a case-control study. Am J Epidemiol 119, 473–486. <https://doi.org/10.1093/oxfordjournals.aje.a113764>

Doyle, T.J., Zheng, W., Cerhan, J.R., Hong, C.P., Sellers, T.A., Kushi, L.H., Folsom, A.R., 1997. The association of drinking water source and chlorination by-products with cancer incidence among postmenopausal women in Iowa: a prospective cohort study. Am J Public Health 87, 1168–1176. <https://doi.org/10.2105/AJPH.87.7.1168>

Drewnowski, A., Rehm, C.D., Constant, F., 2013. Water and beverage consumption among children age 4-13y in the United States: analyses of 2005-2010 NHANES data. Nutr J 12, 85. <https://doi.org/10.1186/1475-2891-12-85>

Dulout, F.N., Grillo, C.A., Seoane, A.I., Maderna, C.R., Nilsson, R., Vahter, M., Darroudi, F., Natarajan, A.T., 1996. Chromosomal aberrations in peripheral blood lymphocytes from native Andean women and children from northwestern Argentina exposed to arsenic in drinking water. Mutat Res 370, 151–158. <https://doi.org/10.1016/s0165-1218(96)00060-2>

Dupont, C., Campagne, A., Constant, F., 2014. Efficacy and safety of a magnesium sulfate-rich natural mineral water for patients with functional constipation. Clin Gastroenterol Hepatol 12, 1280–1287. <https://doi.org/10.1016/j.cgh.2013.12.005>

Dupont, D., Adamowicz, W.L.V., Krupnick, A., 2010. Differences in water consumption choices in Canada: the role of socio-demographics, experiences, and perceptions of health risks. J Water Health 8, 671–686. <https://doi.org/10.2166/wh.2010.143>

Dutta, K., Prasad, P., Sinha, D., 2015. Chronic low level arsenic exposure evokes inflammatory responses and DNA damage. Int J Hyg Environ Health 218, 564–574. <https://doi.org/10.1016/j.ijheh.2015.06.003>

Ede, A.O., Nwaokoro, J.C., Iwuala, C.C., Amadi, A.N., Akpelu, U.A., 2014. The provision of potable water in eradication of Guinea worm infection in Ezza North, Southeastern, Nigeria. J Community Health 39, 980–986. <https://doi.org/10.1007/s10900-014-9842-x>

Edwards, M., 2014. Fetal Death and Reduced Birth Rates Associated with Exposure to Lead-Contaminated Drinking Water. Environ. Sci. Technol. 48, 739–746. <https://doi.org/10.1021/es4034952>

Egorov, A.I., Tereschenko, A.A., Altshul, L.M., Vartiainen, T., Samsonov, D., LaBrecque, B., Mäki-Paakkanen, J., Drizhd, N.L., Ford, T.E., 2003. Exposures to drinking water chlorination by-products in a Russian city. International Journal of Hygiene and Environmental Health 206, 539–551. <https://doi.org/10.1078/1438-4639-00244>

Eichelberger, L., Murphy, G., Etemadi, A., Abnet, C.C., Islami, F., Shakeri, R., Malekzadeh, R., Dawsey, S.M., 2015. Risk of gastric cancer by water source: evidence from the Golestan case-control study. PLoS One 10, e0128491. <https://doi.org/10.1371/journal.pone.0128491>

El Azar, G.E., Habib, R.R., Mahfoud, Z., El-Fadel, M., Zurayk, R., Jurdi, M., Nuwayhid, I., 2009. Effect of women’s perceptions and household practices on children’s waterborne illness in a low income community. Ecohealth 6, 169–179. <https://doi.org/10.1007/s10393-009-0239-8>

el-Sayed, N.A., Mahfouz, A.A., Nofal, L., Ismail, H.M., Gad, A., Abu Zeid, H., 1998. Iodine deficiency disorders among school children in upper Egypt: an epidemiologic study. J Trop Pediatr 44, 270–274. <https://doi.org/10.1093/tropej/44.5.270>

El-Badry, A.A., Al-Antably, A.S.A., Hassan, M.A., Hanafy, N.A., Abu-Sarea, E.Y., 2015. Molecular seasonal, age and gender distributions of Cryptosporidium in diarrhoeic Egyptians: distinct endemicity. Eur J Clin Microbiol Infect Dis 34, 2447–2453. <https://doi.org/10.1007/s10096-015-2502-y>

El-Damanawi, R., Harris, T., Sandford, R.N., Karet Frankl, F.E., Hiemstra, T.F., 2017. Patient Survey of current water Intake practices in autosomal dominant Polycystic kidney disease: the SIPs survey. Clin Kidney J 10, 305–309. <https://doi.org/10.1093/ckj/sfw153>

Elwood, J.M., Coldman, A.J., 1981. Water composition in the etiology of anencephalus. Am J Epidemiol 113, 681–690. <https://doi.org/10.1093/oxfordjournals.aje.a113148>

Engel, R.R., Smith, A.H., 1994. Arsenic in Drinking Water and Mortality from Vascular Disease: An Ecologic Analysis in 30 Counties in the United States. Archives of Environmental Health: An International Journal 49, 418–427. <https://doi.org/10.1080/00039896.1994.9954996>

Engelaer, F.M., Koopman, J.J.E., van Bodegom, D., Eriksson, U.K., Westendorp, R.G.J., 2014. Determinants of epidemiologic transition in rural Africa: the role of socioeconomic status and drinking water source. Transactions of The Royal Society of Tropical Medicine and Hygiene 108, 372–379. <https://doi.org/10.1093/trstmh/tru053>

Engström, K., Vahter, M., Mlakar, S.J., Concha, G., Nermell, B., Raqib, R., Cardozo, A., Broberg, K., 2011. Polymorphisms in arsenic(+III oxidation state) methyltransferase (AS3MT) predict gene expression of AS3MT as well as arsenic metabolism. Environ Health Perspect 119, 182–188. <https://doi.org/10.1289/ehp.1002471>

Engström, K.S., Hossain, M.B., Lauss, M., Ahmed, S., Raqib, R., Vahter, M., Broberg, K., 2013. Efficient arsenic metabolism--the AS3MT haplotype is associated with DNA methylation and expression of multiple genes around AS3MT. PLoS One 8, e53732. <https://doi.org/10.1371/journal.pone.0053732>

Erdei, E., Shuey, C., Pacheco, B., Cajero, M., Lewis, J., Rubin, R.L., 2019. Elevated autoimmunity in residents living near abandoned uranium mine sites on the Navajo Nation. J Autoimmun 99, 15–23. <https://doi.org/10.1016/j.jaut.2019.01.006>

Erickson, J.D., 1980. Down syndrome, water fluoridation, and maternal age. Teratology 21, 177–180. <https://doi.org/10.1002/tera.1420210207>

Espejo-Herrera, N., Gràcia-Lavedan, E., Boldo, E., Aragonés, N., Pérez-Gómez, B., Pollán, M., Molina, A.J., Fernández, T., Martín, V., La Vecchia, C., Bosetti, C., Tavani, A., Polesel, J., Serraino, D., Gómez Acebo, I., Altzibar, J.M., Ardanaz, E., Burgui, R., Pisa, F., Fernández-Tardón, G., Tardón, A., Peiró, R., Navarro, C., Castaño-Vinyals, G., Moreno, V., Righi, E., Aggazzotti, G., Basagaña, X., Nieuwenhuijsen, M., Kogevinas, M., Villanueva, C.M., 2016. Colorectal cancer risk and nitrate exposure through drinking water and diet. Int J Cancer 139, 334–346. <https://doi.org/10.1002/ijc.30083>

Esteban, J.G., González, C., Bargues, M.D., Angles, R., Sánchez, C., Náquira, C., Mas-Coma, S., 2002. High fascioliasis infection in children linked to a man-made irrigation zone in Peru. Trop Med Int Health 7, 339–348. <https://doi.org/10.1046/j.1365-3156.2002.00870.x>

Eu, A., Rs, H., Sd, M., 2010. Knowledge and risk factors of intestinal parasitic infections among women in Makurdi, Benue State. Asian Pacific Journal of Tropical Medicine 3, 993–996. <https://doi.org/10.1016/S1995-7645(11)60016-3>

Fabiani, L., Leoni, V., Vitali, M., 1999. Bone-fracture incidence rate in two Italian regions with different fluoride concentration levels in drinking water. J Trace Elem Med Biol 13, 232–237. <https://doi.org/10.1016/S0946-672X(99)80041-8>

Fagerli, K., O’Connor, K., Kim, S., Kelley, M., Odhiambo, A., Faith, S., Otieno, R., Nygren, B., Kamb, M., Quick, R., 2017. Impact of the Integration of Water Treatment, Hygiene, Nutrition, and Clean Delivery Interventions on Maternal Health Service Use. Am J Trop Med Hyg 96, 1253–1260. <https://doi.org/10.4269/ajtmh.16-0709>

Fagliano, J., Berry, M., Bove, F., Burke, T., 1990. Drinking water contamination and the incidence of leukemia: an ecologic study. Am J Public Health 80, 1209–1212. <https://doi.org/10.2105/ajph.80.10.1209>

Fajardo, Val Andrew, Fajardo, Val Andrei, LeBlanc, P.J., MacPherson, R.E.K., 2018. Examining the Relationship between Trace Lithium in Drinking Water and the Rising Rates of Age-Adjusted Alzheimer’s Disease Mortality in Texas. J Alzheimers Dis 61, 425–434. <https://doi.org/10.3233/JAD-170744>

Fakhri, Y., Gea, O.C., Ferrante, M., Bay, A., Avazpour, M., Moradi, B., Zandsalimi, Y., Amirhajeloo, L.R., Langarizadeh, G., Keramati, H., 2016. Assessment of concentration of radon 222 and effective dose; Bandar Abbas city (Iran) citizens exposed through drinking tap water. International Journal of Pharmacy and Technology 8, 10782–10793.

Fakhri, Y., Mohseni, S.M., Jafarzadeh, S., Langarizadeh, G., Moradi, B., Zandsalimi, Y., Rahimizadeh, A., Mirzaei, M., 2015. Assessment of carcinogenic and non-carcinogenic risk lead in bottled water in different age groups in Bandar Abbas Ciry, Iran. Glob J Health Sci 7, 286–294. <https://doi.org/10.5539/gjhs.v7n4p286>

Fakhri, Y., Mousavi Khaneghah, A., Hadiani, M.R., Keramati, H., Hosseini Pouya, R., Moradi, B., da Silva, B.S., 2017. Non-carcinogenic risk assessment induced by heavy metals content of the bottled water in Iran. Toxin Reviews 36, 313–321. <https://doi.org/10.1080/15569543.2017.1358747>

Fan, C.-K., Lee, L.-W., Liao, C.-W., Huang, Y.-C., Lee, Y.-L., Chang, Y.-T., da Costa, Â. dos S.R.J., Gil, V., Chi, L.-H., Nara, T., Tsubouchi, A., Akinwale, O.P., 2012. Toxoplasma gondii infection: relationship between seroprevalence and risk factors among primary schoolchildren in the capital areas of Democratic Republic of São Tomé and Príncipe, West Africa. Parasit Vectors 5, 141. <https://doi.org/10.1186/1756-3305-5-141>

Fängström, B., Moore, S., Nermell, B., Kuenstl, L., Goessler, W., Grandér, M., Kabir, I., Palm, B., Arifeen, S.E., Vahter, M., 2008. Breast-feeding protects against arsenic exposure in Bangladeshi infants. Environ Health Perspect 116, 963–969. <https://doi.org/10.1289/ehp.11094>

Farzan, S.F., Chen, Y., Wu, F., Jiang, J., Liu, M., Baker, E., Korrick, S.A., Karagas, M.R., 2015. Blood Pressure Changes in Relation to Arsenic Exposure in a U.S. Pregnancy Cohort. Environ Health Perspect 123, 999–1006. <https://doi.org/10.1289/ehp.1408472>

Farzan, S.F., Korrick, S., Li, Z., Enelow, R., Gandolfi, A.J., Madan, J., Nadeau, K., Karagas, M.R., 2013. In utero arsenic exposure and infant infection in a United States cohort: a prospective study. Environ Res 126, 24–30. <https://doi.org/10.1016/j.envres.2013.05.001>

Fatmi, Z., Abbasi, I.N., Ahmed, M., Kazi, A., Kayama, F., 2013. Burden of skin lesions of arsenicosis at higher exposure through groundwater of taluka Gambat district Khairpur, Pakistan: a cross-sectional survey. Environ Geochem Health 35, 341–346. <https://doi.org/10.1007/s10653-012-9498-3>

Fentie, T., Erqou, S., Gedefaw, M., Desta, A., 2013. Epidemiology of human fascioliasis and intestinal parasitosis among schoolchildren in Lake Tana Basin, northwest Ethiopia. Trans R Soc Trop Med Hyg 107, 480–486. <https://doi.org/10.1093/trstmh/trt056>

Ferdosi, H., Dissen, E.K., Afari-Dwamena, N.A., Li, J., Chen, R., Feinleib, M., Lamm, S.H., 2016. Arsenic in Drinking Water and Lung Cancer Mortality in the United States: An Analysis Based on US Counties and 30 Years of Observation (1950-1979). J Environ Public Health 2016, 1602929. <https://doi.org/10.1155/2016/1602929>

Ferede, A., Lemessa, F., Tafa, M., Sisay, S., 2017. The prevalence of malnutrition and its associated risk factors among women of reproductive age in Ziway Dugda district, Arsi Zone, Oromia Regional State, Ethiopia. Public Health 152, 1–8. <https://doi.org/10.1016/j.puhe.2017.06.011>

Fernandez, M.I., López, J.F., Vivaldi, B., Coz, F., 2012. Long-term impact of arsenic in drinking water on bladder cancer health care and mortality rates 20 years after end of exposure. J Urol 187, 856–861. <https://doi.org/10.1016/j.juro.2011.10.157>

Fernando, W.B.N.T., Nanayakkara, N., Gunarathne, L., Chandrajith, R., 2020. Serum and urine fluoride levels in populations of high environmental fluoride exposure with endemic CKDu: a case-control study from Sri Lanka. Environ Geochem Health 42, 1497–1504. <https://doi.org/10.1007/s10653-019-00444-x>

Ferreira-Pêgo, C., Guelinckx, I., Moreno, L.A., Kavouras, S.A., Gandy, J., Martinez, H., Bardosono, S., Abdollahi, M., Nasseri, E., Jarosz, A., Babio, N., Salas-Salvadó, J., 2015. Total fluid intake and its determinants: cross-sectional surveys among adults in 13 countries worldwide. Eur J Nutr 54 Suppl 2, 35–43. <https://doi.org/10.1007/s00394-015-0943-9>

Fertmann, R., Hentschel, S., Dengler, D., Janssen, U., Lommel, A., 2004. Lead exposure by drinking water: an epidemiologial study in Hamburg, Germany. Int J Hyg Environ Health 207, 235–244. <https://doi.org/10.1078/1438-4639-00285>

Fillol, C., Dor, F., Clozel, B., Goria, S., Seta, N., 2010. Does arsenic in soil contribute to arsenic urinary concentrations in a French population living in a naturally arsenic contaminated area? Sci Total Environ 408, 6011–6016. <https://doi.org/10.1016/j.scitotenv.2010.08.039>

Flaten, T.P., 1992. Chlorination of drinking water and cancer incidence in Norway. Int J Epidemiol 21, 6–15. <https://doi.org/10.1093/ije/21.1.6>

Flugelman, A.A., Dubnov, J., Jacob, L., Stein, N., Habib, S., Rishpon, S., 2019. Epidemiologic Surveillance in Israel of Cryptosporidium, a Unique Waterborne Notifiable Pathogen, and Public Health Policy. Isr Med Assoc J 21, 589–594.

Folasade, I.B., 2000. Environmental Factors, Situation of Women and Child Mortality in Southwestern Nigeria. Social Science and Medicine 51, 1473–1489.

Font-Ribera, L., Cotta, J.C., Gómez-Gutiérrez, A., Villanueva, C.M., 2017. Trihalomethane concentrations in tap water as determinant of bottled water use in the city of Barcelona. J Environ Sci (China) 58, 77–82. <https://doi.org/10.1016/j.jes.2017.04.025>

Font-Ribera, L., Gràcia-Lavedan, E., Aragonés, N., Pérez-Gómez, B., Pollán, M., Amiano, P., Jiménez-Zabala, A., Castaño-Vinyals, G., Roca-Barceló, A., Ardanaz, E., Burgui, R., Molina, A.J., Fernández-Villa, T., Gómez-Acebo, I., Dierssen-Sotos, T., Moreno, V., Fernandez-Tardon, G., Peiró, R., Kogevinas, M., Villanueva, C.M., 2018. Long-term exposure to trihalomethanes in drinking water and breast cancer in the Spanish multicase-control study on cancer (MCC-SPAIN). Environ Int 112, 227–234. <https://doi.org/10.1016/j.envint.2017.12.031>

Font-Ribera, L., Kogevinas, M., Nieuwenhuijsen, M.J., Grimalt, J.O., Villanueva, C.M., 2010. Patterns of water use and exposure to trihalomethanes among children in Spain. Environ Res 110, 571–579. <https://doi.org/10.1016/j.envres.2010.05.008>

Fordyce, F.M., Johnson, C.C., Navaratna, U.R., Appleton, J.D., Dissanayake, C.B., 2000. Selenium and iodine in soil, rice and drinking water in relation to endemic goitre in Sri Lanka. Sci Total Environ 263, 127–141. <https://doi.org/10.1016/s0048-9697(00)00684-7>

Forrer, A., Khieu, V., Schär, F., Vounatsou, P., Chammartin, F., Marti, H., Muth, S., Odermatt, P., 2018. Strongyloides stercoralis and hookworm co-infection: spatial distribution and determinants in Preah Vihear Province, Cambodia. Parasit Vectors 11, 33. <https://doi.org/10.1186/s13071-017-2604-8>

Forson, A.O., Arthur, I., Olu-Taiwo, M., Glover, K.K., Pappoe-Ashong, P.J., Ayeh-Kumi, P.F., 2017. Intestinal parasitic infections and risk factors: a cross-sectional survey of some school children in a suburb in Accra, Ghana. BMC Res Notes 10, 485. <https://doi.org/10.1186/s13104-017-2802-7>

Forssén, U.M., Herring, A.H., Savitz, D.A., Nieuwenhuijsen, M.J., Murphy, P.A., Singer, P.C., Wright, J.M., 2007. Predictors of use and consumption of public drinking water among pregnant women. J Expo Sci Environ Epidemiol 17, 159–169. <https://doi.org/10.1038/sj.jes.7500488>

Forssén, U.M., Wright, J.M., Herring, A.H., Savitz, D.A., Nieuwenhuijsen, M.J., Murphy, P.A., 2009. Variability and predictors of changes in water use during pregnancy. J Expo Sci Environ Epidemiol 19, 593–602. <https://doi.org/10.1038/jes.2008.59>

Francescato, M.P., Venuto, I., Stella, A.B., Stel, G., Mallardi, F., Cauci, S., 2019. Sex differences in hydration status among adolescent elite soccer players. Journal of Human Sport and Exercise 14, 265–280. <https://doi.org/10.14198/jhse.2019.142.02>

Franken, S.C.M., Smit, C.R., Buijzen, M., 2018. Promoting Water Consumption on a Caribbean Island: An Intervention Using Children’s Social Networks at Schools. Int J Environ Res Public Health 15, 713. <https://doi.org/10.3390/ijerph15040713>

Fredrick, T., Ponnaiah, M., Murhekar, M.V., Jayaraman, Y., David, J.K., Vadivoo, S., Joshua, V., 2015. Cholera Outbreak Linked with Lack of Safe Water Supply Following a Tropical Cyclone in Pondicherry, India, 2012. Journal of Health, Population and Nutrition 33, 31.

Freedman, D.M., Cantor, K.P., Lee, N.L., Chen, L.S., Lei, H.H., Ruhl, C.E., Wang, S.S., 1997. Bladder cancer and drinking water: a population-based case-control study in Washington County, Maryland (United States). Cancer Causes Control 8, 738–744. <https://doi.org/10.1023/a:1018431421567>

Freeman, M.C., Quick, R.E., Abbott, D.P., Ogutu, P., Rheingans, R., 2009. Increasing equity of access to point-of-use water treatment products through social marketing and entrepreneurship: a case study in western Kenya. J Water Health 7, 527–534. <https://doi.org/10.2166/wh.2009.063>

Freeman, M.C., Trinies, V., Boisson, S., Mak, G., Clasen, T., 2012. Promoting household water treatment through women’s self help groups in Rural India: assessing impact on drinking water quality and equity. PLoS One 7, e44068. <https://doi.org/10.1371/journal.pone.0044068>

Freni, S.C., 1994. Exposure to high fluoride concentrations in drinking water is associated with decreased birth rates. J Toxicol Environ Health 42, 109–121. <https://doi.org/10.1080/15287399409531866>

Frost, F., Muller, T., Craun, G., Mihály, K., György, B., Calderon, R., 2005. Serological responses to Cryptosporidium antigens among women using riverbank-filtered water, conventionally filtered surface water and groundwater in Hungary. Journal of Water and Health 3, 77–82. <https://doi.org/10.2166/wh.2005.0008>

Fu, C.-J., Chuang, T.-W., Lin, H.-S., Wu, C.-H., Liu, Y.-C., Langinlur, M.K., Lu, M.-Y., Hsiao, W.W.-W., Fan, C.-K., 2014a. Seroepidemiology of Toxocara canis infection among primary schoolchildren in the capital area of the Republic of the Marshall Islands. BMC Infect Dis 14, 261. <https://doi.org/10.1186/1471-2334-14-261>

Fu, C.-J., Chuang, T.-W., Lin, H.-S., Wu, C.-H., Liu, Y.-C., Langinlur, M.K., Lu, M.-Y., Hsiao, W.W.-W., Fan, C.-K., 2014b. Toxoplasma gondii infection: seroprevalence and associated risk factors among primary school children in the capital area of the Republic of the Marshall Islands. Jpn J Infect Dis 67, 405–410. <https://doi.org/10.7883/yoken.67.405>

Fu, S., Wu, J., Li, Y., Liu, Y., Gao, Y., Yao, F., Qiu, C., Song, L., Wu, Y., Liao, Y., Sun, D., 2014. Urinary arsenic metabolism in a Western Chinese population exposed to high-dose inorganic arsenic in drinking water: Influence of ethnicity and genetic polymorphisms. Toxicology and Applied Pharmacology 274, 117–123. <https://doi.org/10.1016/j.taap.2013.11.004>

Fukushima, R., Rigolizzo, D.S., Maia, L.P., Sampaio, F.C., Lauris, J.R.P., Buzalaf, M. a. R., 2009. Environmental and individual factors associated with nail fluoride concentration. Caries Res 43, 147–154. <https://doi.org/10.1159/000211718>

Gagnon, F., Lampron-Goulet, E., Normandin, L., Langlois, M.-F., 2016. Measurements of Arsenic in the Urine and Nails of Individuals Exposed to Low Concentrations of Arsenic in Drinking Water From Private Wells in a Rural Region of Québec, Canada. J Environ Health 78, 76–83.

Galan, P., Arnaud, M.J., Czernichow, S., Delabroise, A.M., Preziosi, P., Bertrais, S., Franchisseur, C., Maurel, M., Favier, A., Hercberg, S., 2002. Contribution of mineral waters to dietary calcium and magnesium intake in a French adult population. J Am Diet Assoc 102, 1658–1662. <https://doi.org/10.1016/s0002-8223(02)90353-6>

Galgallo, D.A., Roka, Z.G., Boru, W.G., Abill, K., Ransom, J., 2018. Investigation of a typhoid fever epidemic in Moyale Sub-County, Kenya, 2014-2015. J Health Popul Nutr 37, 14. <https://doi.org/10.1186/s41043-018-0144-2>

Gallagher, L.G., Vieira, V.M., Ozonoff, D., Webster, T.F., Aschengrau, A., 2011. Risk of breast cancer following exposure to tetrachloroethylene-contaminated drinking water in Cape Cod, Massachusetts: reanalysis of a case-control study using a modified exposure assessment. Environ Health 10, 47. <https://doi.org/10.1186/1476-069X-10-47>

Gallagher, L.G., Webster, T.F., Aschengrau, A., Vieira, V.M., 2010. Using Residential History and Groundwater Modeling to Examine Drinking Water Exposure and Breast Cancer. Environ Health Perspect 118, 749–755. <https://doi.org/10.1289/ehp.0901547>

Gallaher, M.M., Herndon, J.L., Nims, L.J., Sterling, C.R., Grabowski, D.J., Hull, H.F., 1989. Cryptosporidiosis and surface water. Am J Public Health 79, 39–42.

Galletti, M., D’Annibale, L., Pinto, V., Cremisini, C., 2003. Uranium daily intake and urinary excretion: a preliminary study in Italy. Health Phys 85, 228–235. <https://doi.org/10.1097/00004032-200308000-00013>

Gamble, M.V., Liu, X., Ahsan, H., Pilsner, R., Ilievski, V., Slavkovich, V., Parvez, F., Levy, D., Factor-Litvak, P., Graziano, J.H., 2005. Folate, homocysteine, and arsenic metabolism in arsenic-exposed individuals in Bangladesh. Environ Health Perspect 113, 1683–1688. <https://doi.org/10.1289/ehp.8084>

Gao, S., Lin, P.-I., Mostofa, G., Quamruzzaman, Q., Rahman, M., Rahman, M.L., Su, L., Hsueh, Y., Weisskopf, M., Coull, B., Christiani, D.C., 2019. Determinants of arsenic methylation efficiency and urinary arsenic level in pregnant women in Bangladesh. Environmental Health 18, 94. <https://doi.org/10.1186/s12940-019-0530-2>

Gardner, R., Hamadani, J., Grandér, M., Tofail, F., Nermell, B., Palm, B., Kippler, M., Vahter, M., 2011. Persistent Exposure to Arsenic via Drinking Water in Rural Bangladesh Despite Major Mitigation Efforts. Am J Public Health 101, S333–S338. <https://doi.org/10.2105/AJPH.2010.300025>

Gardner, R.M., Nermell, B., Kippler, M., Grandér, M., Li, L., Ekström, E.-C., Rahman, A., Lönnerdal, B., Hoque, A.M.W., Vahter, M., 2011. Arsenic methylation efficiency increases during the first trimester of pregnancy independent of folate status. Reprod Toxicol 31, 210–218. <https://doi.org/10.1016/j.reprotox.2010.11.002>

Gasem, M.H., Dolmans, W.M., Keuter, M.M., Djokomoeljanto, R.R., 2001. Poor food hygiene and housing as risk factors for typhoid fever in Semarang, Indonesia. Trop Med Int Health 6, 484–490. <https://doi.org/10.1046/j.1365-3156.2001.00734.x>

Gatseva, P.D., Argirova, M.D., 2008. High-nitrate levels in drinking water may be a risk factor for thyroid dysfunction in children and pregnant women living in rural Bulgarian areas. Int J Hyg Environ Health 211, 555–559. <https://doi.org/10.1016/j.ijheh.2007.10.002>

Gautam, S., Min, H., Kim, H., Jeong, H.-S., 2019. Determining factors for the prevalence of anemia in women of reproductive age in Nepal: Evidence from recent national survey data. PLOS ONE 14, e0218288. <https://doi.org/10.1371/journal.pone.0218288>

Gbadebo, A.M., Oyesanya, T.M., 2005. Assessment of iodine deficiency and goitre incidence in parts of Yewa Area of Ogun State, Southwestern Nigeria. Environ Geochem Health 27, 491–499. <https://doi.org/10.1007/s10653-005-6982-z>

Ge, P., Zhang, Z., Wang, Y., Cao, Y., Xi, J., 2013. Iodine status in vulnerable groups Hinxia Hui autonomous prefecture, China 18, 68–71.

Gebreegziabher, T., Regassa, N., 2019. Ethiopia’s high childhood undernutrition explained: analysis of the prevalence and key correlates based on recent nationally representative data. Public Health Nutr 22, 2099–2109. <https://doi.org/10.1017/S1368980019000569>

Geere, J.-A., Bartram, J., Bates, L., Danquah, L., Evans, B., Fisher, M.B., Groce, N., Majuru, B., Mokoena, M.M., Mukhola, M.S., Nguyen-Viet, H., Duc, P.P., Williams, A.R., Schmidt, W.-P., Hunter, P.R., 2018. Carrying water may be a major contributor to disability from musculoskeletal disorders in low income countries: a cross-sectional survey in South Africa, Ghana and Vietnam. J Glob Health 8, 010406. <https://doi.org/10.7189/jogh.08.010406>

Geere, J.L., Mokoena, M.M., Jagals, P., Poland, F., Hartley, S., 2010. How do children perceive health to be affected by domestic water carrying? Qualitative findings from a mixed methods study in rural South Africa. Child: Care, Health and Development 36, 818–826. <https://doi.org/10.1111/j.1365-2214.2010.01098.x>

Gelmann, E.R., Gurzau, E., Gurzau, A., Goessler, W., Kunrath, J., Yeckel, C.W., McCarty, K.M., 2013. A pilot study: the importance of inter-individual differences in inorganic arsenic metabolism for birth weight outcome. Environ Toxicol Pharmacol 36, 1266–1275. <https://doi.org/10.1016/j.etap.2013.10.006>

Genius, M., Hatzaki, E., Kouromichelaki, E.M., Kouvakis, G., Nikiforaki, S., Tsagarakis, K.P., 2008. Evaluating Consumers’ Willingness to Pay for Improved Potable Water Quality and Quantity. Water Resour Manage 22, 1825–1834. <https://doi.org/10.1007/s11269-008-9255-7>

George, J., Suriyanarayanan, S., 2016. Seasonal patterns of drinking water consumption pattern in Mysore City, Karnataka, South India. Journal of Water, Sanitation and Hygiene for Development 6, 569–575. <https://doi.org/10.2166/washdev.2016.082>

Getz, K.D., Janulewicz, P.A., Rowe, S., Weinberg, J.M., Winter, M.R., Martin, B.R., Vieira, V.M., White, R.F., Aschengrau, A., 2012. Prenatal and early childhood exposure to tetrachloroethylene and adult vision. Environ Health Perspect 120, 1327–1332. <https://doi.org/10.1289/ehp.1103996>

Ghaderpoori, M., Paydar, M., Zarei, A., Alidadi, H., Najafpoor, A.A., Gohary, A.H., Shams, M., 2019. Health risk assessment of fluoride in water distribution network of Mashhad, Iran. Human and Ecological Risk Assessment: An International Journal 25, 851–862. <https://doi.org/10.1080/10807039.2018.1453297>

Ghafuri, Y., Yunesian, M., Nabizadeh, R., Mesdaghinia, A., Dehghani, M.H., Alimohammadi, M., 2018. Platinum cytotoxic drugs in the municipal wastewater and drinking water, a validation method and health risk assessment. Human and Ecological Risk Assessment: An International Journal 24, 784–796. <https://doi.org/10.1080/10807039.2017.1400372>

Ghosh, U., Bose, S., Bramhachari, R., Mandal, S., 2016. Expressing collective voices on children’s health: photovoice exploration with mothers of young children from the Indian Sundarbans. BMC Health Services Research 16, 625. <https://doi.org/10.1186/s12913-016-1866-8>

Gibb, H., Haver, C., Kozlov, K., Centeno, J.A., Jurgenson, V., Kolker, A., Conko, K.M., Landa, E.R., Xu, H., 2011. Biomarkers of mercury exposure in two eastern Ukraine cities. J Occup Environ Hyg 8, 187–193. <https://doi.org/10.1080/15459624.2011.556984>

Gilbert-Diamond, D., Emond, J.A., Baker, E.R., Korrick, S.A., Karagas, M.R., 2016. Relation between in Utero Arsenic Exposure and Birth Outcomes in a Cohort of Mothers and Their Newborns from New Hampshire. Environ Health Perspect 124, 1299–1307. <https://doi.org/10.1289/ehp.1510065>

Gillette-Guyonnet, S., Andrieu, S., Nourhashemi, F., de La Guéronnière, V., Grandjean, H., Vellas, B., 2005. Cognitive impairment and composition of drinking water in women: findings of the EPIDOS Study. Am J Clin Nutr 81, 897–902. <https://doi.org/10.1093/ajcn/81.4.897>

Ginsberg, G.L., Hattis, D.B., Zoeller, R.T., Rice, D.C., 2007. Evaluation of the U.S. EPA/OSWER Preliminary Remediation Goal for Perchlorate in Groundwater: Focus on Exposure to Nursing Infants. Environmental Health Perspectives 115, 361–369. <https://doi.org/10.1289/ehp.9533>

Giri, S., Singh, A.K., Mahato, M.K., 2020. Monte Carlo simulation-based probabilistic health risk assessment of metals in groundwater via ingestion pathway in the mining areas of Singhbhum copper belt, India. Int J Environ Health Res 30, 447–460. <https://doi.org/10.1080/09603123.2019.1599101>

Godebo, T., Paul, C., Jeuland, M., Tekle-Haimanot, R., 2019. Biomonitoring of metals and trace elements in urine of central Ethiopian populations. International Journal of Hygiene and Environmental Health 222. <https://doi.org/10.1016/j.ijheh.2018.12.007>

Gold, E.B., Blount, B.C., O’Neill Rasor, M., Lee, J.S., Alwis, U., Srivastav, A., Kim, K., 2013. Thyroid hormones and thyroid disease in relation to perchlorate dose and residence near a superfund site. J Expo Sci Environ Epidemiol 23, 399–408. <https://doi.org/10.1038/jes.2012.90>

Gong, G., Basom, J., Mattevada, S., Onger, F., 2015. Association of hypothyroidism with low-level arsenic exposure in rural West Texas. Environ Res 138, 154–160. <https://doi.org/10.1016/j.envres.2015.02.001>

Gong, G., O’Bryant, S.E., 2012. Low-level arsenic exposure, AS3MT gene polymorphism and cardiovascular diseases in rural Texas counties. Environ Res 113, 52–57. <https://doi.org/10.1016/j.envres.2012.01.003>

Gonsebatt, M.E., Vega, L., Montero, R., Garcia-Vargas, G., Del Razo, L.M., Albores, A., Cebrian, M.E., Ostrosky-Wegman, P., 1994. Lymphocyte replicating ability in individuals exposed to arsenic via drinking water. Mutat Res 313, 293–299. <https://doi.org/10.1016/0165-1161(94)90059-0>

Gonsebatt, M.E., Vega, L., Salazar, A.M., Montero, R., Guzmán, P., Blas, J., Del Razo, L.M., García-Vargas, G., Albores, A., Cebrián, M.E., Kelsh, M., Ostrosky-Wegman, P., 1997. Cytogenetic effects in human exposure to arsenic. Mutat Res 386, 219–228. <https://doi.org/10.1016/s1383-5742(97)00009-4>

González-Weller, D., Gutiérrez, Á.J., Rubio, C., Revert, C., Hardisson, A., 2012. A total diet study of nickel intake in a Spanish population (Canary Islands). Int J Food Sci Nutr 63, 902–912. <https://doi.org/10.3109/09637486.2012.687363>

Goodman, K.J., 1994. Helicobacter pylori infection in the Colombian Andes: A population-based study of transmission pathways (Ph.D.). ProQuest Dissertations and Theses. University of California, Los Angeles, Ann Arbor.

Goswami, S., Jha, A., Sivan, S.P., Dambhare, D., Gupta, S.S., 2019. Outbreak investigation of cholera outbreak in a slum area of urban Wardha, India: An interventional epidemiological study. J Family Med Prim Care 8, 1112–1116. <https://doi.org/10.4103/jfmpc.jfmpc_308_18>

Gottlieb, M.S., Carr, J.K., Morris, D.T., 1981. Cancer and drinking water in Louisiana: colon and rectum. Int J Epidemiol 10, 117–125. <https://doi.org/10.1093/ije/10.2.117>

Gouda, J., Gupta, A.K., Yadav, A.K., 2015. Association of child health and household amenities in high focus states in India: a district-level analysis. BMJ Open 5, e007589. <https://doi.org/10.1136/bmjopen-2015-007589>

Grady, C.A., Van Nguyen, K., Van Nguyen, T., Blatchley, E.R., III, 2018. Who is being left behind? An analysis of improved drinking water and basic sanitation access in the Vietnamese Mekong Delta. Journal of Water, Sanitation and Hygiene for Development 8, 508–519. <https://doi.org/10.2166/washdev.2018.005>

Graham, J.P., Hirai, M., Kim, S.-S., 2016. An Analysis of Water Collection Labor among Women and Children in 24 Sub-Saharan African Countries. PLoS One 11, e0155981. <https://doi.org/10.1371/journal.pone.0155981>

Graham, M.J., Larsen, U., Xu, X., 1999. Secular trend in age at menarche in China: a case study of two rural counties in Anhui Province. J Biosoc Sci 31, 257–267. <https://doi.org/10.1017/s0021932099002576>

Grazuleviciene, R., Kapustinskiene, V., Vencloviene, J., Buinauskiene, J., Nieuwenhuijsen, M.J., 2013. Risk of congenital anomalies in relation to the uptake of trihalomethane from drinking water during pregnancy. Occup Environ Med 70, 274–282. <https://doi.org/10.1136/oemed-2012-101093>

Grazuleviciene, R., Nadisauskiene, R., Buinauskiene, J., Grazulevicius, T., 2009. Effects of Elevated Levels of Manganese and Iron in Drinking Water on Birth Outcomes. Pol. J. Environ. Stud. 18, 819–825.

Grazuleviciene, R., Nieuwenhuijsen, M.J., Vencloviene, J., Kostopoulou-Karadanelli, M., Krasner, S.W., Danileviciute, A., Balcius, G., Kapustinskiene, V., 2011. Individual exposures to drinking water trihalomethanes, low birth weight and small for gestational age risk: a prospective Kaunas cohort study. Environ Health 10, 32. <https://doi.org/10.1186/1476-069X-10-32>

Green, R., Lanphear, B., Hornung, R., Flora, D., Martinez-Mier, E.A., Neufeld, R., Ayotte, P., Muckle, G., Till, C., 2019. Association Between Maternal Fluoride Exposure During Pregnancy and IQ Scores in Offspring in Canada. JAMA Pediatrics 173, 940–948. <https://doi.org/10.1001/jamapediatrics.2019.1729>

Greenlee, A.R., Arbuckle, T.E., Chyou, P.-H., 2003. Risk factors for female infertility in an agricultural region. Epidemiology 14, 429–436. <https://doi.org/10.1097/01.EDE.0000071407.15670.aa>

Griffith, J., Duncan, R.C., Riggan, W.B., Pellom, A.C., 1989. Cancer mortality in U.S. counties with hazardous waste sites and ground water pollution. Arch Environ Health 44, 69–74. <https://doi.org/10.1080/00039896.1989.9934378>

Gross, E., Elshiewy, O., 2019. Choice and quantity demand for improved and unimproved public water sources in rural areas: Evidence from Benin. Journal of rural studies.

Grossman, D.S., Slusky, D.J.G., 2019. The Impact of the Flint Water Crisis on Fertility. Demography 56, 2005–2031. <https://doi.org/10.1007/s13524-019-00831-0>

Guha Mazumder, D.N., Haque, R., Ghosh, N., De, B.K., Santra, A., Chakraborty, D., Smith, A.H., 1998. Arsenic levels in drinking water and the prevalence of skin lesions in West Bengal, India. Int J Epidemiol 27, 871–877. <https://doi.org/10.1093/ije/27.5.871>

Guidotti, T.L., Calhoun, T., Davies-Cole, J.O., Knuckles, M.E., Stokes, L., Glymph, C., Lum, G., Moses, M.S., Goldsmith, D.F., Ragain, L., 2007. Elevated Lead in Drinking Water in Washington, DC, 2003–2004: The Public Health Response. Environ Health Perspect 115, 695–701. <https://doi.org/10.1289/ehp.8722>

Gulis, G., Czompolyova, M., Cerhan, J.R., 2002. An ecologic study of nitrate in municipal drinking water and cancer incidence in Trnava District, Slovakia. Environ Res 88, 182–187. <https://doi.org/10.1006/enrs.2002.4331>

Gulliford, M.C., Mahabir, D., 1998. Social inequalities in morbidity from diabetes mellitus in public primary care clinics in Trinidad and Tobago. Soc Sci Med 46, 137–144. <https://doi.org/10.1016/s0277-9536(97)00155-x>

Guo, H., 1998. Using Ecological Data to Estimate a Regression Model for Individual Data: The Association between Arsenic in Drinking Water and Incidence of Skin Cancer. Environmental Research 79, 82–93. <https://doi.org/10.1006/enrs.1998.3863>

Guo, H.-R., 2011. Age adjustment in ecological studies: using a study on arsenic ingestion and bladder cancer as an example. BMC Public Health 11, 820. <https://doi.org/10.1186/1471-2458-11-820>

Guo, H.-R., 2004. Arsenic level in drinking water and mortality of lung cancer (Taiwan). Cancer Causes Control 15, 171–177. <https://doi.org/10.1023/B:CACO.0000019503.02851.b0>

Guo, H.-R., 2003. The lack of a specific association between arsenic in drinking water and hepatocellular carcinoma. Journal of Hepatology 39, 383–388. <https://doi.org/10.1016/S0168-8278(03)00297-6>

Guo, H.R., Chiang, H.S., Hu, H., Lipsitz, S.R., Monson, R.R., 1997. Arsenic in drinking water and incidence of urinary cancers. Epidemiology 8, 545–550. <https://doi.org/10.1097/00001648-199709000-00012>

Guo, H.-R., Wang, N.-S., Hu, H., Monson, R.R., 2004. Cell type specificity of lung cancer associated with arsenic ingestion. Cancer Epidemiol Biomarkers Prev 13, 638–643.

Guo, H.R., Yu, H.S., Hu, H., Monson, R.R., 2001. Arsenic in drinking water and skin cancers: cell-type specificity (Taiwan, ROC). Cancer Causes Control 12, 909–916. <https://doi.org/10.1023/a:1013712203455>

Gupta, M., Bashar, M., Mahajan, S., Murugan, S., Kankaria, A., Bhag, C., 2019. Epidemiological investigation of an acute viral hepatitis outbreak in an urbanized rural area in a North Indian Union Territory. International Journal of Infectious Diseases 79, 130. <https://doi.org/10.1016/j.ijid.2018.11.317>

Gupta, R., Sanjeev, R.K., Agarwal, A., Tomar, R.P.S., Kumar, N., Dutt, V., Gupta, S., 2019. A study of hepatitis A virus seropositivity among children aged between 1 and 5 years of age: Implications for universal immunization. Med J Armed Forces India 75, 335–338. <https://doi.org/10.1016/j.mjafi.2018.11.007>

Gupta, V., Garg, R.K., Pant, K.K., Khattri, S., 2014. A study on risk factors for Parkinson׳s disease in Indian population. Bioinformation 10, 342–346. <https://doi.org/10.6026/97320630010342>

Gurley, E.S., Hossain, M.J., Paul, R.C., Sazzad, H.M.S., Islam, M.S., Parveen, S., Faruque, L.I., Husain, M., Ara, K., Jahan, Y., Rahman, M., Luby, S.P., 2014. Outbreak of hepatitis E in urban Bangladesh resulting in maternal and perinatal mortality. Clin Infect Dis 59, 658–665. <https://doi.org/10.1093/cid/ciu383>

Guthmann, J.-P., Klovstad, H., Boccia, D., Hamid, N., Pinoges, L., Nizou, J.-Y., Tatay, M., Diaz, F., Moren, A., Grais, R.F., Ciglenecki, I., Nicand, E., Guerin, P.J., 2006. A large outbreak of hepatitis E among a displaced population in Darfur, Sudan, 2004: the role of water treatment methods. Clin Infect Dis 42, 1685–1691. <https://doi.org/10.1086/504321>

Gyang, V.P., Chuang, T.-W., Liao, C.-W., Lee, Y.-L., Akinwale, O.P., Orok, A., Ajibaye, O., Babasola, A.J., Cheng, P.-C., Chou, C.-M., Huang, Y.-C., Sonko, P., Fan, C.-K., 2019. Intestinal parasitic infections: Current status and associated risk factors among school aged children in an archetypal African urban slum in Nigeria. J Microbiol Immunol Infect 52, 106–113. <https://doi.org/10.1016/j.jmii.2016.09.005>

Gyasi, S.F., Boamah, B., Awuah, E., Otabil, K.B., 2018. A Perspective Analysis of Dams and Water Quality: The Bui Power Project on the Black Volta, Ghana. Journal of Environmental and Public Health 2018, e6471525. <https://doi.org/10.1155/2018/6471525>

Gyllenhammar, I., Berger, U., Sundström, M., McCleaf, P., Eurén, K., Eriksson, S., Ahlgren, S., Lignell, S., Aune, M., Kotova, N., Glynn, A., 2015. Influence of contaminated drinking water on perfluoroalkyl acid levels in human serum--A case study from Uppsala, Sweden. Environ Res 140, 673–683. <https://doi.org/10.1016/j.envres.2015.05.019>

Habi, M., Harrouz, O., 2015. Domestic water conservation practices in Tlemcen City (Algeria). Appl Water Sci 5, 161–169. <https://doi.org/10.1007/s13201-014-0175-9>

Habib, R.R., Elzein, K., Hojeij, S., 2013. The association between women’s self-rated health and satisfaction with environmental services in an underserved community in Lebanon. Women Health 53, 451–467. <https://doi.org/10.1080/03630242.2013.806387>

Habyarimana, F., Zewotir, T., Ramroop, S., 2018. Spatial Distribution and Analysis of Risk Factors Associated with Anemia Among Women of Reproductive Age: Case of 2014 Rwanda Demographic and Health Survey Data. The Open Public Health Journal 11. <https://doi.org/10.2174/1874944501811010425>

Hadi, A., Parveen, R., 2004. Arsenicosis in Bangladesh: prevalence and socio-economic correlates. Public Health 118, 559–564. <https://doi.org/10.1016/j.puhe.2003.11.002>

Hafeman, D., Factor-Litvak, P., Cheng, Z., van Geen, A., Ahsan, H., 2007. Association between manganese exposure through drinking water and infant mortality in Bangladesh. Environ Health Perspect 115, 1107–1112. <https://doi.org/10.1289/ehp.10051>

Haghighatdoost, F., Feizi, A., Esmaillzadeh, A., Rashidi-Pourfard, N., Keshteli, A.H., Roohafza, H., Adibi, P., 2018. Drinking plain water is associated with decreased risk of depression and anxiety in adults: Results from a large cross-sectional study. World J Psychiatry 8, 88–96. <https://doi.org/10.5498/wjp.v8.i3.88>

Hajissa, K., Muhajir, A.E.M.A., Eshag, H.A., Alfadel, A., Nahied, E., Dahab, R., Ali, S.M., Mohammed, M., Gaafar, M., Mohamed, Z., 2018. Prevalence of schistosomiasis and associated risk factors among school children in Um-Asher Area, Khartoum, Sudan. BMC Res Notes 11, 779. <https://doi.org/10.1186/s13104-018-3871-y>

Halder, G.E., Bearman, G., Sanogo, K., Stevens, M.P., 2013. Water sanitation, access, use and self-reported diarrheal disease in rural Honduras. Rural Remote Health 13, 2413.

Hall, M., Chen, Y., Ahsan, H., Slavkovich, V., van Geen, A., Parvez, F., Graziano, J., 2006. Blood arsenic as a biomarker of arsenic exposure: results from a prospective study. Toxicology 225, 225–233. <https://doi.org/10.1016/j.tox.2006.06.010>

Hallenbeck, W.H., Brenniman, G.R., Anderson, R.J., 1981. High sodium in drinking water and its effect on blood pressure. Am J Epidemiol 114, 817–826. <https://doi.org/10.1093/oxfordjournals.aje.a113252>

Hamadani, J.D., Tofail, F., Nermell, B., Gardner, R., Shiraji, S., Bottai, M., Arifeen, S.E., Huda, S.N., Vahter, M., 2011. Critical windows of exposure for arsenic-associated impairment of cognitive function in pre-school girls and boys: a population-based cohort study. Int J Epidemiol 40, 1593–1604. <https://doi.org/10.1093/ije/dyr176>

Han, K., Shin, D.-W., Lee, T.-Y., Lee, Y.-H., 2008. Seroprevalence of Toxoplasma gondii infection and risk factors associated with seropositivity of pregnant women in Korea. J Parasitol 94, 963–965. <https://doi.org/10.1645/GE-1435.1>

Han, Y.-Y., Weissfeld, J.L., Davis, D.L., Talbott, E.O., 2009. Arsenic levels in ground water and cancer incidence in Idaho: an ecologic study. Int Arch Occup Environ Health 82, 843–849. <https://doi.org/10.1007/s00420-008-0362-9>

Hanafi, M.I., Mohamed, A.M., 2013. Helicobacter pylori infection: seroprevalence and predictors among healthy individuals in Al Madinah, Saudi Arabia. J Egypt Public Health Assoc 88, 40–45. <https://doi.org/10.1097/01.EPX.0000427043.99834.a4>

Hancock, M.L., Bichet, D.G., Eckert, G.J., Bankir, L., Wagner, M.A., Pratt, J.H., 2010. Race, sex, and the regulation of urine osmolality: observations made during water deprivation. Am J Physiol Regul Integr Comp Physiol 299, R977-980. <https://doi.org/10.1152/ajpregu.00289.2010>

Handayani, T., Mujiani, null, Hull, V., Rohde, J.E., 1983. Child mortality in a rural Javanese village: a prospective study. Int J Epidemiol 12, 88–92. <https://doi.org/10.1093/ije/12.1.88>

Hanh, H.T., Kim, K.-W., Bang, S., Hoa, N.M., 2011. Community exposure to arsenic in the Mekong river delta, Southern Vietnam. J Environ Monit 13, 2025–2032. <https://doi.org/10.1039/c1em10037h>

Hanson, S.A., France, C.R., 2004. Predonation water ingestion attenuates negative reactions to blood donation. Transfusion 44, 924–928. <https://doi.org/10.1111/j.1537-2995.2004.03426.x>

Haque, M.A., Platts-Mills, J.A., Mduma, E., Bodhidatta, L., Bessong, P., Shakoor, S., Kang, G., Kosek, M.N., Lima, A.A.M., Shrestha, S.K., Alam, M.A., Havt, A., Samie, A., Guerrant, R.L., Lang, D., Mahfuz, M., Bhutta, Z.A., Houpt, E.R., Ahmed, T., 2019. Determinants of Campylobacter infection and association with growth and enteric inflammation in children under 2 years of age in low-resource settings. Sci Rep 9, 17124. <https://doi.org/10.1038/s41598-019-53533-3>

Harari, F., Åkesson, A., Casimiro, E., Lu, Y., Vahter, M., 2016. Exposure to lithium through drinking water and calcium homeostasis during pregnancy: A longitudinal study. Environ Res 147, 1–7. <https://doi.org/10.1016/j.envres.2016.01.031>

Harris, A.R., Pickering, A.J., Boehm, A.B., Mrisho, M., Davis, J., 2019. Comparison of analytical techniques to explain variability in stored drinking water quality and microbial hand contamination of female caregivers in Tanzania. Environ. Sci.: Processes Impacts 21, 893–903. <https://doi.org/10.1039/C8EM00460A>

Harris, L.M., 2008. Water Rich, Resource Poor: Intersections of Gender, Poverty, and Vulnerability in Newly Irrigated Areas of Southeastern Turkey. World Development, Special Section: Social Movements and the Dynamics of Rural Development in Latin America (pp. 2874-2952) 36, 2643–2662. <https://doi.org/10.1016/j.worlddev.2008.03.004>

Hasan, G., Assiri, A., Marzuuk, N., Daef, E., Abdelwahab, S., Ahmed, A., Mohamad, I., Al-Eyadhy, A., Alhaboob, A., Temsah, M.-H., 2016. Incidence and characteristics of hepatitis E virus infection in children in Assiut, Upper Egypt. J Int Med Res 44, 1115–1122. <https://doi.org/10.1177/0300060516659575>

Hasan, M.M., Richardson, A., 2017. How sustainable household environment and knowledge of healthy practices relate to childhood morbidity in South Asia: analysis of survey data from Bangladesh, Nepal and Pakistan. BMJ Open 7, e015019. <https://doi.org/10.1136/bmjopen-2016-015019>

Haug, L.S., Salihovic, S., Jogsten, I.E., Thomsen, C., van Bavel, B., Lindström, G., Becher, G., 2010. Levels in food and beverages and daily intake of perfluorinated compounds in Norway. Chemosphere 80, 1137–1143. <https://doi.org/10.1016/j.chemosphere.2010.06.023>

He, S., Wu, J., 2019. Hydrogeochemical Characteristics, Groundwater Quality, and Health Risks from Hexavalent Chromium and Nitrate in Groundwater of Huanhe Formation in Wuqi County, Northwest China. Expo Health 11, 125–137. <https://doi.org/10.1007/s12403-018-0289-7>

He, T., Ohgami, N., Li, X., Yajima, I., Negishi-Oshino, R., Kato, Y., Ohgami, K., Xu, H., Ahsan, N., Akhand, A.A., Kato, M., 2019. Hearing loss in humans drinking tube well water with high levels of iron in arsenic–polluted area. Sci Rep 9, 9028. <https://doi.org/10.1038/s41598-019-45524-1>

He, W., Qin, N., Kong, X., Liu, W., He, Q., Ouyang, H., Yang, C., Jiang, Y., Wang, Q., Yang, B., Xu, F., 2013. Spatio-temporal distributions and the ecological and health risks of phthalate esters (PAEs) in the surface water of a large, shallow Chinese lake. Science of The Total Environment 461–462, 672–680. <https://doi.org/10.1016/j.scitotenv.2013.05.049>

He, X., Karagas, M.R., Murray, C., 2018. Impact of receipt of private well arsenic test results on maternal use of contaminated drinking water in a U.S. population. Sci Total Environ 643, 1005–1012. <https://doi.org/10.1016/j.scitotenv.2018.06.228>

Heck, J.E., Chen, Y., Grann, V.R., Slavkovich, V., Parvez, F., Ahsan, H., 2008. Arsenic exposure and anemia in Bangladesh: a population-based study. J Occup Environ Med 50, 80–87. <https://doi.org/10.1097/JOM.0b013e31815ae9d4>

Heikens, A., Widianarko, B., Dewi, I.C., de Boer, J.L.M., Seinen, W., van Leeuwen, K., 2005. The Impact of the Hyperacid Ijen Crater Lake. Part II: A Total Diet Study. Environ Geochem Health 27, 475–483. <https://doi.org/10.1007/s10653-005-5229-3>

Helbich, M., Leitner, M., Kapusta, N.D., 2015. Lithium in drinking water and suicide mortality: interplay with lithium prescriptions. Br J Psychiatry 207, 64–71. <https://doi.org/10.1192/bjp.bp.114.152991>

Henjum, S., Barikmo, I., Strand, T.A., Oshaug, A., Torheim, L.E., 2012. Iodine-induced goitre and high prevalence of anaemia among Saharawi refugee women. Public Health Nutr 15, 1512–1518. <https://doi.org/10.1017/S1368980011002886>

Hernandez-Cortazar, I., Karla Y., A.-V., Guzmán-Marín, E., Segura-Correa, J., Ortega, A., Carrillo-Martínez, J., Gonzalez Losa, M., Conde-Ferraez, L., Jimenez-coello, M., 2016. Toxoplasma gondii in women with recent abortion from Southern Mexico. Asian Pacific Journal of Tropical Disease 6, 193–198. <https://doi.org/10.1016/S2222-1808(15)61012-X>

Hertz-Picciotto, I., Swan, S.H., Neutra, R.R., Samuels, S.J., 1989. Spontaneous abortions in relation to consumption of tap water: an application of methods from survival analysis to a pregnancy follow-up study. Am J Epidemiol 130, 79–93. <https://doi.org/10.1093/oxfordjournals.aje.a115325>

Hestvik, E., Tylleskar, T., Kaddu-Mulindwa, D.H., Ndeezi, G., Grahnquist, L., Olafsdottir, E., Tumwine, J.K., 2010. Helicobacter pylori in apparently healthy children aged 0-12 years in urban Kampala, Uganda: a community-based cross sectional survey. BMC Gastroenterol 10, 62. <https://doi.org/10.1186/1471-230X-10-62>

Hestvik, E., Tylleskar, T., Ndeezi, G., Grahnquist, L., Olafsdottir, E., Tumwine, J.K., Kaddu-Mulindwa, D.H., 2011. Prevalence of Helicobacter pylori in HIV-infected, HAART-naïve Ugandan children: a hospital-based survey. J Int AIDS Soc 14, 34. <https://doi.org/10.1186/1758-2652-14-34>

Heukelbach, J., Meyer-Cirkel, V., Moura, R.C.S., Gomide, M., Queiroz, J.A.N., Saweljew, P., Liesenfeld, O., 2007. Waterborne toxoplasmosis, northeastern Brazil. Emerg Infect Dis 13, 287–289. <https://doi.org/10.3201/eid1302.060686>

Hillier, S., Cooper, C., Kellingray, S., Russell, G., Hughes, H., Coggon, D., 2000. Fluoride in drinking water and risk of hip fracture in the UK: a case-control study. Lancet 355, 265–269. <https://doi.org/10.1016/S0140-6736(99)07161-5>

Hinwood, A.L., Callan, A.C., Ramalingam, M., Boyce, M., Heyworth, J., McCafferty, P., Odland, J.Ø., 2013. Cadmium, lead and mercury exposure in non smoking pregnant women. Environ Res 126, 118–124. <https://doi.org/10.1016/j.envres.2013.07.005>

Hinwood, A.L., Jolley, D.J., Sim, M.R., 1999. Cancer incidence and high environmental arsenic concentrations in rural populations: Results of an ecological study. International Journal of Environmental Health Research 9, 131–141. <https://doi.org/10.1080/09603129973272>

Hiremath, B.R., J, S.K., 2015. Diarrhoeal disease outbreak in a rural area of Karnataka. Indian Journal of Community Health 27, 462–466.

Ho, C.-K., Yang, Y.-H., Yang, C.-Y., 2011. Nitrates in drinking water and the risk of death from brain cancer: does hardness in drinking water matter? J Toxicol Environ Health A 74, 747–756. <https://doi.org/10.1080/15287394.2011.539143>

Hoen, A.G., Madan, J.C., Li, Z., Coker, M., Lundgren, S.N., Morrison, H.G., Palys, T., Jackson, B.P., Sogin, M.L., Cottingham, K.L., Karagas, M.R., 2018. Sex-specific associations of infants’ gut microbiome with arsenic exposure in a US population. Sci Rep 8, 12627. <https://doi.org/10.1038/s41598-018-30581-9>

Hoffman, C.S., Mendola, P., Savitz, D.A., Herring, A.H., Loomis, D., Hartmann, K.E., Singer, P.C., Weinberg, H.S., Olshan, A.F., 2008. Drinking water disinfection by-product exposure and fetal growth. Epidemiology 19, 729–737. <https://doi.org/10.1097/EDE.0b013e3181812bd4>

Hofman, A., Valkenburg, H.A., Vaandrager, G.J., 1980. Increased blood pressure in schoolchildren related to high sodium levels in drinking water. J Epidemiol Community Health 34, 179–181. <https://doi.org/10.1136/jech.34.3.179>

Hölzer, J., Midasch, O., Rauchfuss, K., Kraft, M., Reupert, R., Angerer, J., Kleeschulte, P., Marschall, N., Wilhelm, M., 2008. Biomonitoring of Perfluorinated Compounds in Children and Adults Exposed to Perfluorooctanoate-Contaminated Drinking Water. Environ Health Perspect 116, 651–657. <https://doi.org/10.1289/ehp.11064>

Honda, R., Swaddiwudhipong, W., Nishijo, M., Mahasakpan, P., Teeyakasem, W., Ruangyuttikarn, W., Satarug, S., Padungtod, C., Nakagawa, H., 2010. Cadmium induced renal dysfunction among residents of rice farming area downstream from a zinc-mineralized belt in Thailand. Toxicol Lett 198, 26–32. <https://doi.org/10.1016/j.toxlet.2010.04.023>

Hong, Rathavuth, Hong, Rathmony, 2007. Economic inequality and undernutrition in women: multilevel analysis of individual, household, and community levels in Cambodia. Food Nutr Bull 28, 59–66. <https://doi.org/10.1177/156482650702800107>

Hooton, T.M., Vecchio, M., Iroz, A., Tack, I., Dornic, Q., Seksek, I., Lotan, Y., 2018. Effect of Increased Daily Water Intake in Premenopausal Women With Recurrent Urinary Tract Infections: A Randomized Clinical Trial. JAMA Internal Medicine 178, 1509–1515. <https://doi.org/10.1001/jamainternmed.2018.4204>

Hopenhayn, C., Bush, H.M., Bingcang, A., Hertz-Picciotto, I., 2006. Association between arsenic exposure from drinking water and anemia during pregnancy. J Occup Environ Med 48, 635–643. <https://doi.org/10.1097/01.jom.0000205457.44750.9f>

Hopenhayn, C., Huang, B., Christian, J., Peralta, C., Ferreccio, C., Atallah, R., Kalman, D., 2003. Profile of urinary arsenic metabolites during pregnancy. Environ Health Perspect 111, 1888–1891. <https://doi.org/10.1289/ehp.6254>

Hopenhayn-Rich, C., Biggs, M.L., Fuchs, A., Bergoglio, R., Tello, E.E., Nicolli, H., Smith, A.H., 1996a. Bladder cancer mortality associated with arsenic in drinking water in Argentina. Epidemiology 7, 117–124. <https://doi.org/10.1097/00001648-199603000-00003>

Hopenhayn-Rich, C., Biggs, M.L., Kalman, D.A., Moore, L.E., Smith, A.H., 1996b. Arsenic methylation patterns before and after changing from high to lower concentrations of arsenic in drinking water. Environ Health Perspect 104, 1200–1207. <https://doi.org/10.1289/ehp.961041200>

Hopenhayn-Rich, C., Biggs, M.L., Smith, A.H., 1998. Lung and kidney cancer mortality associated with arsenic in drinking water in Córdoba, Argentina. Int J Epidemiol 27, 561–569. <https://doi.org/10.1093/ije/27.4.561>

Hopenhayn-Rich, C., Biggs, M.L., Smith, A.H., Kalman, D.A., Moore, L.E., 1996c. Methylation study of a population environmentally exposed to arsenic in drinking water. Environ Health Perspect 104, 620–628. <https://doi.org/10.1289/ehp.96104620>

Hoque, B.A., Hoque, M.M., 1994. Partnership in rural water supply and sanitation: a case study from Bangladesh. Health Policy and Planning 9, 288–293. <https://doi.org/10.1093/heapol/9.3.288>

Hoque, B.A., Hoque, M.M., Ahmed, T., Islam, S., Azad, A.K., Ali, N., Hossain, M., Hossain, M.S., 2004. Demand-based water options for arsenic mitigation: an experience from rural Bangladesh. Public Health 118, 70–77. <https://doi.org/10.1016/S0033-3506(03)00135-5>

Hoque, B.A., Mahmood, A.A., Quadiruzzaman, M., Khan, F., Ahmed, S.A., Shafique, S., Rahman, M., Morshed, G., Chowdhury, T., Rahman, M.M., Khan, F.H., Shahjahan, M., Begum, M., Hoque, M.M., 2000. Recommendations for water supply in arsenic mitigation: a case study from Bangladesh. Public Health (Stockton) 114, 488–494.

Hoque, S.F., Hope, R., 2018. The water diary method – proof-of-concept and policy implications for monitoring water use behaviour in rural Kenya. Water Policy 20, 725–743. <https://doi.org/10.2166/wp.2018.179>

Hossain, K., Suzuki, T., Hasibuzzaman, M.M., Islam, Md.S., Rahman, A., Paul, S.K., Tanu, T., Hossain, S., Saud, Z.A., Rahman, M., Nikkon, F., Miyataka, H., Himeno, S., Nohara, K., 2017. Chronic exposure to arsenic, LINE-1 hypomethylation, and blood pressure: a cross-sectional study in Bangladesh. Environ Health 16, 20. <https://doi.org/10.1186/s12940-017-0231-7>

Hossain, M.A., Rahman, M.M., Murrill, M., Das, B., Roy, B., Dey, S., Maity, D., Chakraborti, D., 2013. Water consumption patterns and factors contributing to water consumption in arsenic affected population of rural West Bengal, India. Sci Total Environ 463–464, 1217–1224. <https://doi.org/10.1016/j.scitotenv.2012.06.057>

Hossain, M.A., Sultana, F., Chowdhury, M.H., Khan, M.K., 2019. Environmental Situation in Some Selected Villages of Bangladesh. Mymensingh Med J 28, 508–514.

Houweling, E., Hall, R., Carzolio, M., Vance, E., 2016. ‘My Neighbour Drinks Clean Water, While I Continue To Suffer’: An Analysis of the Intra-Community Impacts of a Rural Water Supply Project in Mozambique. Journal of Development Studies 53. <https://doi.org/10.1080/00220388.2016.1224852>

Howe, H.L., Wolfgang, P.E., Burnett, W.S., Nasca, P.C., Youngblood, L., 1989. Cancer incidence following exposure to drinking water with asbestos leachate. Public Health Rep 104, 251–256.

Hsu, L.-I., Hsieh, F.-I., Wang, Y.-H., Lai, T.-S., Wu, M.-M., Chen, C.-J., Chiou, H.-Y., Hsu, K.-H., 2017. Arsenic Exposure From Drinking Water and the Incidence of CKD in Low to Moderate Exposed Areas of Taiwan: A 14-Year Prospective Study. Am J Kidney Dis 70, 787–797. <https://doi.org/10.1053/j.ajkd.2017.06.012>

Hsu, Y.-H., Li, S.-Y., Chiou, H.-Y., Yeh, P.-M., Liou, J.-C., Hsueh, Y.-M., Chang, S.-H., Chen, C.-J., 1997. Spontaneous and induced sister chromatid exchanges and delayed cell proliferation in peripheral lymphocytes of Bowen’s disease patients and matched controls of arseniasis-hyperendemic villages in Taiwan. Mutation Research/Reviews in Mutation Research 386, 241–251. <https://doi.org/10.1016/S1383-5742(97)00007-0>

Hu, X.C., Tokranov, A.K., Liddie, J., Zhang, X., Grandjean, P., Hart, J.E., Laden, F., Sun, Q., Yeung, L.W.Y., Sunderland, E.M., 2019. Tap Water Contributions to Plasma Concentrations of Poly- and Perfluoroalkyl Substances (PFAS) in a Nationwide Prospective Cohort of U.S. Women. Environ Health Perspect 127, 67006. <https://doi.org/10.1289/EHP4093>

Huang, J.-W., Cheng, Y.-Y., Sung, T.-C., Guo, H.-R., Sthiannopkao, S., 2014. Association between arsenic exposure and diabetes mellitus in Cambodia. Biomed Res Int 2014, 683124. <https://doi.org/10.1155/2014/683124>

Huang, M., Chen, X., Shao, D., Zhao, Y., Wang, W., Wong, M.H., 2014. Risk assessment of arsenic and other metals via atmospheric particles, and effects of atmospheric exposure and other demographic factors on their accumulations in human scalp hair in urban area of Guangzhou, China. Ecotoxicol Environ Saf 102, 84–92. <https://doi.org/10.1016/j.ecoenv.2013.12.027>

Huang, Y., Ma, X., Tan, Y., Wang, L., Wang, J., Lan, L., Qiu, Z., Luo, J., Zeng, H., Shu, W., 2019. Consumption of Very Low Mineral Water Is Associated with Lower Bone Mineral Content in Children. J Nutr 149, 1994–2000. <https://doi.org/10.1093/jn/nxz161>

Huang, Y.-L., Hsueh, Y.-M., Huang, Y.-K., Yip, P.-K., Yang, M.-H., Chen, C.-J., 2009. Urinary arsenic methylation capability and carotid atherosclerosis risk in subjects living in arsenicosis-hyperendemic areas in southwestern Taiwan. Sci Total Environ 407, 2608–2614. <https://doi.org/10.1016/j.scitotenv.2008.12.061>

Huber, D.R., Blount, B.C., Mage, D.T., Letkiewicz, F.J., Kumar, A., Allen, R.H., 2011. Estimating perchlorate exposure from food and tap water based on US biomonitoring and occurrence data. J Expo Sci Environ Epidemiol 21, 395–407. <https://doi.org/10.1038/jes.2010.31>

Huda, N., Hossain, S., Rahman, M., Karim, M.R., Islam, K., Mamun, A.A., Hossain, M.I., Mohanto, N.C., Alam, S., Aktar, S., Arefin, A., Ali, N., Salam, K.A., Aziz, A., Saud, Z.A., Miyataka, H., Himeno, S., Hossain, K., 2014. Elevated levels of plasma uric acid and its relation to hypertension in arsenic-endemic human individuals in Bangladesh. Toxicol Appl Pharmacol 281, 11–18. <https://doi.org/10.1016/j.taap.2014.09.011>

Hudgens, E.E., Drobna, Z., He, B., Le, X.C., Styblo, M., Rogers, J., Thomas, D.J., 2016. Biological and behavioral factors modify urinary arsenic metabolic profiles in a U.S. population. Environ Health 15, 62. <https://doi.org/10.1186/s12940-016-0144-x>

Humphrey, J.H., Mbuya, M.N.N., Ntozini, R., Moulton, L.H., Stoltzfus, R.J., Tavengwa, N.V., Mutasa, K., Majo, F., Mutasa, B., Mangwadu, G., Chasokela, C.M., Chigumira, A., Chasekwa, B., Smith, L.E., Tielsch, J.M., Jones, A.D., Manges, A.R., Maluccio, J.A., Prendergast, A.J., Humphrey, J.H., Jones, A.D., Manges, A., Mangwadu, G., Maluccio, J.A., Mbuya, M.N.N., Moulton, L.H., Ntozini, R., Prendergast, A.J., Stoltzfus, R.J., Tielsch, J.M., Chasokela, C., Chigumira, A., Heylar, W., Hwena, P., Kembo, G., Majo, F.D., Mutasa, B., Mutasa, K., Rambanepasi, P., Sauramba, V., Tavengwa, N.V., Keilen, F.V.D., Zambezi, C., Chidhanguro, D., Chigodora, D., Chipanga, J.F., Gerema, G., Magara, T., Mandava, M., Mavhudzi, T., Mazhanga, C., Muzaradope, G., Mwapaura, M.T., Phiri, Simon, Tengende, A., Banda, C., Chasekwa, B., Chidamba, L., Chidawanyika, T., Chikwindi, E., Chingaona, L.K., Chiorera, C.K., Dandadzi, A., Govha, M., Gumbo, H., Gwanzura, K.T., Kasaru, S., Makasi, R., Matsika, A.M., Maunze, D., Mazarura, E., Mpofu, Eddington, Mushonga, J., Mushore, T.E., Muzira, T., Nembaware, N., Nkiwane, Sibongile, Nyamwino, P., Rukobo, S.D., Runodamoto, T., Seremwe, S., Simango, P., Tome, J., Tsenesa, B., Amadu, U., Bangira, B., Chiveza, D., Hove, P., Jombe, H.A., Kujenga, D., Madhuyu, L., Makoni, P.M., Maramba, N., Maregere, B., Marumani, E., Masakadze, E., Mazula, P., Munyanyi, C., Musanhu, G., Mushanawani, R.C., Mutsando, S., Nazare, F., Nyarambi, M., Nzuda, W., Sigauke, T., Solomon, M., Tavengwa, T., Biri, F., Chafanza, M., Chaitezvi, C., Chauke, T., Chidzomba, C., Dadirai, T., Fundira, C., Gambiza, A.C., Godzongere, T., Kuona, M., Mafuratidze, T., Mapurisa, I., Mashedze, T., Moyo, Nokuthula, Musariri, C., Mushambadope, M., Mutsonziwa, T.R., Muzondo, A., Mwareka, R., Nyamupfukudza, J., Saidi, B., Sakuhwehwe, T., Sikalima, G., Tembe, J., Chekera, T.E., Chihombe, O., Chikombingo, M., Chirinda, T., Chivizhe, A., Hove, R., Kufa, R., Machikopa, T.F., Mandaza, W., Mandongwe, L., Manhiyo, F., Manyaga, E., Mapuranga, P., Matimba, F.S., Matonhodze, P., Mhuri, S., Mike, J., Ncube, B., Nderecha, W.T.S., Noah, M., Nyamadzawo, C., Penda, J., Saidi, A., Shonhayi, S., Simon, C., Tichagwa, M., Chamakono, R., Chauke, A., Gatsi, A.F., Hwena, B., Jawi, H., Kaisa, B., Kamutanho, S., Kaswa, T., Kayeruza, P., Lunga, J., Magogo, N., Manyeruke, D., Mazani, P., Mhuriyengwe, F., Mlambo, F., Moyo, Stephen, Mpofu, T., Mugava, M., Mukungwa, Y., Muroyiwa, F., Mushonga, E., Nyekete, S., Rinashe, T., Sibanda, K., Chemhuru, M., Chikunya, J., Chikwavaire, V.F., Chikwiriro, C., Chimusoro, A., Chinyama, J., Gwinji, G., Hoko-Sibanda, N., Kandawasvika, R., Madzimure, T., Maponga, B., Mapuranga, A., Marembo, J., Matsunge, L., Maunga, S., Muchekeza, M., Muti, M., Nyamana, M., Azhuda, E., Bhoroma, U., Biriyadi, A., Chafota, E., Chakwizira, A., Chamhamiwa, A., Champion, T., Chazuza, S., Chikwira, B., Chingozho, C., Chitabwa, A., Dhurumba, A., Furidzirai, A., Gandanga, A., Gukuta, C., Macheche, B., Marihwi, B., Masike, B., Mutangandura, E., Mutodza, B., Mutsindikwa, A., Mwale, A., Ndhlovu, R., Nduna, N., Nyamandi, C., Ruvata, E., Sithole, B., Urayai, R., Vengesa, B., Zorounye, M., Bamule, M., Bande, M., Chahuruva, K., Chidumba, L., Chigove, Z., Chiguri, K., Chikuni, S., Chikwanda, R., Chimbi, T., Chingozho, M., Chinhamo, O., Chinokuramba, R., Chinyoka, C., Chipenzi, X., Chipute, R., Chiribhani, G., Chitsinga, M., Chiwanga, C., Chiza, A., Chombe, F., Denhere, M., Dhamba, E., Dhamba, M., Dube, J., Dzimbanhete, F., Dzingai, G., Fusira, S., Gonese, M., Gota, J., Gumure, K., Gwaidza, P., Gwangwava, M., Gwara, W., Gwauya, M., Gwiba, M., Hamauswa, J., Hlasera, S., Hlukani, E., Hotera, J., Jakwa, L., Jangara, G., Janyure, M., Jari, C., Juru, D., Kapuma, T., Konzai, P., Mabhodha, M., Maburutse, S., Macheka, C., Machigaya, T., Machingauta, F., Machokoto, E., Madhumba, E., Madziise, L., Madziva, C., Madzivire, M., Mafukise, M., Maganga, M., Maganga, S., Mageja, E., Mahanya, M., Mahaso, E., Mahleka, S., Makanhiwa, P., Makarudze, M., Makeche, C., Makopa, N., Makumbe, R., Mandire, M., Mandiyanike, E., Mangena, E., Mangiro, F., Mangwadu, A., Mangwengwe, T., Manhidza, J., Manhovo, F., Manono, I., Mapako, S., Mapfumo, E., Mapfumo, Timothy, Mapuka, J., Masama, D., Masenge, G., Mashasha, M., Mashivire, V., Matunhu, M., Mavhoro, P., Mawuka, G., Mazango, I., Mazhata, N., Mazuva, D., Mazuva, M., Mbinda, F., Mborera, J., Mfiri, U., Mhandu, F., Mhike, C., Mhike, T., Mhuka, A., Midzi, J., Moyo, Siqondeni, Mpundu, M., Msekiwa, N., Msindo, D., Mtisi, C., Muchemwa, G., Mujere, N., Mukaro, E., Muketiwa, K., Mungoi, S., Munzava, E., Muoki, R., Mupura, H., Murerwa, E., Murisi, C., Muroyiwa, L., Muruvi, M., Musemwa, N., Mushure, C., Mutero, J., Mutero, P., Mutumbu, P., Mutya, C., Muzanango, L., Muzembi, M., Muzungunye, D., Mwazha, V., Ncube, T., Ndava, T., Ndlovu, N., Nehowa, P., Ngara, D., Nguruve, L., Nhigo, P., Nkiwane, Samukeliso, Nyanyai, L., Nzombe, J., Office, E., Paul, B., Pavari, S., Ranganai, S., Ratisai, S., Rugara, M., Rusere, P., Sakala, J., Sango, P., Shava, S., Shekede, M., Shizha, C., Sibanda, T., Tapambwa, N., Tembo, J., Tinago, N., Tinago, V., Toindepi, T., Tovigepi, J., Tuhwe, M., Tumbo, K., Zaranyika, T., Zaru, T., Zimidzi, K., Zindo, M., Zindonda, M., Zinhumwe, N., Zishiri, L., Ziyambi, E., Zvinowanda, J., Bepete, E., Chiwira, C., Chuma, N., Fari, A., Gavi, S., Gunha, V., Hakunandava, F., Huku, C., Hungwe, G., Maduke, G., Manyewe, E., Mapfumo, Tecla, Marufu, I., Mashiri, Chenesai, Mazenge, S., Mbinda, E., Mhuri, A., Muguti, C., Munemo, L., Musindo, L., Ngada, L., Nyembe, D., Taruvinga, R., Tobaiwa, E., Banda, S., Chaipa, J., Chakaza, P., Chandigere, M., Changunduma, A., Chibi, C., Chidyagwai, O., Chidza, E., Chigatse, N., Chikoto, L., Chingware, V., Chinhamo, J., Chinhoro, M., Chiripamberi, A., Chitavati, E., Chitiga, R., Chivanga, N., Chivese, T., Chizema, F., Dera, S., Dhliwayo, A., Dhononga, P., Dimingo, E., Dziyani, M., Fambi, T., Gambagamba, L., Gandiyari, S., Gomo, C., Gore, S., Gundani, J., Gundani, R., Gwarima, L., Gwaringa, C., Gwenya, S., Hamilton, R., Hlabano, A., Hofisi, E., Hofisi, F., Hungwe, S., Hwacha, S., Hwara, A., Jogwe, R., Kanikani, A., Kuchicha, L., Kutsira, M., Kuziyamisa, K., Kuziyamisa, M., Kwangware, B., Lozani, P., Mabuto, J., Mabuto, V., Mabvurwa, L., Machacha, R., Machaya, C., Madembo, R., Madya, S., Madzingira, S., Mafa, L., Mafuta, F., Mafuta, J., Mahara, A., Mahonye, S., Maisva, A., Makara, A., Makover, M., Mambongo, E., Mambure, M., Mandizvidza, E., Mangena, G., Manjengwa, E., Manomano, J., Mapfumo, M., Mapfurire, A., Maphosa, L., Mapundo, J., Mare, D., Marecha, F., Marecha, S., Mashiri, Christine, Masiya, M., Masuku, T., Masvimbo, P., Matambo, S., Matarise, G., Matinanga, L., Matizanadzo, J., Maunganidze, M., Mawere, B., Mawire, C., Mazvanya, Y., Mbasera, M., Mbono, M., Mhakayakora, C., Mhlanga, N., Mhosva, B., Moyo, Nomuhle, Moyo, O., Moyo, R., Mpakami, C., Mpedzisi, R., Mpofu, Elizabeth, Mpofu, Estery, Mtetwa, M., Muchakachi, J., Mudadada, T., Mudzingwa, K., Mugwira, M., Mukarati, T., Munana, A., Munazo, J., Munyeki, O., Mupfeka, P., Murangandi, G., Muranganwa, M., Murenjekwa, J., Muringo, N., Mushaninga, T., Mutaja, F., Mutanha, D., Mutemeri, P., Mutero, B., Muteya, E., Muvembi, S., Muzenda, T., Mwenjota, A., Ncube, S., Ndabambi, T., Ndava, N., Ndlovu, E., Nene, E., Ngazimbi, E., Ngwalati, A., Nyama, T., Nzembe, A., Pabwaungana, E., Phiri, Sekai, Pukuta, R., Rambanapasi, M., Rera, T., Samanga, V., Shirichena, S., Shoko, C., Shonhe, M., Shuro, C., Sibanda, J., Sibangani, E., Sibangani, N., Sibindi, N., Sitotombe, M., Siwawa, P., Tagwirei, M., Taruvinga, P., Tavagwisa, A., Tete, E., Tete, Y., Thandiwe, E., Tibugari, A., Timothy, S., Tongogara, R., Tshuma, L., Tsikira, M., Tumba, C., Watinaye, R., Zhiradzango, E., Zimunya, E., Zinengwa, L., Ziupfu, M., Ziyambe, J., Church, J.A., Desai, A., Fundira, D., Gough, E., Kambarami, R.A., Matare, C.R., Malaba, T.R., Mupfudze, T., Ngure, F., Smith, L.E., Curtis, V., Dickin, K.L., Habicht, J.-P., Masimirembwa, C., Morgan, P., Pelto, G.H., Sheffner-Rogers, C., Thelingwani, R., Turner, P., Zungu, L., Makadzange, T., Mujuru, H.A., Nyachowe, C., Chakadai, R., Chanyau, G., Makamure, M.G., Chiwariro, H., Mtetwa, T., Chikunya, J., Maguwu, L., Nyadundu, S., Moyo, T., Chayima, B., Mvindi, L., Rwenhamo, P., Muzvarwandoga, S., Chimukangara, R., Njovo, H., Makoni, T., 2019. Independent and combined effects of improved water, sanitation, and hygiene, and improved complementary feeding, on child stunting and anaemia in rural Zimbabwe: a cluster-randomised trial. The Lancet Global Health 7, e132–e147. <https://doi.org/10.1016/S2214-109X(18)30374-7>

Hurley, S., Houtz, E., Goldberg, D., Wang, M., Park, J.-S., Nelson, D.O., Reynolds, P., Bernstein, L., Anton-Culver, H., Horn-Ross, P., Petreas, M., 2016. Preliminary Associations between the Detection of Perfluoroalkyl Acids (PFAAs) in Drinking Water and Serum Concentrations in a Sample of California Women. Environ. Sci. Technol. Lett. 3, 264–269. <https://doi.org/10.1021/acs.estlett.6b00154>

Huyck, K.L., Kile, M.L., Mahiuddin, G., Quamruzzaman, Q., Rahman, M., Breton, C.V., Dobson, C.B., Frelich, J., Hoffman, E., Yousuf, J., Afroz, S., Islam, S., Christiani, D.C., 2007. Maternal arsenic exposure associated with low birth weight in Bangladesh. J Occup Environ Med 49, 1097–1104. <https://doi.org/10.1097/JOM.0b013e3181566ba0>

Hwang, B.-F., Jaakkola, J.J.K., 2012. Risk of Stillbirth in the Relation to Water Disinfection By-Products: A Population-Based Case-Control Study in Taiwan. PLOS ONE 7, e33949. <https://doi.org/10.1371/journal.pone.0033949>

Hyllestad, S., Veneti, L., Bugge, A.B., Rosenberg, T.G., Nygård, K., Aavitsland, P., 2019. Compliance with water advisories after water outages in Norway. BMC Public Health 19, 1188. <https://doi.org/10.1186/s12889-019-7504-8>

Hynds, P.D., Misstear, B.D., Gill, L.W., 2012. Investigation of water consumption patterns among Irish adults for waterborne quantitative microbial risk assessment (QMRA). J Water Health 10, 453–464. <https://doi.org/10.2166/wh.2012.025>

Iglesia, I., Guelinckx, I., De Miguel-Etayo, P.M., González-Gil, E.M., Salas-Salvadó, J., Kavouras, S.A., Gandy, J., Martínez, H., Bardosono, S., Abdollahi, M., Nasseri, E., Jarosz, A., Ma, G., Carmuega, E., Thiébaut, I., Moreno, L.A., 2015. Total fluid intake of children and adolescents: cross-sectional surveys in 13 countries worldwide. Eur J Nutr 54 Suppl 2, 57–67. <https://doi.org/10.1007/s00394-015-0946-6>

Igra, A.M., Harari, F., Lu, Y., Casimiro, E., Vahter, M., 2016. Boron exposure through drinking water during pregnancy and birth size. Environ Int 95, 54–60. <https://doi.org/10.1016/j.envint.2016.07.017>

Iijima, Y., Karama, M., Oundo, J.O., Honda, T., 2001. Prevention of bacterial diarrhea by pasteurization of drinking water in Kenya. Microbiol Immunol 45, 413–416. <https://doi.org/10.1111/j.1348-0421.2001.tb02639.x>

Ileka-Priouzeau, S., Campagna, C., Legay, C., Deonandan, R., Rodriguez, M.J., Levallois, P., 2015. Women exposure during pregnancy to haloacetaldehydes and haloacetonitriles in drinking water and risk of small-for-gestational-age neonate. Environ Res 137, 338–348. <https://doi.org/10.1016/j.envres.2015.01.005>

Inobaya, M.T., Chau, T.N., Ng, S.-K., MacDougall, C., Olveda, R.M., Tallo, V.L., Landicho, J.M., Malacad, C.M., Aligato, M.F., Guevarra, J.B., Ross, A.G., 2018. Mass drug administration and the sustainable control of schistosomiasis: an evaluation of treatment compliance in the rural Philippines. Parasites & Vectors 11, 441. <https://doi.org/10.1186/s13071-018-3022-2>

Inoue-Choi, M., Jones, R.R., Anderson, K.E., Cantor, K.P., Cerhan, J.R., Krasner, S., Robien, K., Weyer, P.J., Ward, M.H., 2015. Nitrate and nitrite ingestion and risk of ovarian cancer among postmenopausal women in Iowa. Int J Cancer 137, 173–182. <https://doi.org/10.1002/ijc.29365>

Inoue-Choi, M., Ward, M.H., Cerhan, J.R., Weyer, P.J., Anderson, K.E., Robien, K., 2012. Interaction of nitrate and folate on the risk of breast cancer among postmenopausal women. Nutr Cancer 64, 685–694. <https://doi.org/10.1080/01635581.2012.687427>

Inoue-Choi, M., Weyer, P.J., Jones, R.R., Booth, B.J., Cantor, K.P., Robien, K., Ward, M.H., 2016. Atrazine in public water supplies and risk of ovarian cancer among postmenopausal women in the Iowa Women’s Health Study. Occup Environ Med 73, 582–587. <https://doi.org/10.1136/oemed-2016-103575>

Iriani, D.U., Matsukawa, T., Tadjudin, M.K., Itoh, H., Yokoyama, K., 2012. Cross-sectional study on the effects of socioeconomic factors on lead exposure in children by gender in Serpong, Indonesia. Int J Environ Res Public Health 9, 4135–4149. <https://doi.org/10.3390/ijerph9114135>

Iroegbu, C.U., Ene-Obong, H.N., Uwaegbute, A.C., Amazigo, U.V., 2000. Bacteriological quality of weaning food and drinking water given to children of market women in Nigeria: implications for control of diarrhoea. J Health Popul Nutr 18, 157–162.

Ishii, N., Terao, T., Araki, Y., Kohno, K., Mizokami, Y., Shiotsuki, I., Hatano, K., Makino, M., Kodama, K., Iwata, N., 2015. Low risk of male suicide and lithium in drinking water. J Clin Psychiatry 76, 319–326. <https://doi.org/10.4088/JCP.14m09218>

Ishii, N., Terao, T., Matsuzaki, H., Inoue, T., Takaesu, Y., Kohno, K., Takeshima, M., Baba, H., Honma, H., 2017. Lithium in drinking water may be negatively associated with depressive temperament in the nonclinical population. Clinical Neuropsychopharmacology and Therapeutics 8, 7–11. <https://doi.org/10.5234/cnpt.8.7>

Islam, L.N., Nabi, A.H.M.N., Rahman, M.M., Khan, M.A., Kazi, A.I., 2004. Association of clinical complications with nutritional status and the prevalence of leukopenia among arsenic patients in Bangladesh. Int J Environ Res Public Health 1, 74–82. <https://doi.org/10.3390/ijerph2004020074>

Islam, Md.A., Sakakibara, H., Karim, Md.R., Sekine, M., 2011. Evaluation of risk communication for rural water supply management: a case study of a coastal area of Bangladesh. Journal of Risk Research 14, 1237–1262. <https://doi.org/10.1080/13669877.2011.574315>

Islam, M.R., Attia, J., Alauddin, M., McEvoy, M., McElduff, P., Slater, C., Islam, M.M., Akhter, A., d’Este, C., Peel, R., Akter, S., Smith, W., Begg, S., Milton, A.H., 2014. Availability of arsenic in human milk in women and its correlation with arsenic in urine of breastfed children living in arsenic contaminated areas in Bangladesh. Environ Health 13, 101. <https://doi.org/10.1186/1476-069X-13-101>

Islam, R., Khan, I., Hassan, S.N., McEvoy, M., D’Este, C., Attia, J., Peel, R., Sultana, M., Akter, S., Milton, A.H., 2012. Association between type 2 diabetes and chronic arsenic exposure in drinking water: a cross sectional study in Bangladesh. Environ Health 11, 38. <https://doi.org/10.1186/1476-069X-11-38>

Isoke, J., van Dijk, M.P., 2014. Factors influencing selection of drinking water technologies for urban informal settlements in Kampala. Water and Environment Journal 28, 423–433. <https://doi.org/10.1111/wej.12058>

Isoke, J., van Dijk, M.P., 2013. Factors influencing selection of drinking water technologies for urban informal settlements in Kampala: Drinking water technologies in Kampala. Water Environ J n/a-n/a. <https://doi.org/10/gqbxvm>

Issa, M., McHenry, M., Issa, A.A., Blackwood, R.A., 2015. Access to Safe Water and Personal Hygiene Practices in the Kulandia Refugee Camp (Jerusalem). Infect Dis Rep 7, 6040. <https://doi.org/10.4081/idr.2015.6040>

Iszatt, N., Nieuwenhuijsen, M.J., Nelson, P., Elliott, P., Toledano, M.B., 2011. Water consumption and use, trihalomethane exposure, and the risk of hypospadias. Pediatrics 127, e389-397. <https://doi.org/10.1542/peds.2009-3356>

Izanloo, H., Asadi-Ghalhari, M., Mohebi, S., Hasanpoor, F., Hozoori, M., 2019. The relation between brewed tea and drinking water with urinary fluoride concentration among youths. Progr Nutr 21, 422–427. <https://doi.org/10.23751/pn.v21i1-S.5998>

Jabbar, F., Asif, M., Dutani, H., Hussain, A., Malik, A., Kamal, M.A., Rasool, M., 2015. Assessment of the role of general, biochemical and family history characteristics in kidney stone formation. Saudi J Biol Sci 22, 65–68. <https://doi.org/10.1016/j.sjbs.2014.06.002>

Jacqmin, H., Commenges, D., Letenneur, L., Barberger-Gateau, P., Dartigues, J.F., 1994. Components of drinking water and risk of cognitive impairment in the elderly. Am J Epidemiol 139, 48–57. <https://doi.org/10.1093/oxfordjournals.aje.a116934>

Jahan, Y., Moriyama, M., Hossain, S., Rahman, M.M., Ferdous, F., Ahmed, S., Das, S.K., Hossain, M.I., Faruque, A.S.G., Ahmed, T., Chisti, M.J., 2019. Relation of childhood diarrheal morbidity with the type of tube well used and associated factors of Shigella sonnei diarrhea in rural Bangladesh site of the Global Enteric Multicenter Study. Trop Med Health 47, 29. <https://doi.org/10.1186/s41182-019-0158-5>

Jain, R.B., 2015. Association of arsenic exposure with smoking, alcohol, and caffeine consumption: data from NHANES 2005-2010. Environ Toxicol Pharmacol 39, 651–658. <https://doi.org/10.1016/j.etap.2015.01.011>

Jaka, H., Mushi, M.F., Mirambo, M.M., Wilson, L., Seni, J., Mtebe, M., Mshana, S.E., 2016. Sero-prevalence and associated factors of Helicobacter pylori infection among adult patients with dyspepsia attending the gastroenterology unit in a tertiary hospital in Mwanza, Tanzania. Afr Health Sci 16, 684–689. <https://doi.org/10.4314/ahs.v16i3.7>

Jang, S., Cheon, C., Jang, B.-H., Park, S., Oh, S.-M., Shin, Y.-C., Ko, S.-G., 2016. Relationship Between Water Intake and Metabolic/Heart Diseases: Based on Korean National Health and Nutrition Examination Survey. Osong Public Health Res Perspect 7, 289–295. <https://doi.org/10.1016/j.phrp.2016.08.007>

Janmohamed, A., Karakochuk, C.D., McLean, J., Green, T.J., 2016. Improved Sanitation Facilities are Associated with Higher Body Mass Index and Higher Hemoglobin Concentration Among Rural Cambodian Women in the First Trimester of Pregnancy. Am J Trop Med Hyg 95, 1211–1215. <https://doi.org/10.4269/ajtmh.16-0278>

Javins, B., Hobbs, G., Ducatman, A.M., Pilkerton, C., Tacker, D., Knox, S.S., 2013. Circulating maternal perfluoroalkyl substances during pregnancy in the C8 Health Study. Environ Sci Technol 47, 1606–1613. <https://doi.org/10.1021/es3028082>

Jayasumana, C., Paranagama, P., Agampodi, S., Wijewardane, C., Gunatilake, S., Siribaddana, S., 2015. Drinking well water and occupational exposure to Herbicides is associated with chronic kidney disease, in Padavi-Sripura, Sri Lanka. Environ Health 14, 6. <https://doi.org/10.1186/1476-069X-14-6>

Jensen, O.M., 1982. Nitrate in drinking water and cancer in northern Jutland, Denmark, with special reference to stomach cancer. Ecotoxicol Environ Saf 6, 258–267. <https://doi.org/10.1016/0147-6513(82)90016-1>

Jeong, S.W., Lee, C.K., Suh, C.H., Kim, K.H., Son, B.C., Kim, J.H., Lee, J.T., Lee, S.W., Park, Y.B., Lee, J.W., Yu, S.-D., Moon, C.S., Kim, D.H., Lee, S.Y., 2014. Blood lead concentration and related factors in Korea from the 2008 National Survey for Environmental Pollutants in the Human Body. Int J Hyg Environ Health 217, 871–877. <https://doi.org/10.1016/j.ijheh.2014.06.006>

Jia, Y., Hu, T., Hang, C.-Y., Yang, R., Li, X., Chen, Z.-L., Mei, Y.-D., Zhang, Q.-H., Huang, K.-C., Xiang, Q.-Y., Pan, X.-Y., Yan, Y.-T., Wang, X.-L., Wang, S.-S., Hang, Z., Tang, F.-X., Liu, D., Zhou, J., Xi, L., Wang, H., Lu, Y.-P., Ma, D., Wang, S.-X., Li, S., 2012. Case-Control Study of Diet in Patients with Cervical Cancer or Precancerosis in Wufeng, a High Incidence Region in China. Asian Pacific Journal of Cancer Prevention 13, 5299–5302. <https://doi.org/10.7314/APJCP.2012.13.10.5299>

Jiang, X., Castelao, J.E., Groshen, S., Cortessis, V.K., Shibata, D.K., Conti, D.V., Gago-Dominguez, M., 2008. Water intake and bladder cancer risk in Los Angeles County. Int J Cancer 123, 1649–1656. <https://doi.org/10.1002/ijc.23711>

Jin, C., Yao, Q., Zhou, Y., Shi, R., Gao, Y., Wang, C., Tian, Y., 2020. Exposure to triclosan among pregnant women in northern China: urinary concentrations, sociodemographic predictors, and seasonal variability. Environ Sci Pollut Res Int 27, 4840–4848. <https://doi.org/10.1007/s11356-019-07294-1>

Jinadu, M.K., Olusi, S.O., Agun, J.I., Fabiyi, A.K., 1991. Childhood diarrhoea in rural Nigeria. I. Studies on prevalence, mortality and socio-environmental factors. J Diarrhoeal Dis Res 9, 323–327.

Johnson, R.C., Boni, G., Barogui, Y., Sopoh, G.E., Houndonougbo, M., Anagonou, E., Agossadou, D., Diez, G., Boko, M., 2015. Assessment of water, sanitation, and hygiene practices and associated factors in a Buruli ulcer endemic district in Benin (West Africa). BMC Public Health 15, 801. <https://doi.org/10.1186/s12889-015-2154-y>

Johnson, S., Joshi, V., 1982. Dracontiasis in Rajasthan. VI. Epidemiology of dracontiasis in Barmer district, Western Rajasthan, India. Int J Epidemiol 11, 26–30. <https://doi.org/10.1093/ije/11.1.26>

Johnston, A.R., Gillespie, T.R., Rwego, I.B., McLachlan, T.L.T., Kent, A.D., Goldberg, T.L., 2010. Molecular epidemiology of cross-species Giardia duodenalis transmission in western Uganda. PLoS Negl Trop Dis 4, e683. <https://doi.org/10.1371/journal.pntd.0000683>

Jones, Andria Q., Dewey, C.E., Doré, K., Majowicz, S.E., McEwen, S.A., Waltner-Toews, D., Henson, S.J., Mathews, E., 2007. A qualitative exploration of the public perception of municipal drinking water. Water Policy 9, 425–438. <https://doi.org/10.2166/wp.2007.019>

Jones, A. Q., Majowicz, S.E., Edge, V.L., Thomas, M.K., MacDougall, L., Fyfe, M., Atashband, S., Kovacs, S.J., 2007. Drinking water consumption patterns in British Columbia: an investigation of associations with demographic factors and acute gastrointestinal illness. Sci Total Environ 388, 54–65. <https://doi.org/10.1016/j.scitotenv.2007.08.028>

Jones, R.R., DellaValle, C.T., Weyer, P.J., Robien, K., Cantor, K.P., Krasner, S., Beane Freeman, L.E., Ward, M.H., 2019. Ingested nitrate, disinfection by-products, and risk of colon and rectal cancers in the Iowa Women’s Health Study cohort. Environ Int 126, 242–251. <https://doi.org/10.1016/j.envint.2019.02.010>

Jones, R.R., Weyer, P.J., DellaValle, C.T., Inoue-Choi, M., Anderson, K.E., Cantor, K.P., Krasner, S., Robien, K., Freeman, L.E.B., Silverman, D.T., Ward, M.H., 2016. Nitrate from Drinking Water and Diet and Bladder Cancer Among Postmenopausal Women in Iowa. Environ Health Perspect 124, 1751–1758. <https://doi.org/10.1289/EHP191>

Jones, R.R., Weyer, P.J., DellaValle, C.T., Robien, K., Cantor, K.P., Krasner, S., Beane Freeman, L.E., Ward, M.H., 2017. Ingested Nitrate, Disinfection By-products, and Kidney Cancer Risk in Older Women: Epidemiology 28, 703–711. <https://doi.org/10.1097/EDE.0000000000000647>

Joshi, A., Prasad, S., Kasav, J.B., Segan, M., Singh, A.K., 2013. Water and sanitation hygiene knowledge attitude practice in urban slum settings. Glob J Health Sci 6, 23–34. <https://doi.org/10.5539/gjhs.v6n2p23>

Jovanovic, D., Rasic-Milutinovic, Z., Paunovic, K., Jakovljevic, B., Plavsic, S., Milosevic, J., 2013. Low levels of arsenic in drinking water and type 2 diabetes in Middle Banat region, Serbia. Int J Hyg Environ Health 216, 50–55. <https://doi.org/10.1016/j.ijheh.2012.01.001>

Jovanović, D.D., Paunović, K., Manojlović, D.D., Jakovljević, B., Rasic-Milutinović, Z., Dojcinović, B.P., 2012. Arsenic in drinking water and acute coronary syndrome in Zrenjanin municipality, Serbia. Environ Res 117, 75–82. <https://doi.org/10.1016/j.envres.2012.04.016>

Joyce, S.J., Cook, A., Newnham, J., Brenters, M., Ferguson, C., Weinstein, P., 2008. Water disinfection by-products and pre-labor rupture of membranes. Am J Epidemiol 168, 514–521. <https://doi.org/10.1093/aje/kwn188>

Ju, H., Zhang, J., Sun, C., 2018. Occurrence, spatial distribution and risk and hazard assessments of antibiotics in drinking water sources of a polluted large river basin in China. Aquatic Ecosystem Health & Management 21, 107–117. <https://doi.org/10.1080/14634988.2017.1391659>

Kabir, M.A., Al-Amin, A.Q., Alam, G.M., Matim, M.A., 2011. Early childhood mortality and affecting factors in developing countries: an experience from Bangladesh. International Journal of Pharmacology.

Kadono, T., Inaoka, T., Murayama, N., Ushijima, K., Nagano, M., Nakamura, S., Watanabe, C., Tamaki, K., Ohtsuka, R., 2002. Skin manifestations of arsenicosis in two villages in Bangladesh. Int J Dermatol 41, 841–846. <https://doi.org/10.1046/j.1365-4362.2002.01668.x>

Kafle, K., Balasubramanya, S., Horbulyk, T., 2019. Prevalence of chronic kidney disease in Sri Lanka: A profile of affected districts reliant on groundwater. Sci Total Environ 694, 133767. <https://doi.org/10.1016/j.scitotenv.2019.133767>

Källén, B.A., Robert, E., 2000. Drinking water chlorination and delivery outcome-a registry-based study in Sweden. Reprod Toxicol 14, 303–309. <https://doi.org/10.1016/s0890-6238(00)00086-1>

Kanarek, M.S., Conforti, P.M., Jackson, L.A., Cooper, R.C., Murchio, J.C., 1980. Asbestos in drinking water and cancer incidence in the San Francisco Bay area. Am J Epidemiol 112, 54–72. <https://doi.org/10.1093/oxfordjournals.aje.a112976>

Kanarek, M.S., Young, T.B., 1982. Drinking water treatment and risk of cancer death in Wisconsin. Environ Health Perspect 46, 179–186. <https://doi.org/10.1289/ehp.8246179>

Kang, Y., Kim, J., 2019. Age-specific risk factors for child anaemia in Myanmar: Analysis from the Demographic and Health Survey 2015-2016. Matern Child Nutr 15, e12870. <https://doi.org/10.1111/mcn.12870>

Kang, Y.-H., Cong, W., Zhang, X.-Y., Wang, C.-F., Shan, X.-F., Qian, A.-D., 2017. Hepatitis E virus seroprevalence among farmers, veterinarians and control subjects in Jilin province, Shandong province and Inner Mongolia Autonomous Region, China. J Med Virol 89, 872–877. <https://doi.org/10.1002/jmv.24693>

Kannan, K., Praamsma, M.L., Oldi, J.F., Kunisue, T., Sinha, R.K., 2009. Occurrence of perchlorate in drinking water, groundwater, surface water and human saliva from India. Chemosphere 76, 22–26. <http://dx.doi.org/10.1016/j.chemosphere.2009.02.054>

Karakochuk, C.D., Murphy, H.M., Whitfield, K.C., Barr, S.I., Vercauteren, S.M., Talukder, A., Porter, K., Kroeun, H., Eath, M., McLean, J., Green, T.J., 2015. Elevated levels of iron in groundwater in Prey Veng province in Cambodia: a possible factor contributing to high iron stores in women. J Water Health 13, 575–586. <https://doi.org/10.2166/wh.2014.297>

Karpas, Z., Paz-Tal, O., Lorber, A., Salonen, L., Komulainen, H., Auvinen, A., Saha, H., Kurttio, P., 2005. Urine, hair, and nails as indicators for ingestion of uranium in drinking water. Health Phys 88, 229–242. <https://doi.org/10.1097/01.hp.0000149883.69107.ab>

Karunanidhi, D., Aravinthasamy, P., Subramani, T., Roy, P.D., Srinivasamoorthy, K., 2020. Risk of Fluoride-Rich Groundwater on Human Health: Remediation Through Managed Aquifer Recharge in a Hard Rock Terrain, South India. Nat Resour Res 29, 2369–2395. <https://doi.org/10.1007/s11053-019-09592-4>

Kassim, I.A.R., Moloney, G., Busili, A., Nur, A.Y., Paron, P., Jooste, P., Gadain, H., Seal, A.J., 2014. Iodine Intake in Somalia Is Excessive and Associated with the Source of Household Drinking Water. The Journal of Nutrition 144, 375–381. <https://doi.org/10.3945/jn.113.176693>

Kaur, S., Nieuwenhuijsen, M.J., Ferrier, H., Steer, P., 2004. Exposure of pregnant women to tap water related activities. Occupational and Environmental Medicine 61, 454–460. <https://doi.org/10.1136/oem.2003.007351>

Kausar, S., Maann, A.A., Zafar, M.I., Ali, T., 2012. A Study of Storage System for Drinking Water and its Health Impacts at Household Level in Punjab, Pakistan. Pakistan J. of Nutrition 11, 591–595. <https://doi.org/10.3923/pjn.2012.591.595>

Kavosi, E., Hassanzadeh Rostami, Z., Kavosi, Z., Nasihatkon, A., Moghadami, M., Heidari, M., 2014. Prevalence and determinants of under-nutrition among children under six: a cross-sectional survey in Fars province, Iran. Int J Health Policy Manag 3, 71–76. <https://doi.org/10.15171/ijhpm.2014.63>

Kazi, T.G., Arain, M.B., Baig, J.A., Jamali, M.K., Afridi, H.I., Jalbani, N., Sarfraz, R.A., Shah, A.Q., Niaz, A., 2009. The correlation of arsenic levels in drinking water with the biological samples of skin disorders. Sci Total Environ 407, 1019–1026. <https://doi.org/10.1016/j.scitotenv.2008.10.013>

Kenney, E.L., Long, M.W., Cradock, A.L., Gortmaker, S.L., 2015. Prevalence of Inadequate Hydration Among US Children and Disparities by Gender and Race/Ethnicity: National Health and Nutrition Examination Survey, 2009-2012. Am J Public Health 105, e113-118. <https://doi.org/10.2105/AJPH.2015.302572>

Kessing, L.V., Gerds, T.A., Knudsen, N.N., Jørgensen, L.F., Kristiansen, S.M., Voutchkova, D., Ernstsen, V., Schullehner, J., Hansen, B., Andersen, P.K., Ersbøll, A.K., 2017. Lithium in drinking water and the incidence of bipolar disorder: A nation-wide population-based study. Bipolar Disord 19, 563–567. <https://doi.org/10.1111/bdi.12524>

Keszei, A.P., Schouten, L.J., Driessen, A.L.C., Huysentruyt, C.J.R., Keulemans, Y.C.A., Goldbohm, R.A., Brandt, P.A. van den, 2014. Vegetable, fruit and nitrate intake in relation to the risk of Barrett’s oesophagus in a large Dutch cohort. British Journal of Nutrition 111, 1452–1462. <https://doi.org/10.1017/S0007114513003929>

Khadka, K.B., Lieberman, L.S., Giedraitis, V., Bhatta, L., Pandey, G., 2015. The socio-economic determinants of infant mortality in Nepal: analysis of Nepal Demographic Health Survey, 2011. BMC Pediatrics 15, 152. <https://doi.org/10.1186/s12887-015-0468-7>

Khan, A.E., Ireson, A., Kovats, S., Mojumder, S.K., Khusru, A., Rahman, A., Vineis, P., 2011. Drinking Water Salinity and Maternal Health in Coastal Bangladesh: Implications of Climate Change. Environ Health Perspect. <https://doi.org/10.1289/ehp.1002804>

Khan, A.E., Scheelbeek, P.F.D., Shilpi, A.B., Chan, Q., Mojumder, S.K., Rahman, A., Haines, A., Vineis, P., 2014. Salinity in Drinking Water and the Risk of (Pre)Eclampsia and Gestational Hypertension in Coastal Bangladesh: A Case-Control Study. PLOS ONE 9, e108715. <https://doi.org/10.1371/journal.pone.0108715>

Khan, J.R., Awan, N., Misu, F., 2016. Determinants of anemia among 6-59 months aged children in Bangladesh: evidence from nationally representative data. BMC Pediatr 16, 3. <https://doi.org/10.1186/s12887-015-0536-z>

Khan, M.A., Ghazi, H.O., 2007. Helicobacter pylori infection in asymptomatic subjects in Makkah, Saudi Arabia. J Pak Med Assoc 57, 114–117.

Khan, S.M., Bain, R.E.S., Lunze, K., Unalan, T., Beshanski-Pedersen, B., Slaymaker, T., Johnston, R., Hancioglu, A., 2017. Optimizing household survey methods to monitor the Sustainable Development Goals targets 6.1 and 6.2 on drinking water, sanitation and hygiene: A mixed-methods field-test in Belize. PLOS ONE 12, e0189089. <https://doi.org/10.1371/journal.pone.0189089>

Kheradpisheh, Z., Mirzaei, M., Mahvi, A.H., Mokhtari, M., Azizi, R., Fallahzadeh, H., Ehrampoush, M.H., 2018. Impact of Drinking Water Fluoride on Human Thyroid Hormones: A Case- Control Study. Sci Rep 8, 2674. <https://doi.org/10.1038/s41598-018-20696-4>

Khlifi, R., Olmedo, P., Gil, F., Feki-Tounsi, M., Hammami, B., Rebai, A., Hamza-Chaffai, A., 2014. Biomonitoring of cadmium, chromium, nickel and arsenic in general population living near mining and active industrial areas in Southern Tunisia. Environ Monit Assess 186, 761–779. <https://doi.org/10.1007/s10661-013-3415-9>

Khodarahimi, S., Dehghani, Nikpourian, M.H., 2014. Mental Health and Coping Styles of Rural Residents Affected by Drinking Water Shortage in Fars Province: An Ecopsychological Perspective. European Journal of Mental Health 9, 68–86. <https://doi.org/10.5708/EJMH.9.2014.1.5>

Khoder, G., Muhammad, J.S., Mahmoud, I., Soliman, S.S.M., Burucoa, C., 2019. Prevalence of Helicobacter pylori and Its Associated Factors among Healthy Asymptomatic Residents in the United Arab Emirates. Pathogens 8, E44. <https://doi.org/10.3390/pathogens8020044>

Kihira, T., Yoshida, S., Kondo, Tetsuya, Iwai, K., Wada, S., Morinaga, S., Kazimoto, Y., Kondo, Tomoyoshi, Okamoto, K., Kokubo, Y., Kuzuhara, S., 2012. An increase in ALS incidence on the Kii Peninsula, 1960-2009: a possible link to change in drinking water source. Amyotroph Lateral Scler 13, 347–350. <https://doi.org/10.3109/17482968.2012.674140>

Kikuchi, H., Iwane, S., Munakata, A., Tamura, K., Nakaji, S., Sugawara, K., 1999. Trace element levels in drinking water and the incidence of colorectal cancer. Tohoku J Exp Med 188, 217–225. <https://doi.org/10.1620/tjem.188.217>

Kile, M.L., Baccarelli, A., Hoffman, E., Tarantini, L., Quamruzzaman, Q., Rahman, M., Mahiuddin, G., Mostofa, G., Hsueh, Y.-M., Wright, R.O., Christiani, D.C., 2012. Prenatal arsenic exposure and DNA methylation in maternal and umbilical cord blood leukocytes. Environ Health Perspect 120, 1061–1066. <https://doi.org/10.1289/ehp.1104173>

Kile, M.L., Christiani, D.C., 2008. Environmental Arsenic Exposure and Diabetes. JAMA 300, 845–846. <https://doi.org/10.1001/jama.300.7.845>

Kile, M.L., Faraj, J.M., Ronnenberg, A.G., Quamruzzaman, Q., Rahman, M., Mostofa, G., Afroz, S., Christiani, D.C., 2016. A cross sectional study of anemia and iron deficiency as risk factors for arsenic-induced skin lesions in Bangladeshi women. BMC Public Health 16, 158. <https://doi.org/10.1186/s12889-016-2824-4>

Kile, M.L., Houseman, E.A., Breton, C.V., Quamruzzaman, Q., Rahman, M., Mahiuddin, G., Christiani, D.C., 2007a. Association between total ingested arsenic and toenail arsenic concentrations. J Environ Sci Health A Tox Hazard Subst Environ Eng 42, 1827–1834. <https://doi.org/10.1080/10934520701566819>

Kile, M.L., Houseman, E.A., Breton, C.V., Smith, T., Quamruzzaman, Q., Rahman, M., Mahiuddin, G., Christiani, D.C., 2007b. Dietary arsenic exposure in bangladesh. Environ Health Perspect 115, 889–893. <https://doi.org/10.1289/ehp.9462>

Kile, M.L., Rodrigues, E.G., Mazumdar, M., Dobson, C.B., Diao, N., Golam, M., Quamruzzaman, Q., Rahman, M., Christiani, D.C., 2014. A prospective cohort study of the association between drinking water arsenic exposure and self-reported maternal health symptoms during pregnancy in Bangladesh. Environ Health 13, 29. <https://doi.org/10.1186/1476-069X-13-29>

Kilpatrick, S.J., Safford, K.L., Pomeroy, T., Hoedt, L., Scheerer, L., Laros, R.K., 1991. Maternal hydration increases amniotic fluid index. Obstet Gynecol 78, 1098–1102.

Kim, H., Haltmeier, P., Klotz, J.B., Weisel, C.P., 1999. Evaluation of Biomarkers of Environmental Exposures: Urinary Haloacetic Acids Associated with Ingestion of Chlorinated Drinking Water. Environmental Research 80, 187–195. <https://doi.org/10.1006/enrs.1998.3896>

Kim, J., Yang, Y.J., 2014. Plain water intake of Korean adults according to life style, anthropometric and dietary characteristic: the Korea National Health and Nutrition Examination Surveys 2008-2010. Nutr Res Pract 8, 580–588. <https://doi.org/10.4162/nrp.2014.8.5.580>

Kim, J.I., Kim, G., 2016. Relationship Between the Remaining Years of Healthy Life Expectancy in Older Age and National Income Level, Educational Attainment, and Improved Water Quality. Int J Aging Hum Dev 83, 402–417. <https://doi.org/10.1177/0091415016657560>

Kim, Y.K., Cho, M.H., Hyun, H.S., Park, E., Ha, I.-S., Cheong, H.I., Kang, H.G., 2019. Acute kidney injury associated with Yersinia pseudotuberculosis infection: Forgotten but not gone. Kidney Res Clin Pract 38, 347–355. <https://doi.org/10.23876/j.krcp.19.001>

King, C.C., Chen, C.J., You, S.L., Chuang, Y.C., Huang, H.H., Tsai, W.C., 1989. Community-wide epidemiological investigation of a typhoid outbreak in a rural township in Taiwan, Republic of China. Int J Epidemiol 18, 254–260. <https://doi.org/10.1093/ije/18.1.254>

King, W.D., Marrett, L.D., Woolcott, C.G., 2000. Case-control study of colon and rectal cancers and chlorination by-products in treated water. Cancer Epidemiol Biomarkers Prev 9, 813–818.

Kippler, M., Wagatsuma, Y., Rahman, A., Nermell, B., Persson, L.-Å., Raqib, R., Vahter, M., 2012. Environmental exposure to arsenic and cadmium during pregnancy and fetal size: a longitudinal study in rural Bangladesh. Reprod Toxicol 34, 504–511. <https://doi.org/10.1016/j.reprotox.2012.08.002>

Kirk, A.B., Dyke, J.V., Ohira, S.-I., Dasgupta, P.K., 2013. Relative source contributions for perchlorate exposures in a lactating human cohort. Sci Total Environ 443, 939–943. <https://doi.org/10.1016/j.scitotenv.2012.11.072>

Kleinjans, J.C., Albering, H.J., Marx, A., van Maanen, J.M., van Agen, B., ten Hoor, F., Swaen, G.M., Mertens, P.L., 1991. Nitrate contamination of drinking water: evaluation of genotoxic risk in human populations. Environ Health Perspect 94, 189–193.

Knox, S.S., Jackson, T., Frisbee, S.J., Javins, B., Ducatman, A.M., 2011a. Perfluorocarbon exposure, gender and thyroid function in the C8 Health Project. J Toxicol Sci 36, 403–410. <https://doi.org/10.2131/jts.36.403>

Knox, S.S., Jackson, T., Javins, B., Frisbee, S.J., Shankar, A., Ducatman, A.M., 2011b. Implications of early menopause in women exposed to perfluorocarbons. J Clin Endocrinol Metab 96, 1747–1753. <https://doi.org/10.1210/jc.2010-2401>

Ko, M.K., Yothin Sawangdee, Rossarin Gray, Pojjana Hunchangsith, 2017. Ecological Analysis of Community-Level Socioeconomic Determinants of Infant and Under-Five Mortality in Myanmar: an Analysis of the 2014 Myanmar Population and Housing Census. Journal of Health Research 31, 1. <https://doi.org/10.14456/JHR.2017.8>

Kobayashi, E., Suwazono, Y., Dochi, M., Honda, R., Kido, T., 2009a. Association of lifetime cadmium intake or drinking Jinzu River water with the occurrence of renal tubular dysfunction. Environmental Toxicology 24, 421–428. <http://dx.doi.org/10.1002/tox.20444>

Kobayashi, E., Suwazono, Y., Dochi, M., Honda, R., Kido, T., 2009b. Estimation of Benchmark Rice Cadmium Doses as Threshold Values for Abnormal Urinary Findings with Adjustment for Consumption of Jinzu River Water. Bull Environ Contam Toxicol 83, 102–107. <https://doi.org/10.1007/s00128-009-9766-9>

Kobayashi, E., Suwazono, Y., Dochi, M., Honda, R., Kido, T., 2009c. Influence of consumption of cadmium-polluted rice or Jinzu River water on occurrence of renal tubular dysfunction and/or Itai-itai disease. Biol Trace Elem Res 127, 257–268. <https://doi.org/10.1007/s12011-008-8239-z>

Kocher, D.C., Hoffman, F.O., 2011. Drinking water standard for tritium-what’s the risk? Health Phys 101, 274–285. <https://doi.org/10.1097/HP.0b013e31820ff161>

Kodjebacheva, G.D., Lapeyrouse, L.M., Rahrig, J., Walker, L., Campbell, K., 2019. Strategies to Promote Safe Water Drinking Identified by and for Women of Reproductive Age. Am J Health Promot 33, 756–759. <https://doi.org/10.1177/0890117118820172>

Koivusalo, M., Hakulinen, T., Vartiainen, T., Pukkala, E., Jaakkola, J.J., Tuomisto, J., 1998. Drinking water mutagenicity and urinary tract cancers: a population-based case-control study in Finland. Am J Epidemiol 148, 704–712. <https://doi.org/10.1093/aje/148.7.704>

Koivusalo, M., Pukkala, E., Vartiainen, T., Jaakkola, J.J., Hakulinen, T., 1997. Drinking water chlorination and cancer-a historical cohort study in Finland. Cancer Causes Control 8, 192–200. <https://doi.org/10.1023/a:1018420229802>

Koivusalo, M., Vartiainen, T., Hakulinen, T., Pukkala, E., Jaakkola, J.J., 1995. Drinking water mutagenicity and leukemia, lymphomas, and cancers of the liver, pancreas, and soft tissue. Arch Environ Health 50, 269–276. <https://doi.org/10.1080/00039896.1995.9935953>

Komarulzaman, A., de Jong, E., Smits, J., 2019. Effects of water and health on primary school enrolment and absenteeism in Indonesia. J Water Health 17, 633–646. <https://doi.org/10.2166/wh.2019.044>

Kondakis, X.G., Makris, N., Leotsinidis, M., Prinou, M., Papapetropoulos, T., 1989. Possible health effects of high manganese concentration in drinking water. Arch Environ Health 44, 175–178. <https://doi.org/10.1080/00039896.1989.9935883>

Konthonbut, P., Kongtip, P., Nankongnab, N., Tipayamongkholgul, M., Yoosook, W., Woskie, S., 2018. Paraquat Exposure of Pregnant Women and Neonates in Agricultural Areas in Thailand. IJERPH 15, 1163. <https://doi.org/10.3390/ijerph15061163>

Kookana, R.S., Maheshwari, B., Dillon, P., Dave, S.H., Soni, P., Bohra, H., Dashora, Y., Purohit, R.C., Ward, J., Oza, S., Katara, P., Yadav, K.K., Varua, M.E., Grewal, H.S., Packham, R., Jodha, A.S., Patel, A., 2016. Groundwater scarcity impact on inclusiveness and women empowerment: Insights from school absenteeism of female students in two watersheds in India. International Journal of Inclusive Education 20, 1155–1171. <https://doi.org/10.1080/13603116.2016.1155664>

Kousa, A., Havulinna, A.S., Moltchanova, E., Taskinen, O., Nikkarinen, M., Salomaa, V., Karvonen, M., 2008. Magnesium in well water and the spatial variation of acute myocardial infarction incidence in rural Finland. Applied Geochemistry 23, 632–640. <https://doi.org/10.1016/j.apgeochem.2007.10.015>

Kousa, A., Puustinen, N., Karvonen, M., Moltchanova, E., 2012. The regional association of rising type 2 diabetes incidence with magnesium in drinking water among young adults. Environ Res 112, 126–128. <https://doi.org/10.1016/j.envres.2011.11.001>

Kramer, M.D., Lynch, C.F., Isacson, P., Hanson, J.W., 1992. The association of waterborne chloroform with intrauterine growth retardation. Epidemiology 3, 407–413. <https://doi.org/10.1097/00001648-199209000-00005>

Kröger, H., Alhava, E., Honkanen, R., Tuppurainen, M., Saarikoski, S., 1994. The effect of fluoridated drinking water on axial bone mineral density — a population-based study. Bone and Mineral 27, 33–41. <https://doi.org/10.1016/S0169-6009(08)80184-5>

Kullar, S.S., Shao, K., Surette, C., Foucher, D., Mergler, D., Cormier, P., Bellinger, D.C., Barbeau, B., Sauvé, S., Bouchard, M.F., 2019. A benchmark concentration analysis for manganese in drinking water and IQ deficits in children. Environ Int 130, 104889. <https://doi.org/10.1016/j.envint.2019.05.083>

Kulmala, T., Vaahtera, M., Ndekha, M., Cullinan, T., Salin, M.-L., Koivisto, A.-M., 2009. Socio-Economic support for good health in rural Malawi. E Af Med Jrnl 77. <https://doi.org/10.4314/eamj.v77i3.46616>

Kumar, M., Patel, A.K., Das, A., Kumar, P., Goswami, R., Deka, P., Das, N., 2017. Hydrogeochemical controls on mobilization of arsenic and associated health risk in Nagaon district of the central Brahmaputra Plain, India. Environ Geochem Health 39, 161–178. <https://doi.org/10.1007/s10653-016-9816-2>

Kumar, S., Forand, S., Babcock, G., Richter, W., Hart, T., Hwang, S.-A., 2014. Total trihalomethanes in public drinking water supply and birth outcomes: a cross-sectional study. Matern Child Health J 18, 996–1006. <https://doi.org/10.1007/s10995-013-1328-4>

Kumar, S., Singh, R., Venkatesh, A.S., Udayabhanu, G., Sahoo, P.R., 2019. Medical Geological assessment of fluoride contaminated groundwater in parts of Indo-Gangetic Alluvial plains. Sci Rep 9, 16243. <https://doi.org/10.1038/s41598-019-52812-3>

Kumar, T., 2017. Vibrio Cholerae Outbreak in Batala Town, Punjab, India 2012. Journal of Communicable Diseases 49, 35–40. <https://doi.org/10.24321/0019.5138.201705>

Kuo, H.-W., Chen, P.-S., Ho, S.-C., Wang, L.-Y., Yang, C.-Y., 2010a. Trihalomethanes in drinking water and the risk of death from rectal cancer: does hardness in drinking water matter? J Toxicol Environ Health A 73, 807–818. <https://doi.org/10.1080/15287391003689267>

Kuo, H.-W., Peng, C.-Y., Feng, A., Wu, T.-N., Yang, C.-Y., 2011. Magnesium in drinking water modifies the association between trihalomethanes and the risk of death from colon cancer. J Toxicol Environ Health A 74, 392–403. <https://doi.org/10.1080/15287394.2011.538836>

Kuo, H.-W., Tiao, M.-M., Tsai, S.-S., Wu, T.-N., Yang, C.-Y., 2010b. Does calcium in drinking water modify the association between trihalomethanes and the risk of death from colon cancer? J Toxicol Environ Health A 73, 657–668. <https://doi.org/10.1080/15287390903578513>

Kuo, H.-W., Tiao, M.-M., Wu, T.-N., Yang, C.-Y., 2009. Trihalomethanes in Drinking Water and the Risk of Death from Colon Cancer in Taiwan. Journal of Toxicology and Environmental Health, Part A 72, 1217–1222. <https://doi.org/10.1080/15287390903129176>

Kuo, H.-W., Wu, T.-N., Yang, C.-Y., 2007. Nitrates in drinking water and risk of death from rectal cancer in Taiwan. J Toxicol Environ Health A 70, 1717–1722. <https://doi.org/10.1080/15287390701457704>

Kuo, Y.-C., Lo, Y.-S., Guo, H.-R., 2017. Lung Cancer Associated with Arsenic Ingestion: Cell-type Specificity and Dose Response. Epidemiology 28 Suppl 1, S106–S112. <https://doi.org/10.1097/EDE.0000000000000743>

Kuok, K.K., Chiu, P.C., 2018. Indigenous drinking-water consumption pattern of residents in Kuching city: results of a pilot study. Journal of Water, Sanitation and Hygiene for Development 8, 817–824. <https://doi.org/10.2166/washdev.2018.004>

Kurttio, P., Gustavsson, N., Vartiainen, T., Pekkanen, J., 1999. Exposure to natural fluoride in well water and hip fracture: a cohort analysis in Finland. Am J Epidemiol 150, 817–824. <https://doi.org/10.1093/oxfordjournals.aje.a010086>

Kurttio, P., Harmoinen, A., Saha, H., Salonen, L., Karpas, Z., Komulainen, H., Auvinen, A., 2006. Kidney toxicity of ingested uranium from drinking water. Am J Kidney Dis 47, 972–982. <https://doi.org/10.1053/j.ajkd.2006.03.002>

Kurttio, P., Komulainen, H., Leino, A., Salonen, L., Auvinen, A., Saha, H., 2005. Bone as a Possible Target of Chemical Toxicity of Natural Uranium in Drinking Water. Environ Health Perspect 113, 68–72. <https://doi.org/10.1289/ehp.7475>

Kuusi, M., Aavitsland, P., Gondrosen, B., Kapperud, G., 2003. Incidence of gastroenteritis in Norway – a population-based survey. Epidemiol. Infect. 131, 591–597. <https://doi.org/10.1017/S0950268803008744>

Kwami, C.S., Godfrey, S., Gavilan, H., Lakhanpaul, M., Parikh, P., 2019. Water, Sanitation, and Hygiene: Linkages with Stunting in Rural Ethiopia. Int J Environ Res Public Health 16, E3793. <https://doi.org/10.3390/ijerph16203793>

Kwok, R.K., Kaufmann, R.B., Jakariya, M., 2006. Arsenic in drinking-water and reproductive health outcomes: a study of participants in the Bangladesh Integrated Nutrition Programme. J Health Popul Nutr 24, 190–205.

Kwok, R.K., Mendola, P., Liu, Z.Y., Savitz, D.A., Heiss, G., Ling, H.L., Xia, Y., Lobdell, D., Zeng, D., Thorp, J.M., Creason, J.P., Mumford, J.L., 2007. Drinking water arsenic exposure and blood pressure in healthy women of reproductive age in Inner Mongolia, China. Toxicol Appl Pharmacol 222, 337–343. <https://doi.org/10.1016/j.taap.2007.04.003>

Kwong, R.C., Karagas, M.R., Kelsey, K.T., Mason, R.A., Tanyos, S.A., Schned, A.R., Marsit, C.J., Andrew, A.S., 2010. Arsenic exposure predicts bladder cancer survival in a US population. World J Urol 28, 487–492. <https://doi.org/10.1007/s00345-009-0477-y>

Labrique, A.B., Zaman, K., Hossain, Z., Saha, P., Yunus, M., Hossain, A., Ticehurst, J., Kmush, B., Nelson, K.E., 2013. An Exploratory Case Control Study of Risk Factors for Hepatitis E in Rural Bangladesh. PLoS ONE 8, e61351. <https://doi.org/10.1371/journal.pone.0061351>

Lagare, A., Ibrahim, A., Ousmane, S., Issaka, B., Zaneidou, M., Kadadé, G., Testa, J., 2018. Outbreak of Hepatitis E Virus Infection in Displaced Persons Camps in Diffa Region, Niger, 2017. Am J Trop Med Hyg 99, 1055–1057. <https://doi.org/10.4269/ajtmh.17-0950>

Laine, J.E., Bailey, K.A., Rubio-Andrade, M., Olshan, A.F., Smeester, L., Drobná, Z., Herring, A.H., Stýblo, M., García-Vargas, G.G., Fry, R.C., 2015. Maternal Arsenic Exposure, Arsenic Methylation Efficiency, and Birth Outcomes in the Biomarkers of Exposure to ARsenic (BEAR) Pregnancy Cohort in Mexico. Environ Health Perspect 123, 186–192. <https://doi.org/10.1289/ehp.1307476>

Laine, J.E., Ilievski, V., Richardson, D.B., Herring, A.H., Stýblo, M., Rubio-Andrade, M., Garcia-Vargas, G., Gamble, M.V., Fry, R.C., 2018. Maternal one carbon metabolism and arsenic methylation in a pregnancy cohort in Mexico. Journal of Exposure Science and Environmental Epidemiology 28, 505–514. <https://doi.org/10.1038/s41370-018-0041-1>

LaKind, J.S., Naiman, D.Q., Hays, S.M., Aylward, L.L., Blount, B.C., 2010. Public health interpretation of trihalomethane blood levels in the United States: NHANES 1999-2004. J Expo Sci Environ Epidemiol 20, 255–262. <https://doi.org/10.1038/jes.2009.35>

Lamm, S.H., Boroje, I.J., Ferdosi, H., Ahn, J., 2018. Lung Cancer Risk and Low (≤50 μg/L) Drinking Water Arsenic Levels for US Counties (2009–2013)—A Negative Association. Int J Environ Res Public Health 15, 1200. <https://doi.org/10.3390/ijerph15061200>

Lan, C.-F., Lin, I.-F., Wang, S.-J., 1995. Fluoride in Drinking Water and the Bone Mineral Density of Women in Taiwan. Int J Epidemiol 24, 1182–1187. <https://doi.org/10.1093/ije/24.6.1182>

Lardner, D., Passafaro, M., Gotimer, K.F., Guernsey, D., Padgett, C., Sundar, S., 2015. Regional water use practices in the Kwahu East district of Ghana and the potential influence of diarrheal disease. European Scientific Journal, ESJ.

Lawrence, C.E., Taylor, P.R., Trock, B.J., Reilly, A.A., 1984. Trihalomethanes in drinking water and human colorectal cancer. J Natl Cancer Inst 72, 563–568.

Lee, H.-S., Park, S., Kim, M.-H., 2014. Factors associated with low water intake among South Korean adolescents - Korea National Health and Nutrition Examination Survey, 2007-2010. Nutr Res Pract 8, 74–80. <https://doi.org/10.4162/nrp.2014.8.1.74>

Lee, J.-J., Jang, C.-S., Wang, S.-W., Liu, C.-W., 2007. Evaluation of potential health risk of arsenic-affected groundwater using indicator kriging and dose response model. Sci Total Environ 384, 151–162. <https://doi.org/10.1016/j.scitotenv.2007.06.021>

Lee, Y.-H., Jeong, H.G., Kong, W.H., Lee, S.-H., Cho, H.-I., Nam, H.-S., Ismail, H.A.H.A., Alla, G.N.A., Oh, C.H., Hong, S.-T., 2015. Reduction of Urogenital Schistosomiasis with an Integrated Control Project in Sudan. PLoS Negl Trop Dis 9, e3423. <https://doi.org/10.1371/journal.pntd.0003423>

Lehmann, R., Wapniarz, M., Hofmann, B., Pieper, B., Haubitz, I., Allolio, B., 1998. Drinking water fluoridation: Bone mineral density and hip fracture incidence. Bone 22, 273–278. <https://doi.org/10.1016/S8756-3282(97)00273-1>

Leung, W.K., Lin, S.-R., Ching, J.Y.L., To, K.-F., Ng, E.K.W., Chan, F.K.L., Lau, J.Y.W., Sung, J.J.Y., 2004. Factors predicting progression of gastric intestinal metaplasia: results of a randomised trial on Helicobacter pylori eradication. Gut 53, 1244–1249. <https://doi.org/10.1136/gut.2003.034629>

Leurs, L.J., Schouten, L.J., Mons, M.N., Goldbohm, R.A., van den Brandt, P.A., 2010. Relationship between Tap Water Hardness, Magnesium, and Calcium Concentration and Mortality due to Ischemic Heart Disease or Stroke in the Netherlands. Environmental Health Perspectives 118, 414–420. <https://doi.org/10.1289/ehp.0900782>

Levy, M., Leclerc, B.-S., 2012. Fluoride in drinking water and osteosarcoma incidence rates in the continental United States among children and adolescents. Cancer Epidemiol 36, e83-88. <https://doi.org/10.1016/j.canep.2011.11.008>

Levy, S.M., Warren, J.J., Phipps, K., Letuchy, E., Broffitt, B., Eichenberger-Gilmore, J., Burns, T.L., Kavand, G., Janz, K.F., Torner, J.C., Pauley, C.A., 2014. Effects of life-long fluoride intake on bone measures of adolescents: a prospective cohort study. J Dent Res 93, 353–359. <https://doi.org/10.1177/0022034514520708>

Lewandowski, T.A., Peterson, M.K., Charnley, G., 2015. Iodine supplementation and drinking-water perchlorate mitigation. Food Chem Toxicol 80, 261–270. <https://doi.org/10.1016/j.fct.2015.03.014>

Lewis, C., Suffet, I.H., Hoggatt, K., Ritz, B., 2007. Estimated effects of disinfection by-products on preterm birth in a population served by a single water utility. Environ Health Perspect 115, 290–295. <https://doi.org/10.1289/ehp.9394>

Lewis, C., Suffet, I.H., Ritz, B., 2006. Estimated effects of disinfection by-products on birth weight in a population served by a single water utility. Am J Epidemiol 163, 38–47. <https://doi.org/10.1093/aje/kwj009>

Lewis, D.R., Southwick, J.W., Ouellet-Hellstrom, R., Rench, J., Calderon, R.L., 1999. Drinking water arsenic in Utah: A cohort mortality study. Environ Health Perspect 107, 359–365. <https://doi.org/10.1289/ehp.99107359>

Li, H., Engström, K., Vahter, M., Broberg, K., 2012. Arsenic exposure through drinking water is associated with longer telomeres in peripheral blood. Chem Res Toxicol 25, 2333–2339. <https://doi.org/10.1021/tx300222t>

Li, H., Liu, Q., Wang, W., Yang, L., Li, Y., Feng, F., Zhao, X., Hou, K., Wang, G., 2009. Fluoride in drinking water, brick tea infusion and human urine in two counties in Inner Mongolia, China. J Hazard Mater 167, 892–895. <https://doi.org/10.1016/j.jhazmat.2009.01.094>

Li, J., Zhao, H., Xia, W., Zhou, Y., Xu, S., Cai, Z., 2019. Nine phthalate metabolites in human urine for the comparison of health risk between population groups with different water consumptions. Science of The Total Environment 649, 1532–1540. <https://doi.org/10.1016/j.scitotenv.2018.08.294>

Li, J.Y., Ershow, A.G., Chen, Z.J., Wacholder, S., Li, G.Y., Guo, W., Li, B., Blot, W.J., 1989. A case-control study of cancer of the esophagus and gastric cardia in Linxian. Int J Cancer 43, 755–761. <https://doi.org/10.1002/ijc.2910430502>

Li, N., Ho, W., Wu, R.S.S., Tsang, E.P.K., Ying, G.-G., Deng, W.-J., 2019. Ultra violet filters in the urine of preschool children and drinking water. Environment International 133, 105246. <https://doi.org/10.1016/j.envint.2019.105246>

Li, P., Li, X., Meng, X., Li, M., Zhang, Y., 2016. Appraising Groundwater Quality and Health Risks from Contamination in a Semiarid Region of Northwest China. Expo Health 8, 361–379. <https://doi.org/10.1007/s12403-016-0205-y>

Li, Q., Yu, Y., Wang, F., Chen, S., Yin, Y., Lin, H., Che, F., Sun, P., Qin, J., Liu, J., Wang, H., 2014. Urinary perchlorate exposure and risk in women of reproductive age in a fireworks production area of China. Arch Environ Contam Toxicol 67, 42–49. <https://doi.org/10.1007/s00244-014-0042-6>

Li, W., Liu, E., BeLue, R., 2018. Household water treatment and the nutritional status of primary-aged children in India: findings from the India human development survey. Global Health 14, 37. <https://doi.org/10.1186/s12992-018-0356-7>

Li, X., Ying, G.-G., Zhao, J.-L., Chen, Z.-F., Lai, H.-J., Su, H.-C., 2013. 4-Nonylphenol, bisphenol-A and triclosan levels in human urine of children and students in China, and the effects of drinking these bottled materials on the levels. Environ Int 52, 81–86. <https://doi.org/10.1016/j.envint.2011.03.026>

Li, Y., Fletcher, T., Mucs, D., Scott, K., Lindh, C.H., Tallving, P., Jakobsson, K., 2018. Half-lives of PFOS, PFHxS and PFOA after end of exposure to contaminated drinking water. Occup Environ Med 75, 46–51. <https://doi.org/10.1136/oemed-2017-104651>

Li, Y., Liang, C., Slemenda, C.W., Ji, R., Sun, S., Cao, J., Emsley, C.L., Ma, F., Wu, Y., Ying, P., Zhang, Y., Gao, S., Zhang, W., Katz, B.P., Niu, S., Cao, S., Johnston, C.C., 2001. Effect of long-term exposure to fluoride in drinking water on risks of bone fractures. J Bone Miner Res 16, 932–939. <https://doi.org/10.1359/jbmr.2001.16.5.932>

Li, Z., Karp, H., Zerlin, A., Lee, T.Y.A., Carpenter, C., Heber, D., 2010. Absorption of silicon from artesian aquifer water and its impact on bone health in postmenopausal women: a 12 week pilot study. Nutr J 9, 44. <https://doi.org/10.1186/1475-2891-9-44>

Li, Z., Yang, K., Xie, C., Yang, Q., Lei, X., Wang, H., 2021. Assessment of potential health risk of major contaminants of groundwater in a densely populated agricultural area. Environ Geochem Health 43, 663–682. <https://doi.org/10.1007/s10653-019-00470-9>

Liao, C.-M., Lin, T.-L., Chen, S.-C., 2007. A Weibull-PBPK model for assessing risk of arsenic-induced skin lesions in children. Sci Total Environ 392, 203–217. <https://doi.org/10.1016/j.scitotenv.2007.12.017>

Liao, Y.-H., Chen, P.-S., Chiu, H.-F., Yang, C.-Y., 2013. Magnesium in drinking water modifies the association between nitrate ingestion and risk of death from esophageal cancer. J Toxicol Environ Health A 76, 192–200. <https://doi.org/10.1080/15287394.2013.752324>

Liaugaudaite, V., Mickuviene, N., Raskauskiene, N., Naginiene, R., Sher, L., 2017. Lithium levels in the public drinking water supply and risk of suicide: A pilot study. J Trace Elem Med Biol 43, 197–201. <https://doi.org/10.1016/j.jtemb.2017.03.009>

Liaw, J., Marshall, G., Yuan, Y., Ferreccio, C., Steinmaus, C., Smith, A.H., 2008. Increased childhood liver cancer mortality and arsenic in drinking water in northern Chile. Cancer Epidemiol Biomarkers Prev 17, 1982–1987. <https://doi.org/10.1158/1055-9965.EPI-07-2816>

Lim, W.H., Wong, G., Lewis, J.R., Lok, C.E., Polkinghorne, K.R., Hodgson, J., Lim, E.M., Prince, R.L., 2017. Total volume and composition of fluid intake and mortality in older women: a cohort study. BMJ Open 7, e011720. <https://doi.org/10.1136/bmjopen-2016-011720>

Lin, D.-B., Lin, J.-B., Chen, C.-Y., Chen, S.-C., Chen, W.-K., 2007. Seroprevalence of Helicobacter pylori infection among schoolchildren and teachers in Taiwan. Helicobacter 12, 258–264. <https://doi.org/10.1111/j.1523-5378.2007.00496.x>

Lin, H.-J., Sung, T.-I., Chen, C.-Y., Guo, H.-R., 2013. Arsenic levels in drinking water and mortality of liver cancer in Taiwan. J Hazard Mater 262, 1132–1138. <https://doi.org/10.1016/j.jhazmat.2012.12.049>

Lin, P.-I.D., Bromage, S., Mostofa, M.G., Allen, J., Oken, E., Kile, M.L., Christiani, D.C., 2017. Associations between Diet and Toenail Arsenic Concentration among Pregnant Women in Bangladesh: A Prospective Study. Nutrients 9, E420. <https://doi.org/10.3390/nu9040420>

Lin, Y.-L., Liao, Y.-S., Liao, L.-R., Chen, F.-N., Kuo, H.-M., He, S., 2008. Seroprevalence and sources of Toxoplasma infection among indigenous and immigrant pregnant women in Taiwan. Parasitol Res 103, 67–74. <https://doi.org/10.1007/s00436-008-0928-1>

Lindberg, A.-L., Ekström, E.-C., Nermell, B., Rahman, M., Lönnerdal, B., Persson, L.-A., Vahter, M., 2008a. Gender and age differences in the metabolism of inorganic arsenic in a highly exposed population in Bangladesh. Environ Res 106, 110–120. <https://doi.org/10.1016/j.envres.2007.08.011>

Lindberg, A.-L., Rahman, M., Persson, L.-A., Vahter, M., 2008b. The risk of arsenic induced skin lesions in Bangladeshi men and women is affected by arsenic metabolism and the age at first exposure. Toxicol Appl Pharmacol 230, 9–16. <https://doi.org/10.1016/j.taap.2008.02.001>

Lindberg, A.-L., Sohel, N., Rahman, M., Persson, L.Å., Vahter, M., 2010. Impact of Smoking and Chewing Tobacco on Arsenic-Induced Skin Lesions. Environmental Health Perspectives 118, 533–538. <https://doi.org/10.1289/ehp.0900728>

Linos, A., Petralias, A., Christophi, C.A., Christoforidou, E., Kouroutou, P., Stoltidis, M., Veloudaki, A., Tzala, E., Makris, K.C., Karagas, M.R., 2011. Oral ingestion of hexavalent chromium through drinking water and cancer mortality in an industrial area of Greece--an ecological study. Environ Health 10, 50. <https://doi.org/10.1186/1476-069X-10-50>

Liou, S.-H., Wu, T.-N., Chiang, H.-C., Yang, G.-Y., Wu, Y.-Q., Lai, J.-S., Ho, S.-T., Guo, Y.-L., Ko, Y.-C., Chang, P.-Y., 1994. Blood lead levels in the general population of Taiwan, Republic of China. Int. Arch Occup Environ Heath 66, 255–260. <https://doi.org/10.1007/BF00454364>

Liou, S.H., Wu, T.N., Chiang, H.C., Yang, G.Y., Yang, T., Wu, Y.Q., Lai, J.S., Ho, S.T., Lee, C.C., Ko, Y.C., Ko, K.N., Chang, P.Y., 1996. Blood lead levels in Taiwanese adults: distribution and influencing factors. Sci Total Environ 180, 211–219. <https://doi.org/10.1016/0048-9697(96)80245-2>

Liou, S.-H., Wu, T.-N., Chiang, H.-C., Yang, T., Yang, G.-Y., Wu, Y.-Q., Lai, J.-S., Ho, S.-T., Guo, Y.-L., Ko, Y.-C., Ko, K.-N., Chang, P.-Y., 1996. Three-year survey of blood lead levels in 8828 Taiwanese adults. Int. Arch Occup Environ Heath 68, 80–87. <https://doi.org/10.1007/BF00381239>

Liou, S.-H., Yang, G.C.C., Wang, C.-L., Chiu, Y.-H., 2014. Monitoring of PAEMs and beta-agonists in urine for a small group of experimental subjects and PAEs and beta-agonists in drinking water consumed by the same subjects. J Hazard Mater 277, 169–179. <https://doi.org/10.1016/j.jhazmat.2014.02.024>

Liu, E., Balasubramaniam, D., Hunt, A.F., 2016. Does access to water matter? A study on the nutritional status of primary-aged children in India. Journal of Public Health 38, e419–e424. <https://doi.org/10.1093/pubmed/fdv149>

Liu, J., Gao, Y., Liu, H., Sun, J., Liu, Y., Wu, J., Li, D., Sun, D., 2017. Assessment of relationship on excess arsenic intake from drinking water and cognitive impairment in adults and elders in arsenicosis areas. Int J Hyg Environ Health 220, 424–430. <https://doi.org/10.1016/j.ijheh.2016.12.004>

Liu, L., Liu, J., Wang, D., Shen, H., Jia, Q., 2020. Effect of Urinary Iodine Concentration in Pregnant and Lactating Women, and in Their Infants Residing in Areas with Excessive Iodine in Drinking Water in Shanxi Province, China. Biol Trace Elem Res 193, 326–333. <https://doi.org/10.1007/s12011-019-01716-4>

Liu, Ling, Wang, M., Li, Y., Liu, H., Hou, C., Zeng, Q., Li, P., Zhao, Q., Dong, L., Yu, X., Liu, Li, Zhang, S., Wang, A., 2019. Low-to-moderate fluoride exposure in relation to overweight and obesity among school-age children in China. Ecotoxicol Environ Saf 183, 109558. <https://doi.org/10.1016/j.ecoenv.2019.109558>

Liu, P., Liu, L., Shen, H., Jia, Q., Wang, J., Zheng, H., Ma, J., Zhou, D., Liu, S., Su, X., 2014. The Standard, Intervention Measures and Health Risk for High Water Iodine Areas. PLOS ONE 9, e89608. <https://doi.org/10.1371/journal.pone.0089608>

Liu, S., Zhu, Z., Fan, C., Qiu, Y., Zhao, J., 2011. Seasonal variation effects on the formation of trihalomethane during chlorination of water from Yangtze River and associated cancer risk assessment. J Environ Sci (China) 23, 1503–1511. <https://doi.org/10.1016/s1001-0742(10)60573-6>

Ljung, K.S., Kippler, M.J., Goessler, W., Grandér, G.M., Nermell, B.M., Vahter, M.E., 2009. Maternal and Early Life Exposure to Manganese in Rural Bangladesh. Environ. Sci. Technol. 43, 2595–2601. <https://doi.org/10.1021/es803143z>

Llopis-González, A., Marti Requena, P., Sagrado-Vives, Gimeno-Clemente, Monforte-Monleón, Yusà-Pelecha, Morales Suárez-Varela, M., 2011. Ecological Study on Digestive and Bladder Cancer in Relation to the Level of Trihalomethanes in Drinking Water. International Journal of Environmental Research 5, 613–620.

Loffredo, C.A., Aposhian, H.V., Cebrian, M.E., Yamauchi, H., Silbergeld, E.K., 2003. Variability in human metabolism of arsenic. Environ Res 92, 85–91. <https://doi.org/10.1016/s0013-9351(02)00081-6>

Longstreet, D.A., Heath, D.L., Panaretto, K.S., Vink, R., 2007. Correlations suggest low magnesium may lead to higher rates of type 2 diabetes in Indigenous Australians. Rural Remote Health 7, 843.

Lopez, B., Jones, J.L., Mury, M.A., Wilson, M., Klein, R., Luby, S., Maguire, J.H., 2005. Toxoplasma Gondii infection in rural Guatemalan children. The American Journal of Tropical Medicine and Hygiene 72, 295–300. <https://doi.org/10.4269/ajtmh.2005.72.295>

Lopez-Espinosa, M.-J., Fletcher, T., Armstrong, B., Genser, B., Dhatariya, K., Mondal, D., Ducatman, A., Leonardi, G., 2011. Association of Perfluorooctanoic Acid (PFOA) and Perfluorooctane Sulfonate (PFOS) with age of puberty among children living near a chemical plant. Environ Sci Technol 45, 8160–8166. <https://doi.org/10.1021/es1038694>

Luben, T.J., Nuckols, J.R., Mosley, B.S., Hobbs, C., Reif, J.S., 2008. Maternal exposure to water disinfection by-products during gestation and risk of hypospadias. Occup Environ Med 65, 420–429. <https://doi.org/10.1136/oem.2007.034256>

Lucchini, R.G., Guazzetti, S., Zoni, S., Donna, F., Peter, S., Zacco, A., Salmistraro, M., Bontempi, E., Zimmerman, N.J., Smith, D.R., 2012. Tremor, olfactory and motor changes in Italian adolescents exposed to historical ferro-manganese emission. Neurotoxicology 33, 687–696. <https://doi.org/10.1016/j.neuro.2012.01.005>

Lumen, A., George, N.I., 2017. Evaluation of the risk of perchlorate exposure in a population of late-gestation pregnant women in the United States: Application of probabilistic biologically-based dose response modeling. Toxicol Appl Pharmacol 322, 9–14. <https://doi.org/10.1016/j.taap.2017.02.021>

Lv, S., Xie, L., Xu, D., Wang, Y., Jia, L., Du, Y., 2016. Effect of reducing iodine excess on children’s goiter prevalence in areas with high iodine in drinking water. Endocrine 52, 296–304. <https://doi.org/10.1007/s12020-015-0742-3>

Lv, S., Xu, D., Wang, Y., Jun, Z., Jia, L., Du, Y., 2015. Impact of removing iodised salt on children’s goitre status in areas with excessive iodine in drinking-water. Br J Nutr 113, 114–119. <https://doi.org/10.1017/S0007114514003365>

Lv, S., Zhao, J., Xu, D., Chong, Zhenshui, Chong, Zhengshui, Jia, L., Du, Y., Ma, J., Rutherford, S., 2012. An epidemiological survey of children’s iodine nutrition and goitre status in regions with mildly excessive iodine in drinking water in Hebei Province, China. Public Health Nutr 15, 1168–1173. <https://doi.org/10.1017/S1368980012000146>

Mabasa, E., Mabapa, N., Jooste, P., Mbhenyane, X., 2019. Iodine status of pregnant women and children age 6 to 12 years feeding from the same food basket in Mopani district, Limpopo province, South Africa. South African Journal of Clinical Nutrition 32, 76–82. <https://doi.org/10.1080/16070658.2018.1449370>

MacLehose, R.F., Savitz, D.A., Herring, A.H., Hartmann, K.E., Singer, P.C., Weinberg, H.S., 2008. Drinking water disinfection by-products and time to pregnancy. Epidemiology 19, 451–458. <https://doi.org/10.1097/EDE.0b013e31816a23eb>

Mahalingaiah, S., Winter, M.R., Aschengrau, A., 2016. Association of prenatal and early life exposure to tetrachloroethylene (PCE) with polycystic ovary syndrome and other reproductive disorders in the cape cod health study: A retrospective cohort study. Reprod Toxicol 65, 87–94. <https://doi.org/10.1016/j.reprotox.2016.07.005>

Maharjan, M., Shrestha, R.R., Ahmad, S.A., Watanabe, C., Ohtsuka, R., 2006. Prevalence of arsenicosis in terai, Nepal. J Health Popul Nutr 24, 246–252.

Maharjan, M., Watanabe, C., Ahmad, S.A., Ohtsuka, R., 2005. Arsenic contamination in drinking water and skin manifestations in lowland Nepal: the first community-based survey. Am J Trop Med Hyg 73, 477–479.

Maharjan, M., Watanabe, C., Ahmad, S.A., Umezaki, M., Ohtsuka, R., 2007. Mutual interaction between nutritional status and chronic arsenic toxicity due to groundwater contamination in an area of Terai, lowland Nepal. J Epidemiol Community Health 61, 389–394. <https://doi.org/10.1136/jech.2005.045062>

Maheswaran, R., Morris, S., Falconer, S., Grossinho, A., Perry, I., Wakefield, J., Elliott, P., 1999. Magnesium in drinking water supplies and mortality from acute myocardial infarction in north west England. Heart 82, 455–460. <https://doi.org/10.1136/hrt.82.4.455>

Mahmud, M.A., Chappell, C., Hossain, M.M., Habib, M., Dupont, H.L., 1995. Risk factors for development of first symptomatic Giardia infection among infants of a birth cohort in rural Egypt. Am J Trop Med Hyg 53, 84–88.

Mahoney, M.C., Nasca, P.C., Burnett, W.S., Melius, J.M., 1991. Bone cancer incidence rates in New York State: time trends and fluoridated drinking water. Am J Public Health 81, 475–479.

Makris, Konstantinos C, Andra, S.S., Herrick, L., Christophi, C.A., Snyder, S.A., Hauser, R., 2013. Association of drinking-water source and use characteristics with urinary antimony concentrations. J Expo Sci Environ Epidemiol 23, 120–127. <https://doi.org/10.1038/jes.2012.104>

Makris, K. C., Andra, S.S., Jia, A., Herrick, L., Christophi, C.A., Snyder, S.A., Hauser, R., 2013. Association between water consumption from polycarbonate containers and bisphenol A intake during harsh environmental conditions in summer. Environ Sci Technol 47, 3333–3343. <https://doi.org/10.1021/es304038k>

Mallin, K., 1990. Investigation of a bladder cancer cluster in northwestern Illinois. Am J Epidemiol 132, S96-106. <https://doi.org/10.1093/oxfordjournals.aje.a115795>

Manassaram, D.M., Backer, L.C., Messing, R., Fleming, L.E., Luke, B., Monteilh, C.P., 2010. Nitrates in drinking water and methemoglobin levels in pregnancy: a longitudinal study. Environ Health 9, 60. <https://doi.org/10.1186/1476-069X-9-60>

Mao, G., Ding, G., Lou, X., Zhang, R., Zheng, P., Mo, Z., Wang, X., Zhu, W., Zhou, J., Gu, F., 2015. Survey of iodine nutritional status in 2011, Zhejiang, China. Asia Pac J Clin Nutr 24, 234–244. <https://doi.org/10.6133/apjcn.2015.24.2.08>

Maraver, F., Vitoria, I., Ferreira-Pêgo, C., Armijo, F., Salas-Salvadó, J., 2015. Magnesium in tap and bottled mineral water in Spain and its contribution to nutritional recommendations. Nutr Hosp 31, 2297–2312. <https://doi.org/10.3305/nh.2015.31.5.8589>

Marcus, P.M., Savitz, D.A., Millikan, R.C., Morgenstern, H., 1998. Female breast cancer and trihalomethane levels in drinking water in North Carolina. Epidemiology 9, 156–160.

Marie, C., Léger, S., Guttmann, A., Marchiset, N., Rivière, O., Perthus, I., Lémery, D., Vendittelli, F., Sauvant-Rochat, M.-P., 2018. In utero exposure to arsenic in tap water and congenital anomalies: A French semi-ecological study. Int J Hyg Environ Health 221, 1116–1123. <https://doi.org/10.1016/j.ijheh.2018.07.012>

Marque, S., Jacqmin-Gadda, H., Dartigues, J.-F., Commenges, D., 2003. Cardiovascular mortality and calcium and magnesium in drinking water: an ecological study in elderly people. Eur J Epidemiol 18, 305–309. <https://doi.org/10.1023/a:1023618728056>

Marshall, G., Ferreccio, C., Yuan, Y., Bates, M.N., Steinmaus, C., Selvin, S., Liaw, J., Smith, A.H., 2007. Fifty-year study of lung and bladder cancer mortality in Chile related to arsenic in drinking water. J Natl Cancer Inst 99, 920–928. <https://doi.org/10.1093/jnci/djm004>

Martinez, H., 2014a. Fluid intake in Mexican adults; a cross-sectional study. Nutr Hosp 29, 1179–1187. <https://doi.org/10.3305/nh.2014.29.5.7447>

Martinez, H., 2014b. Fluid consumption by Mexican women during pregnancy and first semester of lactation. Biomed Res Int 2014, 603282. <https://doi.org/10.1155/2014/603282>

Martínez, V., Creus, A., Venegas, W., Arroyo, A., Beck, J.P., Gebel, T.W., Surrallés, J., Marcos, R., 2004. Evaluation of micronucleus induction in a Chilean population environmentally exposed to arsenic. Mutat Res 564, 65–74. <https://doi.org/10.1016/j.mrgentox.2004.07.008>

Mashau, F., Ncube, E.J., Voyi, K., 2019. Maternal urinary levels of trichloroacetic acid and association with adverse pregnancy outcomes. J Water Health 17, 884–895. <https://doi.org/10.2166/wh.2019.109>

Masoumi, S.J., Mehrabani, D., Moradi, F., Zare, N., Saberi-Firouzi, M., Mazloom, Z., 2015. The prevalence of dyspepsia symptoms and its correlation with the quality of life among Qashqai Turkish migrating nomads in Fars Province, Southern Iran. Pak J Med Sci 31, 325–330. <https://doi.org/10.12669/pjms.312.6956>

Mastrantonio, M., Bai, E., Uccelli, R., Cordiano, V., Screpanti, A., Crosignani, P., 2018. Drinking water contamination from perfluoroalkyl substances (PFAS): an ecological mortality study in the Veneto Region, Italy. Eur J Public Health 28, 180–185. <https://doi.org/10.1093/eurpub/ckx066>

Matanock, A., Anderson, T., Ayers, T., Likicho, L., Wamimbi, R., Lu, X., Emeetai, T., Kakande, C., Mutabazi, M., Quick, R., 2016. Integrating Water Treatment into Antenatal Care: Impact on Use of Maternal Health Services and Household Water Treatment by Mothers—Rural Uganda, 2013. Am J Trop Med Hyg 94, 1150–1156. <https://doi.org/10.4269/ajtmh.15-0356>

May, D.L., 1995. Patient perceptions of self-induced water intoxication. Archives of Psychiatric Nursing 9, 295–304. <https://doi.org/10.1016/S0883-9417(95)80049-2>

Mazumder, D.N., Haque, R., Ghosh, N., De, B.K., Santra, A., Chakraborti, D., Smith, A.H., 2000. Arsenic in drinking water and the prevalence of respiratory effects in West Bengal, India. Int J Epidemiol 29, 1047–1052. <https://doi.org/10.1093/ije/29.6.1047>

Mbereko, A., Scott, D., John Chimbari, M., 2016. The relationship between HIV and AIDS and water scarcity in Nyamakate resettlements land, north-central Zimbabwe. Afr J AIDS Res 15, 349–357. <https://doi.org/10.2989/16085906.2016.1247735>

Mbugua, S., Musikoyo, E., Ndungi, F., Sang, R., Kamau-Mbuthia, E., Ngotho, D., 2014. Determinants of diarrhea among young children under the age of five in Kenya, evidence from KDHS 2008-09. APS 28, 1046. <https://doi.org/10.11564/28-0-556>

McDonald, C., Hoque, R., Huda, N., Cherry, N., 2007. Risk of arsenic-related skin lesions in Bangladeshi villages at relatively low exposure: a report from Gonoshasthaya Kendra. Bull World Health Organ 85, 668–673. <https://doi.org/10.2471/blt.06.036764>

McDonald, C., Hoque, R., Huda, N., Cherry, N., 2006. Prevalence of arsenic-related skin lesions in 53 widely-scattered villages of Bangladesh: an ecological survey. J Health Popul Nutr 24, 228–235.

McElroy, J.A., Gangnon, R.E., Newcomb, P.A., Kanarek, M.S., Anderson, H.A., Brook, J.V., Trentham-Dietz, A., Remington, P.L., 2007. Risk of breast cancer for women living in rural areas from adult exposure to atrazine from well water in Wisconsin. J Expo Sci Environ Epidemiol 17, 207–214. <https://doi.org/10.1038/sj.jes.7500511>

McElroy, J.A., Trentham-Dietz, A., Gangnon, R.E., Hampton, J.M., Bersch, A.J., Kanarek, M.S., Newcomb, P.A., 2008. Nitrogen-nitrate exposure from drinking water and colorectal cancer risk for rural women in Wisconsin, USA. J Water Health 6, 399–409. <https://doi.org/10.2166/wh.2008.048>

McGeehin, M.A., Reif, J.S., Becher, J.C., Mangione, E.J., 1993. Case-control study of bladder cancer and water disinfection methods in Colorado. Am J Epidemiol 138, 492–501. <https://doi.org/10.1093/oxfordjournals.aje.a116883>

McLennan, J.D., 2015. Choosing bottled over tapped: drinking water in the Dominican Republic. Journal of Water, Sanitation and Hygiene for Development 5, 9–16. <https://doi.org/10.2166/washdev.2014.076>

Meacher, D.M., Menzel, D.B., Dillencourt, M.D., Bic, L.F., Schoof, R.A., Yost, L.J., Eickhoff, J.C., Farr, C.H., 2002. Estimation of Multimedia Inorganic Arsenic Intake in the U.S. Population. Human and Ecological Risk Assessment: An International Journal 8, 1697–1721. <https://doi.org/10.1080/20028091057565>

Mechenro, J., Venugopal, G., Buvnesh Kumar, M., Balakrishnan, D., Ramakrishna, B.S., 2018. Vitamin D status in Kancheepuram District, Tamil Nadu, India. BMC Public Health 18, 1345. <https://doi.org/10.1186/s12889-018-6244-5>

Meena, C., Dwivedi, S., Rathore, S., Gonmei, Z., Gs, T., Bala, K., Ss, M., 2017. Assessment of skeletal fluorosis among children in two blocks of rural area, Jaipur District, Rajasthan, India. Asian Journal of Pharmaceutical and Clinical Research 10, 322. <https://doi.org/10.22159/ajpcr.2017.v10i9.19993>

Meliker, J.R., Wahl, R.L., Cameron, L.L., Nriagu, J.O., 2007. Arsenic in drinking water and cerebrovascular disease, diabetes mellitus, and kidney disease in Michigan: a standardized mortality ratio analysis. Environ Health 6, 4. <https://doi.org/10.1186/1476-069X-6-4>

Melkonian, S., Argos, M., Chen, Y., Parvez, F., Pierce, B., Ahmed, A., Islam, T., Ahsan, H., 2012. Intakes of Several Nutrients Are Associated with Incidence of Arsenic-Related Keratotic Skin Lesions in Bangladesh12. J Nutr 142, 2128–2134. <https://doi.org/10.3945/jn.112.165720>

Mendez, W., Dederick, E., Cohen, J., 2010. Drinking water contribution to aggregate perchlorate intake of reproductive-age women in the United States estimated by dietary intake simulation and analysis of urinary excretion data. J Expo Sci Environ Epidemiol 20, 288–297. <https://doi.org/10.1038/jes.2009.50>

Mendez, W.M., Eftim, S., Cohen, J., Warren, I., Cowden, J., Lee, J.S., Sams, R., 2017. Relationships between arsenic concentrations in drinking water and lung and bladder cancer incidence in U.S. counties. J Expo Sci Environ Epidemiol 27, 235–243. <https://doi.org/10.1038/jes.2016.58>

Meng, F., Zhao, R., Liu, P., Liu, L., Liu, S., 2013. Assessment of iodine status in children, adults, pregnant women and lactating women in iodine-replete areas of China. PLoS One 8, e81294. <https://doi.org/10.1371/journal.pone.0081294>

Mensink, G.B.M., Beitz, R., 2004. Food and nutrient intake in East and West Germany, 8 years after the reunification—The German Nutrition Survey 1998. Eur J Clin Nutr 58, 1000–1010. <https://doi.org/10.1038/sj.ejcn.1601923>

Mercer, N., Hanrahan, M., 2017. “Straight from the heavens into your bucket”: domestic rainwater harvesting as a measure to improve water security in a subarctic indigenous community. International Journal of Circumpolar Health 76, 1312223. <https://doi.org/10.1080/22423982.2017.1312223>

Merrill, R.D., Shamim, A.A., Ali, H., Jahan, N., Labrique, A.B., Christian, P., West, K.P., 2012a. Groundwater iron assessment and consumption by women in rural northwestern Bangladesh. Int J Vitam Nutr Res 82, 5–14. <https://doi.org/10.1024/0300-9831/a000089>

Merrill, R.D., Shamim, A.A., Ali, H., Jahan, N., Labrique, A.B., Schulze, K., Christian, P., West, K.P., 2011. Iron status of women is associated with the iron concentration of potable groundwater in rural Bangladesh. J Nutr 141, 944–949. <https://doi.org/10.3945/jn.111.138628>

Merrill, R.D., Shamim, A.A., Ali, H., Labrique, A.B., Schulze, K., Christian, P., West, K.P., 2012b. High prevalence of anemia with lack of iron deficiency among women in rural Bangladesh: a role for thalassemia and iron in groundwater. Asia Pac J Clin Nutr 21, 416–424.

Messier, V., Lévesque, B., Proulx, J.-F., Rochette, L., Libman, M.D., Ward, B.J., Serhir, B., Couillard, M., Ogden, N.H., Dewailly, E., Hubert, B., Déry, S., Barthe, C., Murphy, D., Dixon, B., 2009. Seroprevalence of Toxoplasma gondii among Nunavik Inuit (Canada). Zoonoses Public Health 56, 188–197. <https://doi.org/10.1111/j.1863-2378.2008.01177.x>

Messier, V., Lévesque, B., Proulx, J.-F., Rochette, L., Serhir, B., Couillard, M., Ward, B.J., Libman, M.D., Dewailly, E., Déry, S., 2012. Seroprevalence of seven zoonotic infections in Nunavik, Quebec (Canada). Zoonoses Public Health 59, 107–117. <https://doi.org/10.1111/j.1863-2378.2011.01424.x>

Meusburger, S., Reichart, S., Kapfer, S., Schableger, K., Fretz, R., Allerberger, F., 2007. Outbreak of acute gastroenteritis of unknown etiology caused by contaminated drinking water in a rural village in Austria, August 2006. Wien Klin Wochenschr 119, 717–721. <https://doi.org/10.1007/s00508-007-0916-y>

Meyer, I., Heinrich, J., Trepka, M.J., Krause, C., Schulz, C., Meyer, E., Lippold, U., 1998. The effect of lead in tap water on blood lead in children in a smelter town. Sci Total Environ 209, 255–271.

Mhaskar, R.S., Ricardo, I., Azliyati, A., Laxminarayan, R., Amol, B., Santosh, W., Boo, K., 2013. Assessment of Risk Factors of Helicobacter Pylori Infection and Peptic Ulcer Disease. J Glob Infect Dis 5, 60–67. <https://doi.org/10.4103/0974-777X.112288>

Michaud, D.S., Kogevinas, M., Cantor, K.P., Villanueva, C.M., Garcia-Closas, M., Rothman, N., Malats, N., Real, F.X., Serra, C., Garcia-Closas, R., Tardon, A., Carrato, A., Dosemeci, M., Silverman, D.T., 2007. Total Fluid and Water Consumption and the Joint Effect of Exposure to Disinfection By-Products on Risk of Bladder Cancer. Environmental Health Perspectives 115, 1569–1572. <https://doi.org/10.1289/ehp.10281>

Michel-Ramirez, G., Recio-Vega, R., Lantz, R.C., Gandolfi, A.J., Olivas-Calderon, E., Chau, B.T., Amistadi, M.K., 2020. Assessment of YAP gene polymorphisms and arsenic interaction in Mexican women with breast cancer. J Appl Toxicol 40, 342–351. <https://doi.org/10.1002/jat.3907>

Migeot, V., Albouy-Llaty, M., Carles, C., Limousi, F., Strezlec, S., Dupuis, A., Rabouan, S., 2013. Drinking-water exposure to a mixture of nitrate and low-dose atrazine metabolites and small-for-gestational age (SGA) babies: a historic cohort study. Environ Res 122, 58–64. <https://doi.org/10.1016/j.envres.2012.12.007>

Miles, A.M., Singer, P.C., Ashley, D.L., Lynberg, M.C., Mendola, P., Langlois, P.H., Nuckols, J.R., 2002. Comparison of trihalomethanes in tap water and blood. Environ Sci Technol 36, 1692–1698. <https://doi.org/10.1021/es001991j>

Milla-Tobarra, M., García-Hermoso, A., Lahoz-García, N., Notario-Pacheco, B., Lucas-de la Cruz, L., Pozuelo-Carrascosa, D.P., García-Meseguer, M.J., Martínez-Vizcaíno, V., 2016. The association between water intake, body composition and cardiometabolic factors among children - The Cuenca study. Nutr Hosp 33, 312. <https://doi.org/10.20960/nh.312>

Miller, E., Buys, L., 2008. Water-Recycling In South-East Queensland, Australia: What Do Men And Women Think? Rural Society 18, 220–229. <https://doi.org/10.5172/rsj.351.18.3.220>

Milton, A.H., Hasan, Z., Rahman, A., Rahman, M., 2001. Chronic Arsenic Poisoning and Respiratory Effects in Bangladesh. Journal of Occupational Health 43, 136–140. <https://doi.org/10.1539/joh.43.136>

Milton, A.H., Shahidullah, S.M., Smith, W., Hossain, K.S., Hasan, Z., Ahmed, K.T., 2010. Association between Chronic Arsenic Exposure and Nutritional Status among the Women of Child Bearing Age: A Case-Control Study in Bangladesh. Int J Environ Res Public Health 7, 2811–2821. <https://doi.org/10.3390/ijerph7072811>

Milton, A.H., Smith, W., Rahman, B., Hasan, Z., Kulsum, U., Dear, K., Rakibuddin, M., Ali, A., 2005. Chronic arsenic exposure and adverse pregnancy outcomes in bangladesh. Epidemiology 16, 82–86. <https://doi.org/10.1097/01.ede.0000147105.94041.e6>

Mirza, N., Tarannum, T., Ahmed, T., 2017. Contribution of Drinking Water to Dietary Intakes of Nutrient Minerals in Bangladesh. <https://doi.org/10.1061/9780784480618.016#sthash.iReAAnbE.dpuf>

Mishra, B.K., Gupta, S.K., Sinha, A., 2014. Human health risk analysis from disinfection by-products (DBPs) in drinking and bathing water of some Indian cities. Iranian Journal of Environmental Health Science & Engineering 12, 1.

Mittelmark, M.B., Bull, T., 2010. Social determinants of rest deprivation amongst Ghanaian women: national and urban-rural comparisons with data from a cross-sectional nationally representative survey. BMC Public Health 10, 580. <https://doi.org/10.1186/1471-2458-10-580>

Miyazaki, K., Ushijima, K., Kadono, T., Inaoka, T., Watanabe, C., Ohtsuka, R., 2003. Negative Correlation between Urinary Selenium and Arsenic Levels of the Residents Living in an Arsenic-Contaminated Area in Bangladesh. Journal of Health Science 49, 239–242. <https://doi.org/10.1248/jhs.49.239>

Mo, J., Xia, Y., Wade, T.J., Schmitt, M., Le, X.C., Dang, R., Mumford, J.L., 2006. Chronic Arsenic Exposure and Oxidative Stress: OGG1 Expression and Arsenic Exposure, Nail Selenium, and Skin Hyperkeratosis in Inner Mongolia. Environ Health Perspect 114, 835–841. <https://doi.org/10.1289/ehp.8723>

Moghaddam, M.A., Azadegan, M., Maknoon, R., 2014. Public awareness and performance regarding nitrate pollution in nitrate-polluted area of Tehran, Iran. <https://doi.org/10.30638/EEMJ.2014.064>

Mohammadi, A.A., Yousefi, M., Yaseri, M., Jalilzadeh, M., Mahvi, A.H., 2017. Skeletal fluorosis in relation to drinking water in rural areas of West Azerbaijan, Iran. Sci Rep 7, 17300. <https://doi.org/10.1038/s41598-017-17328-8>

Mohammed Mahdy, A.K., Lim, Y. a. L., Surin, J., Wan, K.L., Al-Mekhlafi, M.S.H., 2008. Risk factors for endemic giardiasis: highlighting the possible association of contaminated water and food. Trans R Soc Trop Med Hyg 102, 465–470. <https://doi.org/10.1016/j.trstmh.2008.02.004>

Mohammed, N., Rilwanu, T., 2016. Nature of Hydrogeology, Water Supply, and the Role of Women in the Management of Water in Urban and Peri-Urban Kano, Nigeria 2, 287–296.

Mohd Ridzwan, S.F., Anual, Z.F., Sahani, M., Ghazali, A.R., 2013. Neurobehavioral Performance of Estate Residents with Privately-Treated Water Supply. Iran J Public Health 42, 1374–1386.

Moist, L., Sontrop, J.M., Garg, A.X., Clark, W.F., Suri, R.S., Gratton, R., Salvadori, M., Nevis, I., Macnab, J.J., 2010. Risk of pregnancy-related hypertension within 5 years of exposure to drinking water contaminated with Escherichia coli O157:H7. J Clin Hypertens (Greenwich) 12, 613–620. <https://doi.org/10.1111/j.1751-7176.2010.00288.x>

Mondal, D., Lopez-Espinosa, M.-J., Armstrong, B., Stein, C.R., Fletcher, T., 2012. Relationships of perfluorooctanoate and perfluorooctane sulfonate serum concentrations between mother-child pairs in a population with perfluorooctanoate exposure from drinking water. Environ Health Perspect 120, 752–757. <https://doi.org/10.1289/ehp.1104538>

Monnard, C.R., Grasser, E.K., 2017. Water ingestion decreases cardiac workload time-dependent in healthy adults with no effect of gender. Sci Rep 7, 7939. <https://doi.org/10.1038/s41598-017-08446-4>

Montenegro-Bethancourt, G., Vossenaar, M., Doak, C.M., Solomons, N.W., 2009. Total daily water intake in Guatemalan children. Food Nutr Bull 30, 340–350. <https://doi.org/10.1177/156482650903000405>

Moon, K.A., Guallar, E., Umans, J.G., Devereux, R.B., Best, L.G., Francesconi, K.A., Goessler, W., Pollak, J., Silbergeld, E.K., Howard, B.V., Navas-Acien, A., 2013. Association between Low to Moderate Arsenic Exposure and Incident Cardiovascular Disease. A Prospective Cohort Study. Ann Intern Med 159, 649–659. <https://doi.org/10.7326/0003-4819-159-10-201311190-00719>

Moore, L.E., Smith, A.H., Eng, C., DeVries, S., Kalman, D., Bhargava, V., Chew, K., Ferreccio, C., Rey, O.A., Hopenhayn, C., Biggs, M.L., Bates, M.N., Waldman, F.M., 2003. P53 alterations in bladder tumors from arsenic and tobacco exposed patients. Carcinogenesis 24, 1785–1791. <https://doi.org/10.1093/carcin/bgg136>

Mora, A.M., van Wendel de Joode, B., Mergler, D., Córdoba, L., Cano, C., Quesada, R., Smith, D.R., Menezes-Filho, J.A., Lundh, T., Lindh, C.H., Bradman, A., Eskenazi, B., 2014. Blood and Hair Manganese Concentrations in Pregnant Women from the Infants’ Environmental Health Study (ISA) in Costa Rica. Environ. Sci. Technol. 48, 3467–3476. <https://doi.org/10.1021/es404279r>

Morakinyo, O.M., Adebowale, S.A., Oloruntoba, E.O., 2015. Wealth status and sex differential of household head: implication for source of drinking water in Nigeria. Arch Public Health 73, 58. <https://doi.org/10.1186/s13690-015-0105-9>

Morales-Suarez-Varela, M., Llopis Gonzalez, A., Tejerizo Perez, M.L., Ferrandiz Ferragud, J., 1993. Concentration of nitrates in drinking water and its relationship with bladder cancer. J Environ Pathol Toxicol Oncol 12, 229–236.

Morales-Suarez-Varela, M.M., Llopis-Gonzalez, A., Tejerizo-Perez, M.L., 1995. Impact of nitrates in drinking water on cancer mortality in Valencia, Spain. Eur J Epidemiol 11, 15–21. <https://doi.org/10.1007/BF01719941>

Mordi, R.M., Okaka, C.E., 2009. Prevalence of Intestinal Parasites in Edo State. International Journal of Health Research 2, 253–257. <https://doi.org/10.4314/ijhr.v2i3.47909>

Morgan, C., Bowling, M., Bartram, J., Lyn Kayser, G., 2017. Water, sanitation, and hygiene in schools: Status and implications of low coverage in Ethiopia, Kenya, Mozambique, Rwanda, Uganda, and Zambia. Int J Hyg Environ Health 220, 950–959. <https://doi.org/10.1016/j.ijheh.2017.03.015>

Mortensen, M.E., Birch, R., Wong, L.-Y., Valentin-Blasini, L., Boyle, E.B., Caldwell, K.L., Merrill, L.S., Moye, J., Blount, B.C., 2016. Thyroid antagonists and thyroid indicators in U.S. pregnant women in the Vanguard Study of the National Children’s Study. Environ Res 149, 179–188. <https://doi.org/10.1016/j.envres.2016.05.017>

Mostafa, M.G., McDonald, J.C., Cherry, N.M., 2008. Lung cancer and exposure to arsenic in rural Bangladesh. Occupational and Environmental Medicine 65, 765–768. <https://doi.org/10.1136/oem.2007.037895>

Moura, I.P. da S., Ferreira, I.P., Pontes, A.N., Bichara, C.N.C., 2019. Toxoplasmosis knowledge and preventive behavior among pregnant women in the city of Imperatriz, Maranhão, Brazil. Cien Saude Colet 24, 3933–3946. <https://doi.org/10.1590/1413-812320182410.21702017>

Mtapuri-Zinyowera, S., Ruhanya, V., Midzi, N., Berejena, C., Chin’ombe, N., Nziramasanga, P., Nyandoro, G., Mduluza, T., 2014. Human parasitic protozoa in drinking water sources in rural Zimbabwe and their link to HIV infection. Germs 4, 86–91. <https://doi.org/10.11599/germs.2014.1061>

Mueller, B.A., Newton, K., Holly, E.A., Preston-Martin, S., 2001. Residential water source and the risk of childhood brain tumors. Environ Health Perspect 109, 551–556.

Mueller, B.A., Searles Nielsen, S., Preston-Martin, S., Holly, E.A., Cordier, S., Filippini, G., Peris-Bonet, R., Choi, N.W., 2004. Household water source and the risk of childhood brain tumours: results of the SEARCH International Brain Tumor Study. Int J Epidemiol 33, 1209–1216. <https://doi.org/10.1093/ije/dyh215>

Mukherjee, B., Bindhani, B., Saha, H., Sinha, D., Ray, M.R., 2014. Platelet hyperactivity, neurobehavioral symptoms and depression among Indian women chronically exposed to low level of arsenic. Neurotoxicology 45, 159–167. <https://doi.org/10.1016/j.neuro.2014.10.011>

Mukherjee, S.C., Rahman, M.M., Chowdhury, U.K., Sengupta, M.K., Lodh, D., Chanda, C.R., Saha, K.C., Chakraborti, D., 2003. Neuropathy in arsenic toxicity from groundwater arsenic contamination in West Bengal, India. J Environ Sci Health A Tox Hazard Subst Environ Eng 38, 165–183. <https://doi.org/10.1081/ese-120016887>

Mukherjee, S.C., Saha, K.C., Pati, S., Dutta, R.N., Rahman, M.M., Sengupta, M.K., Ahamed, S., Lodh, D., Das, B., Hossain, M.A., Nayak, B., Mukherjee, A., Chakraborti, D., Dulta, S.K., Palit, S.K., Kaies, I., Barua, A.K., Asad, K.A., 2005. Murshidabad--one of the nine groundwater arsenic-affected districts of West Bengal, India. Part II: dermatological, neurological, and obstetric findings. Clin Toxicol (Phila) 43, 835–848. <https://doi.org/10.1080/15563650500357495>

Mulyani, E.Y., Hardinsyah, H., Briawan, D., Santoso, B.I., 2018. The Impact of Dehydration in the Third Trimesters on Pregnancy Outcome-Infant Birth Weight and Length. Jurnal Gizi dan Pangan 13, 157–164. <https://doi.org/10.25182/jgp.2018.13.3.157-164>

Mumford, J.L., Wu, K., Xia, Y., Kwok, R., Yang, Z., Foster, J., Sanders, W.E., 2007. Chronic Arsenic Exposure and Cardiac Repolarization Abnormalities with QT Interval Prolongation in a Population-based Study. Environ Health Perspect 115, 690–694. <https://doi.org/10.1289/ehp.9686>

Mummery, W.K., Duncan, M., Kift, R., 2007. Socio-economic differences in public opinion regarding water fluoridation in Queensland. Aust N Z J Public Health 31, 336–339. <https://doi.org/10.1111/j.1753-6405.2007.00082.x>

Munger, R., Isacson, P., Hu, S., Burns, T., Hanson, J., Lynch, C.F., Cherryholmes, K., Van Dorpe, P., Hausler, W.J., 1997. Intrauterine growth retardation in Iowa communities with herbicide-contaminated drinking water supplies. Environ Health Perspect 105, 308–314.

Muñoz, A., Chervona, Y., Hall, M., Kluz, T., Gamble, M.V., Costa, M., 2015. Sex-specific patterns and deregulation of endocrine pathways in the gene expression profiles of Bangladeshi adults exposed to arsenic contaminated drinking water. Toxicology and Applied Pharmacology 284, 330–338. <https://doi.org/10.1016/j.taap.2015.02.025>

Muñoz-Antoli, C., Pavón, A., Marcilla, A., Toledo, R., Esteban, J.G., 2014. Prevalence and risk factors related to intestinal parasites among children in Department of Rio San Juan, Nicaragua. Trans R Soc Trop Med Hyg 108, 774–782. <https://doi.org/10.1093/trstmh/tru160>

Murebwayire, E., Njanaake, K., Ngabonziza, J.C.S., Jaoko, W., Njunwa, K.J., 2017. Seroprevalence and risk factors of Toxoplasma gondii infection among pregnant women attending antenatal care in Kigali, Rwanda. Tanzania Journal of Health Research 19. <https://doi.org/10.4314/thrb.v19i1.2>

Murenjekwa, W., Makasi, R., Ntozini, R., Chasekwa, B., Mutasa, K., Moulton, L.H., Tielsch, J.M., Humphrey, J.H., Smith, L.E., Prendergast, A.J., Bourke, C.D., for the SHINE Trial Team, 2021. Determinants of Urogenital Schistosomiasis Among Pregnant Women and its Association With Pregnancy Outcomes, Neonatal Deaths, and Child Growth. The Journal of Infectious Diseases 223, 1433–1444. <https://doi.org/10.1093/infdis/jiz664>

Murray, M.P., Sharmin, R., 2015. Groundwater arsenic and education attainment in Bangladesh. J Health Popul Nutr 33, 20. <https://doi.org/10.1186/s41043-015-0029-6>

Mustafa, D., Younis, U., Elhag, A., Khartoum, Sudan, 2018. The relationship between the fluoride levels in drinking water and the schooling performance of children in rural areas of Khartoum state, Sudan. Fluoride 51, 102–113.

Myers, S.L., Lobdell, D.T., Liu, Z., Xia, Y., Ren, H., Li, Y., Kwok, R.K., Mumford, J.L., Mendola, P., 2010. Maternal drinking water arsenic exposure and perinatal outcomes in inner Mongolia, China. J Epidemiol Community Health 64, 325–329. <https://doi.org/10.1136/jech.2008.084392>

N, S., M, V., M, A., M, R., A, R., Pk, S., Ps, K., La, P., 2010. Spatial patterns of fetal loss and infant death in an arsenic-affected area in Bangladesh. International journal of health geographics 9. <https://doi.org/10.1186/1476-072X-9-53>

Nadali, A., Rahmani, A., Asgari, G., Leili, M., Norouzi, H.A., Naghibi, A., 2019. The Assessment of Trihalomethanes Concentrations in Drinking Water of Hamadan and Tuyserkan Cities, Western Iran and Its Health Risk on the Exposed Population. J Res Health Sci 19, e00441.

Nagarajappa, R., Pujara, P., Sharda, A.J., Asawa, K., Tak, M., Aapaliya, P., Bhanushali, N., 2013. Comparative Assessment of Intelligence Quotient among Children Living in High and Low Fluoride Areas of Kutch, India-a Pilot Study. Iran J Public Health 42, 813–818.

Nahar, N., 2009. Impacts of arsenic contamination in groundwater: case study of some villages in Bangladesh. Environ Dev Sustain 11, 571–588. <https://doi.org/10.1007/s10668-007-9130-3>

Nahian, M.A., Ahmed, A., Lázár, A.N., Hutton, C.W., Salehin, M., Streatfield, P.K., 2018. Drinking water salinity associated health crisis in coastal Bangladesh. Elementa: Science of the Anthropocene 6, 2. <https://doi.org/10.1525/elementa.143>

Naik, S.R., Aggarwal, R., Salunke, P.N., Mehrotra, N.N., 1992. A large waterborne viral hepatitis E epidemic in Kanpur, India. Bull World Health Organ 70, 597–604.

Nakachi, K., Limtrakul, P., Sonklin, P., Sonklin, O., Jarern, C.T., Lipigorngoson, S., Arai, K., Sone, Y., Imai, K., Suga, K., Matsuyama, S., Shimizu, H., Takahashi, T., Suttajit, M., 1999. Risk factors for lung cancer among Northern Thai women: epidemiological, nutritional, serological, and bacteriological surveys of residents in high- and low-incidence areas. Jpn J Cancer Res 90, 1187–1195. <https://doi.org/10.1111/j.1349-7006.1999.tb00694.x>

Nakibirango, J., Mugenyi, V., Nsaba, D., Nsimemukama, A., Rugera, S.P., Okongo, B., 2019. Prevalence of cryptosporidiosis and hygiene practices among HIV/AIDS patients in southwest Uganda. HIV AIDS (Auckl) 11, 141–145. <https://doi.org/10.2147/HIV.S206195>

Narain, V., 2014. Shifting the Focus From Women to Gender Relations: Assessing the Impacts of Water Supply Interventions in the Morni–Shiwalik Hills of Northwest India. mred 34, 208–213. <https://doi.org/10.1659/MRD-JOURNAL-D-13-00104.1>

Nascimento, S., Baierle, M., Göethel, G., Barth, A., Brucker, N., Charão, M., Sauer, E., Gauer, B., Arbo, M.D., Altknecht, L., Jager, M., Dias, A.C.G., de Salles, J.F., Saint’ Pierre, T., Gioda, A., Moresco, R., Garcia, S.C., 2016. Associations among environmental exposure to manganese, neuropsychological performance, oxidative damage and kidney biomarkers in children. Environ Res 147, 32–43. <https://doi.org/10.1016/j.envres.2016.01.035>

Nassir, A.M., 2019. Prevalence and characterization of urolithiasis in the Western region of Saudi Arabia. Urol Ann 11, 347–352. <https://doi.org/10.4103/UA.UA_56_19>

Navasumrit, P., Chaisatra, K., Promvijit, J., Parnlob, V., Waraprasit, S., Chompoobut, C., Binh, T.T., Hai, D.N., Bao, N.D., Hai, N.K., Kim, K.-W., Samson, L.D., Graziano, J.H., Mahidol, C., Ruchirawat, M., 2019. Exposure to arsenic in utero is associated with various types of DNA damage and micronuclei in newborns: a birth cohort study. Environ Health 18, 51. <https://doi.org/10.1186/s12940-019-0481-7>

Naz, A., Mishra, B., Gupta, S., 2016. Human Health Risk Assessment of Chromium in Drinking Water: A Case Study of Sukinda Chromite Mine, Odisha, India. Water Quality Exposure and Health 8. <https://doi.org/10.1007/s12403-016-0199-5>

Nazir, M.M., Akhtar, M., Maqbool, A., Waheed, A., Sajid, M.A., Ali, M.A., Oneeb, M., Alam, M.A., Ahmad, A.N., Nazir, N., Fatima, S., Lindsay, D.S., 2017. Antibody Prevalence and Risk Factors for Toxoplasma gondii Infection in Women from Multan, Pakistan. Zoonoses Public Health 64, 537–542. <https://doi.org/10.1111/zph.12336>

Neamtiu, I., Bloom, M.S., Gati, G., Goessler, W., Surdu, S., Pop, C., Braeuer, S., Fitzgerald, E.F., Baciu, C., Lupsa, I.R., Anastasiu, D., Gurzau, E., 2015. Pregnant women in Timis County, Romania are exposed primarily to low-level (<10 μg/L) arsenic through residential drinking water consumption. Int J Hyg Environ Health 218, 371–379. <https://doi.org/10.1016/j.ijheh.2015.01.004>

Negero, J., Yohannes, M., Woldemichael, K., Tegegne, D., 2017. Seroprevalence and potential risk factors of T. gondii infection in pregnant women attending antenatal care at Bonga Hospital, Southwestern Ethiopia. Int J Infect Dis 57, 44–49. <https://doi.org/10.1016/j.ijid.2017.01.013>

Nerbrand, C., Svärdsudd, K., Ek, J., Tibblin, G., 1992. Cardiovascular mortality and morbidity in seven counties in Sweden in relation to water hardness and geological settings. The project: myocardial infarction in mid-Sweden. Eur Heart J 13, 721–727. <https://doi.org/10.1093/oxfordjournals.eurheartj.a060246>

Nergiz-Unal, R., Akal Yildiz, E., Samur, G., Besler, H.T., Rakicioğlu, N., 2017. Trends in fluid consumption and beverage choices among adults reveal preferences for ayran and black tea in central Turkey: Drinking habits in adults. Nutr Diet 74, 74–81. <https://doi.org/10.1111/1747-0080.12316>

Nerkar, S.S., Tamhankar, A.J., Johansson, E., Lundborg, C.S., 2013. Improvement in health and empowerment of families as a result of watershed management in a tribal area in India - a qualitative study. BMC Int Health Hum Rights 13, 42. <https://doi.org/10.1186/1472-698X-13-42>

Nermell, B., Lindberg, A.-L., Rahman, M., Berglund, M., Persson, L.A., El Arifeen, S., Vahter, M., 2008. Urinary arsenic concentration adjustment factors and malnutrition. Environ Res 106, 212–218. <https://doi.org/10.1016/j.envres.2007.08.005>

Neuberger, J.S., Mulhall, M., Pomatto, M.C., Sheverbush, J., Hassanein, R.S., 1990. Health problems in Galena, Kansas: A heavy metal mining Superfund site. Science of The Total Environment 94, 261–272. <https://doi.org/10.1016/0048-9697(90)90175-T>

Ng, J.C., Noller, B.N., Naidu, R., Bundschuh, J., Bhattacharya, P. (Eds.), 2012. Arsenic metabolism, lactate dehydrogenase and electrocardiogram abnormality among residents in the arseniasis-endemic areas of southwestern Taiwan, in: Understanding the Geological and Medical Interface of Arsenic - As 2012. CRC Press, pp. 205–206. <https://doi.org/10.1201/b12522-67>

Ngui, R., Lim, Y.A.L., Amir, N.F.H., Nissapatorn, V., Mahmud, R., 2011. Seroprevalence and sources of toxoplasmosis among Orang Asli (indigenous) communities in Peninsular Malaysia. Am J Trop Med Hyg 85, 660–666. <https://doi.org/10.4269/ajtmh.2011.11-0058>

Nguyen, T.N., Jakus, P.M., Riddel, M., Shaw, W.D., 2010. An empirical model of perceived mortality risks for selected U.S. Arsenic hot spots. Risk Anal 30, 1550–1562. <https://doi.org/10.1111/j.1539-6924.2010.01450.x>

Nguyen, V.A., Bang, S., Viet, P.H., Kim, K.-W., 2009. Contamination of groundwater and risk assessment for arsenic exposure in Ha Nam province, Vietnam. Environment International 35, 466–472. <https://doi.org/10.1016/j.envint.2008.07.014>

Niedzwiecki, M.M., Liu, X., Hall, M.N., Thomas, T., Slavkovich, V., Ilievski, V., Levy, D., Alam, S., Siddique, A.B., Parvez, F., Graziano, J.H., Gamble, M.V., 2015. Sex-specific associations of arsenic exposure with global DNA methylation and hydroxymethylation in leukocytes: results from two studies in Bangladesh. Cancer Epidemiol Biomarkers Prev 24, 1748–1757. <https://doi.org/10.1158/1055-9965.EPI-15-0432>

Nielsen, G.D., Søderberg, U., Jørgensen, P.J., Templeton, D.M., Rasmussen, S.N., Andersen, K.E., Grandjean, P., 1999. Absorption and retention of nickel from drinking water in relation to food intake and nickel sensitivity. Toxicol Appl Pharmacol 154, 67–75. <https://doi.org/10.1006/taap.1998.8577>

Nielsen-Pincus, M., Sussman, P., Bennett, D.E., Gosnell, H., Parker, R., 2017. The Influence of Place on the Willingness to Pay for Ecosystem Services. Society & Natural Resources 30, 1423–1441. <https://doi.org/10.1080/08941920.2017.1347976>

Nimri, L.F., 1994. Prevalence of giardiasis among primary school children. Child Care Health Dev 20, 231–237. <https://doi.org/10.1111/j.1365-2214.1994.tb00386.x>

Ning, Y.J., Wang, X., Ren, L., Guo, X., 2013. Effects of Dietary Factors on Selenium Levels of Children to Prevent Kashin–Beck Disease During a High-Prevalence Period in an Endemic Area: a Cohort Study. Biol Trace Elem Res 153, 58–68. <https://doi.org/10.1007/s12011-013-9651-6>

Nissapatorn, V., Suwanrath, C., Sawangjaroen, N., Ling, L.Y., Chandeying, V., 2011. Toxoplasmosis-Serological Evidence and Associated Risk Factors among Pregnant Women in Southern Thailand. Am J Trop Med Hyg 85, 243–247. <https://doi.org/10.4269/ajtmh.2011.10-0633>

Nissensohn, M., Sánchez-Villegas, A., Ortega, R.M., Aranceta-Bartrina, J., Gil, Á., González-Gross, M., Varela-Moreiras, G., Serra-Majem, L., 2016. Beverage Consumption Habits and Association with Total Water and Energy Intakes in the Spanish Population: Findings of the ANIBES Study. Nutrients 8, 232. <https://doi.org/10.3390/nu8040232>

Njunda, A.L., Assob, J.C.N., Nsagha, D.S., Kamga, H.L., Nde, P.F., Yugah, V.C., 2011. Seroprevalence of Toxoplasma gondii infection among pregnant women in Cameroon. J Public Health Africa 2, e24. <https://doi.org/10.4081/jphia.2011.e24>

Nounkeu, C., Kamgno, J., Dharod, J., 2019. Assessment of the relationship between water insecurity, hygiene practices, and incidence of diarrhea among children from rural households of the Menoua Division, West Cameroon. J Public Health Afr 10, 951. <https://doi.org/10.4081/jphia.2019.951>

Nouraie, M., Latifi-Navid, S., Rezvan, H., Radmard, A.-R., Maghsudlu, M., Zaer-Rezaii, H., Amini, S., Siavoshi, F., Malekzadeh, R., 2009. Childhood hygienic practice and family education status determine the prevalence of Helicobacter pylori infection in Iran. Helicobacter 14, 40–46. <https://doi.org/10.1111/j.1523-5378.2009.00657.x>

Nurdin, M.S., Hadju, V., Ansariadi, A., Zulkifli, A., Arundhana, A.I., 2018. Determinants of anemia among pregnant women in jeneponto regency. Asian Pac. J. Health Sci. 5, 130–133. <https://doi.org/10.21276/apjhs.2018.5.1.28>

Nwobodo, C.E., Agwu, A.E., Irohibe, I.J., 2013. Role of Intra-Household Water Management Practices in Transforming Water Resources Available for Agricultural Activities in Benue State, Nigeria. Journal of Agricultural Extension 17, 122–129. <https://doi.org/10.4314/jae.v17i2.16>

Nyati-Jokomo, Z., Chimbari, M.J., 2017. Risk factors for schistosomiasis transmission among school children in Gwanda district, Zimbabwe. Acta Trop 175, 84–90. <https://doi.org/10.1016/j.actatropica.2017.03.033>

Obaidat, M.M., Stringer, A.P., Roess, A.A., 2019. Seroprevalence, risk factors and spatial distribution of West Nile virus in Jordan. Transactions of The Royal Society of Tropical Medicine and Hygiene 113, 24–30. <https://doi.org/10.1093/trstmh/try111>

Obiri-Yeboah, D., Asante Awuku, Y., Adu, J., Pappoe, F., Obboh, E., Nsiah, P., Amoako-Sakyi, D., Simpore, J., 2018. Sero-prevalence and risk factors for hepatitis E virus infection among pregnant women in the Cape Coast Metropolis, Ghana. PLoS One 13, e0191685. <https://doi.org/10.1371/journal.pone.0191685>

O’Bryant, S.E., Edwards, M., Menon, C.V., Gong, G., Barber, R., 2011. Long-term low-level arsenic exposure is associated with poorer neuropsychological functioning: a Project FRONTIER study. Int J Environ Res Public Health 8, 861–874. <https://doi.org/10.3390/ijerph8030861>

Ochoa-Acuña, H., Frankenberger, J., Hahn, L., Carbajo, C., 2009. Drinking-water herbicide exposure in Indiana and prevalence of small-for-gestational-age and preterm delivery. Environ Health Perspect 117, 1619–1624. <https://doi.org/10.1289/ehp.0900784>

Ochoa-Martínez, Á.C., Ruiz-Vera, T., Almendarez-Reyna, C.I., Zarazúa, S., Carrizales-Yáñez, L., Pérez-Maldonado, I.N., 2019. Impact of arsenic exposure on clinical biomarkers indicative of cardiovascular disease risk in Mexican women. Ecotoxicol Environ Saf 169, 678–686. <https://doi.org/10.1016/j.ecoenv.2018.11.088>

O’Connor, L., Walton, J., Flynn, A., 2014. Water intakes and dietary sources of a nationally representative sample of Irish adults. J Hum Nutr Diet 27, 550–556. <https://doi.org/10.1111/jhn.12189>

Ohgami, H., Terao, T., Shiotsuki, I., Ishii, N., Iwata, N., 2009. Lithium levels in drinking water and risk of suicide. Br J Psychiatry 194, 464–465; discussion 446. <https://doi.org/10.1192/bjp.bp.108.055798>

Ohno, K., Yanase, T., Matsuo, Y., Kimura, T., Rahman, M.H., Magara, Y., Matsui, Y., 2007. Arsenic intake via water and food by a population living in an arsenic-affected area of Bangladesh. Sci Total Environ 381, 68–76. <https://doi.org/10.1016/j.scitotenv.2007.03.019>

O’Leary, E.S., Vena, J.E., Freudenheim, J.L., Brasure, J., 2004. Pesticide exposure and risk of breast cancer: a nested case-control study of residentially stable women living on Long Island. Environ Res 94, 134–144. <https://doi.org/10.1016/j.envres.2003.08.001>

Olife, I.C., Okaka, A.N., Dioka, C.E., Meludu, S.C., Orisakwe, O.E., 2007. Iodine status and the effect of soil erosion on trace elements in Nanka and Oba towns of Anambra State, Nigeria. Ann Chim 97, 895–903. <https://doi.org/10.1002/adic.200790074>

Oliveira, P., Zagalo, J., Madeira, N., Neves, O., 2019. Lithium in Public Drinking Water and Suicide Mortality in Portugal: Initial Approach. Acta Med Port 32, 47–52. <https://doi.org/10.20344/amp.10744>

Olmos, V., Navoni, J.A., Calcagno, M.L., Sassone, A.H., Villaamil Lepori, E.C., 2015. Influence of the level of arsenic (As) exposure and the presence of T860C polymorphism in human As urinary metabolic profile. Hum Exp Toxicol 34, 170–178. <https://doi.org/10.1177/0960327114533574>

Ononugbo, C.P., Efere, T., 2017. Estimation of radiation risks due to ingestion of water in Ogba/Egbema/Ndoni Local Government Area of Rivers State, Nigeria using risk models. Journal of Applied Sciences and Environmental Management 21, 839. <https://doi.org/10.4314/jasem.v21i5.8>

Onyido, A.E., Anumba, J.U., Ezechukwu, G.C., Ugha, C., Umeanaeto, P.U., Iwueze, M.O., 2017. Intestinal helminth infections among primary school pupils in Ekwulumili Community, Nnewi South Local Government Area, Anambra State. Nig. J. Para. 38, 185. <https://doi.org/10.4314/njpar.v38i2.10>

Opydo-Szymaczek, J., Borysewicz-Lewicka, M., Poland, P., 2004. Urinary fluoride levels for assessment of fluoride exposure of pregnant women in Poznan, Poland. Fluoride 38.

O’Rourke, M.K., Van de Water, P.K., Jin, S., Rogan, S.P., Weiss, A.D., Gordon, S.M., Moschandreas, D.M., Lebowitz, M.D., 1999. Evaluations of primary metals from NHEXAS Arizona: distributions and preliminary exposures. National Human Exposure Assessment Survey. J Expo Anal Environ Epidemiol 9, 435–445. <https://doi.org/10.1038/sj.jea.7500049>

Oskorouchi, H.R., Nie, P., Sousa-Poza, A., 2018. The effect of floods on anemia among reproductive age women in Afghanistan. PLoS ONE 13, e0191726. <https://doi.org/10.1371/journal.pone.0191726>

Otuneme, O.G., Obebe, O.O., Sajobi, T.T., Akinleye, W.A., Faloye, T.G., 2019. Prevalence of Schistosomiasis in a neglected community, South western Nigeria at two points in time, spaced three years apart. Afr Health Sci 19, 1338–1345. <https://doi.org/10.4314/ahs.v19i1.5>

Ozaydin, N., Turkyilmaz, S.A., Cali, S., 2013. Prevalence and risk factors of Helicobacter pylori in Turkey: a nationally-representative, cross-sectional, screening with the ^13^C-Urea breath test. BMC Public Health 13, 1215. <https://doi.org/10.1186/1471-2458-13-1215>

Özen, A.E., Bibiloni, M.D.M., Bouzas, C., Pons, A., Tur, J.A., 2018. Beverage Consumption among Adults in the Balearic Islands: Association with Total Water and Energy Intake. Nutrients 10, 1149. <https://doi.org/10.3390/nu10091149>

Padula, A.M., Huang, H., Baer, R.J., August, L.M., Jankowska, M.M., Jellife-Pawlowski, L.L., Sirota, M., Woodruff, T.J., 2018. Environmental pollution and social factors as contributors to preterm birth in Fresno County. Environ Health 17, 70. <https://doi.org/10.1186/s12940-018-0414-x>

Pai, M.S., Reshma, Manjula, 2016. A Descriptive Study to Assess the Knowledge and Practice Regarding Water, Sanitation and Hygiene among Women in Selected Villages of Udupi District. Journal of Health and Allied Sciences NU 06, 21–27. <https://doi.org/10.1055/s-0040-1708611>

Pan, X.-B., Wang, H.-J., Zhang, B., Liu, Y.-L., Qi, S.-F., Tian, Q.-B., 2020. Plain Water Intake and Association With the Risk of Overweight in the Chinese Adult Population: China Health and Nutrition Survey 2006–2011. Journal of Epidemiology 30, 128. <https://doi.org/10.2188/jea.JE20180223>

Pandey, R.K., Prajapati, P., Sharma, T., Mandal, C.C., Prajapati, V.K., 2016. Epidemiological investigation of a jaundice outbreak in Kishangarh, Rajasthan, India, 2014. J Public Health 24, 83–89. <https://doi.org/10.1007/s10389-015-0702-7>

Panhwar, A.H., Kazi, T.G., Afridi, H.I., Shaikh, H.R., Arain, S.A., Arain, S.S., Brahman, K.D., 2013. Evaluation of calcium and magnesium in scalp hair samples of population consuming different drinking water: risk of kidney stone. Biol Trace Elem Res 156, 67–73. <https://doi.org/10.1007/s12011-013-9850-1>

Panikkar, B., Lemmond, B., Allen, L., DiPirro, C., Kasper, S., 2019. Making the invisible visible: results of a community-led health survey following PFAS contamination of drinking water in Merrimack, New Hampshire. Environ Health 18, 79. <https://doi.org/10.1186/s12940-019-0513-3>

Parekh, R., Pillai, V., 2016. Stunting in India: An Empirical Approach to Human Rights-Based Solutions. Journal of Human Rights and Social Work 1. <https://doi.org/10.1007/s41134-016-0024-x>

Park, S.K., Peng, Q., Ding, N., Mukherjee, B., Harlow, S.D., 2019. Determinants of per- and polyfluoroalkyl substances (PFAS) in midlife women: Evidence of racial/ethnic and geographic differences in PFAS exposure. Environ Res 175, 186–199. <https://doi.org/10.1016/j.envres.2019.05.028>

Parvez, S., Gerona, R.R., Proctor, C., Friesen, M., Ashby, J.L., Reiter, J.L., Lui, Z., Winchester, P.D., 2018. Glyphosate exposure in pregnancy and shortened gestational length: a prospective Indiana birth cohort study. Environ Health 17, 23. <https://doi.org/10.1186/s12940-018-0367-0>

Parvez, S.M., Azad, R., Rahman, M., Unicomb, L., Ram, P.K., Naser, A.M., Stewart, C.P., Jannat, K., Rahman, M.J., Leontsini, E., Winch, P.J., Luby, S.P., 2018. Achieving optimal technology and behavioral uptake of single and combined interventions of water, sanitation hygiene and nutrition, in an efficacy trial (WASH benefits) in rural Bangladesh. Trials 19, 358. <https://doi.org/10.1186/s13063-018-2710-8>

Patel, A.I., Shapiro, D.J., Wang, Y.C., Cabana, M.D., 2013. Sociodemographic characteristics and beverage intake of children who drink tap water. Am J Prev Med 45, 75–82. <https://doi.org/10.1016/j.amepre.2013.04.001>

Patelarou, E., Kargaki, S., Stephanou, E.G., Nieuwenhuijsen, M., Sourtzi, P., Gracia, E., Chatzi, L., Koutis, A., Kogevinas, M., 2011. Exposure to brominated trihalomethanes in drinking water and reproductive outcomes. Occup Environ Med 68, 438–445. <https://doi.org/10.1136/oem.2010.056150>

Pattanaik, B.K., Singh, K., 2005. Socio-economic conditions of Gaddi tribals: Findings from a survey in Kangra district, Himachal Pradesh. Social Change 35, 13–24. <https://doi.org/10.1177/004908570503500202>

Paul, B.K., 2004. Arsenic contamination awareness among the rural residents in Bangladesh. Social Science & Medicine 59, 1741–1755. <https://doi.org/10.1016/j.socscimed.2004.01.037>

Paul, S.K., Islam, M.S., Hasibuzzaman, M.M., Hossain, F., Anjum, A., Saud, Z.A., Haque, M.M., Sultana, P., Haque, A., Andric, K.B., Rahman, A., Karim, M.R., Siddique, A.E., Karim, Y., Rahman, M., Miyataka, H., Xin, L., Himeno, S., Hossain, K., 2019. Higher risk of hyperglycemia with greater susceptibility in females in chronic arsenic-exposed individuals in Bangladesh. Sci Total Environ 668, 1004–1012. <https://doi.org/10.1016/j.scitotenv.2019.03.029>

Pedersen, M., Mendez, M.A., Schoket, B., Godschalk, R.W., Espinosa, A., Landström, A., Villanueva, C.M., Merlo, D.F., Fthenou, E., Gracia-Lavedan, E., Schooten, F.-J. van, Hoek, G., Brunborg, G., 2015. Environmental, Dietary, Maternal, and Fetal Predictors of Bulky DNA Adducts in Cord Blood: A European Mother-Child Study (NewGeneris) 374. <http://dx.doi.org/10.1289/ehp.1408613>

Pellegriti, G., De Vathaire, F., Scollo, C., Attard, M., Giordano, C., Arena, S., Dardanoni, G., Frasca, F., Malandrino, P., Vermiglio, F., Previtera, D.M., D’Azzò, G., Trimarchi, F., Vigneri, R., 2009. Papillary Thyroid Cancer Incidence in the Volcanic Area of Sicily. JNCI: Journal of the National Cancer Institute 101, 1575–1583. <https://doi.org/10.1093/jnci/djp354>

Peragallo Urrutia, R., Merisier, D., Small, M., Urrutia, E., Tinfo, N., Walmer, D.K., 2012. Unmet health needs identified by Haitian women as priorities for attention: a qualitative study. Reprod Health Matters 20, 93–103. <https://doi.org/10.1016/S0968-8080(12)39602-X>

Pereira, M.D.G.C., Atwill, E.R., Barbosa, A.P., Silva, S.A.E., García-Zapata, M.T.A., 2002. Intra-familial and extra-familial risk factors associated with Cryptosporidium parvum infection among children hospitalized for diarrhea in Goiânia, Goiás, Brazil. Am J Trop Med Hyg 66, 787–793. <https://doi.org/10.4269/ajtmh.2002.66.787>

Perna, S., Alaali, Z., Alalwan, T.A., Janahi, E.M., Mustafa, S., Rondanelli, M., Thani, A.S.B., 2019. A Retrospective Epidemiological Study of the Incidence and Risk Factors of Salmonellosis in Bahrain in Children During 2012^−^2016. Pathogens 8, E51. <https://doi.org/10.3390/pathogens8020051>

Pettit, K.A., Kundert, J.R., VanBuren, J.M., Story, W.T., Buresh, C.T., 2017. Close Together, Far Apart: an Assessment of the Public Health Needs of Three Geographically Proximate Communities in Central Haiti. J Health Care Poor Underserved 28, 739–753. <https://doi.org/10.1353/hpu.2017.0071>

Phan, K., Kim, K.-W., Huoy, L., Phan, S., Se, S., Capon, A.G., Hashim, J.H., 2016. Current status of arsenic exposure and social implication in the Mekong River basin of Cambodia. Environ Geochem Health 38, 763–772. <https://doi.org/10.1007/s10653-015-9759-z>

Phaswana-Mafuya, N., 2006. Hygiene status of rural communities in the Eastern Cape of South Africa. Int J Environ Health Res 16, 289–303. <https://doi.org/10.1080/09603120600734279>

Phipps, K.R., Orwoll, E.S., Bevan, L., 1998. The association between water-borne fluoride and bone mineral density in older adults. J Dent Res 77, 1739–1748. <https://doi.org/10.1177/00220345980770091001>

Phipps, K.R., Orwoll, E.S., Mason, J.D., Cauley, J.A., 2000. Community water fluoridation, bone mineral density, and fractures: prospective study of effects in older women. BMJ 321, 860–864. <https://doi.org/10.1136/bmj.321.7265.860>

Phiri, K., Ndlovu, S., Chiname, T., 2014. Climate Change Impacts on Rural Based Women: Emerging Evidence on Coping and Adaptation Strategies in Tsholotsho, Zimbabwe. MJSS 5, 2545. <https://doi.org/10.5901/mjss.2014.v5n23p2545>

Pi, J., Yamauchi, H., Sun, G., Yoshida, T., Aikawa, H., Fujimoto, W., Iso, H., Cui, R., Waalkes, M.P., Kumagai, Y., 2005. Vascular Dysfunction in Patients with Chronic Arsenosis Can Be Reversed by Reduction of Arsenic Exposure. Environmental Health Perspectives 113, 339–341.

Pickering, A.J., Njenga, S.M., Steinbaum, L., Swarthout, J., Lin, A., Arnold, B.F., Stewart, C.P., Dentz, H.N., Mureithi, M., Chieng, B., Wolfe, M., Mahoney, R., Kihara, J., Byrd, K., Rao, G., Meerkerk, T., Cheruiyot, P., Papaiakovou, M., Pilotte, N., Williams, S.A., Colford, J.M., Null, C., 2019. Effects of single and integrated water, sanitation, handwashing, and nutrition interventions on child soil-transmitted helminth and Giardia infections: A cluster-randomized controlled trial in rural Kenya. PLoS Med 16, e1002841. <https://doi.org/10.1371/journal.pmed.1002841>

Pintar, K.D.M., Waltner-Toews, D., Charron, D., Pollari, F., Fazil, A., McEwen, S.A., Nesbitt, A., Majowicz, S., 2009. Water consumption habits of a south-western Ontario community. J Water Health 7, 276–292. <https://doi.org/10.2166/wh.2009.038>

Pirinççioğlu, A.G., Adıgüzel, S., Özekinci, T., 2018. Seropositivity of Hepatitis A in Children Aged 7–14 Years in Diyarbakir Province Center. Med Sci Monit 24, 936–943. <https://doi.org/10.12659/MSM.906861>

Polissar, L., Severson, R.K., Boatman, E.S., 1984. A case-control study of asbestos in drinking water and cancer risk. Am J Epidemiol 119, 456–471. <https://doi.org/10.1093/oxfordjournals.aje.a113763>

Pompili, M., Vichi, M., Dinelli, E., Erbuto, D., Pycha, R., Serafini, G., Giordano, G., Valera, P., Albanese, S., Lima, A., De Vivo, B., Cicchella, D., Rihmer, Z., Fiorillo, A., Amore, M., Girardi, P., Baldessarini, R.J., 2017. Arsenic: Association of regional concentrations in drinking water with suicide and natural causes of death in Italy. Psychiatry Res 249, 311–317. <https://doi.org/10.1016/j.psychres.2017.01.041>

Pompili, M., Vichi, M., Dinelli, E., Pycha, R., Valera, P., Albanese, S., Lima, A., De Vivo, B., Cicchella, D., Fiorillo, A., Amore, M., Girardi, P., Baldessarini, R.J., 2015. Relationships of local lithium concentrations in drinking water to regional suicide rates in Italy. World J Biol Psychiatry 16, 567–574. <https://doi.org/10.3109/15622975.2015.1062551>

Poreddi, V., Ramachandra, null, Thimmaiah, R., Math, S.B., 2015. Human rights violations among economically disadvantaged women with mental illness: An Indian perspective. Indian J Psychiatry 57, 174–180. <https://doi.org/10.4103/0019-5545.158182>

Pou, S.A., Osella, A.R., Diaz, M.D.P., 2011. Bladder cancer mortality trends and patterns in Córdoba, Argentina (1986-2006). Cancer Causes Control 22, 407–415. <https://doi.org/10.1007/s10552-010-9711-6>

Poureslami, H., Horri, A., Garrusi, B., 2011. A comparative study of the IQ of children age 7–9 in a high and a low fluoride water city in Iran. Fluoride 44, 163–7.

Prasad, N., Jenkins, A.P., Naucukidi, L., Rosa, V., Sahu-Khan, A., Kama, M., Jenkins, K.M., Jenney, A.W.J., Jack, S.J., Saha, D., Horwitz, P., Jupiter, S.D., Strugnell, R.A., Mulholland, E.K., Crump, J.A., 2018. Epidemiology and risk factors for typhoid fever in Central Division, Fiji, 2014-2017: A case-control study. PLoS Negl Trop Dis 12, e0006571. <https://doi.org/10.1371/journal.pntd.0006571>

Psutka, R., Priest, P., Davies, T., Rakunuea, T., Iddings, S., Reiffer, A., 2013. Assessing the demographic, behavioural and environmental characteristics and the potential effectiveness of a household water filter in the Republic of Kiribati. Journal of Water, Sanitation and Hygiene for Development 3, 530–540. <https://doi.org/10.2166/washdev.2013.139>

Puklová, V., Krsková, A., Cerná, M., Cejchanová, M., Rehůrková, I., Ruprich, J., Kratzer, K., Kubínová, R., Zimová, M., 2010. The mercury burden of the Czech population: An integrated approach. Int J Hyg Environ Health 213, 243–251. <https://doi.org/10.1016/j.ijheh.2010.02.002>

Punshon, T., Davis, M.A., Marsit, C.J., Theiler, S.K., Baker, E.R., Jackson, B.P., Conway, D.C., Karagas, M.R., 2015. Placental arsenic concentrations in relation to both maternal and infant biomarkers of exposure in a US cohort. J Expo Sci Environ Epidemiol 25, 599–603. <https://doi.org/10.1038/jes.2015.16>

Qian, N., 2018. Bottled Water or Tap Water? A Comparative Study of Drinking Water Choices on University Campuses. Water 10, 59. <https://doi.org/10.3390/w10010059>

Quist, A.J.L., Inoue-Choi, M., Weyer, P.J., Anderson, K.E., Cantor, K.P., Krasner, S., Freeman, L.E.B., Ward, M.H., Jones, R.R., 2018. Ingested nitrate and nitrite, disinfection by-products, and pancreatic cancer risk in postmenopausal women. Int J Cancer 142, 251–261. <https://doi.org/10.1002/ijc.31055>

Radespiel-Tröger, M., Meyer, M., 2013. Association between drinking water uranium content and cancer risk in Bavaria, Germany. Int Arch Occup Environ Health 86, 767–776. <https://doi.org/10.1007/s00420-012-0806-0>

Rafique, T., Ahmed, I., Soomro, F.Y., Khan, M., Shirin, K., 2015. Fluoride Levels in Urine, Blood Plasma and Serum of People Living in an Endemic Fluorosis Area in the Thar Desert, Pakistan. Journal- Chemical Society of Pakistan 37, 1223–1230.

Rager, J.E., Auerbach, S.S., Chappell, G.A., Martin, E., Thompson, C.M., Fry, R.C., 2017. Benchmark Dose Modeling Estimates of the Concentrations of Inorganic Arsenic That Induce Changes to the Neonatal Transcriptome, Proteome, and Epigenome in a Pregnancy Cohort. Chem. Res. Toxicol. 30, 1911–1920. <https://doi.org/10.1021/acs.chemrestox.7b00221>

Rahman, A., Persson, L.-Å., Nermell, B., El Arifeen, S., Ekström, E.-C., Smith, A.H., Vahter, M., 2010. Arsenic exposure and risk of spontaneous abortion, stillbirth, and infant mortality. Epidemiology 21, 797–804. <https://doi.org/10.1097/EDE.0b013e3181f56a0d>

Rahman, A., Vahter, M., Ekström, E.-C., Persson, L.-Å., 2011. Arsenic exposure in pregnancy increases the risk of lower respiratory tract infection and diarrhea during infancy in Bangladesh. Environ Health Perspect 119, 719–724. <https://doi.org/10.1289/ehp.1002265>

Rahman, A., Vahter, M., Ekström, E.-C., Rahman, M., Golam Mustafa, A.H.M., Wahed, M.A., Yunus, M., Persson, L.-A., 2007. Association of arsenic exposure during pregnancy with fetal loss and infant death: a cohort study in Bangladesh. Am J Epidemiol 165, 1389–1396. <https://doi.org/10.1093/aje/kwm025>

Rahman, M., 2010. Factors associated with complications and hazards during delivery: evidence from Bangladesh Demographic and Health Survey. Jordan Medical Journal 44.

Rahman, M., Sohel, N., Kumar Hore, S., Yunus, M., Bhuiya, A., Kim Streatfield, P., 2015. Prenatal arsenic exposure and drowning among children in Bangladesh. Glob Health Action 8, 10.3402/gha.v8.28702. <https://doi.org/10.3402/gha.v8.28702>

Rahman, M., Sohel, N., Yunus, F.M., Alam, N., Nahar, Q., Streatfield, P.K., Yunus, M., 2019. Arsenic exposure and young adult’s mortality risk: A 13-year follow-up study in Matlab, Bangladesh. Environ Int 123, 358–367. <https://doi.org/10.1016/j.envint.2018.12.006>

Rahman, M., Sohel, N., Yunus, M., Chowdhury, M.E., Hore, S.K., Zaman, K., Bhuiya, A., Streatfield, P.K., 2014. A prospective cohort study of stroke mortality and arsenic in drinking water in Bangladeshi adults. BMC Public Health 14, 174. <https://doi.org/10.1186/1471-2458-14-174>

Rahman, M., Sohel, N., Yunus, M., Chowdhury, M.E., Hore, S.K., Zaman, K., Bhuiya, A., Streatfield, P.K., 2013. Increased Childhood Mortality and Arsenic in Drinking Water in Matlab, Bangladesh: A Population-Based Cohort Study. PLOS ONE 8, e55014. <https://doi.org/10.1371/journal.pone.0055014>

Rahman, M., Vahter, M., Sohel, N., Yunus, M., Wahed, M.A., Streatfield, P.K., Ekström, E.-C., Persson, L.A., 2006a. Arsenic exposure and age and sex-specific risk for skin lesions: a population-based case-referent study in Bangladesh. Environ Health Perspect 114, 1847–1852. <https://doi.org/10.1289/ehp.9207>

Rahman, M., Vahter, M., Wahed, M.A., Sohel, N., Yunus, M., Streatfield, P.K., El Arifeen, S., Bhuiya, A., Zaman, K., Chowdhury, A.M.R., Ekström, E.-C., Persson, L.A., 2006b. Prevalence of arsenic exposure and skin lesions. A population based survey in Matlab, Bangladesh. J Epidemiol Community Health 60, 242–248. <https://doi.org/10.1136/jech.2005.040212>

Rahman, M.L., Kile, M.L., Rodrigues, E.G., Valeri, L., Raj, A., Mazumdar, M., Mostofa, G., Quamruzzaman, Q., Rahman, M., Hauser, R., Baccarelli, A., Liang, L., Christiani, D.C., 2018. Prenatal arsenic exposure, child marriage, and pregnancy weight gain: Associations with preterm birth in Bangladesh. Environ Int 112, 23–32. <https://doi.org/10.1016/j.envint.2017.12.004>

Rahman, M.M., Naidu, R., 2011. Arsenic Exposure from Rice and Water Sources in the Noakhali District of Bangladesh. Water Quality Exposure and Health 3, 1–10. <https://doi.org/10.1007/s12403-010-0034-3>

Rahman, S.M., Åkesson, A., Kippler, M., Grandér, M., Hamadani, J.D., Streatfield, P.K., Persson, L.-Å., Arifeen, S.E., Vahter, M., 2013. Elevated Manganese Concentrations in Drinking Water May Be Beneficial for Fetal Survival. PLOS ONE 8, e74119. <https://doi.org/10.1371/journal.pone.0074119>

Rahman, S.M., Kippler, M., Ahmed, S., Palm, B., El Arifeen, S., Vahter, M., 2015. Manganese exposure through drinking water during pregnancy and size at birth: A prospective cohort study. Reprod Toxicol 53, 68–74. <https://doi.org/10.1016/j.reprotox.2015.03.008>

Rahman, S.M., Kippler, M., Tofail, F., Bölte, S., Hamadani, J.D., Vahter, M., 2017. Manganese in Drinking Water and Cognitive Abilities and Behavior at 10 Years of Age: A Prospective Cohort Study. Environ Health Perspect 125, 057003. <https://doi.org/10.1289/EHP631>

Rajasingham, A., Hardy, C., Kamwaga, S., Sebunya, K., Massa, K., Mulungu, J., Martinsen, A., Nyasani, E., Hulland, E., Russell, S., Blanton, C., Nygren, B., Eidex, R., Handzel, T., 2019. Evaluation of an Emergency Bulk Chlorination Project Targeting Drinking Water Vendors in Cholera-Affected Wards of Dar es Salaam and Morogoro, Tanzania. Am J Trop Med Hyg 100, 1335–1341. <https://doi.org/10.4269/ajtmh.18-0734>

Rajasingham, A., Routh, J.A., Loharikar, A., Chemey, E., Ayers, T., Gunda, A.W., Russo, E.T., Wood, S., Quick, R., 2018. Diffusion of Handwashing Knowledge and Water Treatment Practices From Mothers in an Antenatal Hygiene Promotion Program to Nonpregnant Friends and Relatives, Machinga District, Malawi. Int Q Community Health Educ 39, 63–69. <https://doi.org/10.1177/0272684X18797063>

Rana, B.K., Tripathi, R.M., Sahoo, S.K., Sethy, N.K., Sribastav, V.S., Shukla, A.K., Puranik, V.D., 2010. Assessment of natural uranium and 226Ra concentration in ground water around the uranium mine at Narwapahar, Jharkhand, India and its radiological significance. J Radioanal Nucl Chem 285, 711–717. <https://doi.org/10.1007/s10967-010-0608-3>

Raqib, R., Ahmed, S., Sultana, R., Wagatsuma, Y., Mondal, D., Hoque, A.M.W., Nermell, B., Yunus, M., Roy, S., Persson, L.A., Arifeen, S.E., Moore, S., Vahter, M., 2009. Effects of in utero arsenic exposure on child immunity and morbidity in rural Bangladesh. Toxicol Lett 185, 197–202. <https://doi.org/10.1016/j.toxlet.2009.01.001>

Rasheed, H., Kay, P., Slack, R., Gong, Y.Y., 2019. Assessment of arsenic species in human hair, toenail and urine and their association with water and staple food. J Expo Sci Environ Epidemiol 29, 624–632. <https://doi.org/10.1038/s41370-018-0056-7>

Rauniyar, G., Orbeta, A., Sugiyarto, G., 2011. Impact of water supply and sanitation assistance on human welfare in rural Pakistan. Journal of Development Effectiveness 3, 62–102. <https://doi.org/10.1080/19439342.2010.549947>

Ravenscroft, J., Roy, A., Queirolo, E.I., Mañay, N., Martínez, G., Peregalli, F., Kordas, K., 2018. Drinking water lead, iron and zinc concentrations as predictors of blood lead levels and urinary lead excretion in school children from Montevideo, Uruguay. Chemosphere 212, 694–704. <https://doi.org/10.1016/j.chemosphere.2018.07.154>

Ravichandran, M., Boopati, S., 2005. Environmental, Gender and Institutional Dimensions of Drinking Water Supply: a district level case experience in rural Tamil Nadu. The Indian Journal of Social Work 66, 156–174.

Ravikumar, P., Somashekar, R.K., 2018. Distribution of ^222^ Rn in groundwater and estimation of resulting radiation dose to different age groups: A case study from Bangalore City. Human and Ecological Risk Assessment: An International Journal 24, 174–185. <https://doi.org/10.1080/10807039.2017.1373251>

Razdan, P., Patthi, B., Kumar, J.K., Agnihotri, N., Chaudhari, P., Prasad, M., 2017. Effect of Fluoride Concentration in Drinking Water on Intelligence Quotient of 12–14-Year-Old Children in Mathura District: A Cross-Sectional Study. J Int Soc Prev Community Dent 7, 252–258. <https://doi.org/10.4103/jispcd.JISPCD_201_17>

Redman, S., Ormerod, K., Kelley, S., 2019. Reclaiming Suburbia: Differences in Local Identity and Public Perceptions of Potable Water Reuse. Sustainability 11, 564. <https://doi.org/10.3390/su11030564>

Reeve, N.F., Diggle, P.J., Lamden, K., Keegan, T., 2018. A spatial analysis of giardiasis and cryptosporidiosis in relation to public water supply distribution in North West England. Spat Spatiotemporal Epidemiol 27, 61–70. <https://doi.org/10.1016/j.sste.2018.09.002>

Regnier, A., Gurian, P., Mena, K.D., 2015. Drinking water intake and source patterns within a US-Mexico border population. Int J Environ Health Res 25, 21–32. <https://doi.org/10.1080/09603123.2014.893566>

Rehman, K., Fatima, F., Akash, M.S.H., 2019. Biochemical investigation of association of arsenic exposure with risk factors of diabetes mellitus in Pakistani population and its validation in animal model. Environ Monit Assess 191, 511. <https://doi.org/10.1007/s10661-019-7670-2>

Renaud, J., Gagnon, F., Michaud, C., Boivin, S., 2011. Evaluation of the effectiveness of arsenic screening promotion in private wells: a quasi-experimental study. Health Promot Int 26, 465–475. <https://doi.org/10.1093/heapro/dar013>

Revich, B., Aksel, E., Ushakova, T., Ivanova, I., Zhuchenko, N., Klyuev, N., Brodsky, B., Sotskov, Y., 2001. Dioxin exposure and public health in Chapaevsk, Russia. Chemosphere 43, 951–966. <https://doi.org/10.1016/s0045-6535(00)00456-2>

Rezaei, H., Jafari, A., Kamarehie, B., Fakhri, Y., Ghaderpoury, A., Karami, M.A., Ghaderpoori, M., Shams, M., Bidarpoor, F., Salimi, M., 2019. Health-risk assessment related to the fluoride, nitrate, and nitrite in the drinking water in the Sanandaj, Kurdistan County, Iran. Human and Ecological Risk Assessment: An International Journal 25, 1242–1250. <https://doi.org/10.1080/10807039.2018.1463510>

Rezaienia, S., Nasseri, S., Gholami, M., Farzadkia, M., Esrafili, A., 2019. Performance evaluation of point of use water treatment system in health risk reduction of trace metals in drinking water. DWT 139, 246–253. <https://doi.org/10.5004/dwt.2019.23434>

Rhoads-Baeza, M.E., Reis, J., 2012. An exploratory mixed method assessment of low income, pregnant Hispanic women’s understanding of gestational diabetes and dietary change. Health Education Journal 71, 80–89. <https://doi.org/10.1177/0017896910386287>

Rigas, A.S., Ejsing, B.H., Sørensen, E., Pedersen, O.B., Hjalgrim, H., Erikstrup, C., Ullum, H., 2018. Calcium in drinking water: effect on iron stores in Danish blood donors—results from the Danish Blood Donor Study. Transfusion 58, 1468–1473. <https://doi.org/10.1111/trf.14600>

Righi, E., Bechtold, P., Tortorici, D., Lauriola, P., Calzolari, E., Astolfi, G., Nieuwenhuijsen, M.J., Fantuzzi, G., Aggazzotti, G., 2012. Trihalomethanes, chlorite, chlorate in drinking water and risk of congenital anomalies: a population-based case-control study in Northern Italy. Environ Res 116, 66–73. <https://doi.org/10.1016/j.envres.2012.04.014>

Riley, T.J., Cauley, J.A., Murphy, P.A., 1995. Water chlorination and lipo- and apolipoproteins: the relationship in elderly white women of western Pennsylvania. Am J Public Health 85, 570–573. <https://doi.org/10.2105/ajph.85.4.570>

Rimi Abubakar, I., 2019. Factors influencing household access to drinking water in Nigeria. Utilities Policy 58, 40–51. <https://doi.org/10.1016/j.jup.2019.03.005>

Rinsky, J.L., Hopenhayn, C., Golla, V., Browning, S., Bush, H.M., 2012. Atrazine exposure in public drinking water and preterm birth. Public Health Rep 127, 72–80. <https://doi.org/10.1177/003335491212700108>

Robertson, J.S., 1984. Water sodium, urinary electrolytes, and blood pressure of adolescents. J Epidemiol Community Health 38, 186–194. <https://doi.org/10.1136/jech.38.3.186>

Rochette, L.M., Patterson, S.M., 2005. Hydration status and cardiovascular function: effects of hydration enhancement on cardiovascular function at rest and during psychological stress. Int J Psychophysiol 56, 81–91. <https://doi.org/10.1016/j.ijpsycho.2004.10.003>

Rodrigues, E.G., Kile, M., Dobson, C., Amarasiriwardena, C., Quamruzzaman, Q., Rahman, M., Golam, M., Christiani, D.C., 2015. Maternal-infant biomarkers of prenatal exposure to arsenic and manganese. J Expo Sci Environ Epidemiol 25, 639–648. <https://doi.org/10.1038/jes.2015.45>

Rodríguez-Agudelo, Y., Riojas-Rodríguez, H., Ríos, C., Rosas, I., Sabido Pedraza, E., Miranda, J., Siebe, C., Texcalac, J.L., Santos-Burgoa, C., 2006. Motor alterations associated with exposure to manganese in the environment in Mexico. Sci Total Environ 368, 542–556. <https://doi.org/10.1016/j.scitotenv.2006.03.025>

Rollemberg, C.V.V., Silva, M.M.B.L., Rollemberg, K.C., Amorim, F.R., Lessa, N.M.N., Santos, M.D.S., Souza, A.M.B., Melo, E.V., Almeida, R.P., Silva, Â.M., Werneck, G.L., Santos, M.A., Almeida, J.A.P., Jesus, A.R., 2015. Predicting frequency distribution and influence of sociodemographic and behavioral risk factors of Schistosoma mansoni infection and analysis of co-infection with intestinal parasites. Geospat Health 10, 303. <https://doi.org/10.4081/gh.2015.303>

Röllin, H.B., Kootbodien, T., Theodorou, P., Odland, J.Ø., 2014. Prenatal exposure to manganese in South African coastal communities. Environ. Sci.: Processes Impacts 16, 1903–1912. <https://doi.org/10.1039/C4EM00131A>

Root, E.D., Emch, M.E., 2010. Tracing drinking water to its source: An ecological study of the relationship between textile mills and gastroschisis in North Carolina. Health Place 16, 794–802. <https://doi.org/10.1016/j.healthplace.2010.04.004>

Rosborg, I., Hyllén, E., Lidbeck, J., Nihlgård, B., Gerhardsson, L., 2007. Trace element pattern in patients with fibromyalgia. Sci Total Environ 385, 20–27. <https://doi.org/10.1016/j.scitotenv.2007.05.014>

Rosborg, I., Nihlgård, B., Gerhardsson, L., 2003. Hair element concentrations in females in one acid and one alkaline area in southern Sweden. Ambio 32, 440–446. <https://doi.org/10.1579/0044-7447-32.7.440>

Rosinger, A., Tanner, S., 2015. Water from fruit or the river? Examining hydration strategies and gastrointestinal illness among Tsimane’ adults in the Bolivian Amazon. Public Health Nutr 18, 1098–1108. <https://doi.org/10.1017/S1368980014002158>

Roux, S., Baudoin, C., Boute, D., Brazier, M., De La Guéronniere, V., De Vernejoul, M.C., 2004. Biological effects of drinking-water mineral composition on calcium balance and bone remodeling markers. J Nutr Health Aging 8, 380–384.

Rovira, J., Martínez, M.Á., Sharma, R.P., Espuis, T., Nadal, M., Kumar, V., Costopoulou, D., Vassiliadou, I., Leondiadis, L., Domingo, J.L., Schuhmacher, M., 2019. Prenatal exposure to PFOS and PFOA in a pregnant women cohort of Catalonia, Spain. Environ Res 175, 384–392. <https://doi.org/10.1016/j.envres.2019.05.040>

Roychowdhury, T., 2010. Groundwater arsenic contamination in one of the 107 arsenic-affected blocks in West Bengal, India: Status, distribution, health effects and factors responsible for arsenic poisoning. Int J Hyg Environ Health 213, 414–427. <https://doi.org/10.1016/j.ijheh.2010.09.003>

Roychowdhury, T., Tokunaga, H., Ando, M., 2003. Survey of arsenic and other heavy metals in food composites and drinking water and estimation of dietary intake by the villagers from an arsenic-affected area of West Bengal, India. Sci Total Environ 308, 15–35. <https://doi.org/10.1016/S0048-9697(02)00612-5>

Rubenowitz, E., Axelsson, G., Rylander, R., 1999. Magnesium and calcium in drinking water and death from acute myocardial infarction in women. Epidemiology 10, 31–36.

Rubenowitz, E., Molin, I., Axelsson, G., Rylander, R., 2000. Magnesium in drinking water in relation to morbidity and mortality from acute myocardial infarction. Epidemiology 11, 416–421. <https://doi.org/10.1097/00001648-200007000-00009>

Rubin, R., Pearl, M., Kharrazi, M., Blount, B.C., Miller, M.D., Pearce, E.N., Valentin-Blasini, L., DeLorenze, G., Liaw, J., Hoofnagle, A.N., Steinmaus, C., 2017. Maternal perchlorate exposure in pregnancy and altered birth outcomes. Environ Res 158, 72–81. <https://doi.org/10.1016/j.envres.2017.05.030>

Rudnai, T., Sándor, J., Kádár, M., Borsányi, M., Béres, J., Métneki, J., Maráczi, G., Rudnai, P., 2014. Arsenic in drinking water and congenital heart anomalies in Hungary. Int J Hyg Environ Health 217, 813–818. <https://doi.org/10.1016/j.ijheh.2014.05.002>

Ruíz-Vera, T., Ochoa-Martínez, Á.C., Zarazúa, S., Carrizales-Yáñez, L., Pérez-Maldonado, I.N., 2019. Circulating miRNA-126, -145 and -155 levels in Mexican women exposed to inorganic arsenic via drinking water. Environ Toxicol Pharmacol 67, 79–86. <https://doi.org/10.1016/j.etap.2019.02.004>

Rundblad, G., 2008. The semantics and pragmatics of water notices and the impact on public health. Journal of Water and Health 6, 77–86. <https://doi.org/10.2166/wh.2008.130>

Russ, T.C., Killin, L.O.J., Hannah, J., Batty, G.D., Deary, I.J., Starr, J.M., 2020. Aluminium and fluoride in drinking water in relation to later dementia risk. Br J Psychiatry 216, 29–34. <https://doi.org/10.1192/bjp.2018.287>

Russo, E.T., Sheth, A., Menon, M., Wannemuehler, K., Weinger, M., Kudzala, A.C., Tauzie, B., Masuku, H.D., Msowoya, T.E., Quick, R., 2012. Water treatment and handwashing behaviors among non-pregnant friends and relatives of participants in an antenatal hygiene promotion program in Malawi. Am J Trop Med Hyg 86, 860–865. <https://doi.org/10.4269/ajtmh.2012.11-0259>

Sabbagh, H.J., Alamoudi, N.M., Abdulhameed, F.D., Innes, N.P.T., Al-Aama, J.Y., Hummaida, T., Almalik, M., El Derwi, D.A., Mossey, P.A., 2016. Environmental Risk Factors in the Etiology of Nonsyndromic Orofacial Clefts in the Western Region of Saudi Arabia. Cleft Palate Craniofac J 53, 435–443. <https://doi.org/10.1597/14-136>

Sadeq, M., Moe, C.L., Attarassi, B., Cherkaoui, I., Elaouad, R., Idrissi, L., 2008. Drinking water nitrate and prevalence of methemoglobinemia among infants and children aged 1-7 years in Moroccan areas. Int J Hyg Environ Health 211, 546–554. <https://doi.org/10.1016/j.ijheh.2007.09.009>

Sadler, T.D., Rom, W.N., Lyon, J.L., Mason, J.O., 1984. The use of asbestos-cement pipe for public water supply and the incidence of cancer in selected communities in Utah. J Community Health 9, 285–293. <https://doi.org/10.1007/BF01338728>

Saha, A., Hayen, A., Ali, M., Rosewell, A., Clemens, J.D., Raina MacIntyre, C., Qadri, F., 2017. Socioeconomic risk factors for cholera in different transmission settings: An analysis of the data of a cluster randomized trial in Bangladesh. Vaccine 35, 5043–5049. <https://doi.org/10.1016/j.vaccine.2017.07.021>

Saha, K.K., Engström, A., Hamadani, J.D., Tofail, F., Rasmussen, K.M., Vahter, M., 2012. Pre- and Postnatal Arsenic Exposure and Body Size to 2 Years of Age: A Cohort Study in Rural Bangladesh. Environ Health Perspect 120, 1208–1214. <https://doi.org/10.1289/ehp.1003378>

Saha, P., Paul, B., 2018. Suitability Assessment of Surface Water Quality with Reference to Drinking, Irrigation and Fish Culture: A Human Health Risk Perspective. Bull Environ Contam Toxicol 101, 262–271. <https://doi.org/10.1007/s00128-018-2389-2>

Saha, T., Murhekar, M., Hutin, Y.J., Ramamurthy, T., 2009. An urban, water-borne outbreak of diarrhoea and shigellosis in a district town in eastern India. Natl Med J India 22, 237–239.

Sakamoto, M., 2017. Saline Drinking Water and Salt in Diet: An Approximate Picture of the Situation in a Coastal Area of Southeastern Bangladesh. Int J Disaster Risk Sci 8, 109–120. <https://doi.org/10.1007/s13753-017-0130-0>

Sakketa, T.G., Prowse, M., 2018. Women, Wealth and Waterborne Disease: Smallholders’ Willingness to Pay for a Multiple-Use Water Scheme in Ethiopia. The Journal of Development Studies 54, 426–440. <https://doi.org/10.1080/00220388.2016.1265945>

Samanta, G., Das, D., Mandal, B.K., Chowdhury, T.R., Chakraborti, D., Pal, A., Ahamed, S., 2007. Arsenic in the breast milk of lactating women in arsenic-affected areas of West Bengal, India and its effect on infants. J Environ Sci Health A Tox Hazard Subst Environ Eng 42, 1815–1825. <https://doi.org/10.1080/10934520701566785>

Samiee, F., Leili, M., Faradmal, J., Torkshavand, Z., Asadi, G., 2019. Exposure to arsenic through breast milk from mothers exposed to high levels of arsenic in drinking water: Infant risk assessment. Food Control 106, 106669. <https://doi.org/10.1016/j.foodcont.2019.05.034>

Sanchez, T.R., Slavkovich, V., LoIacono, N., van Geen, A., Ellis, T., Chillrud, S.N., Balac, O., Islam, T., Parvez, F., Ahsan, H., Graziano, J.H., Navas-Acien, A., 2018. Urinary metals and metal mixtures in Bangladesh: Exploring environmental sources in the Health Effects of Arsenic Longitudinal Study (HEALS). Environ Int 121, 852–860. <https://doi.org/10.1016/j.envint.2018.10.031>

Sandoval-Carrillo, A., Méndez-Hernández, E.M., Antuna-Salcido, E.I., Salas-Pacheco, S.M., Vázquez-Alaniz, F., Téllez-Valencia, A., Aguilar-Durán, M., Barraza-Salas, M., Castellanos-Juárez, F.X., La Llave-León, O., Salas-Pacheco, J.M., 2016. Arsenic exposure and risk of preeclampsia in a Mexican mestizo population. BMC Pregnancy Childbirth 16, 153. <https://doi.org/10.1186/s12884-016-0946-4>

Sarkar, A., Hanrahan, M., Hudson, A., 2015. Water insecurity in Canadian Indigenous communities: some inconvenient truths. Rural Remote Health 15, 3354.

Sarker, M.M.R., 2010. Determinants of Arsenicosis Patients’ Perception and Social Implications of Arsenic Poisoning through Groundwater in Bangladesh. Int J Environ Res Public Health 7, 3644–3656. <https://doi.org/10.3390/ijerph7103644>

Sartor, F.A., Rondia, D., 1981. Setting legislative norms for environmental lead exposure: results of an epidemiological survey in the east of Belgium. Toxicol Lett 7, 251–257. <https://doi.org/10.1016/0378-4274(81)90077-1>

Sauvant, M.P., Pepin, D., 2000. Geographic variation of the mortality from cardiovascular disease and drinking water in a French small area (Puy de Dome). Environ Res 84, 219–227. <https://doi.org/10.1006/enrs.2000.4081>

Savitz, D.A., Singer, P.C., Herring, A.H., Hartmann, K.E., Weinberg, H.S., Makarushka, C., 2006. Exposure to drinking water disinfection by-products and pregnancy loss. Am J Epidemiol 164, 1043–1051. <https://doi.org/10.1093/aje/kwj300>

Sawangjang, B., Hashimoto, T., Wongrueng, A., Wattanachira, S., Takizawa, S., 2019. Assessment of fluoride intake from groundwater and intake reduction from delivering bottled water in Chiang Mai Province, Thailand. Heliyon 5, e02391. <https://doi.org/10.1016/j.heliyon.2019.e02391>

Sayli, B.S., Tüccar, E., Elhan, A.H., 1998. An Assessment of Fertility in Boron-exposed Turkish Subpopulations. Reproductive Toxicology 12, 297–304. <https://doi.org/10.1016/S0890-6238(98)00013-6>

Schatz, E., Gilbert, L., 2014. “My Legs Affect Me a Lot. … I Can No Longer Walk to the Forest to Fetch Firewood”: Challenges Related to Health and the Performance of Daily Tasks for Older Women in a High HIV Context. Health Care for Women International 35, 771–788. <https://doi.org/10.1080/07399332.2014.900064>

Scheelbeek, P.F.D., Khan, A.E., Mojumder, S., Elliott, P., Vineis, P., 2016. Drinking Water Sodium and Elevated Blood Pressure of Healthy Pregnant Women in Salinity-Affected Coastal Areas. Hypertension 68, 464–470. <https://doi.org/10.1161/HYPERTENSIONAHA.116.07743>

Schläwicke Engström, K., Broberg, K., Concha, G., Nermell, B., Warholm, M., Vahter, M., 2007. Genetic polymorphisms influencing arsenic metabolism: evidence from Argentina. Environ Health Perspect 115, 599–605. <https://doi.org/10.1289/ehp.9734>

Schöpfer, J., Schrauzer, G.N., 2011. Lithium and other elements in scalp hair of residents of Tokyo Prefecture as investigational predictors of suicide risk. Biol Trace Elem Res 144, 418–425. <https://doi.org/10.1007/s12011-011-9114-x>

Schoppen, S., Pérez-Granados, A.M., Carbajal, A., de la Piedra, C., Pilar Vaquero, M., 2005. Bone remodelling is not affected by consumption of a sodium-rich carbonated mineral water in healthy postmenopausal women. Br J Nutr 93, 339–344. <https://doi.org/10.1079/bjn20041332>

Schroeder, C., Bush, V.E., Norcliffe, L.J., Luft, F.C., Tank, J., Jordan, J., Hainsworth, R., 2002. Water drinking acutely improves orthostatic tolerance in healthy subjects. Circulation 106, 2806–2811. <https://doi.org/10.1161/01.cir.0000038921.64575.d0>

Schultz, I.R., Shangraw, R.E., 2006. Effect of short-term drinking water exposure to dichloroacetate on its pharmacokinetics and oral bioavailability in human volunteers: a stable isotope study. Toxicol Sci 92, 42–50. <https://doi.org/10.1093/toxsci/kfj193>

Schwanz, T.G., Llorca, M., Farré, M., Barceló, D., 2016. Perfluoroalkyl substances assessment in drinking waters from Brazil, France and Spain. Sci Total Environ 539, 143–152. <https://doi.org/10.1016/j.scitotenv.2015.08.034>

Schwartz, G.G., Klug, M.G., 2016. Motor neuron disease mortality rates in U.S. states are associated with well water use. Amyotroph Lateral Scler Frontotemporal Degener 17, 528–534. <https://doi.org/10.1080/21678421.2016.1195409>

Seid, H., Kumie, A., 2013. The status of school sanitation facilities in some selected primary and secondary schools in Dessie City Administration, South Wello Zone, Amhara Region. Ethiopian Journal of Health Development 27, 80–84. <https://doi.org/10.4314/ejhd.v27i1>

Seifert, B., Becker, K., Hoffmann, K., Krause, C., Schulz, C., 2000. The German Environmental Survey 1990/1992 (GerES II): a representative population study. J Expo Anal Environ Epidemiol 10, 103–114. <https://doi.org/10.1038/sj.jea.7500075>

Seldén, A.I., Lundholm, C., Edlund, B., Högdahl, C., Ek, B.-M., Bergström, B.E., Ohlson, C.-G., 2009. Nephrotoxicity of uranium in drinking water from private drilled wells. Environ Res 109, 486–494. <https://doi.org/10.1016/j.envres.2009.02.002>

Sen, J., Chaudhuri, A.B.D., 2008. Arsenic exposure through drinking water and its effect on pregnancy outcome in Bengali women. Arh Hig Rada Toksikol 59, 271–275. <https://doi.org/10.2478/10004-1254-59-2008-1871>

Sen, J., Chaudhuri, A.B.D., 2007. Effect of arsenic on the onset of menarcheal age. Bull Environ Contam Toxicol 79, 293–296. <https://doi.org/10.1007/s00128-007-9206-7>

Senterre, C., Dramaix, M., Thiébaut, I., 2014. Fluid intake survey among schoolchildren in Belgium. BMC Public Health 14, 651. <https://doi.org/10.1186/1471-2458-14-651>

Ser, P.H., Banu, B., Jebunnesa, F., Fatema, K., Rosy, N., Yasmin, R., Furusawa, H., Ali, L., Ahmad, S.A., Watanabe, C., 2015. Arsenic exposure increases maternal but not cord serum IgG in Bangladesh. Pediatr Int 57, 119–125. <https://doi.org/10.1111/ped.12396>

Serretta, V., Altieri, V., Morgia, G., Allegro, R., Ruggirello, A., Lallo, A.D., Carrieri, G., Melloni, D., 2009. Cigarette Smoking and Drinking Water Source: Correlation with Clinical Features and Pathology of Superficial Bladder Carcinoma. UIN 82, 318–323. <https://doi.org/10.1159/000209365>

Shaheen, N., Hassan, M., Mahmood, Q., Hayat, Y., 2015. Maternal and fetal blood lead concentrations under non-occupational lead exposure and associated factors in Pakistan. Toxicological & Environmental Chemistry 97, 828–837. <https://doi.org/10.1080/02772248.2015.1060726>

Shaikh, A.A., Khan, S., Thebo, J.A., Lohdi, N., Korejo, A.A., Khaskheli, M.H., Khaskheli, M.I., 2018. Nutritional status and caloric consumption of school going children of Hyderabad having anemic features -. Rawal Medical Journal 43, 337–340.

Shakya, B., Shrestha, S., Madhikarmi, N.L., Adhikari, R., 2012. Intestinal parasitic infection among school children. J Nepal Health Res Counc 10, 20–23.

Shannon, J., White, E., Shattuck, A.L., Potter, J.D., 1996. Relationship of food groups and water intake to colon cancer risk. Cancer Epidemiol Biomarkers Prev 5, 495–502.

Sharma, P.K., Ramakrishnan, R., Hutin, Y., Manickam, P., Gupte, M.D., 2009. Risk factors for typhoid in Darjeeling, West Bengal, India: evidence for practical action. Trop Med Int Health 14, 696–702. <https://doi.org/10.1111/j.1365-3156.2009.02283.x>

Shekar, B.R.C., Suma, S., Kumar, S., Sukhabogi, J.R., Manjunath, B.C., 2013. Malocclusion status among 15 years old adolescents in relation to fluoride concentration and area of residence. Indian J Dent Res 24, 1–7. <https://doi.org/10.4103/0970-9290.114910>

Sherlock, J.C., Ashby, D., Delves, H.T., Forbes, G.I., Moore, M.R., Patterson, W.J., Pocock, S.J., Quinn, M.J., Richards, W.N., Wilson, T.S., 1984. Reduction in exposure to lead from drinking water and its effect on blood lead concentrations. Hum Toxicol 3, 383–392. <https://doi.org/10.1177/096032718400300503>

Sheth, A.N., Russo, E.T., Menon, M., Wannemuehler, K., Weinger, M., Kudzala, A.C., Tauzie, B., Masuku, H.D., Msowoya, T.E., Quick, R., 2010. Impact of the integration of water treatment and handwashing incentives with antenatal services on hygiene practices of pregnant women in Malawi. Am J Trop Med Hyg 83, 1315–1321. <https://doi.org/10.4269/ajtmh.2010.10-0211>

Shi, X., Ayotte, J.D., Onda, A., Miller, S., Rees, J., Gilbert-Diamond, D., Onega, T., Gui, J., Karagas, M., Moeschler, J., 2015. Geospatial association between adverse birth outcomes and arsenic in groundwater in New Hampshire, USA. Environ Geochem Health 37, 333–351. <https://doi.org/10.1007/s10653-014-9651-2>

Shih, Y.-H., Argos, M., Turyk, M.E., 2019. Urinary arsenic concentration, airway inflammation, and lung function in the U.S. adult population. Environ Res 175, 308–315. <https://doi.org/10.1016/j.envres.2019.05.031>

Shih, Y.-H., Islam, T., Hore, S.K., Sarwar, G., Shahriar, M.H., Yunus, M., Graziano, J.H., Harjes, J., Baron, J.A., Parvez, F., Ahsan, H., Argos, M., 2017. Associations between prenatal arsenic exposure with adverse pregnancy outcome and child mortality. Environ Res 158, 456–461. <https://doi.org/10.1016/j.envres.2017.07.004>

Shiotsuki, I., Terao, T., Ishii, N., Takeuchi, S., Kuroda, Y., Kohno, K., Mizokami, Y., Hatano, K., Tanabe, S., Kanehisa, M., Iwata, N., Matusda, S., 2016. Trace lithium is inversely associated with male suicide after adjustment of climatic factors. J Affect Disord 189, 282–286. <https://doi.org/10.1016/j.jad.2015.09.070>

Shrestha, P., Shrestha, D., Magar, D.T., Rai, G., Rai, K.R., Rai, S.K., 2019. Intestinal Parasitic Infections among Prison Inmates in Kathmandu Nepal. J Nepal Health Res Counc 17, 382–387. <https://doi.org/10.33314/jnhrc.v17i3.2015>

Sichert-Hellert, W., Kersting, M., 2004. Home-made carbonated water and the consumption of water and other beverages in children and adolescents: results of the DONALD study. Acta Paediatr 93, 1583–1587. <https://doi.org/10.1080/08035250410033925>

Signes-Pastor, A.J., Bouchard, M.F., Baker, E., Jackson, B.P., Karagas, M.R., 2019. Toenail manganese as biomarker of drinking water exposure: a reliability study from a US pregnancy cohort. J Expo Sci Environ Epidemiol 29, 648–654. <https://doi.org/10.1038/s41370-018-0108-z>

Signes-Pastor, A.J., Mitra, K., Sarkhel, S., Hobbes, M., Burló, F., de Groot, W.T., Carbonell-Barrachina, A.A., 2008. Arsenic speciation in food and estimation of the dietary intake of inorganic arsenic in a rural village of West Bengal, India. J Agric Food Chem 56, 9469–9474. <https://doi.org/10.1021/jf801600j>

Sigurdson, E.E., Levy, B.S., Mandel, J., McHugh, R., Michienzi, L.J., Jagger, H., Pearson, J., 1981. Cancer morbidity investigations: Lessons from the Duluth study of possible effects of asbestos in drinking water. Environmental Research 25, 50–61. <https://doi.org/10.1016/0013-9351(81)90079-7>

Simchen, E., Jeeraphat, S., Shihab, S., Fattal, B., 1991. An epidemic of waterborne Shigella gastroenteritis in Kibbutzim of western Galilee in Israel. Int J Epidemiol 20, 1081–1087. <https://doi.org/10.1093/ije/20.4.1081>

Sindhura, M., Vallepalli, C., Madhavi, B., Appalanaidu, S., 2018. An assessment of water, sanitation and hygiene practices in an Urban slum of Visakhapatnam, Andhra Pradesh. Indian Journal of Public Health Research & Development 9, 26. <https://doi.org/10.5958/0976-5506.2018.00689.7>

Singh, A., Banerjee, T., Kumar, R., Shukla, S.K., 2019. Prevalence of cases of amebic liver abscess in a tertiary care centre in India: A study on risk factors, associated microflora and strain variation of Entamoeba histolytica. PLoS One 14, e0214880. <https://doi.org/10.1371/journal.pone.0214880>

Singh, B., Kataria, N., Garg, V.K., Yadav, P., Kishore, N., Pulhani, V., 2014. Uranium quantification in groundwater and health risk from its ingestion in Haryana, India. Toxicological & Environmental Chemistry 96, 1571–1580. <https://doi.org/10.1080/02772248.2015.1025787>

Singh, S., Sinwal, N., Rathore, H., 2012. Gender involvement in manual material handling (mmh) tasks in agriculture and technology intervention to mitigate the resulting musculoskeletal disorders. Work 41, 4333–4341. <https://doi.org/10.3233/WOR-2012-0728-4333>

Sinha, D., Mukherjee, B., Bindhani, B., Dutta, K., Saha, H., Prasad, P., Ray, M.R., 2014. Chronic Low Level Arsenic Exposure Inflicts Pulmonary and Systemic Inflammation. Cancer Science & Therapy 6, 1–8. <https://doi.org/10.4172/1948-5956.1000250>

Skröder, H., Hawkesworth, S., Kippler, M., El Arifeen, S., Wagatsuma, Y., Moore, S.E., Vahter, M., 2015. Kidney function and blood pressure in preschool-aged children exposed to cadmium and arsenic - potential alleviation by selenium. Environmental Research 140, 205–213. <https://doi.org/10.1016/j.envres.2015.03.038>

Smith, A.H., Arroyo, A.P., Mazumder, D.N., Kosnett, M.J., Hernandez, A.L., Beeris, M., Smith, M.M., Moore, L.E., 2000. Arsenic-induced skin lesions among Atacameño people in Northern Chile despite good nutrition and centuries of exposure. Environ Health Perspect 108, 617–620. <https://doi.org/10.1289/ehp.00108617>

Smith, A.H., Goycolea, M., Haque, R., Biggs, M.L., 1998. Marked Increase in Bladder and Lung Cancer Mortality in a Region of Northern Chile Due to Arsenic in Drinking Water. American Journal of Epidemiology 147, 660–669. <https://doi.org/10.1093/oxfordjournals.aje.a009507>

Smith, A.H., Marshall, G., Roh, T., Ferreccio, C., Liaw, J., Steinmaus, C., 2017. Lung, Bladder, and Kidney Cancer Mortality 40 Years After Arsenic Exposure Reduction. J Natl Cancer Inst 110, 241–249. <https://doi.org/10.1093/jnci/djx201>

Smith, A.H., Marshall, G., Yuan, Y., Liaw, J., Ferreccio, C., Steinmaus, C., 2011. Evidence from Chile that arsenic in drinking water may increase mortality from pulmonary tuberculosis. Am J Epidemiol 173, 414–420. <https://doi.org/10.1093/aje/kwq383>

Smith, A.H., Marshall, G., Yuan, Y., Steinmaus, C., Liaw, J., Smith, M.T., Wood, L., Heirich, M., Fritzemeier, R.M., Pegram, M.D., Ferreccio, C., 2014. Rapid Reduction in Breast Cancer Mortality With Inorganic Arsenic in Drinking Water. EBioMedicine 1, 58–63. <https://doi.org/10.1016/j.ebiom.2014.10.005>

Smith, R.B., Edwards, S.C., Best, N., Wright, J., Nieuwenhuijsen, M.J., Toledano, M.B., 2016. Birth Weight, Ethnicity, and Exposure to Trihalomethanes and Haloacetic Acids in Drinking Water during Pregnancy in the Born in Bradford Cohort. Environmental Health Perspectives 124, 681–689. <https://doi.org/10.1289/ehp.1409480>

Smith, R.B., Toledano, M.B., Wright, J., Raynor, P., Nieuwenhuijsen, M.J., 2009. Tap water use amongst pregnant women in a multi-ethnic cohort. Environ Health 8, S7. <https://doi.org/10.1186/1476-069X-8-S1-S7>

Sohel, N., Kanaroglou, P.S., Persson, L.A., Haq, M.Z., Rahman, M., Vahter, M., 2010. Spatial modelling of individual arsenic exposure via well water: evaluation of arsenic in urine, main water source and influence of neighbourhood water sources in rural Bangladesh. J Environ Monit 12, 1341–1348. <https://doi.org/10.1039/c001708f>

Sohel Rana, M.D., 2009. Status of water use sanitation and hygienic condition of urban slums: A study on Rupsha Ferighat slum, Khulna. Desalination 246, 322–328. <https://doi.org/10.1016/j.desal.2008.04.052>

Sohn, W., Noh, H., Burt, B.A., 2009. Fluoride ingestion is related to fluid consumption patterns. J Public Health Dent 69, 267–275. <https://doi.org/10.1111/j.1752-7325.2009.00133.x>

Son, B.C., Lee, C.K., Suh, C.H., Kim, K.H., Kim, J.H., Jeong, S.U., Kim, D.H., Ryu, J.Y., Lee, S.-W., Kim, S.J., Kwon, Y.M., Park, Y.B., 2019. Blood lead concentration and exposure related factors in Korea from the National Environmental Health Survey (KoNEHS) II (2012-2014). J Occup Environ Hyg 16, 763–774. <https://doi.org/10.1080/15459624.2019.1668000>

Song, T., Chen, Y., Du, S., Yang, F., 2017. Hydrogeochemical evolution and risk assessment of human health in a riverbank filtration site, northeastern China. Human and Ecological Risk Assessment: An International Journal 23, 705–726. <https://doi.org/10.1080/10807039.2016.1277413>

Sorvillo, F., Beall, G., Turner, P.A., Beer, V.L., Kovacs, A.A., Kraus, P., Masters, D., Kerndt, P.R., 1998. Seasonality and factors associated with cryptosporidiosis among individuals with HIV infection. Epidemiol Infect 121, 197–204. <https://doi.org/10.1017/s0950268898001009>

Sowers, M., Whitford, G.M., Clark, M.K., Jannausch, M.L., 2005. Elevated serum fluoride concentrations in women are not related to fractures and bone mineral density. J Nutr 135, 2247–2252. <https://doi.org/10.1093/jn/135.9.2247>

Sowers, M.R., Wallace, R.B., Lemke, J.H., 1986. The relationship of bone mass and fracture history to fluoride and calcium intake: a study of three communities. Am J Clin Nutr 44, 889–898. <https://doi.org/10.1093/ajcn/44.6.889>

Sripaoraya, K., Siriwong, W., Pavittranon, S., Chapman, R.S., 2017. Environmental arsenic exposure and risk of diabetes type 2 in Ron Phibun subdistrict, Nakhon Si Thammarat Province, Thailand: unmatched and matched case-control studies. Risk Manag Healthc Policy 10, 41–48. <https://doi.org/10.2147/RMHP.S128277>

Sroka, J., Wojcik-Fatla, A., Szymanska, J., Dutkiewicz, J., Zajac, V., Zwolinski, J., 2010. The occurrence of Toxoplasma gondii infection in people and animals from rural environment of Lublin region - estimate of potential role of water as a source of infection. Ann Agric Environ Med 17, 125–132.

Ssemugabo, C., Wafula, S.T., Ndejjo, R., Oporia, F., Osuret, J., Musoke, D., Halage, A.A., 2019. Knowledge and practices of households on safe water chain maintenance in a slum community in Kampala City, Uganda. Environmental Health and Preventive Medicine 24, 45. <https://doi.org/10.1186/s12199-019-0799-3>

Stajnko, A., Šlejkovec, Z., Mazej, D., France-Štiglic, A., Briški, A.S., Prpić, I., Špirić, Z., Horvat, M., Falnoga, I., 2019. Arsenic metabolites; selenium; and AS3MT, MTHFR, AQP4, AQP9, SELENOP, INMT, and MT2A polymorphisms in Croatian-Slovenian population from PHIME-CROME study. Environmental Research 170, 301–319. <https://doi.org/10.1016/j.envres.2018.11.045>

Stein, C.R., Savitz, D.A., Bellinger, D.C., 2014. Perfluorooctanoate exposure in a highly exposed community and parent and teacher reports of behaviour in 6-12-year-old children. Paediatr Perinat Epidemiol 28, 146–156. <https://doi.org/10.1111/ppe.12097>

Steinmaus, C., Castriota, F., Ferreccio, C., Smith, A.H., Yuan, Y., Liaw, J., Acevedo, J., Pérez, L., Meza, R., Calcagno, S., Uauy, R., Smith, M.T., 2015. Obesity and excess weight in early adulthood and high risks of arsenic-related cancer in later life. Environ Res 142, 594–601. <https://doi.org/10.1016/j.envres.2015.07.021>

Steinmaus, C., Ferreccio, C., Acevedo, J., Yuan, Y., Liaw, J., Durán, V., Cuevas, S., García, J., Meza, R., Valdés, R., Valdés, G., Benítez, H., VanderLinde, V., Villagra, V., Cantor, K.P., Moore, L.E., Perez, S.G., Steinmaus, S., Smith, A.H., 2014. Increased lung and bladder cancer incidence in adults after in utero and early-life arsenic exposure. Cancer Epidemiol Biomarkers Prev 23, 1529–1538. <https://doi.org/10.1158/1055-9965.EPI-14-0059>

Steinmaus, C.M., Ferreccio, C., Romo, J.A., Yuan, Y., Cortes, S., Marshall, G., Moore, L.E., Balmes, J.R., Liaw, J., Golden, T., Smith, A.H., 2013. Drinking water arsenic in northern chile: high cancer risks 40 years after exposure cessation. Cancer Epidemiol Biomarkers Prev 22, 623–630. <https://doi.org/10.1158/1055-9965.EPI-12-1190>

Stevenson, E.G.J., Greene, L.E., Maes, K.C., Ambelu, A., Tesfaye, Y.A., Rheingans, R., Hadley, C., 2012. Water insecurity in 3 dimensions: an anthropological perspective on water and women’s psychosocial distress in Ethiopia. Soc Sci Med 75, 392–400. <https://doi.org/10.1016/j.socscimed.2012.03.022>

Sthiannopkao, S., Kim, K.-W., Cho, K.H., Wantala, K., Sotham, S., Sokuntheara, C., Kim, J.H., 2010. Arsenic levels in human hair, Kandal Province, Cambodia: The influences of groundwater arsenic, consumption period, age and gender. Applied Geochemistry 25, 81–90. <https://doi.org/10.1016/j.apgeochem.2009.10.003>

Stoler, J., Fink, G., Weeks, J.R., Otoo, R.A., Ampofo, J.A., Hill, A.G., 2012. When urban taps run dry: sachet water consumption and health effects in low income neighborhoods of Accra, Ghana. Health Place 18, 250–262. <https://doi.org/10.1016/j.healthplace.2011.09.020>

Stookey, J.D., 2019. Analysis of 2009–2012 Nutrition Health and Examination Survey (NHANES) Data to Estimate the Median Water Intake Associated with Meeting Hydration Criteria for Individuals Aged 12–80 Years in the US Population. Nutrients 11, 657. <https://doi.org/10.3390/nu11030657>

Stubleski, J., Salihovic, S., Lind, L., Lind, P.M., van Bavel, B., Kärrman, A., 2016. Changes in serum levels of perfluoroalkyl substances during a 10-year follow-up period in a large population-based cohort. Environ Int 95, 86–92. <https://doi.org/10.1016/j.envint.2016.08.002>

Su, X., Wang, H., Zhang, Y., 2013. Health Risk Assessment of Nitrate Contamination in Groundwater: A Case Study of an Agricultural Area in Northeast China. Water Resour Manage 27, 3025–3034. <https://doi.org/10.1007/s11269-013-0330-3>

Suarez-Almazor, M.E., Flowerdew, G., Saunders, L.D., Soskolne, C.L., Russell, A.S., 1993. The fluoridation of drinking water and hip fracture hospitalization rates in two Canadian communities. Am J Public Health 83, 689–693. <https://doi.org/10.2105/ajph.83.5.689>

Subbaraman, R., Nolan, L., Sawant, K., Shitole, S., Shitole, T., Nanarkar, M., Patil-Deshmukh, A., Bloom, D.E., 2015. Multidimensional Measurement of Household Water Poverty in a Mumbai Slum: Looking Beyond Water Quality. PLoS One 10, e0133241. <https://doi.org/10.1371/journal.pone.0133241>

Sucilathangam, G., Anna, T., 2016. Seroepidemiological study of toxoplasmosis in southern districts of Tamil Nadu. J Parasit Dis 40, 381–386. <https://doi.org/10.1007/s12639-014-0514-7>

Suetens, C., Moreno-Reyes, R., Chasseur, C., Mathieu, F., Begaux, F., Haubruge, E., Durand, M.C., Nève, J., Vanderpas, J., 2001. Epidemiological support for a multifactorial aetiology of Kashin-Beck disease in Tibet. Int Orthop 25, 180–187. <https://doi.org/10.1007/s002640100247>

Sugawara, N., Yasui-Furukori, N., Ishii, N., Iwata, N., Terao, T., 2013. Lithium in tap water and suicide mortality in Japan. Int J Environ Res Public Health 10, 6044–6048. <https://doi.org/10.3390/ijerph10116044>

Summerhayes, R.J., Morgan, G.G., Edwards, H.P., Lincoln, D., Earnest, A., Rahman, B., Beard, J.R., 2012. Exposure to trihalomethanes in drinking water and small-for-gestational-age births. Epidemiology 23, 15–22. <https://doi.org/10.1097/EDE.0b013e31823b669b>

Sun, G., Xu, Y., Li, X., Jin, Y., Li, B., Sun, X., 2007. Urinary arsenic metabolites in children and adults exposed to arsenic in drinking water in Inner Mongolia, China. Environ Health Perspect 115, 648–652. <https://doi.org/10.1289/ehp.9271>

Suomi, J., Tuominen, P., Niinistö, S., Virtanen, S.M., Savela, K., Suomi, J., Tuominen, P., Niinistö, S., Virtanen, S.M., Savela, K., 2019. Dietary heavy metal exposure of Finnish 1-year-olds. AIMSAGRI 4, 778–793. <https://doi.org/10.3934/agrfood.2019.3.778>

Surdu, S., Bloom, M.S., Neamtiu, I.A., Pop, C., Anastasiu, D., Fitzgerald, E.F., Gurzau, E.S., 2015. Consumption of arsenic-contaminated drinking water and anemia among pregnant and non-pregnant women in northwestern Romania. Environ Res 140, 657–660. <https://doi.org/10.1016/j.envres.2015.05.020>

Susko, M.L., Bloom, M.S., Neamtiu, I.A., Appleton, A.A., Surdu, S., Pop, C., Fitzgerald, E.F., Anastasiu, D., Gurzau, E.S., 2017. Low-level arsenic exposure via drinking water consumption and female fecundity - A preliminary investigation. Environ Res 154, 120–125. <https://doi.org/10.1016/j.envres.2016.12.030>

Sutrisna, A., Knowles, J., Basuni, A., Menon, R., Sugihantono, A., 2018. Iodine Intake Estimation from the Consumption of Instant Noodles, Drinking Water and Household Salt in Indonesia. Nutrients 10, E324. <https://doi.org/10.3390/nu10030324>

Swan, S.H., Waller, K., Hopkins, B., Windham, G., Fenster, L., Schaefer, C., Neutra, R.R., 1998. A prospective study of spontaneous abortion: relation to amount and source of drinking water consumed in early pregnancy. Epidemiology 9, 126–133.

Syed, E.H., Poudel, K.C., Sakisaka, K., Yasuoka, J., Ahsan, H., Jimba, M., 2012. Quality of Life and Mental Health Status of Arsenic-affected Patients in a Bangladeshi Population. J Health Popul Nutr 30, 262–269. <https://doi.org/10.3329/jhpn.v30i3.12289>

Tambe, M., Patil, S., M, D., Bhagwat, V., 2015. Investigation of an Outbreak of Hepatitis’E’ in a Rural Area of Dhule District in Maharashtra. JKIMSU 4, 109–114.

Tan, H.-Z., Lin, W.-J., Huang, J.-Q., Dai, M., Fu, J.-H., Huang, Q.-H., Chen, W.-M., Xu, Y.-L., Ye, T.-T., Lin, Z.-Y., Lin, X.-S., Cai, J.-X., Dong, Y.-H., Luo, H.-Y., Chen, S.-H., Huang, Y.-L., Yang, J., Lin, A.-X., Yuan, X.-Q., Chen, S.-Y., Wang, K.-S., Zhuang, C.-Y., Wang, S.-C., Lin, L.-L., Zou, X.-F., Song, Z.-H., Fang, X.-H., Chen, T., Zhang, J.-H., Li, K.-Q., Chen, L.-H., Lin, X.-P., Lin, J.-M., Lin, J.-N., Lin, P.-L., Chen, J.-T., Lin, K.-M., Hong, X.-C., Wang, L.-D., Xu, L.-Y., Li, E.-M., Zhang, J.-J., 2017. Updated incidence rates and risk factors of esophageal cancer in Nan’ao Island, a coastal high-risk area in southern China. Dis Esophagus 30, 1–7. <https://doi.org/10.1111/dote.12468>

Tan, L., Sang, Z., Shen, J., Liu, H., Chen, W., Zhao, N., Wei, W., Zhang, G., Zhang, W., 2015. Prevalence of thyroid dysfunction with adequate and excessive iodine intake in Hebei Province, People’s Republic of China. Public Health Nutr 18, 1692–1697. <https://doi.org/10.1017/S1368980014002237>

Tan, R., Suparmanto, S., Warner, G., Putri, M.P., Corwin, A., Hyams, K.C., Graham, R., Winarno, J., Laras, K., Lubis, I., Master, J., Sumardiati, A., Wignall, F.S., 1997. Epidemic and Sporadic Hepatitis E Virus Transmission in West Kalimantan (Borneo), Indonesia. The American Journal of Tropical Medicine and Hygiene 57, 62–65. <https://doi.org/10.4269/ajtmh.1997.57.62>

Tanaka, Y., Sakata, Y., Hara, M., Kawakubo, H., Tsuruoka, N., Yamamoto, K., Itoh, Y., Hidaka, H., Shimoda, R., Iwakiri, R., Fujimoto, K., 2017. Risk Factors for Helicobacter pylori Infection and Endoscopic Reflux Esophagitis in Healthy Young Japanese Volunteers. Intern Med 56, 2979–2983. <https://doi.org/10.2169/internalmedicine.8669-16>

Tasian, G.E., Ross, M., Song, L., Audrain-McGovern, J., Wiebe, D., Warner, S.G., Henderson, B., Patel, A., Furth, S.L., 2019. Ecological Momentary Assessment of Factors Associated with Water Intake among Adolescents with Kidney Stone Disease. J Urol 201, 606–614. <https://doi.org/10.1016/j.juro.2018.07.064>

Taskeen, A., Naeem, I., Atif, M., 2012. Evaluation of blood bisphenol A contents: a case study. West Indian Med J 61, 564–568.

Tauheed, J., Sanchez-Guerra, M., Lee, J.J., Paul, L., Ibne Hasan, M.O.S., Quamruzzaman, Q., Selhub, J., Wright, R.O., Christiani, D.C., Coull, B.A., Baccarelli, A.A., Mazumdar, M., 2017. Associations between post translational histone modifications, myelomeningocele risk, environmental arsenic exposure, and folate deficiency among participants in a case control study in Bangladesh. Epigenetics 12, 484–491. <https://doi.org/10.1080/15592294.2017.1312238>

Tayeh, A., Cairncross, S., Maude, G.H., 1993. Water sources and other determinants of dracunculiasis in the northern region of Ghana. J Helminthol 67, 213–225. <https://doi.org/10.1017/s0022149x00013158>

Tayyem, R.F., Shehadeh, I.N., Abumweis, S.S., Bawadi, H.A., Hammad, S.S., Bani-Hani, K.E., Al-Jaberi, T.M., Alnusair, M.M., 2013. Physical inactivity, water intake and constipation as risk factors for colorectal cancer among adults in Jordan. Asian Pac J Cancer Prev 14, 5207–5212. <https://doi.org/10.7314/apjcp.2013.14.9.5207>

Teh, B.H., Lin, J.T., Pan, W.H., Lin, S.H., Wang, L.Y., Lee, T.K., Chen, C.J., 1994. Seroprevalence and associated risk factors of Helicobacter pylori infection in Taiwan. Anticancer Res 14, 1389–1392.

Tekalign, E., Bajiro, M., Mio Ayana, Tiruneh, A., Tariku Belay, 2019. Prevalence and Intensity of Soil-Transmitted Helminth Infection among Rural Community of Southwest Ethiopia: A Community-Based Study. BioMed Research International 2019, 7. <https://doi.org/10.1155/2019/3687873>

Téllez Téllez, R., Michaud Chacón, P., Reyes Abarca, C., Blount, B.C., Van Landingham, C.B., Crump, K.S., Gibbs, J.P., 2005. Long-term environmental exposure to perchlorate through drinking water and thyroid function during pregnancy and the neonatal period. Thyroid 15, 963–975. <https://doi.org/10.1089/thy.2005.15.963>

Temmar, M., Labat, C., Benkhedda, S., Charifi, M., Thomas, F., Bouafia, M.T., Bean, K., Darne, B., Safar, M.E., Benetos, A., 2007. Prevalence and determinants of hypertension in the Algerian Sahara. J Hypertens 25, 2218–2226. <https://doi.org/10.1097/HJH.0b013e3282dc7549>

Teschke, K., Bellack, N., Shen, H., Atwater, J., Chu, R., Koehoorn, M., MacNab, Y.C., Schreier, H., Isaac-Renton, J.L., 2010. Water and sewage systems, socio-demographics, and duration of residence associated with endemic intestinal infectious diseases: a cohort study. BMC Public Health 10, 767. <https://doi.org/10.1186/1471-2458-10-767>

Thakur, A., Rao, R., Sachdeva, A., 2017. Epidemiological Investigation of Hepatitis E outbreak in a Northern city of India. Journal of Advanced Medical and Dental Sciences Research 5, 29–36. <https://doi.org/10.21276/jamdsr.2017.5.8.09>

Thakur, B., Gupta, V., 2016. Arsenic concentration in drinking water of Bihar: health issues and socio-economic problems. Journal of Water, Sanitation and Hygiene for Development 6. <https://doi.org/10.2166/washdev.2016.047>

Thompson, J., Lorber, M., Toms, L.-M.L., Kato, K., Calafat, A.M., Mueller, J.F., 2010. Use of simple pharmacokinetic modeling to characterize exposure of Australians to perfluorooctanoic acid and perfluorooctane sulfonic acid. Environ Int 36, 390–397. <https://doi.org/10.1016/j.envint.2010.02.008>

Tigabu, E., Petros, B., Endeshaw, T., 2010. Prevalence of Giardiasis and Cryptosporidiosis among children in relation to water sources in Selected Village of Pawi Special District in Benishangul-Gumuz Region, Northwestern Ethiopia. Ethiopian Journal of Health Development 24. <https://doi.org/10.4314/ejhd.v24i3.68387>

Till, C., Green, R., Grundy, J.G., Hornung, R., Neufeld, R., Martinez-Mier, E.A., Ayotte, P., Muckle, G., Lanphear, B., 2018. Community Water Fluoridation and Urinary Fluoride Concentrations in a National Sample of Pregnant Women in Canada. Environ Health Perspect 126, 107001. <https://doi.org/10.1289/EHP3546>

Tobias, R., 2016. Communication About Micropollutants in Drinking Water: Effects of the Presentation and Psychological Processes: Communication About Micropollutants. Risk Analysis 36, 2011–2026. <https://doi.org/10.1111/risa.12485>

Tofail, F., Vahter, M., Hamadani, J.D., Nermell, B., Huda, S.N., Yunus, M., Rahman, M., Grantham-McGregor, S.M., 2009. Effect of arsenic exposure during pregnancy on infant development at 7 months in rural Matlab, Bangladesh. Environ Health Perspect 117, 288–293. <https://doi.org/10.1289/ehp.11670>

Tokunaga, H., Roychowdhury, T., Uchino, T., Ando, M., 2005. Urinary arsenic species in an arsenic-affected area of West Bengal, India (part III). Applied Organometallic Chemistry 19, 246–253. <https://doi.org/10.1002/aoc.791>

Tomberge, V.M.J., Bischof, J.S., Meierhofer, R., Shrestha, A., Inauen, J., 2021. The Physical Burden of Water Carrying and Women’s Psychosocial Well-Being: Evidence from Rural Nepal. Int J Environ Res Public Health 18, 7908. <https://doi.org/10.3390/ijerph18157908>

Tondel, M., Rahman, M., Magnuson, A., Chowdhury, I.A., Faruquee, M.H., Ahmad, S.A., 1999. The relationship of arsenic levels in drinking water and the prevalence rate of skin lesions in Bangladesh. Environ Health Perspect 107, 727–729.

Toole, M.J., Claridge, F., Anderson, D.A., Zhuang, H., Morgan, C., Otto, B., Stewart, T., 2006. Hepatitis E virus infection as a marker for contaminated community drinking water sources in Tibetan villages. Am J Trop Med Hyg 74, 250–254.

Tran, H.H., Bjune, G., Nguyen, B.M., Rottingen, J.A., Grais, R.F., Guerin, P.J., 2005. Risk factors associated with typhoid fever in Son La province, northern Vietnam. Trans R Soc Trop Med Hyg 99, 819–826. <https://doi.org/10.1016/j.trstmh.2005.05.007>

Trevett, A.F., Carter, R.C., Tyrrel, S.F., 2005. Mechanisms leading to post-supply water quality deterioration in rural Honduran communities. Int J Hyg Environ Health 208, 153–161. <https://doi.org/10.1016/j.ijheh.2005.01.024>

Trinies, V., Freeman, M.C., Hennink, M., Clasen, T., 2011. The role of social networks on the uptake of household water filters by women in self-help groups in rural India. Journal of Water, Sanitation and Hygiene for Development 1, 224–232. <http://dx.doi.org/10.2166/washdev.2011.127>

Trudeau, J., Aksan, A.-M., Vásquez, W.F., 2018. Water system unreliability and diarrhea incidence among children in Guatemala. Int J Public Health 63, 241–250. <https://doi.org/10.1007/s00038-017-1054-6>

Trudel, D., Horowitz, L., Wormuth, M., Scheringer, M., Cousins, I.T., Hungerbühler, K., 2008. Estimating consumer exposure to PFOS and PFOA. Risk Anal 28, 251–269. <https://doi.org/10.1111/j.1539-6924.2008.01017.x>

Tsai, C.-F., Lin, D.-B., Chen, S.-C., Chang, Y.-H., Chen, C.-Y., Lin, J.-B., 2011. Seroepidemiology of hepatitis A virus infection among schoolchildren in Taiwan. J Med Virol 83, 196–200. <https://doi.org/10.1002/jmv.22000>

Tsai, S.-M., 1998. Cancer Mortality Trends in a Blackfoot Disease Endemic Community of Taiwan Following Water Source Replacement. Journal of Toxicology and Environmental Health, Part A 55, 389–404. <https://doi.org/10.1080/009841098158322>

Tsai, S.M., Wang, T.N., Ko, Y.C., 1999. Mortality for certain diseases in areas with high levels of arsenic in drinking water. Arch Environ Health 54, 186–193. <https://doi.org/10.1080/00039899909602258>

Tsai, Y.-S., Yang, W.-H., Tong, Y.-C., Lin, J.S.N., Pan, C.-C., Tzai, T.-S., 2005. Experience with primary urethral carcinoma from the blackfoot disease-endemic area of South Taiwan: increased frequency of bulbomembranous adenocarcinoma? Urol Int 74, 229–234. <https://doi.org/10.1159/000083554>

Tseng, H.-P., Wang, Y.-H., Wu, M.-M., The, H.-W., Chiou, H.-Y., Chen, C.-J., 2006. Association between chronic exposure to arsenic and slow nerve conduction velocity among adolescents in Taiwan. J Health Popul Nutr 24, 182–189.

Tuthill, R.W., Calabrese, E.J., 1985. The Massachusetts Blood Pressure Study, Part 2. Modestly elevated levels of sodium in drinking water and blood pressure levels in high school students. Toxicol Ind Health 1, 11–17. <https://doi.org/10.1177/074823378500100102>

Ueki, A., Otsuka, M., 2004. Life style risks of Parkinson’s disease: association between decreased water intake and constipation. J Neurol 251 Suppl 7, vII18-23. <https://doi.org/10.1007/s00415-004-1706-3>

Ülker, K., Çiçek, M., 2013. Effect of maternal hydration on the amniotic fluid volume during maternal rest in the left lateral decubitus position: a randomized prospective study. J Ultrasound Med 32, 955–961. <https://doi.org/10.7863/ultra.32.6.955>

Ulman, C., Gezer, S., Anal, Ö., Töre, I.R., Kirca, Ü., 1998. Arsenic in Human and Cow’s Milk: a Reflection of Environmental Pollution. Water, Air and Soil Pollution 101, 411–416. <https://doi.org/10.1023/A:1004990721068>

Unisa, S., Jagannath, P., Dhir, V., Khandelwal, C., Sarangi, L., Roy, T.K., 2011. Population-based study to estimate prevalence and determine risk factors of gallbladder diseases in the rural Gangetic basin of North India. HPB (Oxford) 13, 117–125. <https://doi.org/10.1111/j.1477-2574.2010.00255.x>

Uwimpuhwe, M., Reddy, P., Barratt, G., Bux, F., 2014. The impact of hygiene and localised treatment on the quality of drinking water in Masaka, Rwanda. J Environ Sci Health A Tox Hazard Subst Environ Eng 49, 434–440. <https://doi.org/10.1080/10934529.2014.854674>

Vahter, M., Concha, G., Nermell, B., Nilsson, R., Dulout, F., Natarajan, A.T., 1995. A unique metabolism of inorganic arsenic in native Andean women. European Journal of Pharmacology: Environmental Toxicology and Pharmacology 293, 455–462. <https://doi.org/10.1016/0926-6917(95)90066-7>

Vahter, M.E., Li, L., Nermell, B., Rahman, A., El Arifeen, S., Rahman, M., Persson, L.A., Ekström, E.-C., 2006. Arsenic exposure in pregnancy: a population-based study in Matlab, Bangladesh. J Health Popul Nutr 24, 236–245.

Valcke, M., Krishnan, K., 2010. An assessment of the interindividual variability of internal dosimetry during multi-route exposure to drinking water contaminants. Int J Environ Res Public Health 7, 4002–4022. <https://doi.org/10.3390/ijerph7114002>

Valenzuela, O.L., Germolec, D.R., Borja-Aburto, V.H., Contreras-Ruiz, J., García-Vargas, G.G., Del Razo, L.M., 2007. Chronic arsenic exposure increases TGFalpha concentration in bladder urothelial cells of Mexican populations environmentally exposed to inorganic arsenic. Toxicol Appl Pharmacol 222, 264–270. <https://doi.org/10.1016/j.taap.2006.12.015>

van Eijk, A.M., Lindblade, K.A., Odhiambo, F., Peterson, E., Rosen, D.H., Karanja, D., Ayisi, J.G., Shi, Y.P., Adazu, K., Slutsker, L., 2009. Geohelminth Infections among Pregnant Women in Rural Western Kenya; a Cross-Sectional Study. PLoS Negl Trop Dis 3, e370. <https://doi.org/10.1371/journal.pntd.0000370>

van Geen, A., Ahmed, K.M., Seddique, A.A., Shamsudduha, M., 2003. Community wells to mitigate the arsenic crisis in Bangladesh. Bull World Health Organ 81, 632–638.

van Loon, A., Botterweck, A., Goldbohm, R., Brants, H., van Klaveren, J., van den Brandt, P., 1998. Intake of nitrate and nitrite and the risk of gastric cancer: a prospective cohort study. Br J Cancer 78, 129–135. <https://doi.org/10.1038/bjc.1998.454>

van Maanen, J.M., Welle, I.J., Hageman, G., Dallinga, J.W., Mertens, P.L., Kleinjans, J.C., 1996. Nitrate contamination of drinking water: relationship with HPRT variant frequency in lymphocyte DNA and urinary excretion of N-nitrosamines. Environ Health Perspect 104, 522–528. <https://doi.org/10.1289/ehp.96104522>

Varsányi, I., Fodré, Z., Bartha, A., 1991. Arsenic in drinking water and mortality in the Southern Great Plain, Hungary. Environ Geochem Health 13, 14–22. <https://doi.org/10.1007/BF01783491>

Venkaiah, K., Meshram, I., Kodavanti, M., Chitty, G.R., Manchala, R., Kumar, S., Kakani, S., Laxmaiah, A., Brahmam, G., 2015. Nutrition profile of under-five year rural children and correlates of undernutrition in central India. Indian Journal of Community Health 27, 486–496.

Verma, R., Singh, A., Khurana, A., Dixit, P., Singh, R., 2017. Practices and attitudinal behavior about drinking water in an urban slum of district Rohtak, Haryana: A community-based study. J Family Med Prim Care 6, 554–557. <https://doi.org/10.4103/2249-4863.222030>

Vieux, F., Maillot, M., Constant, F., Drewnowski, A., 2017. Water and beverage consumption patterns among 4 to 13-year-old children in the United Kingdom. BMC Public Health 17, 479. <https://doi.org/10.1186/s12889-017-4400-y>

Vigeh, M., Yokoyama, K., Matsukawa, T., Shinohara, A., Ohtani, K., 2015. The relation of maternal blood arsenic to anemia during pregnancy. Women Health 55, 42–57. <https://doi.org/10.1080/03630242.2014.972016>

Villanueva, C.M., Cantor, K.P., Cordier, S., Jaakkola, J.J.K., King, W.D., Lynch, C.F., Porru, S., Kogevinas, M., 2004. Disinfection byproducts and bladder cancer: a pooled analysis. Epidemiology 15, 357–367. <https://doi.org/10.1097/01.ede.0000121380.02594.fc>

Villanueva, C.M., Cantor, K.P., Grimalt, J.O., Castaño-Vinyals, G., Malats, N., Silverman, D., Tardon, A., Garcia-Closas, R., Serra, C., Carrato, A., Rothman, N., Real, F.X., Dosemeci, M., Kogevinas, M., 2006. Assessment of lifetime exposure to trihalomethanes through different routes. Occup Environ Med 63, 273–277. <https://doi.org/10.1136/oem.2005.023069>

Villanueva, C.M., Gagniere, B., Monfort, C., Nieuwenhuijsen, M.J., Cordier, S., 2007. Sources of variability in levels and exposure to trihalomethanes. Environ Res 103, 211–220. <https://doi.org/10.1016/j.envres.2006.11.001>

Villanueva, C.M., Gracia-Lavedan, E., Bosetti, C., Righi, E., Molina, A.J., Martín, V., Boldo, E., Aragonés, N., Perez-Gomez, B., Pollan, M., Acebo, I.G., Altzibar, J.M., Zabala, A.J., Ardanaz, E., Peiró, R., Tardón, A., Chirlaque, M.D., Tavani, A., Polesel, J., Serraino, D., Pisa, F., Castaño-Vinyals, G., Espinosa, A., Espejo-Herrera, N., Palau, M., Moreno, V., La Vecchia, C., Aggazzotti, G., Nieuwenhuijsen, M.J., Kogevinas, M., 2017. Colorectal Cancer and Long-Term Exposure to Trihalomethanes in Drinking Water: A Multicenter Case-Control Study in Spain and Italy. Environ Health Perspect 125, 56–65. <https://doi.org/10.1289/EHP155>

Vinceti, M., Ballotari, P., Steinmaus, C., Malagoli, C., Luberto, F., Malavolti, M., Rossi, P.G., 2016. Long-term mortality patterns in a residential cohort exposed to inorganic selenium in drinking water. Environ Res 150, 348–356. <https://doi.org/10.1016/j.envres.2016.06.009>

Vinceti, M., Cann, C.I., Calzolari, E., Vivoli, R., Garavelli, L., Bergomi, M., 2000a. Reproductive outcomes in a population exposed long-term to inorganic selenium via drinking water. Sci Total Environ 250, 1–7. <https://doi.org/10.1016/s0048-9697(99)00419-2>

Vinceti, M., Fantuzzi, G., Monici, L., Cassinadri, M., Predieri, G., Aggazzotti, G., 2004. A retrospective cohort study of trihalomethane exposure through drinking water and cancer mortality in northern Italy. Sci Total Environ 330, 47–53. <https://doi.org/10.1016/j.scitotenv.2004.02.025>

Vinceti, M., Nacci, G., Rocchi, E., Cassinadri, T., Vivoli, R., Marchesi, C., Bergomi, M., 2000b. Mortality in a population with long-term exposure to inorganic selenium via drinking water. J Clin Epidemiol 53, 1062–1068. <https://doi.org/10.1016/s0895-4356(00)00233-x>

Vinceti, M., Rovesti, S., Gabrielli, C., Marchesi, C., Bergomi, M., Martini, M., Vivoli, G., 1995. Cancer mortality in a residential cohort exposed to environmental selenium through drinking water. J Clin Epidemiol 48, 1091–1097. <https://doi.org/10.1016/0895-4356(95)00014-u>

Vinceti, M., Rovesti, S., Marchesi, C., Bergomi, M., Vivoli, G., 1994. Changes in drinking water selenium and mortality for coronary disease in a residential cohort. Biol Trace Elem Res 40, 267–275. <https://doi.org/10.1007/BF02950799>

Vishnupriya, S., Prasad, S., Kasav, J.B., Trout, K., Murthy, S., Surapaneni, K.M., Joshi, A., 2014. Water and sanitation hygiene knowledge, attitudes and practices among school settings in rural Chennai. Journal of Water, Sanitation and Hygiene for Development 5, 192–200. <https://doi.org/10.2166/washdev.2014.052>

Vivek, R., Nihal, L., Illiayaraja, J., Reddy, P.K., Sarkar, R., Eapen, C.E., Kang, G., 2010. Investigation of an epidemic of Hepatitis E in Nellore in south India. Trop Med Int Health 15, 1333–1339. <https://doi.org/10.1111/j.1365-3156.2010.02624.x>

Vogt, L.E., Rukooko, B., Iversen, P.O., Eide, W.B., 2016. Human rights dimensions of food, health and care in children’s homes in Kampala, Uganda - a qualitative study. BMC Int Health Hum Rights 16, 10. <https://doi.org/10.1186/s12914-016-0086-y>

Volkmer, B.G., Ernst, B., Simon, J., Kuefer, R., Bartsch, G., Bach, D., Gschwend, J.E., 2005. Influence of nitrate levels in drinking water on urological malignancies: a community-based cohort study. BJU Int 95, 972–976. <https://doi.org/10.1111/j.1464-410X.2005.05450.x>

Vollaard, A.M., Ali, S., van Asten, H.A.G.H., Widjaja, S., Visser, L.G., Surjadi, C., van Dissel, J.T., 2004. Risk factors for typhoid and paratyphoid fever in Jakarta, Indonesia. JAMA 291, 2607–2615. <https://doi.org/10.1001/jama.291.21.2607>

von Ehrenstein, O.S., Guha Mazumder, D.N., Hira-Smith, M., Ghosh, N., Yuan, Y., Windham, G., Ghosh, A., Haque, R., Lahiri, S., Kalman, D., Das, S., Smith, A.H., 2006. Pregnancy outcomes, infant mortality, and arsenic in drinking water in West Bengal, India. Am J Epidemiol 163, 662–669. <https://doi.org/10.1093/aje/kwj089>

von Ehrenstein, O.S., Mazumder, D.N.G., Yuan, Y., Samanta, S., Balmes, J., Sil, A., Ghosh, N., Hira-Smith, M., Haque, R., Purushothamam, R., Lahiri, S., Das, S., Smith, A.H., 2005. Decrements in lung function related to arsenic in drinking water in West Bengal, India. Am J Epidemiol 162, 533–541. <https://doi.org/10.1093/aje/kwi236>

Waller, K., Swan, S.H., DeLorenze, G., Hopkins, B., 1998. Trihalomethanes in drinking water and spontaneous abortion. Epidemiology 9, 134–140.

Walsh, M., Wallner, G., Jennings, P., 2014. Radioactivity in drinking water supplies in Western Australia. Journal of Environmental Radioactivity 130, 56–62. <https://doi.org/10.1016/j.jenvrad.2013.12.016>

Wan, Y., Xia, W., Yang, S., Pan, X., He, Z., Kannan, K., 2018. Spatial distribution of bisphenol S in surface water and human serum from Yangtze River watershed, China: Implications for exposure through drinking water. Chemosphere 199, 595–602. <https://doi.org/10.1016/j.chemosphere.2018.02.040>

Wang, H.C., Liu, C., He, H.Y., Wang, M.X., 2015. A case-control study on the risk factors of urinary calculus in Uyghur children in the Kashi region. Genet Mol Res 14, 5862–5869. <https://doi.org/10.4238/2015.June.1.3>

Wang, N., Chen, C., Nie, X., Han, B., Li, Q., Chen, Yi, Zhu, C., Chen, Yingchao, Xia, F., Cang, Z., Lu, M., Meng, Y., Zhai, H., Lin, D., Cui, S., Jensen, M.D., Lu, Y., 2015. Blood lead level and its association with body mass index and obesity in China - Results from SPECT-China study. Sci Rep 5, 18299. <https://doi.org/10.1038/srep18299>

Wang, S.-L., Chiou, J.-M., Chen, C.-J., Tseng, C.-H., Chou, W.-L., Wang, C.-C., Wu, T.-N., Chang, L.W., 2003. Prevalence of non-insulin-dependent diabetes mellitus and related vascular diseases in southwestern arseniasis-endemic and nonendemic areas in Taiwan. Environ Health Perspect 111, 155–159. <https://doi.org/10.1289/ehp.5457>

Wang, W., Ye, B., Yang, L., Li, Y., Wang, Y., 2007. Risk assessment on disinfection by-products of drinking water of different water sources and disinfection processes. Environment International 33, 219–225. <https://doi.org/10.1016/j.envint.2006.09.009>

Wang, W.-L., Wu, Q.-Y., Wang, C., He, T., Hu, H.-Y., 2015. Health risk assessment of phthalate esters (PAEs) in drinking water sources of China. Environ Sci Pollut Res Int 22, 3620–3630. <https://doi.org/10.1007/s11356-014-3615-z>

Wang, Y., Zhu, G., Engel, B., Wu, Y., 2020. Probabilistic human health risk assessment of arsenic under uncertainty in drinking water sources in Jiangsu Province, China. Environ Geochem Health 42, 2023–2037. <https://doi.org/10.1007/s10653-019-00476-3>

Wang, Y.-H., Wu, M.-M., Hong, C.-T., Lien, L.-M., Hsieh, Y.-C., Tseng, H.-P., Chang, S.-F., Su, C.-L., Chiou, H.-Y., Chen, C.-J., 2007. Effects of arsenic exposure and genetic polymorphisms of p53, glutathione S-transferase M1, T1, and P1 on the risk of carotid atherosclerosis in Taiwan. Atherosclerosis 192, 305–312. <https://doi.org/10.1016/j.atherosclerosis.2006.07.029>

Wanigasuriya, K.P., Peiris-John, R.J., Wickremasinghe, R., 2011. Chronic kidney disease of unknown aetiology in Sri Lanka: is cadmium a likely cause? BMC Nephrology 12, 32. <https://doi.org/10.1186/1471-2369-12-32>

Ward, M.H., Cantor, K.P., Riley, D., Merkle, S., Lynch, C.F., 2003. Nitrate in public water supplies and risk of bladder cancer. Epidemiology 14, 183–190. <https://doi.org/10.1097/01.EDE.0000050664.28048.DF>

Ward, M.H., Kilfoy, B.A., Weyer, P.J., Anderson, K.E., Folsom, A.R., Cerhan, J.R., 2010. Nitrate intake and the risk of thyroid cancer and thyroid disease. Epidemiology 21, 389–395. <https://doi.org/10.1097/EDE.0b013e3181d6201d>

Ward, M.H., Weyer, P., Wang, A., Cerhan, J.R., 2007. Nitrate From Drinking Water and Diet and Thyroid Disorders and Thyroid Cancer Among Women. Epidemiology 18, S169. <https://doi.org/10.1097/01.ede.0000276851.08147.de>

Watanabe, C., Inaoka, T., Kadono, T., Nagano, M., Nakamura, S., Ushijima, K., Murayama, N., Miyazaki, K., Ohtsuka, R., 2001. Males in rural Bangladeshi communities are more susceptible to chronic arsenic poisoning than females: analyses based on urinary arsenic. Environ Health Perspect 109, 1265–1270. <https://doi.org/10.1289/ehp.011091265>

Watanabe, C., Kawata, A., Sudo, N., Sekiyama, M., Inaoka, T., Bae, M., Ohtsuka, R., 2004. Water intake in an Asian population living in arsenic-contaminated area. Toxicol Appl Pharmacol 198, 272–282. <https://doi.org/10.1016/j.taap.2003.10.024>

Watson, P.E., McDonald, B.W., 2014. Water and nutrient intake in pregnant New Zealand women: association with wheeze in their infants at 18 months. Asia Pac J Clin Nutr 23, 660–670. <https://doi.org/10.6133/apjcn.2014.23.4.13>

Watts, M.J., Middleton, D.R.S., Marriott, A.L., Humphrey, O.S., Hamilton, E.M., Gardner, A., Smith, M., McCormack, V.A., Menya, D., Munishi, M.O., Mmbaga, B.T., Osano, O., 2019. Source apportionment of micronutrients in the diets of Kilimanjaro,Tanzania and Counties of Western Kenya. Sci Rep 9, 14447. <https://doi.org/10.1038/s41598-019-51075-2>

Watts, S.J., 1986. The comparative study of patterns of guinea worm prevalence as a guide to control strategies. Soc Sci Med 23, 975–982. <https://doi.org/10.1016/0277-9536(86)90254-6>

Wei, B., Yu, J., Kong, C., Li, H., Yang, L., Xia, Y., Wu, K., 2018. A follow-up study of the development of skin lesions associated with arsenic exposure duration. Environ Geochem Health 40, 2729–2738. <https://doi.org/10.1007/s10653-018-0136-6>

Wei, B., Yu, J., Li, H., Yang, L., Xia, Y., Wu, K., Gao, J., Guo, Z., Cui, N., 2016. Arsenic Metabolites and Methylation Capacity Among Individuals Living in a Rural Area with Endemic Arseniasis in Inner Mongolia, China. Biol Trace Elem Res 170, 300–308. <https://doi.org/10.1007/s12011-015-0490-5>

Wei, B., Yu, J., Yang, L., Li, H., Chai, Y., Xia, Y., Wu, K., Gao, J., Guo, Z., Cui, N., 2017. Arsenic methylation and skin lesions in migrant and native adult women with chronic exposure to arsenic from drinking groundwater. Environ Geochem Health 39, 89–98. <https://doi.org/10.1007/s10653-016-9809-1>

Wei, B.G., Ye, B.X., Yu, J.P., Yang, L.S., Li, H.R., Xia, Y.J., Wu, K.G., 2017. Blood Pressure Associated with Arsenic Methylation and Arsenic Metabolism Caused by Chronic Exposure to Arsenic in Tube Well Water. Biomed Environ Sci 30, 334–342. <https://doi.org/10.3967/bes2017.044>

Welch, B.M., Branscum, A., Ahmed, S.M., Hystad, P., Smit, E., Afroz, S., Megowan, M., Golam, M., Ibne Hasan, M.O.S., Rahman, M.L., Quamruzzaman, Q., Christiani, D.C., Kile, M.L., 2019. Arsenic exposure and serum antibody concentrations to diphtheria and tetanus toxoid in children at age 5: A prospective birth cohort in Bangladesh. Environ Int 127, 810–818. <https://doi.org/10.1016/j.envint.2019.04.015>

Wen, L.M., Flood, V.M., Simpson, J.M., Rissel, C., Baur, L.A., 2010. Dietary behaviours during pregnancy: findings from first-time mothers in southwest Sydney, Australia. International Journal of Behavioral Nutrition and Physical Activity 7, 13. <https://doi.org/10.1186/1479-5868-7-13>

Wesselink, A.K., Hatch, E.E., Wise, L.A., Rothman, K.J., Vieira, V.M., Aschengrau, A., 2018. Exposure to tetrachloroethylene-contaminated drinking water and time to pregnancy. Environ Res 167, 136–143. <https://doi.org/10.1016/j.envres.2018.07.012>

Westrell, T., Andersson, Y., Stenström, T.A., 2006. Drinking water consumption patterns in Sweden. J Water Health 4, 511–522.

Weyer, P.J., Cerhan, J.R., Kross, B.C., Hallberg, G.R., Kantamneni, J., Breuer, G., Jones, M.P., Zheng, W., Lynch, C.F., 2001. Municipal drinking water nitrate level and cancer risk in older women: the Iowa Women’s Health Study. Epidemiology 12, 327–338. <https://doi.org/10.1097/00001648-200105000-00013>

Whitaker, H.J., Nieuwenhuijsen, M.J., Best, N.G., 2003. The relationship between water concentrations and individual uptake of chloroform: a simulation study. Environ Health Perspect 111, 688–694. <https://doi.org/10.1289/ehp.5963>

Wilkens, L.R., Kadir, M.M., Kolonel, L.N., Nomura, A.M., Hankin, J.H., 1996. Risk factors for lower urinary tract cancer: the role of total fluid consumption, nitrites and nitrosamines, and selected foods. Cancer Epidemiol Biomarkers Prev 5, 161–166.

Wilkins, J.R., III, Comstock, G.W., 1981. Source of drinking water at home and site-specific cancer incidence in Washington County, Maryland. American Journal of Epidemiology 114, 178–190. <https://doi.org/10.1093/oxfordjournals.aje.a113181>

Windham, G.C., Swan, S.H., Fenster, L., Neutra, R.R., 1992. Tap or bottled water consumption and spontaneous abortion: a 1986 case-control study in California. Epidemiology 3, 113–119. <https://doi.org/10.1097/00001648-199203000-00008>

Windham, G.C., Waller, K., Anderson, M., Fenster, L., Mendola, P., Swan, S., 2003. Chlorination by-products in drinking water and menstrual cycle function. Environ Health Perspect 111, 935–941; discussion A409. <https://doi.org/10.1289/ehp.5922>

Winterbottom, E.F., Ban, Y., Sun, X., Capobianco, A.J., Marsit, C.J., Chen, X., Wang, L., Karagas, M.R., Robbins, D.J., 2019. Transcriptome-wide analysis of changes in the fetal placenta associated with prenatal arsenic exposure in the New Hampshire Birth Cohort Study. Environ Health 18, 100. <https://doi.org/10.1186/s12940-019-0535-x>

Winterbottom, E.F., Fei, D.L., Koestler, D.C., Giambelli, C., Wika, E., Capobianco, A.J., Lee, E., Marsit, C.J., Karagas, M.R., Robbins, D.J., 2015. GLI3 Links Environmental Arsenic Exposure and Human Fetal Growth. EBioMedicine 2, 536–543. <https://doi.org/10.1016/j.ebiom.2015.04.019>

Witmans, M.R., McDuffie, H.H., Karunanayake, C., Kerrich, R., Pahwa, P., 2008. An exploratory study of chemical elements in drinking water and non-Hodgkin’s lymphoma. Toxicological & Environmental Chemistry 90, 1227–1247. <https://doi.org/10.1080/02772240801937370>

Wolka, E., Shiferaw, S., Biadgilign, S., 2014. Epidemiological study of risk factors for goiter among primary schoolchildren in southern Ethiopia. Food Nutr Bull 35, 20–27. <https://doi.org/10.1177/156482651403500103>

Wones, R.G., Deck, C.C., Stadler, B., Roark, S., Hogg, E., Frohman, L.A., 1993. Lack of effect of drinking water chlorine on lipid and thyroid metabolism in healthy humans. Environ Health Perspect 99, 375–381.

Wongsasuluk, P., Chotpantarat, S., Siriwong, W., Robson, M., 2018. Using hair and fingernails in binary logistic regression for bio-monitoring of heavy metals/metalloid in groundwater in intensively agricultural areas, Thailand. Environ Res 162, 106–118. <https://doi.org/10.1016/j.envres.2017.11.024>

Wood, S., Foster, J., Kols, A., 2012. Understanding why women adopt and sustain home water treatment: insights from the Malawi antenatal care program. Soc Sci Med 75, 634–642. <https://doi.org/10.1016/j.socscimed.2011.09.018>

Wrensch, M., Swan, S., Lipscomb, J., Epstein, D., Fenster, L., Claxton, K., Murphy, P.J., Shusterman, D., Neutra, R., 1990. Pregnancy outcomes in women potentially exposed to solvent-contaminated drinking water in San Jose, California. Am J Epidemiol 131, 283–300. <https://doi.org/10.1093/oxfordjournals.aje.a115498>

Wrensch, M., Swan, S.H., Lipscomb, J., Epstein, D.M., Neutra, R.R., Fenster, L., 1992. Spontaneous abortions and birth defects related to tap and bottled water use, San Jose, California, 1980-1985. Epidemiology 3, 98–103. <https://doi.org/10.1097/00001648-199203000-00006>

Wright, C.J., Sargeant, J.M., Edge, V.L., Ford, J.D., Farahbakhsh, K., Shiwak, I., Flowers, C., Gordon, A.C., RICG, IHACC Research Team, Harper, S.L., 2018. How are perceptions associated with water consumption in Canadian Inuit? A cross-sectional survey in Rigolet, Labrador. Sci Total Environ 618, 369–378. <https://doi.org/10.1016/j.scitotenv.2017.10.255>

Wu, F., Chi, L., Ru, H., Parvez, F., Slavkovich, V., Eunus, M., Ahmed, A., Islam, T., Rakibuz-Zaman, M., Hasan, R., Sarwar, G., Graziano, J.H., Ahsan, H., Lu, K., Chen, Y., 2018. Arsenic Exposure from Drinking Water and Urinary Metabolomics: Associations and Long-Term Reproducibility in Bangladesh Adults. Environ Health Perspect 126, 017005. <https://doi.org/10.1289/EHP1992>

Wu, J., Yunus, M., Streatfield, P.K., van Geen, A., Escamilla, V., Akita, Y., Serre, M., Emch, M., 2011. Impact of tubewell access and tubewell depth on childhood diarrhea in Matlab, Bangladesh. Environ Health 10, 109. <https://doi.org/10.1186/1476-069X-10-109>

Wu, W., Zhou, F., Wang, Y., Ning, Y., Yang, J.-Y., Zhou, Y.-K., 2017. Phthalate levels and related factors in children aged 6-12 years. Environ Pollut 220, 990–996. <https://doi.org/10.1016/j.envpol.2016.11.049>

Xiang, Q., Chen, L., Xd, C., Wang, C., Liang, Y., Liao, Q., Fan, D., Hong, P., Zhang, M., 2005. Serum fluoride and skeletal fluorosis in two villages in Jiangsu Province, China. Fluoride Research report Fluoride 3838, 178–184.

Xinwei, L., Xiaolan, Z., 2004. Study of the radon concentrations in drinking water from three main cities of Shaanxi Province, China. <https://doi.org/10.1007/S00254-004-0966-7>

Xu, P., Huang, S., Wang, Z., Lagos, G., 2006a. Daily intakes of copper, zinc and arsenic in drinking water by population of Shanghai, China. Sci Total Environ 362, 50–55. <https://doi.org/10.1016/j.scitotenv.2005.05.022>

Xu, P., Lagos, G., Huang, S., Wang, Z., 2006b. Summer Exposure Assessment of Cu and Zn in Drinking Water in Shanghai, China. Journal of Environmental Science and Health, Part A 41, 2465–2481. <https://doi.org/10.1080/10934520600927419>

Xu, X., Niu, T., Christiani, D.C., Weiss, S.T., Zhou, Y., Chen, C., Yang, J., Fang, Z., Jiang, Z., Liang, W., Zhang, F., 1997. Environmental and occupational determinants of blood pressure in rural communities in China. Annals of Epidemiology 7, 95–106.

Yadav, D., Tamrakar, D., Baral, R., Jha, P., Gautam, S., Pokharel, P., 2014. Outbreak of Cholera in Tilathi VDC Saptari Nepal. Kathmandu Univ. Med. J. 10, 36–39. <https://doi.org/10.3126/kumj.v10i4.10992>

Yallew, W.W., Terefe, M.W., Herchline, T.E., Sharma, H.R., Bitew, B.D., Kifle, M.W., Tetemke, D.M., Tefera, M.A., Adane, M.M., 2012. Assessment of water, sanitation, and hygiene practice and associated factors among people living with HIV/AIDS home based care services in Gondar city, Ethiopia. BMC Public Health 12, 1057. <https://doi.org/10.1186/1471-2458-12-1057>

Yan, Y.-Q., Dong, Z.-L., Dong, L., Wang, F.-R., Yang, X.-M., Jin, X.-Y., Lin, L.-X., Sun, Y.-N., Chen, Z.-P., 2011. Trimester- and method-specific reference intervals for thyroid tests in pregnant Chinese women: methodology, euthyroid definition and iodine status can influence the setting of reference intervals. Clin Endocrinol (Oxf) 74, 262–269. <https://doi.org/10.1111/j.1365-2265.2010.03910.x>

Yang, C.-Y., 2004. Drinking water chlorination and adverse birth outcomes in Taiwan. Toxicology 198, 249–254. <https://doi.org/10.1016/j.tox.2004.01.032>

Yang, C.-Y., Chang, C.-C., Ho, S.-C., Chiu, H.-F., 2008. Is colon cancer mortality related to arsenic exposure? J Toxicol Environ Health A 71, 533–538. <https://doi.org/10.1080/15287390801907509>

Yang, C.Y., Cheng, B.H., Tsai, S.S., Wu, T.N., Lin, M.C., Lin, K.C., 2000a. Association between chlorination of drinking water and adverse pregnancy outcome in Taiwan. Environ Health Perspect 108, 765–768. <https://doi.org/10.1289/ehp.00108765>

Yang, C.-Y., Chiu, H.-F., 1998. Calcium and magnesium in drinking water and risk of death from rectal cancer. International Journal of Cancer 77, 528–532. [https://doi.org/10.1002/(SICI)1097-0215(19980812)77:4<528::AID-IJC9>3.0.CO;2-W](https://doi.org/10.1002/(SICI)1097-0215(19980812)77:4%3c528::AID-IJC9%3e3.0.CO;2-W)

Yang, C.-Y., Chiu, H.-F., Chang, C.-C., Wu, T.-N., Sung, F.-C., 2002. Association of very low birth weight with calcium levels in drinking water. Environ Res 89, 189–194. <https://doi.org/10.1006/enrs.2002.4369>

Yang, C.Y., Chiu, H.F., Cheng, M.F., Hsu, T.Y., Cheng, M.F., Wu, T.N., 2000b. Calcium and magnesium in drinking water and the risk of death from breast cancer. J Toxicol Environ Health A 60, 231–241.

Yang, C.-Y., Xiao, Z.-P., Ho, S.-C., Wu, T.-N., Tsai, S.-S., 2007. Association between trihalomethane concentrations in drinking water and adverse pregnancy outcome in Taiwan. Environmental Research 104, 390–395. <https://doi.org/10.1016/j.envres.2007.01.006>

Yang, J., Yan, L., Zhang, M., Wang, Y., Wang, C., Xiang, Q., 2015. Associations between the polymorphisms of GSTT1, GSTM1 and methylation of arsenic in the residents exposed to low-level arsenic in drinking water in China. J Hum Genet 60, 387–394. <https://doi.org/10.1038/jhg.2015.39>

Yang, J., Zhao, Z., Li, Y., Krewski, D., Wen, S.W., 2009. A multi-level analysis of risk factors for Schistosoma japonicum infection in China. Int J Infect Dis 13, e407-412. <https://doi.org/10.1016/j.ijid.2009.02.005>

Yang, L., Chai, Y., Yu, J., Wei, B., Xia, Y., Wu, K., Gao, J., Guo, Z., Cui, N., 2017. Associations of arsenic metabolites, methylation capacity, and skin lesions caused by chronic exposure to high arsenic in tube well water. Environ Toxicol 32, 28–36. <https://doi.org/10.1002/tox.22209>

Yang, M.-H., Chen, K.-K., Yen, C.-C., Wang, W.-S., Chang, Y.-H., Huang, W.J.-S., Fan, F.S., Chiou, T.-J., Liu, J.-H., Chen, P.-M., 2002. Unusually high incidence of upper urinary tract urothelial carcinoma in Taiwan. Urology 59, 681–687. <https://doi.org/10.1016/s0090-4295(02)01529-7>

Yang, N., Wang, D., Xing, M., Li, C., Li, J., Wu, A., Sang, X., Feng, Y., Jiang, N., Chen, Q., 2017. Seroepidemiology and Risk Factors of Toxoplasma gondii Infection among the Newly Enrolled Undergraduates and Postgraduate Students in China. Front Microbiol 8, 2092. <https://doi.org/10.3389/fmicb.2017.02092>

Yasar, A., Khan, N.Y., Batool, A., Tabinda, A.B., Mehmood, R., Iqbal, A., 2011. Womens perception of water quality and its impacts on health in Gangapur, Pakistan. Pak J Nutr 10, 702–706.

Yasar, Abdullah, Khan, N.Y., Batool, A., Tabinda, A.B., Mehmood, R., Iqbal, A., 2011. Women Perception of Water Quality and its Impacts on Health in Gangapur, Pakistan. Pakistan Journal of Nutrition.

Yazbeck, C., Kloppmann, W., Cottier, R., Sahuquillo, J., Debotte, G., Huel, G., 2005. Health impact evaluation of boron in drinking water: a geographical risk assessment in Northern France. Environ Geochem Health 27, 419–427. <https://doi.org/10.1007/s10653-005-1796-6>

Yeh, T.-C., Tai, Y.-S., Pu, Y.-S., Chen, C.-H., 2015. Characteristics of arsenic-related bladder cancer: A study from Nationwide Cancer Registry Database in Taiwan. Urological Science 26, 103–108. <https://doi.org/10.1016/j.urols.2015.05.002>

Yentür Doni, N., Şimşek, Z., Gürses, G., Yıldız Zeyrek, F., Akbaba, M., 2017. The knowledge and high seroprevalence of hepatitis A in a high-risk group (agricultural reproductive-aged women) in the southeastern region of Turkey. Turk J Med Sci 47, 1055–1060. <https://doi.org/10.3906/sag-1505-22>

Ye-qing, X., Fu-qing, C., Jia-tong, Z., Guo-ming, Z., Jin-fa, D., Qu-yun, D., Hui-min, L., 2012. An outbreak of hepatitis A associated with a contaminated well in a middle school, Guangxi, China. Western Pac Surveill Response J 3, 44–47. <https://doi.org/10.5365/WPSAR.2012.3.4.014>

Yılmaz, B.K., Evliyaoğlu, Ö., Yorgancı, A., Özyer, Ş., Üstün, Y.E., 2020. Serum concentrations of heavy metals in women with endometrial polyps. J Obstet Gynaecol 40, 541–545. <https://doi.org/10.1080/01443615.2019.1634022>

Yohannes, A.G., Streatfield, K., Bost, L., 1992. Child morbidity patterns in Ethiopia. J Biosoc Sci 24, 143–155. <https://doi.org/10.1017/s0021932000019684>

Young, T.B., Kanarek, M.S., Tsiatis, A.A., 1981. Epidemiologic study of drinking water chlorination and Wisconsin female cancer mortality. J Natl Cancer Inst 67, 1191–1198.

Yousefi, M., Mohammadi, A.A., Yaseri, M., Mahvi, A.H., 2017. Epidemiology of drinking water fluoride and its contribution to fertility, infertility, and abortion: an ecological study in West Azerbaijan Province, Poldasht County, Iran. Research report 11.

Yousuf, F.A., Siddiqui, R., Subhani, F., Khan, N.A., 2013. Status of free-living amoebae (Acanthamoeba spp., Naegleria fowleri, Balamuthia mandrillaris) in drinking water supplies in Karachi, Pakistan. J Water Health 11, 371–375. <https://doi.org/10.2166/wh.2013.112>

Yuan, Y., Marshall, G., Ferreccio, C., Steinmaus, C., Liaw, J., Bates, M., Smith, A.H., 2010. Kidney cancer mortality: fifty-year latency patterns related to arsenic exposure. Epidemiology 21, 103–108. <https://doi.org/10.1097/EDE.0b013e3181c21e46>

Yuan, Y., Marshall, G., Ferreccio, C., Steinmaus, C., Selvin, S., Liaw, J., Bates, M.N., Smith, A.H., 2007. Acute myocardial infarction mortality in comparison with lung and bladder cancer mortality in arsenic-exposed region II of Chile from 1950 to 2000. Am J Epidemiol 166, 1381–1391. <https://doi.org/10.1093/aje/kwm238>

Zaida, F., Chadrame, S., Sedki, A., Lekouch, N., Bureau, F., Arhan, P., Bouglé, D., 2007. Lead and aluminium levels in infants’ hair, diet, and the local environment in the Moroccan city of Marrakech. Sci Total Environ 377, 152–158. <https://doi.org/10.1016/j.scitotenv.2006.10.017>

Zamora, M.L., Tracy, B.L., Zielinski, J.M., Meyerhof, D.P., Moss, M.A., 1998. Chronic Ingestion of Uranium in Drinking Water: A Study of Kidney Bioeffects in Humans. Toxicol Sci 43, 68–77. <https://doi.org/10.1093/toxsci/43.1.68>

Zanini, B., Ricci, C., Bandera, F., Caselani, F., Magni, A., Laronga, A.M., Lanzini, A., San Felice del Benaco Study Investigators, 2012. Incidence of post-infectious irritable bowel syndrome and functional intestinal disorders following a water-borne viral gastroenteritis outbreak. Am J Gastroenterol 107, 891–899. <https://doi.org/10.1038/ajg.2012.102>

Zaterka, S., Eisig, J.N., Chinzon, D., Rothstein, W., 2007. Factors related to Helicobacter pylori prevalence in an adult population in Brazil. Helicobacter 12, 82–88. <https://doi.org/10.1111/j.1523-5378.2007.00474.x>

Zeighami, E.A., Watson, A.P., Craun, G.F., 1990. Chlorination, Water Hardness and Serum Cholesterol in Forty-six Wisconsin Communities. International Journal of Epidemiology 19, 49–58. <https://doi.org/10.1093/ije/19.1.49>

Zender, R., Bachand, A.M., Reif, J.S., 2001. Exposure to tap water during pregnancy. J Expo Anal Environ Epidemiol 11, 224–230. <https://doi.org/10.1038/sj.jea.7500163>

Zeng, X.-J., Jiang, W.-S., Xie, S.-Y., Chen, Y.-D., Gu, X.-N., Ge, J., Hang, C.-Q., Li, Z.-J., Chen, H.-G., 2019. Effect of integrated control intervention on soil-transmitted helminth infections in Jiangxi province in southeast China. Acta Trop 194, 148–154. <https://doi.org/10.1016/j.actatropica.2019.04.001>

Zhai, Y., Zhao, X., Teng, Y., Li, X., Zhang, J., Wu, J., Zuo, R., 2017. Groundwater nitrate pollution and human health risk assessment by using HHRA model in an agricultural area, NE China. Ecotoxicol Environ Saf 137, 130–142. <https://doi.org/10.1016/j.ecoenv.2016.11.010>

Zhang, B., Zhou, A.-F., Zhu, C.-C., Zhang, L., Xiang, B., Chen, Z., Hu, R.-H., Zhang, Y.-Q., Qiu, L., Zhang, Y.-M., Xiong, C.-D., Du, Y.-K., Shi, Y.-Q., 2013. Risk factors for cervical cancer in rural areas of Wuhan China: a matched case-control study. Asian Pac J Cancer Prev 14, 7595–7600. <https://doi.org/10.7314/apjcp.2013.14.12.7595>

Zhang, C., Mao, G., He, S., Yang, Z., Yang, W., Zhang, X., Qiu, W., Ta, N., Cao, L., Yang, H., Guo, X., 2013. Relationship between long-term exposure to low-level arsenic in drinking water and the prevalence of abnormal blood pressure. J Hazard Mater 262, 1154–1158. <https://doi.org/10.1016/j.jhazmat.2012.09.045>

Zhang, H., Chang, S., Wang, L., Wang, W., 2018. Estimating and comparing the cancer risks from THMs and low-level arsenic in drinking water based on disability-adjusted life years. Water Res 145, 83–93. <https://doi.org/10.1016/j.watres.2018.08.012>

Zhang, H., Wu, M., Yang, L., Wu, J., Hu, Y., Han, J., Gu, Y., Li, X., Wang, H., Ma, L., Yang, X., 2019. Evaluation of median urinary iodine concentration cut-off for defining iodine deficiency in pregnant women after a long term USI in China. Nutr Metab (Lond) 16, 62. <https://doi.org/10.1186/s12986-019-0381-4>

Zhang, J., Xu, L.C., 2016. The long-run effects of treated water on education: The rural drinking water program in China. Journal of Development Economics 122, 1–15. <https://doi.org/10.1016/j.jdeveco.2016.04.004>

Zhang, W., Gabos, S., Schopflocher, D., Li, X.-F., Gati, W.P., Hrudey, S.E., 2009. Validation of urinary trichloroacetic acid as a biomarker of exposure to drinking water disinfection by-products. J Water Health 7, 359–371. <https://doi.org/10.2166/wh.2009.009>

Zhao, A., Cao, S., Gao, H., Xiao, Q., Win, N., Zhang, Y., 2016. Anemia Among Lactating Mothers in Kokang, Myanmar. Southeast Asian J Trop Med Public Health 47, 1298–1305.

Zhao, M.X., Zhou, G.Y., Zhu, J.Y., Gong, B., Hou, J.X., Zhou, T., Duan, L.J., Ding, Z., Cui, L.X., Ba, Y., 2015. Fluoride Exposure, Follicle Stimulating Hormone Receptor Gene Polymorphism and Hypothalamus-pituitary-ovarian Axis Hormones in Chinese Women. Biomed Environ Sci 28, 696–700. <https://doi.org/10.3967/bes2015.099>

Zhou, L., Turvey, C.G., 2018. Drinking water and off‐farm labour supply: between‐gender and within‐gender bias. Aust J Agric Resour Econ 62, 103–120. <https://doi.org/10.1111/1467-8489.12239>

Zhou, W., Zhao, S., Tong, C., Chen, L., Yu, X., Yuan, T., Aimuzi, R., Luo, F., Tian, Y., Zhang, J., Shanghai Birth Cohort study, 2019. Dietary intake, drinking water ingestion and plasma perfluoroalkyl substances concentration in reproductive aged Chinese women. Environ Int 127, 487–494. <https://doi.org/10.1016/j.envint.2019.03.075>

Zhu, H., Zhu, L., Yan, B., Guan, J., 2014. Accumulation of Lead, Zinc, and Copper in Scalp Hair of Residents in a Long-Term Irrigation Area Downstream of the Second Songhua River, Northeast China. Human and Ecological Risk Assessment: An International Journal 20, 137–149. <https://doi.org/10.1080/10807039.2012.740655>

Zhu, Y., Huang, B., Li, Q.X., Wang, J., 2015. Organochlorine pesticides in follicular fluid of women undergoing assisted reproductive technologies from central China. Environ Pollut 207, 266–272. <https://doi.org/10.1016/j.envpol.2015.09.030>

Zietz, B.P., Lass, J., Suchenwirth, R., 2007. Assessment and management of tap water lead contamination in Lower Saxony, Germany. Int J Environ Health Res 17, 407–418. <https://doi.org/10.1080/09603120701628719>

Zimeri, A.M., Robb, S.W., Hassan, S.M., Hire, R.R., Davis, M.B., 2015. Assessing Heavy Metal and PCB Exposure from Tap Water by Measuring Levels in Plasma from Sporadic Breast Cancer Patients, a Pilot Study. Int J Environ Res Public Health 12, 15683–15691. <https://doi.org/10.3390/ijerph121215013>

Zimoch, I., Lobos, E., 2016. Evaluation of health risk caused by chloroform in drinking water. Desalination and Water Treatment 57, 1027–1033. <https://doi.org/10.1080/19443994.2015.1033134>

Zincir, H., Kaya Erten, Z., Ozkan, F., Seviğ, U., Başer, M., Elmalı, F., 2012. Prevalence of urinary tract infections and its risk factors in elementary school students. Urol Int 88, 194–197. <https://doi.org/10.1159/000335554>

Zolnikov, T.R., Blodgett Salafia, E., 2016. Improved relationships in eastern Kenya from water interventions and access to water. Health Psychol 35, 273–280. <https://doi.org/10.1037/hea0000301>

Zou, S., Wu, F., Guo, C., Song, J., Huang, C., Zhu, Z., Yu, H., Guo, Y., Lu, X., Ruan, Y., 2012. Iodine nutrition and the prevalence of thyroid disease after salt iodization: a cross-sectional survey in Shanghai, a coastal area in China. PLoS One 7, e40718. <https://doi.org/10.1371/journal.pone.0040718>

Zou, Y.-F., Feng, C.-C., Zhu, J.-M., Tao, J.-H., Chen, G.-M., Ye, Q.-L., Cen, H., Leng, R.-X., Pan, F.-M., Pan, H.-F., Li, R., Fan, Y.-G., Wang, B., Li, X.-P., Zhang, F.-Y., Ye, D.-Q., 2014. Prevalence of systemic lupus erythematosus and risk factors in rural areas of Anhui Province. Rheumatol Int 34, 347–356. <https://doi.org/10.1007/s00296-013-2902-1>
